# Bottom-up synthesis of protein-based nanomaterials from engineered β-solenoid proteins

**DOI:** 10.1371/journal.pone.0229319

**Published:** 2020-02-21

**Authors:** Zeyu Peng, Maria D. R. Peralta, Daniel L. Cox, Michael D. Toney

**Affiliations:** 1 Department of Chemistry, University of California Davis, Davis, California, United States of America; 2 Department of Physics, University of California Davis, Davis, California, United States of America; University of South Carolina, UNITED STATES

## Abstract

Biomolecular self-assembly is an emerging bottom-up approach for the synthesis of novel nanomaterials. DNA and viruses have both been used to create scaffolds but the former lacks chemical diversity and the latter lack spatial control. To date, the use of protein scaffolds to template materials on the nanoscale has focused on amyloidogenic proteins that are known to form fibrils or two-protein systems where a second protein acts as a cross-linker. We previously developed a unique approach for self-assembly of nanomaterials based on engineering β-solenoid proteins (BSPs) to polymerize into micrometer-length fibrils. BSPs have highly regular geometries, tunable lengths, and flat surfaces that are amenable to engineering and functionalization. Here, we present a newly engineered BSP based on the antifreeze protein of the beetle *Rhagium inquisitor* (RiAFP-m9), which polymerizes into stable fibrils under benign conditions. Gold nanoparticles were used to functionalize the RiAFP-m9 fibrils as well as those assembled from the previously described SBAFP-m1 protein. Cysteines incorporated into the sequences provide site-specific gold attachment. Additionally, silver was deposited on the gold-labelled fibrils by electroless plating to create nanowires. These results bolster prospects for programable self-assembly of BSPs to create scaffolds for functional nanomaterials.

## Introduction

The bottom-up approach for synthesizing functional nanomaterials and devices, in which molecular building blocks are designed to assemble into nanostructures, is becoming both more attractive and more tractable. DNA-based nanostructured scaffolds (DNA "origami")[1] are now well developed and allow for highly ordered templating of nanoparticles.[2–4] However, the restricted chemistry of DNA bases limits facile derivatization; the attachment of nanoparticles to DNA scaffolds generally requires pre-functionalization. For example, nanoparticles have been functionalized with 3’ thiol-modified oligonucleotides[2, 3] or psoralen[5] to allow attachment to DNA. Moreover, DNA nanostructures are unstable to stress such as nucleases, temperatures above 60 °C[6] and pH extremes[7], although DNA can be carbonized to template growth of remarkably stable high temperature carbon nanostructures.[8] DNA nanotubes filled with amyloid fibrils have also been produced.[9] These composite self-assembled materials contain both protein and DNA, and the amyloid fibrils can be organized by DNA origami constructions.[9] Proteins are an attractive alternative for the synthesis of structure-controlled nanomaterials, but the pathway to them is not as simple as with DNA.

Proteins have chemically diverse amino acid side chains, which makes them excellent prospects for scaffolds with defined binding characteristics. Belcher and collaborators have used viruses (M13 bacteriophage) for self-assembly of different inorganic materials, using genetically modified viral coat proteins with material-specific binding peptides isolated by phage display.[10–13] However, because viruses are large and multivalent, and templating sites are restricted, they do not lend themselves to precise nanoscale templating of materials. Recently, artificial minimal viral coat proteins were reported to polymerize into rods on DNA templates, presenting an alternative.[14]

Recent reviews highlight applications of protein- and peptide-based fibers in medicine.[15, 16] Nanomaterials have been constructed with peptide fibrils, such as amyloid fibrils formed from amyloidogenic fragments of Aβ peptide[17–20] or an amyloidogenic peptide from Bgl2p–glucantransferase of yeast cell wall[21], self-assembled collagen-like peptide fibrils[22], and the low-complexity (LC) sequence domain of fused in sarcoma (FUS) protein[23, 24]. However, amyloid fibrils derived from peptides have low longitudinal amino acid diversity (i.e., a single peptide is the lengthwise repeat unit and therefore each rung of the fibril is identical), which limits application and functionalization of synthesized fibrils.

The synthesis of amyloid fibrils from nonamyloidogenic proteins requires harsh conditions in many cases, such as high temperatures[25] and/or acidic pH[26–28]. For materials purposes, alternative routes that employ mild conditions are highly desirable. The creation of protein scaffolds as materials templates has been previously reported, some using amyloidogenic proteins that are known to form fibrils[29, 30] and some using two-protein systems where a second protein acts as a cross-linker[31, 32]. The naturally filament-forming protein γ-prefoldin has been used to engineer self-assembling protein filaments that can be functionalized. [33] Impressive examples of non-fibrous protein-based materials have been engineered recently.[34–36] Here, a flexible alternative for engineering protein fibril-based nanoscale scaffolds under mild conditions, based on β-solenoid protein (BSPs), is discussed.

Amyloid fibrils are highly ordered peptide or protein polymers that have a cross-β structure, in which the peptide backbone runs perpendicular to the long axis of the fibril.[37] Amyloids are best known for causing diseases, such as Alzheimer’s and type II diabetes.[37] They are generally stable to high temperatures[38–40], organic solvents[39, 40], and protease digestion[40]. Engineered fibrils based on amyloid-structured BSPs, whose lengths are on the order of 5 nm, have the potential to generate complex nanostructured materials with excellent strength and structural control.

BSPs have a distinctive secondary structure in which the protein backbone forms a solenoid of β-sheet. Wild type BSPs have either capping structures or structural distortions at one or both termini, which prevents cross-β fibril formation via end-to-end polymerization. Self-assembly of engineered BSPs into micrometer-length fibrils has been demonstrated.[41] Long fibrils self-assemble from end-modified BSPs [41], which are geometrically regular (triangle, rectangle, *etc*.), and have tunable lengths and flat surfaces that can be chemically modified. These unique features of BSP fibrils have promise for programmable, protein-based, self-assembled scaffolds for functional materials with high stability. They combine the mechanical strength and stability that have made spider silk popular for materials applications, while affording an amenability to engineering beyond that of spider silk, because of the established sequence-to-structure relationship of BSPs.

Here, the antifreeze protein (AFP) from the beetle *Rhagium inquisitor* (PDB entry 4DT5)[42] was engineered (RiAFP-m9) to form linear fibrils. Three cysteines per monomer were introduced in the design process to facilitate the binding of gold nanoparticles (AuNPs), since the thiol group of cysteine has a strong interaction with gold.[43–46] Amyloid-gold functional hybrids have been previously produced. [47–49] A second engineered BSP from the spruce budworm (SBAFP-m1), previously shown to self-assemble into fibrils, was modified with an N-terminal 5×cysteine tag (SBAFP-CT) to enable AuNP binding. The two engineered BSPs polymerized into fibrils, which were subsequently labelled with AuNPs and transformed into metallic nanowires by electroless silver plating onto the AuNPs. These results show that self-assembled, polymeric BSPs have excellent potential for the bottom-up design of functional nanomaterials.

The demonstration that these two proteins can be used to create amyloid fibrils conjugated with AuNPs is an important step toward creating programmable and functional nanoscale scaffolds with high stability and tensile strength.[50, 51] Additionally, both wildtype proteins used as the basis for the design of RiAFP and SBAFP-CT are non-amyloidogenic proteins, proving it is possible using simple design concepts to create fibril-based materials systematically.[41]

## Materials and methods

### Materials

Thioflavin-T (ThT) was purchased from Sigma-Aldrich. Carbon coated nickel grids were purchased from Electron Microscopy Science. AuNPs (5 nm) were purchased from Sigma-Aldrich. Tris(2-carboxyethyl)phosphine (TCEP) hydrochloride was purchased from Sigma-Aldrich. AURION R-Gent SE-EM Kit used for electroless silver plating was purchased from Electron Microscopy Science.

### Molecular dynamics (MD) simulations

YASARA (v.15) was employed for MD simulations. The AMBER03 force field was used with explicit water. The “md_run” macro provided with the software was used to set up and run simulations automatically with a periodic boundary simulation box that was 10 Å larger than the protein on all sides. The RiAFP simulations were run at 100 °C for ~5 ns. The “md_analyze” macro was used to analyze trajectories.

### Expression and purification of recombinant proteins

The protein sequence for RiAFP-m9 is: MAHHHHHHSGSGSRAEARGEAMAEGHSRGCATSHANATGHADARSMSEGNAEAYTEAKGTAMATSEASGEARAQTNADGRAHSSSRTHGRADSTASAKGEAMAEGTSDGDAKSYASADGNACAKSMSTGHADATTNAHGTAMADSNAIGEARAETRAEGRAESSSDTDGC. The RiAFP-m9 gene was synthesized by Thermo Fisher Scientific as GeneArt DNA Strings and cloned into pET-28a by Gibson assembly.[52, 53] Protein was expressed in *E*. *coli* BL21 (DE3) cells. RiAFP-m9 cultures were grown at 37 °C with shaking at 250 rpm to OD_600_ = 0.5–0.6. To induce expression, IPTG was added to a final concentration of 1 mM. Four hours after adding IPTG to the culture, cells were collected by centrifugation (4,000 *g* for 20 min). The cells were resuspended in cold lysis buffer (50 mM Tris-HCl, 200 mM NaCl, 10 mM β-mercaptoethanol (BME), 10 mM imidazole, pH 8) and were lysed by sonication. Soluble and insoluble proteins were separated by centrifugation (10,000 *g* for 30 min). RiAFP-m9 was expressed in the soluble fraction and purified by nickel affinity chromatography. Proteins were eluted stepwise from the column with increasing concentrations of imidazole (20 mM increments from 30–250 mM). The fractions containing purified RiAFP-m9 were combined and the purified protein was stored at 4 °C. RiAFP-m9 at a concentration of 1 mg/mL was used for ESI-MS to verify its molecular weight. To promote fibril formation, purified protein (0.6 mg/mL in 10 mM sodium phosphate, pH 7.4) was incubated at 37 °C for 10 days.

The gene for SBAFP-CT was constructed using PCR, by adding codons for the sequence MCCCCCGG to the 5’ end of the SBAFP-m1 gene.[41] The sequence of SBAFP-CT is: MCCCCCGGASRITNSQIVKSEATNSDINNSQLVDSISTRSQYSDANVKKSVTTDSNIDKSQVYLTTSTGSQYNGIYIRSSDTTGSEISGSSISTSRITNSRITNSQIVKSEATNSDINNSQLVDSISTRSQYSDANVKKSVTTDSNIDKSQVYLTTSTGSQYNGIYIRSSDTTGSEISGSSISTSRIT. The gene was cloned into pET-28a by Gibson assembly.[52, 53] Cultures were induced with 1mM IPTG at OD_600_ = 0.8–0.9, shaken at 250 rpm at 30 °C for 3 hours, and harvested by centrifugation (3,000 *g* for 20 min). The protein was expressed in the insoluble fraction. Multiple rounds of sonication were performed on the inclusion bodies. For the first two rounds of sonication, the insoluble fraction was resuspended in lysis buffer (50 mM Tris-HCl, 100 mM NaCl, 5 mM EDTA, 0.5% Triton X-100, pH 8), while the last two rounds of sonication employed lysis buffer without Triton X-100. Insoluble fractions were collected by centrifugation (8,000 *g* for 30 min). SBAFP-CT was solubilized by stirring overnight at 4 °C in 100 mM Tris-HCl, 10 mM BME, 8 M urea, pH 8. SBAFP-CT was further purified by Q Sepharose anion exchange in the presence of 8 M urea. Protein was loaded onto the column in 100 mM Tris-HCl pH 8, 10 mM NaCl, 10 mM BME, 8 M urea, and eluted with an NaCl gradient from 10 mM to 500 mM. The urea was removed from the purified protein by stepwise dialysis into 10 mM sodium phosphate, pH 7.4. MALDI was performed on a 0.9 mg/mL sample in 10 mM sodium phosphate, pH 7.4. Purified, refolded SBAFP-CT at 0.9 mg/mL was incubated at 37 °C for 5 days to promote fibril formation.

### Circular dichroism (CD) spectra

CD spectra (190 nm to 300 nm) were taken with 0.6 mg/mL RiAFP-m9 and 0.2 mg/mL SBAFP-CT in 10 mM sodium phosphate, pH 7.4, in a 1 mm path length cell using an OLIS DSM 20 instrument at a scan rate proportional to high voltage. Each reported spectrum is an average of 3 scans. CDNN software was used for deconvolution of the CD spectra; the total secondary structure content was normalized to 100%.

### ThT fluorescence assay

A stock solution of ThT was made by dissolving ~2 mg of ThT in 2 mL of PBS, pH 7.4. The concentration of the stock solution was determined by diluting it 100-fold in PBS and measuring absorbance at 416 nm (ε = 26620 M^-1^ cm^-1^). A working solution was prepared from the stock solution by diluting in PBS to a final concentration of 500 μM. In the ThT assay, RiAFP-m9 was 18 μM and ThT was 36 μM in PBS, pH 7.4, while SBAFP-CT was 5 μM and ThT was 10 μM. ThT fluorescence emission spectra were recorded between 465 and 565 nm with excitation at 450 nm using a Horiba FuoroMax-P spectrofluorometer. Each reported spectrum is an average of 3 scans.

### Transmission electron microscopy (TEM) imaging

The protein sample (~10 μL) was loaded on a carbon coated nickel grid and incubated at room temperature for 2 min. The buffer was removed by filter paper and the sample was washed with deionized water twice for 1 min each. After the water was removed, the sample was stained with 2% uranyl acetate at room temperature for 30 s. Excess stain was removed by filter paper and the grid was dried at room temperature. The specimen was observed using a JOEL 1230 TEM with 100 kV of electron acceleration voltage.

### Gold labelling

Purified RiAFP-m9 was dialyzed into 10 mM sodium phosphate, 10 mM BME, pH 7.4. The protein was incubated at 37 °C for 10 days to form fibrils. After fibril formation, the protein was dialyzed to remove BME, and then the protein was diluted to 5 μM. A TCEP stock solution was prepared at a concentration of 200 mM in 10 mM sodium phosphate, pH 7.4. TCEP was added to RiAFP-m9 fibrils to give a final TCEP concentration of 70 μM. A 2 mL solution of 5 nm AuNPs was centrifuged at 13,000 *g* for 45 min. The supernatant was removed and the nanoparticles were resuspended in 100 μL of RiAFP-m9/TCEP solution. The mixture was rotated overnight at room temperature. This method was also used to conjugate AuNPs to SBAFP-CT fibrils. Gold-labelled fibrils were imaged by TEM.

### Electroless silver plating

Gold-labelled RiAFP-m9 or SBAFP-CT fibrils were deposited on a carbon coated nickel grid and incubated at room temperature for 2 min. After removing the solution by filter paper, the specimen was washed with water twice for 1 min each. The grid was placed upside down on distilled water before silver enhancement. Electroless silver plating of gold-labelled fibrils was performed using AURION R-Gent SE-EM Kit. Briefly, the grid was placed upside down on drops of silver enhancement reagent for 30–45 min, and then was washed with water 3 times for 5 min each. After drying by filter paper, the specimen was stained with 2% uranyl acetate at room temperature for 30 s. Silver enhancement of gold-labelled fibrils was observed by TEM. Initiator-to-activator ratios of 1:40 and 1:200 were used for silver enhancement of gold-labelled RiAFP-m9 fibrils, but no obvious difference was found.

## Results

### RiAFP-m9 design

**Fig 1:**
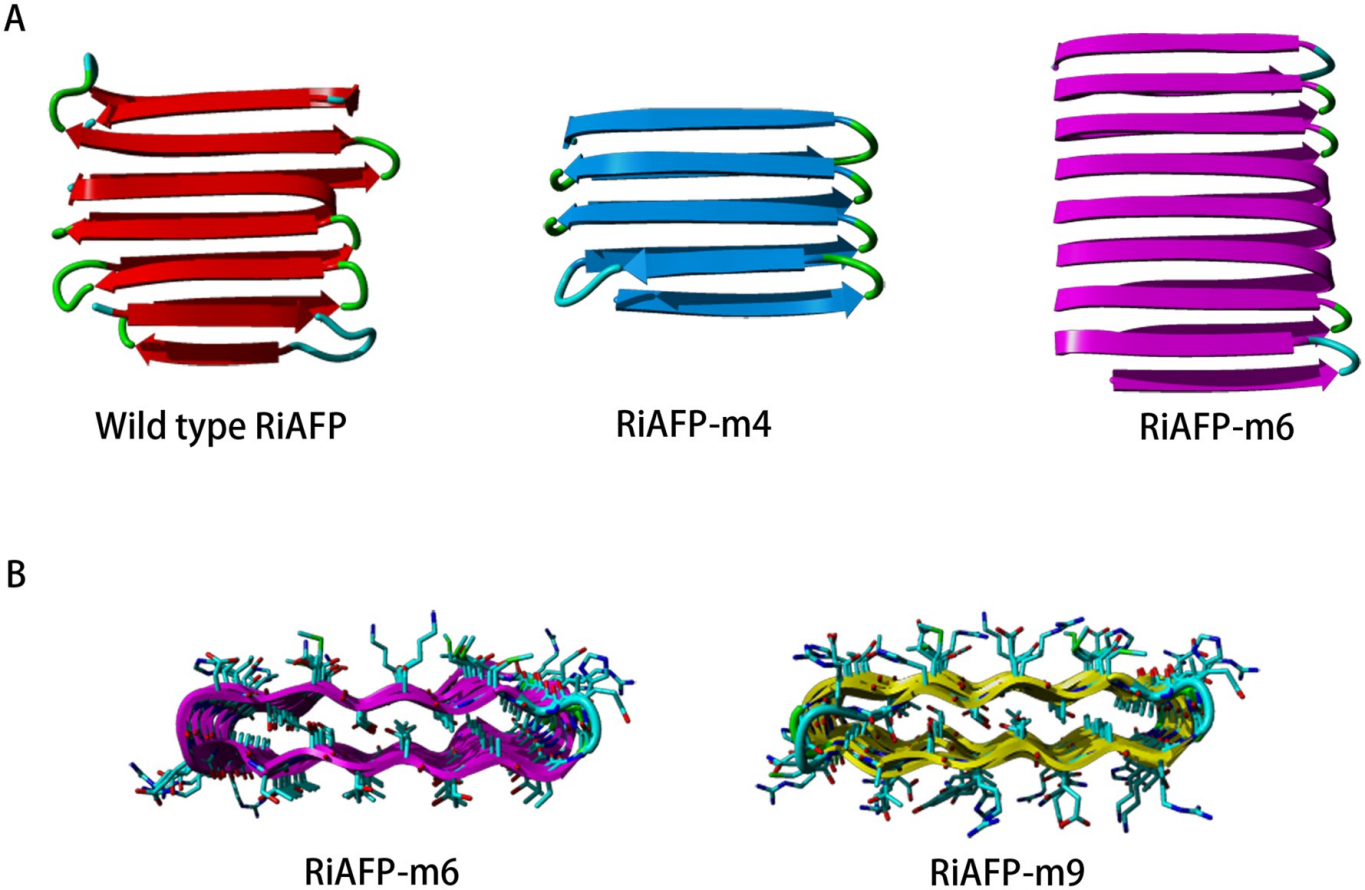
Design of RiAFP-m9. A) The wild type protein has four highly regular solenoid turns in the middle of the structure (residues 20–105) which were extracted from the structure. Residues 90–93 were removed to regularize the edge, leading to RiAFP-m4. RiAFP-m6 was generated by concatenating two RiAFP-m4 structures. B) RiAFP-m9 was developed from RiAFP-m6 by increasing surface hydrophilicity and salt bridging at the interface.

The AFP from the beetle *Rhagium inquisitor* (PDB entry 4DT5)[42] was used as the basis for the design. The design process was similar to that used before,[41] and is outlined in Fig 1. The four structurally regular solenoid turns in the middle of the 4DT5 structure (residues 20–105) were manually extracted from the PDB entry using YASARA. Residues 90–93 were removed to regularize the final β-turn. The 4-turn structure (“RiAFP-m4”, Fig 1A) from this procedure was only 7.4 kDa, so it was concatenated to a copy of itself to form a 14.8 kDa monomer. This larger monomer (RiAFP-m6) was submitted for gene synthesis but failed multiple times using codon optimizations for various organisms to alter the DNA sequence.

The second engineering attempt employed a consensus design approach. A polyalanine version of the modelled RiAFP-m6 structure was submitted to the RosettaDesign server for all-residue modeling.[54] A total of 50 modelled structures were obtained, and the amino acid sequences were extracted and incorporated into a multiple sequence alignment. A single consensus sequence was generated from this multiple sequence alignment using BioEdit, and a structure for this consensus sequence was generated by homology modelling based on the RiAFP-m6 structure using YASARA. MD simulations showed this structure to be stable over several nanoseconds at 100 °C. A synthetic gene was procured and cloned into pET-28a, however protein expression trials showed no protein production.

The third independent round of engineering produced the successful design, RiAFP-m9, which was based on RiAFP-m6. Manual redesign using YASARA was undertaken with the goal of generally increasing surface hydrophilicity as well as salt bridging interactions at the monomer-monomer interface of the modelled dimer. The internal side chains of the structure were not altered from the original PDB entry, only surface residues. Three cysteine residues were introduced on the surface to enhance the binding of gold. An N-terminal 6×histidine affinity tag was added to facilitate purification, followed by a Ser-Gly-Ser-Gly linker. A noncovalent dimer of RiAFP-m9 showed good interface stability in MD simulations at 100 °C for 4 ns. Models for the RiAFP-m6 and RiAFP-m9 proteins are provided in PDB format in the Supporting Information. A comparison of these models clearly shows the amino acid alterations from those of the wild type protein to those of RiAFP-m9, which led to the successful design.

### Protein expression and purification

**Fig 2:**
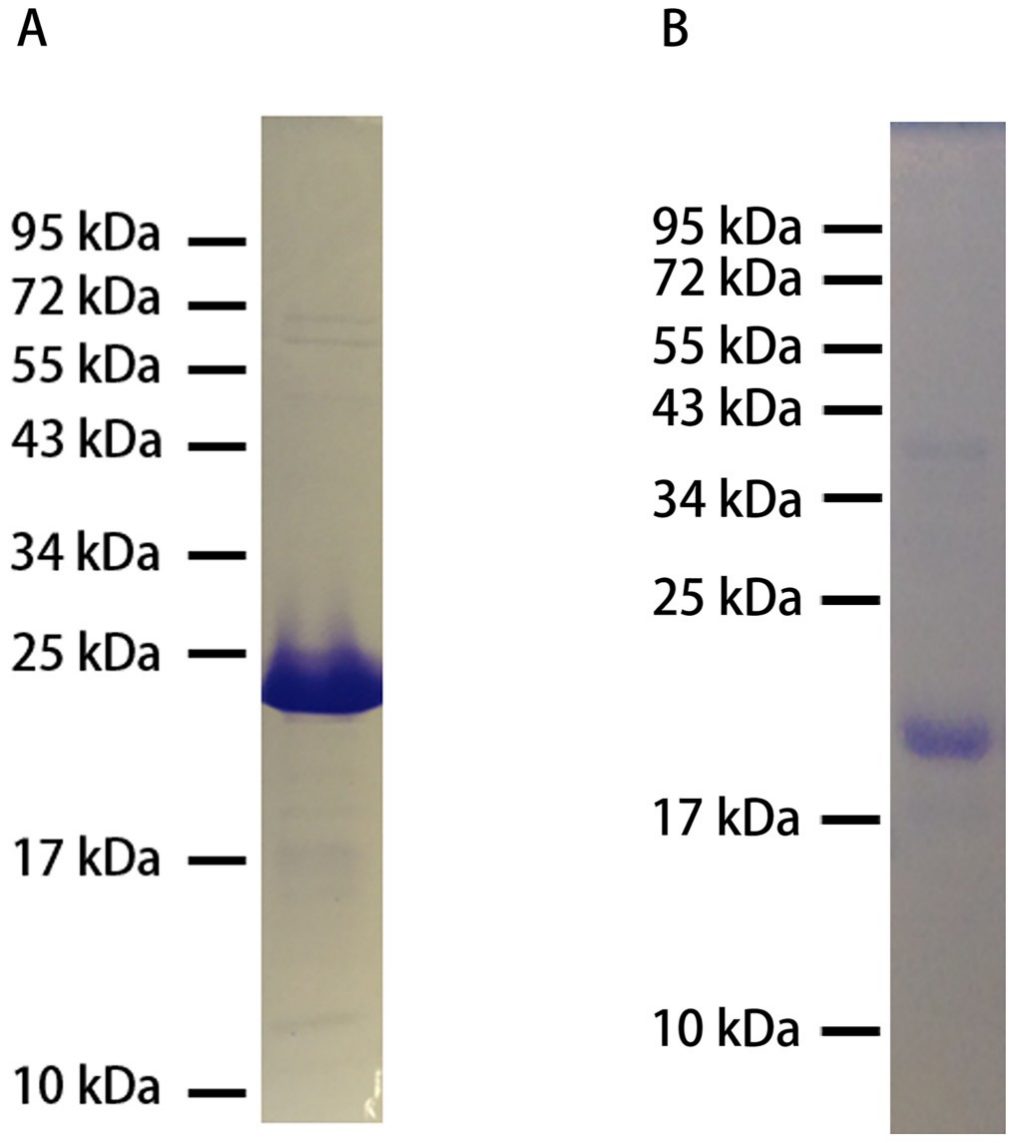
SDS-PAGE of purified recombinant proteins. A) RiAFP-m9 appears at a larger apparent mass (~23 kDa) than the theoretical mass (17 kDa) on SDS-PAGE. The molecular weight of the purified protein is verified by ESI-MS (17 kDa). B) SBAFP-CT runs as expected at ~20 kDa on SDS-PAGE.

The RiAFP-m9 protein expressed well in *E*.*coli*, was found in the soluble fraction, and was purified by nickel affinity chromatography. Approximately 30 mg of purified protein was obtained from 1 L of culture. SDS-PAGE shows that the final preparation of RiAFP-m9 was pure (Fig 2A). RiAFP-m9 ran on SDS-PAGE with an apparent mass (~23 kDa) that is larger than the expected (17 kDa). Similarly, the wild type RiAFP runs at a higher apparent molecular weight (~17 kD) on SDS-PAGE than the true molecular weight (~13 kD).[55] Separation of proteins by SDS-PAGE depends on the homogenous binding of negatively charged SDS to the protein, giving a uniform charge-to-mass ratio, but the amount of bound SDS can differ between proteins. The apparent molecular weight may be affected by charge, hydrophobicity, or simply the amino acid sequence. A number of proteins show anomalous SDS-PAGE migration.[56] The mass of RiAFP-m9 determined by ESI-MS (17 kDa) matches the theoretical mass, confirming the identity of the protein.

SBAFP-CT was found in inclusion bodies. It was purified as reported for SBAFP-m1.[41] Approximately 36 mg of purified protein was obtained from 1 L of culture. As shown in Fig 2B, the final protein preparation was pure and ran at the expected mass on SDS-PAGE (~20 kDa). The mass determined by MALDI (20 kDa) matched the theoretical mass.

### CD spectra

**Fig 3:**
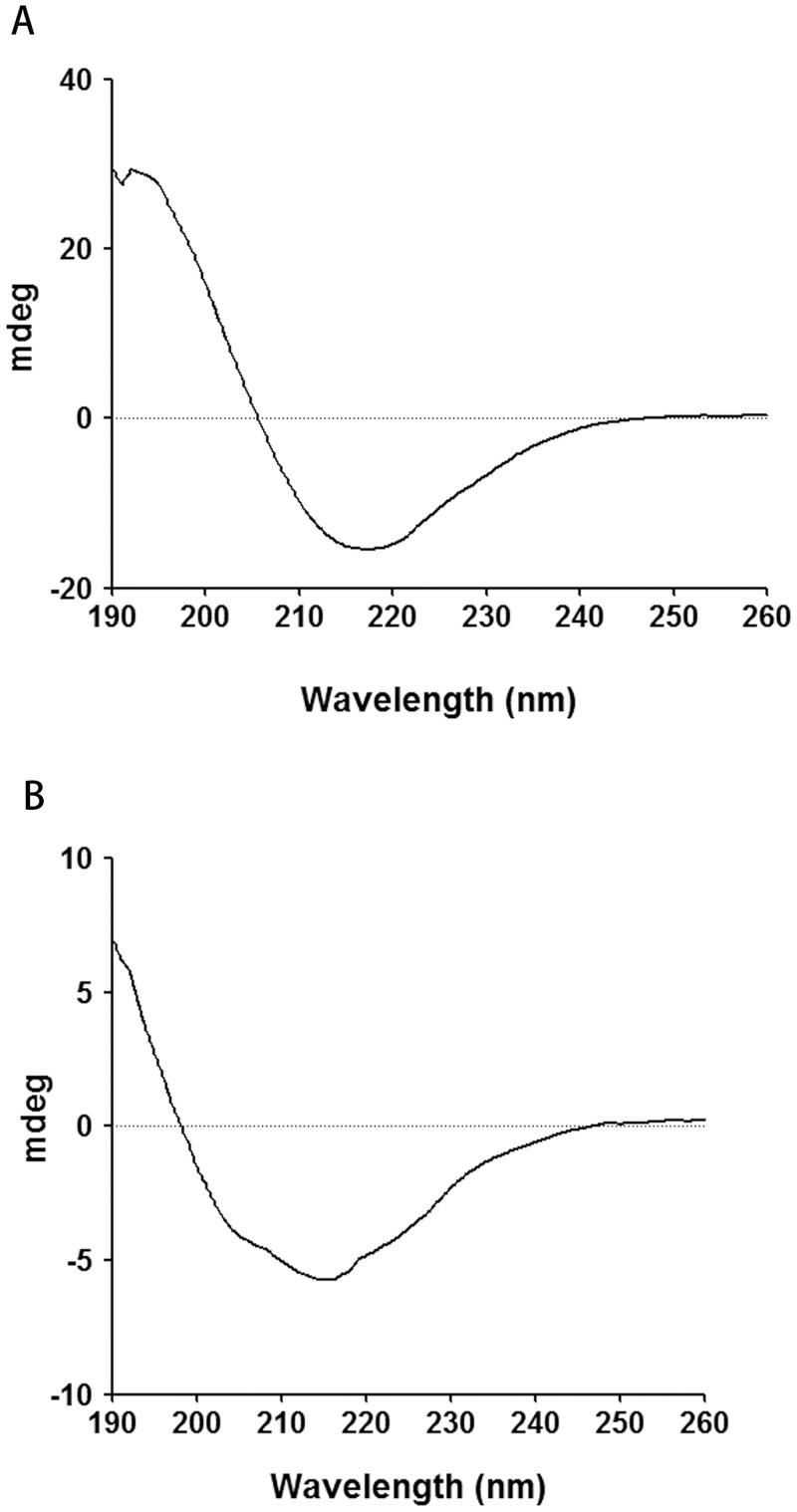
CD spectra of purified recombinant proteins. A) CD spectrum of RiAFP-m9. B) CD spectrum of SBAFP-CT.

The CD spectra for RiAFP-m9 and SBAFP-CT after incubation at 37 °C show a single negative peak at ~218 nm indicative of predominantly β-sheet secondary structure (Fig 3).

The secondary structure content of RiAFP-m9 calculated by YASARA from the final model is 90% β-sheet, 5% β-turn and 5% random coil. That for SBAFP-CT is 85% β-sheet, 4% β-turn and 11% random coil. The secondary structure content determined using CDNN to deconvolute the CD spectra is 53% β-sheet, 15% β-turn, 8% α-helix and 24% random coil for RiAFP-m9, and 48% β-sheet, 17% β-turn, 8% α-helix and 26% random coil for SBAFP-CT.

### ThT fluorescence

**Fig 4:**
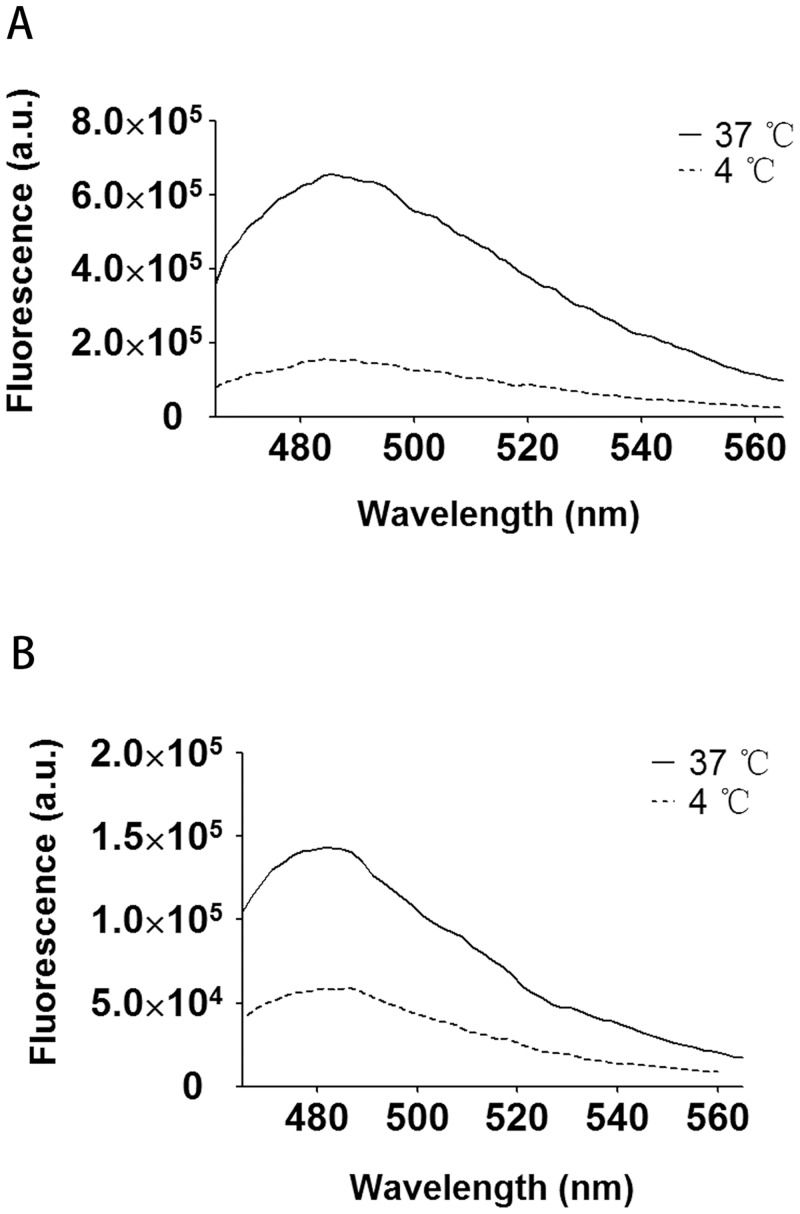
ThT fluorescence analysis of purified recombinant proteins. ThT fluorescence emission at 482 nm before and after incubation at 37 °C. A) RiAFP-m9. B) SBAFP-CT.

ThT fluorescence enhancement due to binding cross-β peptide structure is widely used to identify amyloid fibrils, both *in vivo* and *in vitro*.[57, 58] Fig 4A shows ThT fluorescence spectra of RiAFP-m9 stored at 4 °C and incubated at 37 °C. RiAFP-m9 maintained at 4 °C exhibits low ThT fluorescence, whereas RiAFP-m9 incubated at 37 °C shows enhanced ThT fluorescence at 482 nm. The increase after incubation at 37 °C suggests formation of an extended cross-β structure. Similarly, ThT fluorescence at 482 nm with SBAFP-CT is enhanced after incubation at 37 °C (Fig 4B).

### TEM

**Fig 5:**
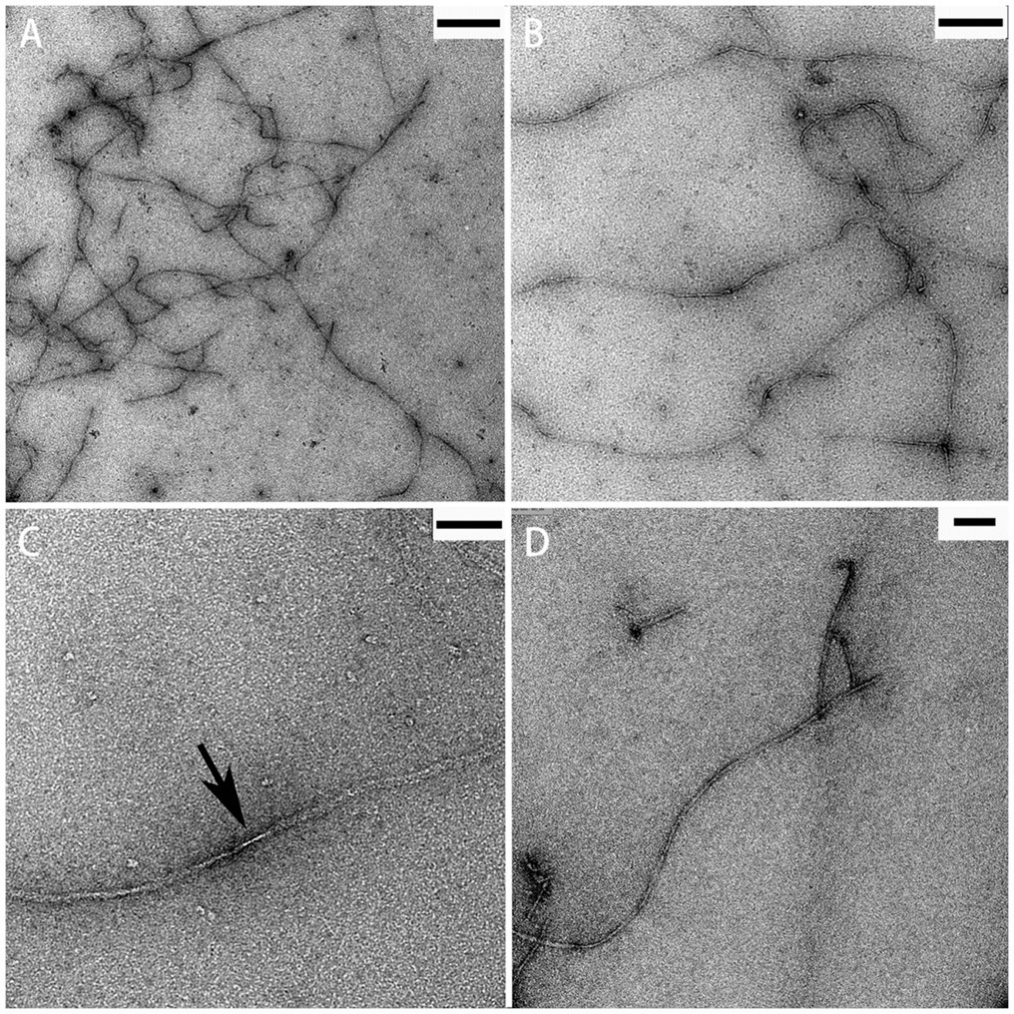
TEM images of self-assembled fibrils. A-C) RiAFP-m9 fibrils formed after incubation at 37 °C. The arrow indicates the twist of a single fibril. D) SBAFP-CT fibrils. Scale bar: A) 500 nm; B) 200 nm (resolution 3.5–3.8 nm); C) 60 nm; D) 100 nm.

TEM images show that purified RiAFP-m9 assembles into micrometer-long fibrils at 37 °C (Fig 5A–5C). Under higher magnification, a twist in the RiAFP-m9 fibril can be seen (Fig 5C). TEM images of SBAFP-CT incubated at 37 °C also show micrometer-long fibrils (Fig 5D). Fibrillar structures with widths of 4.8 nm, 6.8 nm, 10.2 nm, 14.1 nm have been observed in TEM images of RiAFP-m9. The model of the RiAFP-m9 monomer has an average width of 3.7 nm. Within the experimental resolutions noted in Fig 5, these widths correspond to fibrils that are 1, 2, 3 and 4 monomers wide. This can arise via lateral self-association of the fibrils.

### AuNP conjugation

**Fig 6:**
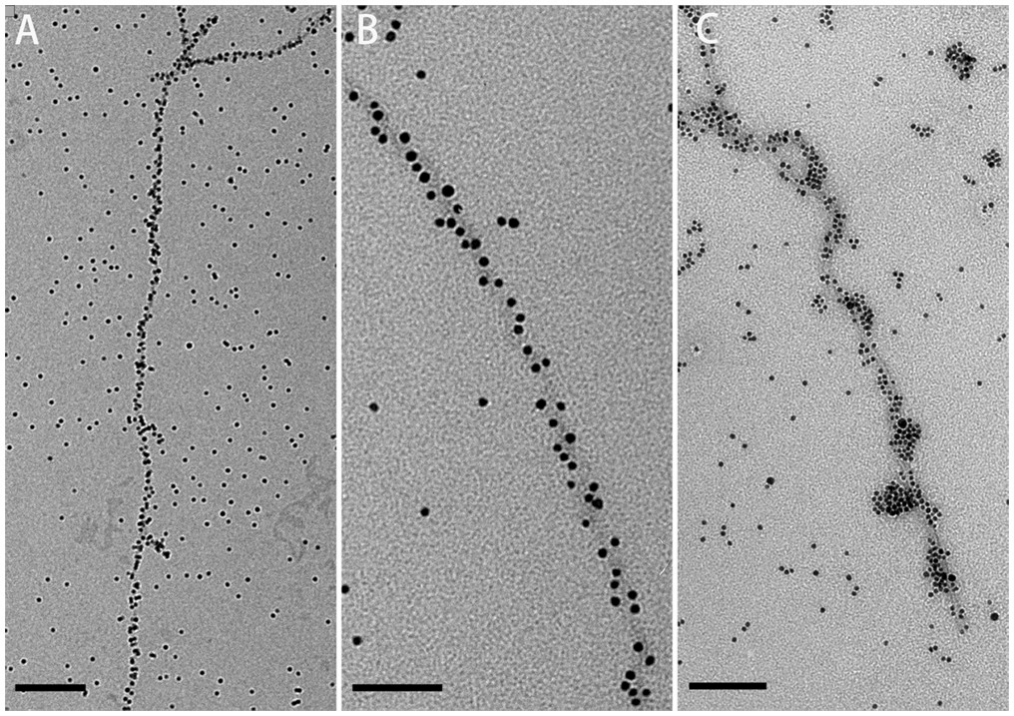
TEM images of representative gold-labelled fibrils. A & B) RiAFP-m9 fibrils labelled with 5 nm AuNPs. C) SBAFP-CT fibrils labelled with 5 nm AuNPs. Scale bar: A) 200 nm; B) 60 nm: C) 100 nm.

RiAFP-m9 has 3 cysteines on its surface, while SBAFP-CT has a 5×cysteine tag on the N-terminus to promote gold binding. Cysteine chemisorbs to gold nanoparticles via its thiol group.[43–46] AuNP conjugation required the presence of TCEP (a reducing agent that is compatible with thiol-dependent reactions)[59]. TCEP-treated fibrils readily reacted with citrate capped 5 nm AuNPs to yield AuNP-conjugated fibrils (Fig 6). The AuNPs were densely distributed along both the RiAFP-m9 and SBAFP-CT fibrils.

### Electroless silver plating

**Fig 7:**
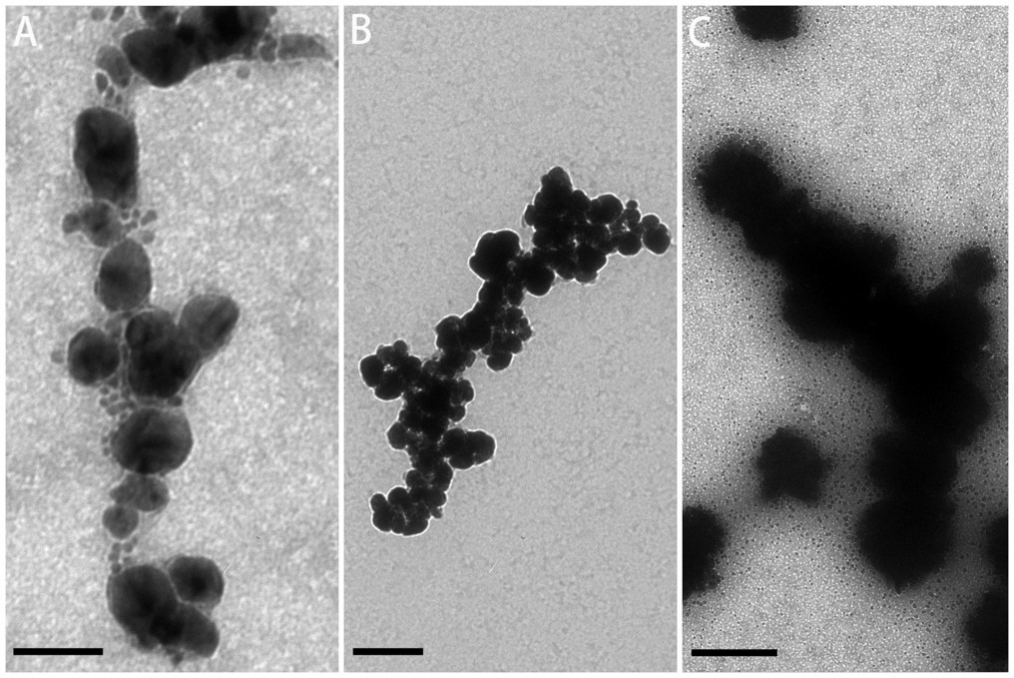
TEM images of silver-enhanced RiAFP-m9 fibrils. A) Short gold-labelled fibrils with a small amount of silver enhancement. B) Longer gold-labelled fibrils with more silver enhancement. C) Long gold-labelled fibrils with strong silver enhancement. Scale bar: A) 60 nm; B) 200 nm; C) 500 nm.

**Fig 8:**
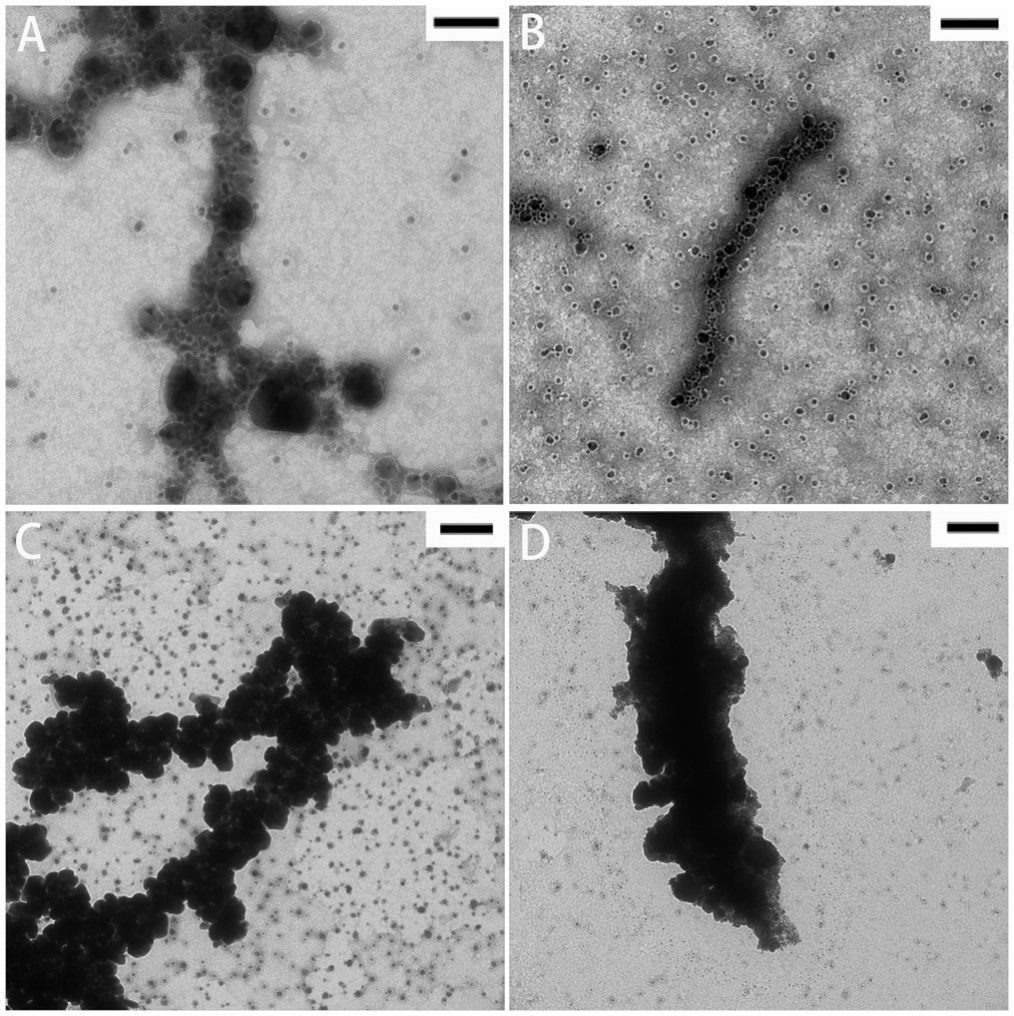
TEM images of silver-enhanced SBAFP-CT fibrils. A & B) Shorter gold-labelled fibrils with a small amount of silver enhancement. C & D) Longer gold-labelled fibrils with strong silver enhancement. Scale bar: A) 60 nm; B) 100 nm; C & D) 200 nm.

Neither RiAFP-m9 nor SBAFP-CT AuNP-conjugated fibrils formed continuous metallic interactions over the length of the fibrils. Therefore, we attempted to bridge the nanoparticles with silver to generate conductive nanowires. A commercial electroless silver plating reagent was used.[22] The results of silver enhancement of AuNP-labeled RiAFP-m9 and SBAFP-CT fibrils are shown in Figs 7 and 8. Silver enhancement gave irregular results. It shortened the AuNP-labeled fibrils of both proteins. Some fibrils were not completely covered with silver (Figs 7A, 8A and 8B), whereas many others were densely covered (Figs 7B, 7C, 8C and 8D). Optimization, including varying the initiator-to-activator ratio, deposition time, etc. did not significantly alter the results.

## Discussion

It was previously demonstrated that two β-solenoid AFPs could be engineered to polymerize into cross-β fibrils under benign conditions.[41] Here, a different β-solenoid AFP from the *Rhagium inquisitor* beetle was engineered (RiAFP-m9) to self-assemble into micrometer-long fibrils using the same basic strategy. RiAFP-m9 is soluble when expressed in *E*. *coli*, unlike the two previously described, engineered β-solenoid AFPs[41], which are expressed in inclusion bodies. RiAFP-m9 has an extraordinarily regular rectangular-like geometry (Fig 1). The polypeptide backbone is 34 Å long, 31 Å wide and 5.8 Å tall. This flat and wide geometry should be beneficial for functionalization and engineering of higher order structures based on RiAFP-m9.

Additionally, a previously engineered spruce budworm AFP[41] was N-terminally modified with a 5×cysteine tag (SBAFP-CT) and shown to polymerize into long fibrils. The fact that both RiAFP-m9 and SBAFP-CT have N-terminal extensions (His and Cys tags, respectively) and retain their ability to self-assemble into fibrils demonstrates that N-terminal peptide extensions do not disrupt the cross-β interface between monomers. The addition of the tags to the proteins was not expected to hinder amyloid fibril formation, as seen in literature examples[24, 30, 32]. Fibril characterization of the tagged proteins was undertaken: CD analysis, ThT fluorescence and TEM imaging. These three independent methods demonstrate the presence of extended amyloid fibrils for the tagged proteins. In addition, that RiAFP-m9 shows a beta-sheet peak in powder diffraction.[50] The tolerance of peptide tags at the termini heralds a variety of peptide and protein fusion constructs, which could be used to functionalize fibrils with catalytic activity, for example, and provide for assembly of higher order structures by fusing to multimerization domains such as the “foldon”[60].

Conjugation of AuNPs to protein monomers prior to polymerization inhibited fibril formation for both proteins. One explanation for these results is that the AuNP surface interacts with the first rung of the BSP (*e*.*g*., citrate-peptide hydrogen bonding) and sterically prevents monomer-monomer association. However, after polymerization, the fibrils readily bound AuNPs.

The gold-labelled fibrils were elaborated into nanowires by electroless silver plating. The length of the wires reaches 2–5 μm, with a width of 0.2–0.5 μm. It may be possible to optimize reaction conditions to produce longer and thinner wires. Fibril fragmentation during silver enhancement suggests that a stronger monomer interface is the key to longer nanowires, which might be achieved by engineering covalent interactions across the interface. Future work aimed at decreasing the diameter and increasing the uniformity of the nanowires will focus on increasing the density of AuNPs on the protein surface, using nanoparticles of different sizes, and using different electroless deposition chemistries. The goal is to produce highly conductive nanowires with lengths of >10 μm and widths of <20 nm.

To date, cross-β fibrils from three BSPs have been successfully produced, each with a different geometric cross-section. Thus, a general strategy for designing cross-β fibrils from BSPs is at hand. Two of the designed BSPs (SBAFP and RiAFP) were shown to have tensile strengths similar to those of spider silk and Kevlar.[51] Compared to DNA scaffolds, BSP scaffolds have more chemical functionality and lend themselves better to industrial production. Compared to viral scaffolds, BSP scaffolds offer greater precision of functional group placement and “engineerability” into higher dimensional structures.

Other protein-based fibrils have been synthesized from proteins subjected to extreme conditions[25–28], amyloidogenic proteins previously known to form fibrils naturally[29, 30] or from two-protein systems where a second protein provides cross-linking[31]. The present approach is unique because it focuses on designing intrinsically stable monomeric BSPs to self-assemble into fibrils under mild conditions. These monomers fibrillize in a cell-free environment, do not require harsh conditions or accessory proteins to assemble, and are extremely stable, highlighting their potential for engineering into 2D and 3D scaffolds that can be used to create functional nanomaterials. The attachment of material-specific binding peptides[61–66] to the N- or C-terminus of BSPs may provide a simple route to template a wide variety of materials.

## Conclusions

**Fig 9:**
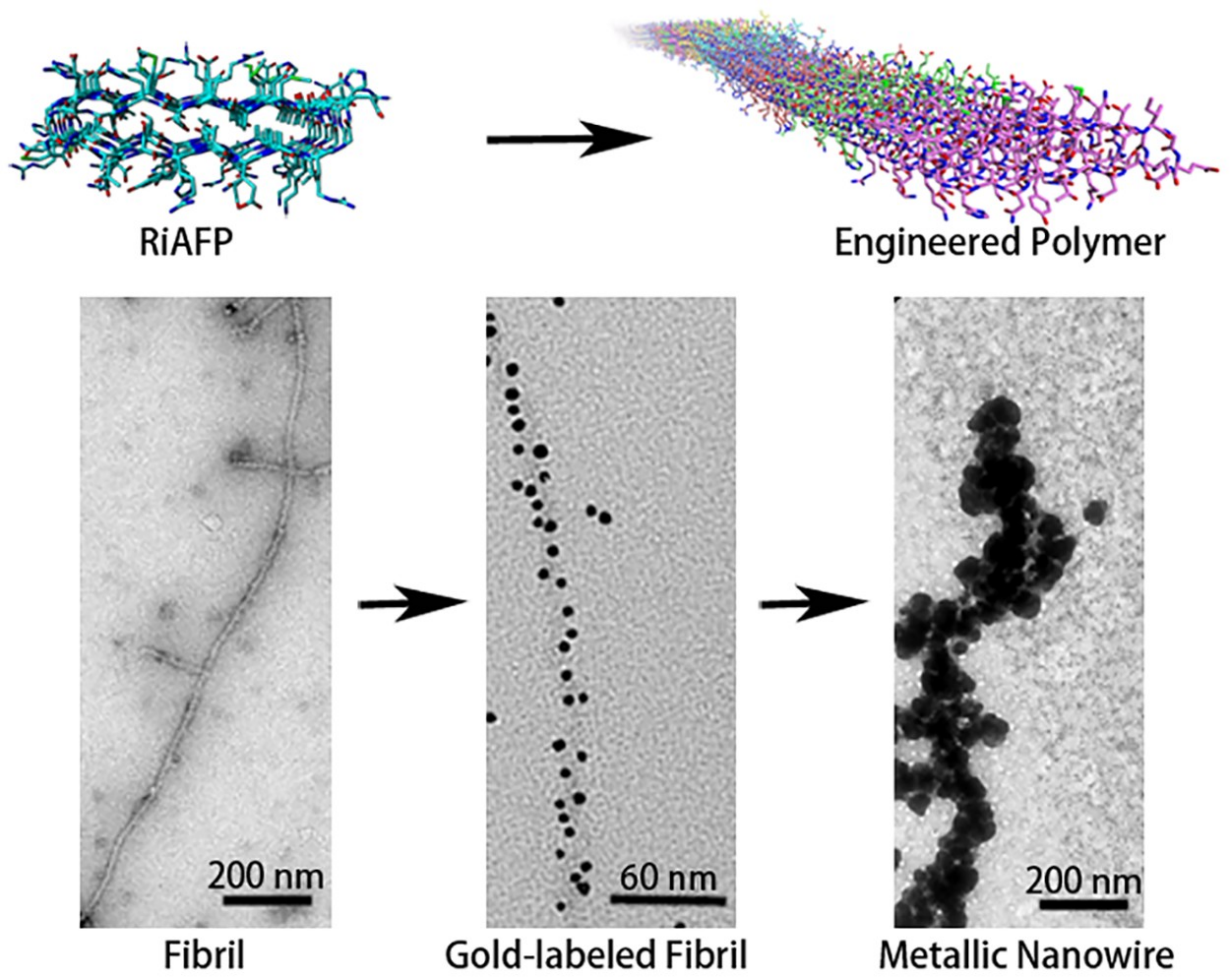
Overview of engineering and functionalizing cross-β fibrils from β-solenoid protein monomers. Fibrils formed from the engineered protein are micrometers long. These fibrils were readily conjugated to 5 nm gold nanoparticles to give densely modified structures. The gold nanoparticle conjugated fibrils were further modified by electroless silver deposition to generate silver nanowires.

In summary, micrometer-length fibrils were created by self-assembly of engineered monomeric BSPs. The design, expression, purification, and self-assembly of a novel BSP protein, RiAFP, was achieved using the general procedures previously applied.[41] RiAFP-m9 has three exposed cysteines while the novel SBAFP-CT described here has a 5×cysteine tag at the N-terminus. Dense chemisorption to 5 nm AuNPs was achieved without additional protein or nanoparticle functionalization. The gold conjugated fibrils were made into nanowires ranging from 2–5 μm in length and 0.2–0.5 μm in width by electroless silver plating (Fig 9).

BSP fibrils are a new class of protein-based scaffolds that hold great promise as a general template for making functional nanomaterials in 1-, 2- and 3-dimensions. The results presented here add an important building block to the nanotechnology armament, and are remarkable for their robustness of assembly, tensile strength, and engineerability.

## Supporting information

S1 File PDB-formatted coordinates for the RiAFP-m6 and RiAFP-m9 models. (DOCX)
