# Supplementary material for: Bottom-up synthesis of protein-based nanomaterials from engineered β-solenoid proteins
Source: PLoS One. 2020 Feb 21;15(2):e0229319. doi: 10.1371/journal.pone.0229319 (PMC7034853; doi:10.1371/journal.pone.0229319)
Supplement: S1 File — (DOCX) [file pone.0229319.s001.docx]

**Supporting Information**

Bottom-up synthesis of protein-based nanomaterials from engineered

β-solenoid proteins

Zeyu Peng, ^1, #a, *^ Maria D.R. Peralta,^1^ Daniel L. Cox^2^ and Michael D. Toney^1^

^1^Department of Chemistry, University of California, Davis, Davis, CA 95616

^2^Department of Physics, University of California, Davis, Davis, CA 95616

^#a^Current address: Department of Microbial Pathogenesis and Immunology, Texas A&M University Health Science Center, College Station, TX 77843

*Corresponding author: zpeng@tamu.edu

Contents: pdb-formatted coordinates for the RiAFP-m6 and RiAFP-m9 models

REMARK RiAFP-m6 model coordinates

SEQRES 1 A 159 SER SER THR ALA THR ALA THR GLY GLU ALA MET ALA SER

SEQRES 2 A 159 GLY THR SER ASP GLY GLU ALA THR SER THR ALA THR ALA

SEQRES 3 A 159 THR GLY HIS ALA THR ALA LYS SER MET SER THR GLY ARG

SEQRES 4 A 159 ALA THR ALA THR THR THR ALA THR GLY THR ALA MET ALA

SEQRES 5 A 159 THR SER ASN ALA ILE GLY GLU ALA THR ALA THR THR THR

SEQRES 6 A 159 ALA THR GLY ARG ALA THR SER SER SER THR THR HIS GLY

SEQRES 7 A 159 ARG ALA THR SER THR ALA THR ALA THR GLY GLU ALA MET

SEQRES 8 A 159 ALA SER GLY THR SER ASP GLY GLU ALA THR SER THR ALA

SEQRES 9 A 159 THR ALA THR GLY HIS ALA THR ALA LYS SER MET SER THR

SEQRES 10 A 159 GLY ARG ALA THR ALA THR THR THR ALA THR GLY THR ALA

SEQRES 11 A 159 MET ALA THR SER ASN ALA ILE GLY GLU ALA THR ALA THR

SEQRES 12 A 159 THR THR ALA THR GLY ARG ALA THR SER SER SER THR THR

SEQRES 13 A 159 HIS GLY ARG

ATOM 1 N SER A 20 31.251 -18.946 -7.492 1.00 8.01 N

ATOM 2 1H SER A 20 31.181 -18.558 -6.573 1.00 8.01 H

ATOM 3 2H SER A 20 32.178 -19.304 -7.606 1.00 8.01 H

ATOM 4 3H SER A 20 30.615 -19.716 -7.549 1.00 8.01 H

ATOM 5 CA SER A 20 30.942 -17.917 -8.534 1.00 7.32 C

ATOM 6 HA SER A 20 31.611 -17.183 -8.417 1.00 7.32 H

ATOM 7 C SER A 20 29.548 -17.325 -8.355 1.00 6.71 C

ATOM 8 O SER A 20 28.542 -18.026 -8.455 1.00 7.40 O

ATOM 9 CB SER A 20 31.058 -18.545 -9.925 1.00 0.00 C

ATOM 10 1HB SER A 20 31.988 -18.874 -10.087 1.00 0.00 H

ATOM 11 2HB SER A 20 30.413 -19.303 -10.024 1.00 0.00 H

ATOM 12 OG SER A 20 30.760 -17.599 -10.937 1.00 0.00 O

ATOM 13 HG SER A 20 29.933 -17.161 -10.732 1.00 0.00 H

ATOM 14 N SER A 21 29.483 -16.025 -8.088 1.00 5.95 N

ATOM 15 H SER A 21 30.323 -15.468 -8.029 1.00 5.95 H

ATOM 16 CA SER A 21 28.204 -15.353 -7.885 1.00 6.66 C

ATOM 17 HA SER A 21 27.412 -15.972 -8.308 1.00 6.66 H

ATOM 18 C SER A 21 28.173 -14.002 -8.592 1.00 5.82 C

ATOM 19 O SER A 21 29.183 -13.302 -8.662 1.00 7.46 O

ATOM 20 CB SER A 21 27.936 -15.177 -6.388 1.00 0.00 C

ATOM 21 1HB SER A 21 26.963 -14.705 -6.250 1.00 0.00 H

ATOM 22 2HB SER A 21 27.926 -16.157 -5.911 1.00 0.00 H

ATOM 23 OG SER A 21 28.937 -14.380 -5.780 1.00 0.00 O

ATOM 24 HG SER A 21 29.777 -14.840 -5.850 1.00 0.00 H

ATOM 25 N THR A 22 27.014 -13.657 -9.143 1.00 5.49 N

ATOM 26 H THR A 22 26.221 -14.280 -9.078 1.00 5.49 H

ATOM 27 CA THR A 22 26.834 -12.397 -9.860 1.00 6.16 C

ATOM 28 HA THR A 22 27.385 -11.616 -9.338 1.00 6.16 H

ATOM 29 C THR A 22 25.353 -12.028 -9.867 1.00 5.28 C

ATOM 30 O THR A 22 24.493 -12.899 -9.730 1.00 8.78 O

ATOM 31 CB THR A 22 27.372 -12.481 -11.306 1.00 0.00 C

ATOM 32 HB THR A 22 28.374 -12.909 -11.283 1.00 0.00 H

ATOM 33 OG1 THR A 22 27.448 -11.163 -11.863 1.00 0.00 O

ATOM 34 HG1 THR A 22 28.224 -10.725 -11.504 1.00 0.00 H

ATOM 35 CG2 THR A 22 26.493 -13.346 -12.203 1.00 0.00 C

ATOM 36 1HG2 THR A 22 26.942 -13.408 -13.194 1.00 0.00 H

ATOM 37 2HG2 THR A 22 25.500 -12.905 -12.289 1.00 0.00 H

ATOM 38 3HG2 THR A 22 26.413 -14.349 -11.785 1.00 0.00 H

ATOM 39 N ALA A 23 25.047 -10.743 -10.011 1.00 4.67 N

ATOM 40 H ALA A 23 25.775 -10.057 -10.152 1.00 4.67 H

ATOM 41 CA ALA A 23 23.666 -10.274 -9.984 1.00 4.90 C

ATOM 42 HA ALA A 23 23.027 -11.029 -10.441 1.00 4.90 H

ATOM 43 C ALA A 23 23.520 -8.977 -10.775 1.00 4.49 C

ATOM 44 O ALA A 23 24.497 -8.258 -10.986 1.00 6.75 O

ATOM 45 CB ALA A 23 23.222 -10.061 -8.543 1.00 5.85 C

ATOM 46 1HB ALA A 23 23.311 -10.996 -7.990 1.00 5.85 H

ATOM 47 2HB ALA A 23 22.183 -9.733 -8.527 1.00 5.85 H

ATOM 48 3HB ALA A 23 23.847 -9.301 -8.075 1.00 5.85 H

ATOM 49 N THR A 24 22.299 -8.673 -11.202 1.00 4.38 N

ATOM 50 H THR A 24 21.527 -9.298 -11.013 1.00 4.38 H

ATOM 51 CA THR A 24 22.016 -7.435 -11.924 1.00 4.55 C

ATOM 52 HA THR A 24 22.682 -6.658 -11.549 1.00 4.55 H

ATOM 53 C THR A 24 20.579 -6.990 -11.667 1.00 4.01 C

ATOM 54 O THR A 24 19.703 -7.816 -11.406 1.00 4.76 O

ATOM 55 CB THR A 24 22.265 -7.584 -13.441 1.00 0.00 C

ATOM 56 HB THR A 24 23.246 -8.033 -13.593 1.00 0.00 H

ATOM 57 OG1 THR A 24 22.249 -6.289 -14.055 1.00 0.00 O

ATOM 58 HG1 THR A 24 22.425 -6.397 -14.992 1.00 0.00 H

ATOM 59 CG2 THR A 24 21.214 -8.460 -14.116 1.00 0.00 C

ATOM 60 1HG2 THR A 24 21.488 -8.611 -15.160 1.00 0.00 H

ATOM 61 2HG2 THR A 24 20.240 -7.973 -14.069 1.00 0.00 H

ATOM 62 3HG2 THR A 24 21.161 -9.428 -13.617 1.00 0.00 H

ATOM 63 N ALA A 25 20.340 -5.685 -11.726 1.00 4.01 N

ATOM 64 H ALA A 25 21.078 -5.041 -11.976 1.00 4.01 H

ATOM 65 CA ALA A 25 19.020 -5.128 -11.452 1.00 4.72 C

ATOM 66 HA ALA A 25 18.270 -5.865 -11.735 1.00 4.72 H

ATOM 67 C ALA A 25 18.780 -3.861 -12.269 1.00 4.13 C

ATOM 68 O ALA A 25 19.723 -3.141 -12.599 1.00 5.18 O

ATOM 69 CB ALA A 25 18.878 -4.832 -9.965 1.00 5.59 C

ATOM 70 1HB ALA A 25 19.039 -5.745 -9.392 1.00 5.59 H

ATOM 71 2HB ALA A 25 17.875 -4.457 -9.764 1.00 5.59 H

ATOM 72 3HB ALA A 25 19.611 -4.082 -9.667 1.00 5.59 H

ATOM 73 N THR A 26 17.522 -3.597 -12.609 1.00 4.82 N

ATOM 74 H THR A 26 16.784 -4.224 -12.321 1.00 4.82 H

ATOM 75 CA THR A 26 17.161 -2.417 -13.392 1.00 5.37 C

ATOM 76 HA THR A 26 18.022 -1.751 -13.438 1.00 5.37 H

ATOM 77 C THR A 26 15.999 -1.658 -12.759 1.00 5.36 C

ATOM 78 O THR A 26 15.096 -2.261 -12.179 1.00 6.16 O

ATOM 79 CB THR A 26 16.744 -2.786 -14.831 1.00 6.60 C

ATOM 80 HB THR A 26 16.462 -1.877 -15.363 1.00 6.60 H

ATOM 81 OG1 THR A 26 15.621 -3.675 -14.794 1.00 7.43 O

ATOM 82 HG1 THR A 26 15.434 -3.961 -15.691 1.00 7.43 H

ATOM 83 CG2 THR A 26 17.881 -3.465 -15.587 1.00 7.34 C

ATOM 84 1HG2 THR A 26 17.586 -3.613 -16.626 1.00 7.34 H

ATOM 85 2HG2 THR A 26 18.101 -4.432 -15.137 1.00 7.34 H

ATOM 86 3HG2 THR A 26 18.772 -2.837 -15.554 1.00 7.34 H

ATOM 87 N GLY A 27 16.019 -0.334 -12.880 1.00 5.68 N

ATOM 88 H GLY A 27 16.767 0.121 -13.382 1.00 5.68 H

ATOM 89 CA GLY A 27 15.008 0.499 -12.251 1.00 5.95 C

ATOM 90 1HA GLY A 27 14.918 1.440 -12.794 1.00 5.95 H

ATOM 91 2HA GLY A 27 14.048 -0.015 -12.276 1.00 5.95 H

ATOM 92 C GLY A 27 15.363 0.807 -10.809 1.00 5.58 C

ATOM 93 O GLY A 27 16.462 1.280 -10.521 1.00 6.00 O

ATOM 94 N GLU A 28 14.437 0.525 -9.901 1.00 6.18 N

ATOM 95 H GLU A 28 13.549 0.135 -10.191 1.00 6.18 H

ATOM 96 CA GLU A 28 14.654 0.705 -8.467 1.00 6.09 C

ATOM 97 HA GLU A 28 15.533 1.328 -8.303 1.00 6.09 H

ATOM 98 C GLU A 28 14.883 -0.655 -7.810 1.00 6.05 C

ATOM 99 O GLU A 28 14.936 -0.782 -6.587 1.00 7.67 O

ATOM 100 CB GLU A 28 13.438 1.372 -7.818 1.00 0.00 C

ATOM 101 1HB GLU A 28 12.600 0.678 -7.865 1.00 0.00 H

ATOM 102 2HB GLU A 28 13.669 1.555 -6.768 1.00 0.00 H

ATOM 103 CG GLU A 28 13.007 2.687 -8.453 1.00 0.00 C

ATOM 104 1HG GLU A 28 12.476 3.279 -7.708 1.00 0.00 H

ATOM 105 2HG GLU A 28 13.893 3.241 -8.765 1.00 0.00 H

ATOM 106 CD GLU A 28 12.092 2.477 -9.647 1.00 0.00 C

ATOM 107 OE1 GLU A 28 10.997 1.898 -9.474 1.00 0.00 O

ATOM 108 OE2 GLU A 28 12.450 2.897 -10.768 1.00 0.00 O

ATOM 109 N ALA A 30 14.988 -1.680 -8.649 1.00 5.99 N

ATOM 110 H ALA A 30 14.962 -1.495 -9.642 1.00 5.99 H

ATOM 111 CA ALA A 30 15.128 -3.064 -8.212 1.00 5.30 C

ATOM 112 HA ALA A 30 14.361 -3.267 -7.464 1.00 5.30 H

ATOM 113 C ALA A 30 16.487 -3.353 -7.580 1.00 5.13 C

ATOM 114 O ALA A 30 17.474 -2.667 -7.844 1.00 6.92 O

ATOM 115 CB ALA A 30 14.896 -3.984 -9.399 1.00 5.69 C

ATOM 116 1HB ALA A 30 15.676 -3.826 -10.145 1.00 5.69 H

ATOM 117 2HB ALA A 30 13.923 -3.773 -9.840 1.00 5.69 H

ATOM 118 3HB ALA A 30 14.920 -5.021 -9.066 1.00 5.69 H

ATOM 119 N MET A 31 16.529 -4.390 -6.749 1.00 4.72 N

ATOM 120 H MET A 31 15.693 -4.941 -6.605 1.00 4.72 H

ATOM 121 CA MET A 31 17.741 -4.783 -6.035 1.00 5.29 C

ATOM 122 HA MET A 31 18.590 -4.222 -6.426 1.00 5.29 H

ATOM 123 C MET A 31 18.006 -6.274 -6.216 1.00 4.60 C

ATOM 124 O MET A 31 17.095 -7.091 -6.081 1.00 4.98 O

ATOM 125 CB MET A 31 17.582 -4.485 -4.542 1.00 6.39 C

ATOM 126 1HB MET A 31 16.689 -4.999 -4.184 1.00 6.39 H

ATOM 127 2HB MET A 31 18.444 -4.886 -4.009 1.00 6.39 H

ATOM 128 CG MET A 31 17.452 -3.006 -4.207 1.00 8.61 C

ATOM 129 1HG MET A 31 16.665 -2.567 -4.821 1.00 8.61 H

ATOM 130 2HG MET A 31 17.173 -2.903 -3.158 1.00 8.61 H

ATOM 131 SD MET A 31 18.996 -2.115 -4.487 1.00 11.04 S

ATOM 132 CE MET A 31 18.397 -0.429 -4.629 1.00 8.94 C

ATOM 133 1HE MET A 31 17.704 -0.357 -5.467 1.00 8.94 H

ATOM 134 2HE MET A 31 19.239 0.242 -4.798 1.00 8.94 H

ATOM 135 3HE MET A 31 17.886 -0.144 -3.709 1.00 8.94 H

ATOM 136 N ALA A 32 19.250 -6.640 -6.505 1.00 3.90 N

ATOM 137 H ALA A 32 19.977 -5.946 -6.605 1.00 3.90 H

ATOM 138 CA ALA A 32 19.613 -8.044 -6.677 1.00 3.55 C

ATOM 139 HA ALA A 32 18.906 -8.651 -6.113 1.00 3.55 H

ATOM 140 C ALA A 32 21.016 -8.322 -6.145 1.00 3.90 C

ATOM 141 O ALA A 32 21.918 -7.501 -6.310 1.00 5.01 O

ATOM 142 CB ALA A 32 19.519 -8.435 -8.146 1.00 4.55 C

ATOM 143 1HB ALA A 32 18.515 -8.229 -8.516 1.00 4.55 H

ATOM 144 2HB ALA A 32 19.730 -9.498 -8.253 1.00 4.55 H

ATOM 145 3HB ALA A 32 20.244 -7.862 -8.724 1.00 4.55 H

ATOM 146 N SER A 33 21.203 -9.469 -5.499 1.00 4.02 N

ATOM 147 H SER A 33 20.427 -10.100 -5.354 1.00 4.02 H

ATOM 148 CA SER A 33 22.511 -9.850 -4.973 1.00 3.79 C

ATOM 149 HA SER A 33 23.277 -9.443 -5.633 1.00 3.79 H

ATOM 150 C SER A 33 22.706 -11.363 -4.898 1.00 4.33 C

ATOM 151 O SER A 33 21.745 -12.121 -4.759 1.00 4.84 O

ATOM 152 CB SER A 33 22.706 -9.249 -3.579 1.00 4.55 C

ATOM 153 1HB SER A 33 23.719 -9.460 -3.236 1.00 4.55 H

ATOM 154 2HB SER A 33 22.567 -8.169 -3.634 1.00 4.55 H

ATOM 155 OG SER A 33 21.777 -9.793 -2.656 1.00 5.25 O

ATOM 156 HG SER A 33 21.941 -9.403 -1.794 1.00 5.25 H

ATOM 157 N GLY A 34 23.960 -11.794 -4.978 1.00 4.96 N

ATOM 158 H GLY A 34 24.719 -11.140 -5.106 1.00 4.96 H

ATOM 159 CA GLY A 34 24.295 -13.202 -4.857 1.00 4.50 C

ATOM 160 1HA GLY A 34 23.522 -13.700 -4.274 1.00 4.50 H

ATOM 161 2HA GLY A 34 24.334 -13.655 -5.848 1.00 4.50 H

ATOM 162 C GLY A 34 25.633 -13.405 -4.172 1.00 4.64 C

ATOM 163 O GLY A 34 26.530 -12.575 -4.312 1.00 4.96 O

ATOM 164 N THR A 35 25.770 -14.495 -3.424 1.00 4.35 N

ATOM 165 H THR A 35 24.993 -15.134 -3.320 1.00 4.35 H

ATOM 166 CA THR A 35 27.014 -14.824 -2.730 1.00 4.02 C

ATOM 167 HA THR A 35 27.851 -14.486 -3.339 1.00 4.02 H

ATOM 168 C THR A 35 27.131 -16.336 -2.551 1.00 4.55 C

ATOM 169 O THR A 35 26.126 -17.013 -2.336 1.00 4.35 O

ATOM 170 CB THR A 35 27.097 -14.112 -1.361 1.00 5.12 C

ATOM 171 HB THR A 35 26.947 -13.044 -1.514 1.00 5.12 H

ATOM 172 OG1 THR A 35 28.396 -14.320 -0.793 1.00 5.51 O

ATOM 173 HG1 THR A 35 28.410 -13.925 0.081 1.00 5.51 H

ATOM 174 CG2 THR A 35 26.043 -14.614 -0.379 1.00 5.97 C

ATOM 175 1HG2 THR A 35 26.228 -15.661 -0.138 1.00 5.97 H

ATOM 176 2HG2 THR A 35 25.049 -14.511 -0.814 1.00 5.97 H

ATOM 177 3HG2 THR A 35 26.094 -14.024 0.536 1.00 5.97 H

ATOM 178 N SER A 36 28.333 -16.888 -2.678 1.00 4.63 N

ATOM 179 H SER A 36 29.150 -16.318 -2.842 1.00 4.63 H

ATOM 180 CA SER A 36 28.512 -18.337 -2.617 1.00 4.58 C

ATOM 181 HA SER A 36 27.860 -18.733 -1.838 1.00 4.58 H

ATOM 182 C SER A 36 29.940 -18.773 -2.296 1.00 5.05 C

ATOM 183 O SER A 36 30.885 -17.996 -2.431 1.00 6.22 O

ATOM 184 CB SER A 36 28.094 -18.952 -3.954 1.00 5.69 C

ATOM 185 1HB SER A 36 28.180 -20.036 -3.893 1.00 5.69 H

ATOM 186 2HB SER A 36 27.054 -18.697 -4.155 1.00 5.69 H

ATOM 187 OG SER A 36 28.901 -18.472 -5.015 1.00 7.12 O

ATOM 188 HG SER A 36 29.105 -19.212 -5.594 1.00 7.12 H

ATOM 189 N ASP A 37 30.083 -20.020 -1.858 1.00 6.11 N

ATOM 190 H ASP A 37 29.264 -20.602 -1.742 1.00 6.11 H

ATOM 191 CA ASP A 37 31.375 -20.599 -1.495 1.00 7.09 C

ATOM 192 HA ASP A 37 32.112 -20.296 -2.238 1.00 7.09 H

ATOM 193 C ASP A 37 31.279 -22.126 -1.445 1.00 7.13 C

ATOM 194 O ASP A 37 30.208 -22.632 -1.122 1.00 6.93 O

ATOM 195 CB ASP A 37 31.803 -20.078 -0.119 1.00 0.00 C

ATOM 196 1HB ASP A 37 32.741 -20.558 0.161 1.00 0.00 H

ATOM 197 2HB ASP A 37 31.973 -19.003 -0.183 1.00 0.00 H

ATOM 198 CG ASP A 37 30.768 -20.339 0.963 1.00 0.00 C

ATOM 199 OD1 ASP A 37 29.692 -19.703 0.957 1.00 0.00 O

ATOM 200 OD2 ASP A 37 31.032 -21.167 1.860 1.00 0.00 O

ATOM 201 N GLY A 38 32.316 -22.916 -1.711 1.00 8.52 N

ATOM 202 H GLY A 38 32.225 -23.898 -1.497 1.00 8.52 H

ATOM 203 CA GLY A 38 33.569 -22.489 -2.313 1.00 9.47 C

ATOM 204 1HA GLY A 38 33.796 -21.464 -2.023 1.00 9.47 H

ATOM 205 2HA GLY A 38 34.371 -23.122 -1.933 1.00 9.47 H

ATOM 206 C GLY A 38 33.589 -22.582 -3.827 1.00 8.75 C

ATOM 207 O GLY A 38 34.306 -21.829 -4.484 1.00 11.96 O

ATOM 208 N GLU A 39 32.811 -23.503 -4.386 1.00 9.12 N

ATOM 209 H GLU A 39 32.269 -24.122 -3.799 1.00 9.12 H

ATOM 210 CA GLU A 39 32.661 -23.639 -5.835 1.00 9.05 C

ATOM 211 HA GLU A 39 33.290 -22.907 -6.344 1.00 9.05 H

ATOM 212 C GLU A 39 31.209 -23.388 -6.236 1.00 6.82 C

ATOM 213 O GLU A 39 30.845 -23.464 -7.408 1.00 8.59 O

ATOM 214 CB GLU A 39 33.064 -25.045 -6.288 1.00 0.00 C

ATOM 215 1HB GLU A 39 33.236 -25.666 -5.408 1.00 0.00 H

ATOM 216 2HB GLU A 39 32.237 -25.480 -6.849 1.00 0.00 H

ATOM 217 CG GLU A 39 34.307 -25.083 -7.163 1.00 0.00 C

ATOM 218 1HG GLU A 39 34.179 -24.390 -7.995 1.00 0.00 H

ATOM 219 2HG GLU A 39 35.165 -24.762 -6.571 1.00 0.00 H

ATOM 220 CD GLU A 39 34.572 -26.472 -7.713 1.00 0.00 C

ATOM 221 OE1 GLU A 39 33.733 -26.992 -8.480 1.00 0.00 O

ATOM 222 OE2 GLU A 39 35.619 -27.063 -7.375 1.00 0.00 O

ATOM 223 N ALA A 40 30.380 -23.112 -5.235 1.00 5.73 N

ATOM 224 H ALA A 40 30.760 -23.011 -4.304 1.00 5.73 H

ATOM 225 CA ALA A 40 28.939 -22.969 -5.405 1.00 6.38 C

ATOM 226 HA ALA A 40 28.565 -23.853 -5.920 1.00 6.38 H

ATOM 227 C ALA A 40 28.555 -21.754 -6.246 1.00 6.39 C

ATOM 228 O ALA A 40 29.283 -20.764 -6.318 1.00 7.96 O

ATOM 229 CB ALA A 40 28.284 -22.904 -4.033 1.00 0.00 C

ATOM 230 1HB ALA A 40 28.685 -22.060 -3.472 1.00 0.00 H

ATOM 231 2HB ALA A 40 28.488 -23.825 -3.489 1.00 0.00 H

ATOM 232 3HB ALA A 40 27.207 -22.789 -4.146 1.00 0.00 H

ATOM 233 N THR A 41 27.388 -21.839 -6.876 1.00 5.91 N

ATOM 234 H THR A 41 26.797 -22.645 -6.716 1.00 5.91 H

ATOM 235 CA THR A 41 26.915 -20.820 -7.808 1.00 6.38 C

ATOM 236 HA THR A 41 27.694 -20.072 -7.945 1.00 6.38 H

ATOM 237 C THR A 41 25.657 -20.148 -7.268 1.00 5.07 C

ATOM 238 O THR A 41 24.707 -20.827 -6.880 1.00 6.00 O

ATOM 239 CB THR A 41 26.582 -21.448 -9.178 1.00 9.04 C

ATOM 240 HB THR A 41 25.753 -22.146 -9.058 1.00 9.04 H

ATOM 241 OG1 THR A 41 27.724 -22.161 -9.666 1.00 12.54 O

ATOM 242 HG1 THR A 41 27.462 -22.657 -10.445 1.00 12.54 H

ATOM 243 CG2 THR A 41 26.197 -20.389 -10.205 1.00 11.48 C

ATOM 244 1HG2 THR A 41 25.265 -19.908 -9.908 1.00 11.48 H

ATOM 245 2HG2 THR A 41 26.060 -20.863 -11.177 1.00 11.48 H

ATOM 246 3HG2 THR A 41 26.987 -19.641 -10.277 1.00 11.48 H

ATOM 247 N SER A 42 25.627 -18.820 -7.246 1.00 4.36 N

ATOM 248 H SER A 42 26.436 -18.288 -7.535 1.00 4.36 H

ATOM 249 CA SER A 42 24.421 -18.101 -6.846 1.00 4.85 C

ATOM 250 HA SER A 42 23.566 -18.756 -7.004 1.00 4.85 H

ATOM 251 C SER A 42 24.211 -16.842 -7.682 1.00 4.00 C

ATOM 252 O SER A 42 25.105 -16.004 -7.799 1.00 6.02 O

ATOM 253 CB SER A 42 24.485 -17.742 -5.360 1.00 4.79 C

ATOM 254 1HB SER A 42 23.567 -17.232 -5.071 1.00 4.79 H

ATOM 255 2HB SER A 42 24.586 -18.655 -4.773 1.00 4.79 H

ATOM 256 OG SER A 42 25.593 -16.898 -5.105 1.00 5.43 O

ATOM 257 HG SER A 42 26.396 -17.416 -5.200 1.00 5.43 H

ATOM 258 N THR A 43 23.035 -16.721 -8.288 1.00 4.22 N

ATOM 259 H THR A 43 22.330 -17.437 -8.173 1.00 4.22 H

ATOM 260 CA THR A 43 22.733 -15.587 -9.159 1.00 4.40 C

ATOM 261 HA THR A 43 23.477 -14.809 -8.990 1.00 4.40 H

ATOM 262 C THR A 43 21.355 -15.000 -8.877 1.00 4.25 C

ATOM 263 O THR A 43 20.428 -15.722 -8.509 1.00 4.58 O

ATOM 264 CB THR A 43 22.766 -15.980 -10.651 1.00 6.18 C

ATOM 265 HB THR A 43 22.533 -15.103 -11.254 1.00 6.18 H

ATOM 266 OG1 THR A 43 21.785 -16.995 -10.897 1.00 7.73 O

ATOM 267 HG1 THR A 43 20.919 -16.635 -10.692 1.00 7.73 H

ATOM 268 CG2 THR A 43 24.136 -16.510 -11.059 1.00 7.05 C

ATOM 269 1HG2 THR A 43 24.909 -15.804 -10.756 1.00 7.05 H

ATOM 270 2HG2 THR A 43 24.167 -16.633 -12.142 1.00 7.05 H

ATOM 271 3HG2 THR A 43 24.318 -17.474 -10.584 1.00 7.05 H

ATOM 272 N ALA A 44 21.222 -13.692 -9.063 1.00 4.22 N

ATOM 273 H ALA A 44 22.006 -13.143 -9.386 1.00 4.22 H

ATOM 274 CA ALA A 44 19.963 -12.997 -8.810 1.00 3.91 C

ATOM 275 HA ALA A 44 19.148 -13.719 -8.867 1.00 3.91 H

ATOM 276 C ALA A 44 19.702 -11.907 -9.846 1.00 4.09 C

ATOM 277 O ALA A 44 20.613 -11.183 -10.248 1.00 5.06 O

ATOM 278 CB ALA A 44 19.980 -12.396 -7.412 1.00 4.40 C

ATOM 279 1HB ALA A 44 20.095 -13.189 -6.673 1.00 4.40 H

ATOM 280 2HB ALA A 44 19.044 -11.868 -7.231 1.00 4.40 H

ATOM 281 3HB ALA A 44 20.811 -11.696 -7.325 1.00 4.40 H

ATOM 282 N THR A 45 18.452 -11.806 -10.284 1.00 3.81 N

ATOM 283 H THR A 45 17.756 -12.463 -9.958 1.00 3.81 H

ATOM 284 CA THR A 45 18.031 -10.792 -11.247 1.00 4.32 C

ATOM 285 HA THR A 45 18.799 -10.023 -11.328 1.00 4.32 H

ATOM 286 C THR A 45 16.726 -10.160 -10.773 1.00 3.59 C

ATOM 287 O THR A 45 15.800 -10.874 -10.391 1.00 5.04 O

ATOM 288 CB THR A 45 17.801 -11.417 -12.639 1.00 5.80 C

ATOM 289 HB THR A 45 16.970 -12.120 -12.583 1.00 5.80 H

ATOM 290 OG1 THR A 45 18.981 -12.121 -13.044 1.00 6.79 O

ATOM 291 HG1 THR A 45 18.793 -12.579 -13.867 1.00 6.79 H

ATOM 292 CG2 THR A 45 17.489 -10.355 -13.687 1.00 7.13 C

ATOM 293 1HG2 THR A 45 16.528 -9.891 -13.467 1.00 7.13 H

ATOM 294 2HG2 THR A 45 17.443 -10.822 -14.671 1.00 7.13 H

ATOM 295 3HG2 THR A 45 18.270 -9.594 -13.685 1.00 7.13 H

ATOM 296 N ALA A 46 16.640 -8.834 -10.797 1.00 3.81 N

ATOM 297 H ALA A 46 17.423 -8.277 -11.109 1.00 3.81 H

ATOM 298 CA ALA A 46 15.420 -8.142 -10.390 1.00 4.29 C

ATOM 299 HA ALA A 46 14.586 -8.836 -10.476 1.00 4.29 H

ATOM 300 C ALA A 46 15.131 -6.943 -11.290 1.00 3.95 C

ATOM 301 O ALA A 46 16.051 -6.257 -11.734 1.00 5.13 O

ATOM 302 CB ALA A 46 15.533 -7.706 -8.936 1.00 4.76 C

ATOM 303 1HB ALA A 46 14.591 -7.262 -8.617 1.00 4.76 H

ATOM 304 2HB ALA A 46 16.334 -6.973 -8.833 1.00 4.76 H

ATOM 305 3HB ALA A 46 15.751 -8.571 -8.310 1.00 4.76 H

ATOM 306 N THR A 47 13.859 -6.695 -11.587 1.00 4.04 N

ATOM 307 H THR A 47 13.129 -7.284 -11.212 1.00 4.04 H

ATOM 308 CA THR A 47 13.482 -5.609 -12.490 1.00 4.82 C

ATOM 309 HA THR A 47 14.347 -4.964 -12.646 1.00 4.82 H

ATOM 310 C THR A 47 12.340 -4.752 -11.952 1.00 5.03 C

ATOM 311 O THR A 47 11.418 -5.258 -11.311 1.00 5.88 O

ATOM 312 CB THR A 47 13.017 -6.142 -13.860 1.00 5.41 C

ATOM 313 HB THR A 47 12.747 -5.298 -14.495 1.00 5.41 H

ATOM 314 OG1 THR A 47 11.867 -6.976 -13.678 1.00 6.69 O

ATOM 315 HG1 THR A 47 11.209 -6.476 -13.188 1.00 6.69 H

ATOM 316 CG2 THR A 47 14.110 -6.948 -14.553 1.00 6.48 C

ATOM 317 1HG2 THR A 47 14.304 -7.866 -13.998 1.00 6.48 H

ATOM 318 2HG2 THR A 47 15.025 -6.357 -14.610 1.00 6.48 H

ATOM 319 3HG2 THR A 47 13.785 -7.203 -15.562 1.00 6.48 H

ATOM 320 N GLY A 48 12.399 -3.456 -12.238 1.00 5.74 N

ATOM 321 H GLY A 48 13.210 -3.081 -12.711 1.00 5.74 H

ATOM 322 CA GLY A 48 11.350 -2.530 -11.847 1.00 6.29 C

ATOM 323 1HA GLY A 48 11.312 -1.708 -12.561 1.00 6.29 H

ATOM 324 2HA GLY A 48 10.388 -3.043 -11.856 1.00 6.29 H

ATOM 325 C GLY A 48 11.581 -1.951 -10.466 1.00 6.04 C

ATOM 326 O GLY A 48 12.130 -0.860 -10.328 1.00 7.17 O

ATOM 327 N HIS A 49 11.155 -2.691 -9.448 1.00 7.24 N

ATOM 328 H HIS A 49 10.666 -3.556 -9.639 1.00 7.24 H

ATOM 329 CA HIS A 49 11.324 -2.320 -8.046 1.00 7.44 C

ATOM 330 HA HIS A 49 12.221 -1.711 -7.932 1.00 7.44 H

ATOM 331 C HIS A 49 11.460 -3.587 -7.200 1.00 7.78 C

ATOM 332 O HIS A 49 11.367 -3.559 -5.972 1.00 9.08 O

ATOM 333 CB HIS A 49 10.101 -1.514 -7.597 1.00 0.00 C

ATOM 334 1HB HIS A 49 9.708 -0.963 -8.451 1.00 0.00 H

ATOM 335 2HB HIS A 49 9.332 -2.210 -7.262 1.00 0.00 H

ATOM 336 CG HIS A 49 10.384 -0.538 -6.498 1.00 0.00 C

ATOM 337 ND1 HIS A 49 10.924 -0.875 -5.277 1.00 0.00 N

ATOM 338 HD1 HIS A 49 11.203 -1.801 -4.987 1.00 0.00 H

ATOM 339 CD2 HIS A 49 10.183 0.804 -6.434 1.00 0.00 C

ATOM 340 HD2 HIS A 49 9.767 1.402 -7.231 1.00 0.00 H

ATOM 341 CE1 HIS A 49 11.031 0.247 -4.551 1.00 0.00 C

ATOM 342 HE1 HIS A 49 11.434 0.288 -3.549 1.00 0.00 H

ATOM 343 NE2 HIS A 49 10.571 1.292 -5.210 1.00 0.00 N

ATOM 344 N ALA A 51 11.650 -4.719 -7.869 1.00 5.09 N

ATOM 345 H ALA A 51 11.767 -4.683 -8.872 1.00 5.09 H

ATOM 346 CA ALA A 51 11.659 -6.032 -7.232 1.00 4.61 C

ATOM 347 HA ALA A 51 10.843 -6.077 -6.511 1.00 4.61 H

ATOM 348 C ALA A 51 12.960 -6.318 -6.485 1.00 4.04 C

ATOM 349 O ALA A 51 13.970 -5.648 -6.699 1.00 5.46 O

ATOM 350 CB ALA A 51 11.415 -7.094 -8.293 1.00 5.38 C

ATOM 351 1HB ALA A 51 11.228 -8.052 -7.809 1.00 5.38 H

ATOM 352 2HB ALA A 51 12.287 -7.175 -8.942 1.00 5.38 H

ATOM 353 3HB ALA A 51 10.544 -6.822 -8.889 1.00 5.38 H

ATOM 354 N THR A 52 12.942 -7.323 -5.616 1.00 4.43 N

ATOM 355 H THR A 52 12.089 -7.848 -5.470 1.00 4.43 H

ATOM 356 CA THR A 52 14.127 -7.717 -4.857 1.00 4.42 C

ATOM 357 HA THR A 52 14.982 -7.152 -5.225 1.00 4.42 H

ATOM 358 C THR A 52 14.417 -9.207 -5.016 1.00 4.05 C

ATOM 359 O THR A 52 13.535 -10.041 -4.814 1.00 4.74 O

ATOM 360 CB THR A 52 13.951 -7.405 -3.358 1.00 6.38 C

ATOM 361 HB THR A 52 13.160 -8.034 -2.951 1.00 6.38 H

ATOM 362 OG1 THR A 52 13.581 -6.030 -3.203 1.00 7.81 O

ATOM 363 HG1 THR A 52 12.873 -5.839 -3.821 1.00 7.81 H

ATOM 364 CG2 THR A 52 15.241 -7.646 -2.582 1.00 8.46 C

ATOM 365 1HG2 THR A 52 16.063 -7.106 -3.054 1.00 8.46 H

ATOM 366 2HG2 THR A 52 15.468 -8.712 -2.567 1.00 8.46 H

ATOM 367 3HG2 THR A 52 15.119 -7.291 -1.559 1.00 8.46 H

ATOM 368 N ALA A 53 15.653 -9.543 -5.370 1.00 3.48 N

ATOM 369 H ALA A 53 16.342 -8.821 -5.534 1.00 3.48 H

ATOM 370 CA ALA A 53 16.063 -10.933 -5.549 1.00 3.86 C

ATOM 371 HA ALA A 53 15.291 -11.581 -5.135 1.00 3.86 H

ATOM 372 C ALA A 53 17.374 -11.224 -4.821 1.00 3.97 C

ATOM 373 O ALA A 53 18.334 -10.463 -4.942 1.00 4.44 O

ATOM 374 CB ALA A 53 16.204 -11.234 -7.035 1.00 4.72 C

ATOM 375 1HB ALA A 53 16.477 -12.279 -7.173 1.00 4.72 H

ATOM 376 2HB ALA A 53 16.978 -10.599 -7.466 1.00 4.72 H

ATOM 377 3HB ALA A 53 15.258 -11.041 -7.540 1.00 4.72 H

ATOM 378 N LYS A 54 17.423 -12.305 -4.049 1.00 3.86 N

ATOM 379 H LYS A 54 16.605 -12.895 -3.965 1.00 3.86 H

ATOM 380 CA LYS A 54 18.620 -12.660 -3.286 1.00 3.61 C

ATOM 381 HA LYS A 54 19.473 -12.112 -3.690 1.00 3.61 H

ATOM 382 C LYS A 54 18.936 -14.150 -3.358 1.00 3.76 C

ATOM 383 O LYS A 54 18.073 -14.979 -3.074 1.00 4.35 O

ATOM 384 CB LYS A 54 18.443 -12.277 -1.814 1.00 0.00 C

ATOM 385 1HB LYS A 54 17.582 -12.816 -1.418 1.00 0.00 H

ATOM 386 2HB LYS A 54 19.328 -12.601 -1.266 1.00 0.00 H

ATOM 387 CG LYS A 54 18.239 -10.789 -1.566 1.00 0.00 C

ATOM 388 1HG LYS A 54 19.082 -10.237 -1.983 1.00 0.00 H

ATOM 389 2HG LYS A 54 17.322 -10.463 -2.057 1.00 0.00 H

ATOM 390 CD LYS A 54 18.135 -10.493 -0.077 1.00 0.00 C

ATOM 391 1HD LYS A 54 17.321 -11.081 0.347 1.00 0.00 H

ATOM 392 2HD LYS A 54 19.069 -10.776 0.409 1.00 0.00 H

ATOM 393 CE LYS A 54 17.867 -9.017 0.170 1.00 0.00 C

ATOM 394 1HE LYS A 54 18.660 -8.429 -0.299 1.00 0.00 H

ATOM 395 2HE LYS A 54 16.917 -8.746 -0.297 1.00 0.00 H

ATOM 396 NZ LYS A 54 17.824 -8.764 1.648 1.00 0.00 N

ATOM 397 1HZ LYS A 54 18.701 -9.027 2.076 1.00 0.00 H

ATOM 398 2HZ LYS A 54 17.083 -9.302 2.077 1.00 0.00 H

ATOM 399 3HZ LYS A 54 17.658 -7.783 1.833 1.00 0.00 H

ATOM 400 N SER A 55 20.172 -14.491 -3.705 1.00 3.50 N

ATOM 401 H SER A 55 20.853 -13.773 -3.911 1.00 3.50 H

ATOM 402 CA SER A 55 20.581 -15.889 -3.813 1.00 3.85 C

ATOM 403 HA SER A 55 19.777 -16.523 -3.441 1.00 3.85 H

ATOM 404 C SER A 55 21.840 -16.193 -3.007 1.00 3.48 C

ATOM 405 O SER A 55 22.748 -15.368 -2.921 1.00 5.07 O

ATOM 406 CB SER A 55 20.837 -16.237 -5.281 1.00 4.32 C

ATOM 407 1HB SER A 55 21.628 -15.594 -5.668 1.00 4.32 H

ATOM 408 2HB SER A 55 21.155 -17.277 -5.356 1.00 4.32 H

ATOM 409 OG SER A 55 19.666 -16.049 -6.054 1.00 5.06 O

ATOM 410 HG SER A 55 19.919 -16.006 -6.981 1.00 5.06 H

ATOM 411 N MET A 56 21.898 -17.388 -2.428 1.00 3.82 N

ATOM 412 H MET A 56 21.104 -18.013 -2.473 1.00 3.82 H

ATOM 413 CA MET A 56 23.081 -17.842 -1.704 1.00 3.77 C

ATOM 414 HA MET A 56 23.958 -17.391 -2.165 1.00 3.77 H

ATOM 415 C MET A 56 23.220 -19.359 -1.794 1.00 3.75 C

ATOM 416 O MET A 56 22.220 -20.074 -1.845 1.00 4.60 O

ATOM 417 CB MET A 56 23.018 -17.399 -0.239 1.00 0.00 C

ATOM 418 1HB MET A 56 23.958 -17.665 0.244 1.00 0.00 H

ATOM 419 2HB MET A 56 22.915 -16.314 -0.210 1.00 0.00 H

ATOM 420 CG MET A 56 21.867 -18.012 0.546 1.00 0.00 C

ATOM 421 1HG MET A 56 20.930 -17.751 0.054 1.00 0.00 H

ATOM 422 2HG MET A 56 21.971 -19.097 0.538 1.00 0.00 H

ATOM 423 SD MET A 56 21.790 -17.448 2.258 1.00 0.00 S

ATOM 424 CE MET A 56 23.159 -18.338 3.010 1.00 0.00 C

ATOM 425 1HE MET A 56 24.098 -18.013 2.563 1.00 0.00 H

ATOM 426 2HE MET A 56 23.031 -19.408 2.850 1.00 0.00 H

ATOM 427 3HE MET A 56 23.179 -18.132 4.081 1.00 0.00 H

ATOM 428 N SER A 57 24.450 -19.860 -1.835 1.00 3.70 N

ATOM 429 H SER A 57 25.255 -19.250 -1.799 1.00 3.70 H

ATOM 430 CA SER A 57 24.677 -21.297 -1.959 1.00 3.84 C

ATOM 431 HA SER A 57 23.890 -21.816 -1.414 1.00 3.84 H

ATOM 432 C SER A 57 26.020 -21.731 -1.379 1.00 4.67 C

ATOM 433 O SER A 57 26.987 -20.971 -1.421 1.00 4.48 O

ATOM 434 CB SER A 57 24.597 -21.697 -3.434 1.00 4.96 C

ATOM 435 1HB SER A 57 24.762 -22.770 -3.528 1.00 4.96 H

ATOM 436 2HB SER A 57 23.604 -21.457 -3.815 1.00 4.96 H

ATOM 437 OG SER A 57 25.563 -21.001 -4.203 1.00 5.59 O

ATOM 438 HG SER A 57 25.466 -21.263 -5.122 1.00 5.59 H

ATOM 439 N THR A 58 26.085 -22.940 -0.829 1.00 4.69 N

ATOM 440 H THR A 58 25.261 -23.525 -0.788 1.00 4.69 H

ATOM 441 CA THR A 58 27.333 -23.449 -0.263 1.00 5.34 C

ATOM 442 HA THR A 58 28.126 -22.775 -0.580 1.00 5.34 H

ATOM 443 C THR A 58 27.692 -24.856 -0.733 1.00 5.71 C

ATOM 444 O THR A 58 26.826 -25.720 -0.866 1.00 5.86 O

ATOM 445 CB THR A 58 27.336 -23.471 1.279 1.00 6.55 C

ATOM 446 HB THR A 58 28.323 -23.778 1.623 1.00 6.55 H

ATOM 447 OG1 THR A 58 26.371 -24.419 1.749 1.00 7.24 O

ATOM 448 HG1 THR A 58 26.304 -24.333 2.702 1.00 7.24 H

ATOM 449 CG2 THR A 58 27.015 -22.101 1.866 1.00 7.81 C

ATOM 450 1HG2 THR A 58 27.154 -22.130 2.947 1.00 7.81 H

ATOM 451 2HG2 THR A 58 25.981 -21.836 1.646 1.00 7.81 H

ATOM 452 3HG2 THR A 58 27.681 -21.352 1.439 1.00 7.81 H

ATOM 453 N GLY A 59 28.980 -25.082 -0.973 1.00 6.42 N

ATOM 454 H GLY A 59 29.642 -24.326 -0.857 1.00 6.42 H

ATOM 455 CA GLY A 59 29.471 -26.358 -1.463 1.00 6.74 C

ATOM 456 1HA GLY A 59 30.396 -26.615 -0.947 1.00 6.74 H

ATOM 457 2HA GLY A 59 28.731 -27.134 -1.269 1.00 6.74 H

ATOM 458 C GLY A 59 29.746 -26.287 -2.953 1.00 6.76 C

ATOM 459 O GLY A 59 30.662 -25.588 -3.386 1.00 8.12 O

ATOM 460 N ARG A 60 28.938 -26.996 -3.734 1.00 6.31 N

ATOM 461 H ARG A 60 28.292 -27.648 -3.305 1.00 6.31 H

ATOM 462 CA ARG A 60 28.864 -26.823 -5.185 1.00 7.22 C

ATOM 463 HA ARG A 60 29.479 -25.975 -5.489 1.00 7.22 H

ATOM 464 C ARG A 60 27.418 -26.537 -5.583 1.00 7.28 C

ATOM 465 O ARG A 60 27.018 -26.719 -6.731 1.00 10.66 O

ATOM 466 CB ARG A 60 29.353 -28.080 -5.910 1.00 8.68 C

ATOM 467 1HB ARG A 60 28.732 -28.922 -5.604 1.00 8.68 H

ATOM 468 2HB ARG A 60 29.223 -27.933 -6.982 1.00 8.68 H

ATOM 469 CG ARG A 60 30.815 -28.423 -5.649 1.00 10.30 C

ATOM 470 1HG ARG A 60 31.434 -27.572 -5.935 1.00 10.30 H

ATOM 471 2HG ARG A 60 30.954 -28.616 -4.586 1.00 10.30 H

ATOM 472 CD ARG A 60 31.264 -29.645 -6.440 1.00 12.50 C

ATOM 473 1HD ARG A 60 31.104 -29.451 -7.502 1.00 12.50 H

ATOM 474 2HD ARG A 60 32.331 -29.800 -6.281 1.00 12.50 H

ATOM 475 NE ARG A 60 30.530 -30.854 -6.063 1.00 14.16 N

ATOM 476 HE ARG A 60 29.752 -31.116 -6.649 1.00 14.16 H

ATOM 477 CZ ARG A 60 30.796 -31.629 -5.019 1.00 16.01 C

ATOM 478 NH1 ARG A 60 31.793 -31.395 -4.188 1.00 18.66 N

ATOM 479 1HH1 ARG A 60 32.393 -30.601 -4.346 1.00 18.66 H

ATOM 480 2HH1 ARG A 60 31.945 -32.020 -3.412 1.00 18.66 H

ATOM 481 NH2 ARG A 60 30.033 -32.679 -4.797 1.00 14.46 N

ATOM 482 1HH2 ARG A 60 29.279 -32.884 -5.434 1.00 14.46 H

ATOM 483 2HH2 ARG A 60 30.216 -33.270 -4.001 1.00 14.46 H

ATOM 484 N ALA A 61 26.632 -26.124 -4.595 1.00 6.27 N

ATOM 485 H ALA A 61 27.038 -25.963 -3.684 1.00 6.27 H

ATOM 486 CA ALA A 61 25.196 -25.918 -4.745 1.00 5.65 C

ATOM 487 HA ALA A 61 24.760 -26.809 -5.196 1.00 5.65 H

ATOM 488 C ALA A 61 24.857 -24.732 -5.644 1.00 5.32 C

ATOM 489 O ALA A 61 25.689 -23.855 -5.876 1.00 6.32 O

ATOM 490 CB ALA A 61 24.582 -25.733 -3.365 1.00 0.00 C

ATOM 491 1HB ALA A 61 24.796 -26.607 -2.750 1.00 0.00 H

ATOM 492 2HB ALA A 61 23.503 -25.620 -3.459 1.00 0.00 H

ATOM 493 3HB ALA A 61 25.003 -24.847 -2.890 1.00 0.00 H

ATOM 494 N THR A 62 23.633 -24.716 -6.160 1.00 4.60 N

ATOM 495 H THR A 62 22.983 -25.454 -5.920 1.00 4.60 H

ATOM 496 CA THR A 62 23.182 -23.674 -7.079 1.00 4.61 C

ATOM 497 HA THR A 62 23.970 -22.929 -7.178 1.00 4.61 H

ATOM 498 C THR A 62 21.916 -22.987 -6.573 1.00 4.46 C

ATOM 499 O THR A 62 20.923 -23.650 -6.275 1.00 4.81 O

ATOM 500 CB THR A 62 22.897 -24.260 -8.477 1.00 6.27 C

ATOM 501 HB THR A 62 22.066 -24.961 -8.411 1.00 6.27 H

ATOM 502 OG1 THR A 62 24.062 -24.953 -8.941 1.00 8.08 O

ATOM 503 HG1 THR A 62 24.329 -25.581 -8.266 1.00 8.08 H

ATOM 504 CG2 THR A 62 22.553 -23.167 -9.482 1.00 7.68 C

ATOM 505 1HG2 THR A 62 23.340 -22.412 -9.491 1.00 7.68 H

ATOM 506 2HG2 THR A 62 21.605 -22.702 -9.213 1.00 7.68 H

ATOM 507 3HG2 THR A 62 22.465 -23.606 -10.476 1.00 7.68 H

ATOM 508 N ALA A 63 21.940 -21.661 -6.485 1.00 3.88 N

ATOM 509 H ALA A 63 22.783 -21.158 -6.727 1.00 3.88 H

ATOM 510 CA ALA A 63 20.774 -20.889 -6.065 1.00 3.86 C

ATOM 511 HA ALA A 63 19.911 -21.553 -6.020 1.00 3.86 H

ATOM 512 C ALA A 63 20.466 -19.775 -7.064 1.00 3.67 C

ATOM 513 O ALA A 63 21.331 -18.957 -7.376 1.00 4.75 O

ATOM 514 CB ALA A 63 21.008 -20.308 -4.679 1.00 4.25 C

ATOM 515 1HB ALA A 63 21.920 -19.711 -4.680 1.00 4.25 H

ATOM 516 2HB ALA A 63 21.105 -21.119 -3.957 1.00 4.25 H

ATOM 517 3HB ALA A 63 20.166 -19.676 -4.399 1.00 4.25 H

ATOM 518 N THR A 64 19.243 -19.753 -7.582 1.00 3.90 N

ATOM 519 H THR A 64 18.555 -20.430 -7.276 1.00 3.90 H

ATOM 520 CA THR A 64 18.865 -18.816 -8.638 1.00 4.12 C

ATOM 521 HA THR A 64 19.648 -18.067 -8.747 1.00 4.12 H

ATOM 522 C THR A 64 17.546 -18.110 -8.335 1.00 3.87 C

ATOM 523 O THR A 64 16.551 -18.763 -8.019 1.00 4.48 O

ATOM 524 CB THR A 64 18.711 -19.557 -9.982 1.00 5.25 C

ATOM 525 HB THR A 64 17.857 -20.232 -9.923 1.00 5.25 H

ATOM 526 OG1 THR A 64 19.895 -20.323 -10.237 1.00 6.91 O

ATOM 527 HG1 THR A 64 19.783 -20.784 -11.072 1.00 6.91 H

ATOM 528 CG2 THR A 64 18.502 -18.587 -11.139 1.00 6.15 C

ATOM 529 1HG2 THR A 64 17.544 -18.080 -11.028 1.00 6.15 H

ATOM 530 2HG2 THR A 64 18.507 -19.140 -12.078 1.00 6.15 H

ATOM 531 3HG2 THR A 64 19.305 -17.849 -11.152 1.00 6.15 H

ATOM 532 N THR A 65 17.532 -16.785 -8.440 1.00 3.77 N

ATOM 533 H THR A 65 18.383 -16.289 -8.667 1.00 3.77 H

ATOM 534 CA THR A 65 16.319 -15.998 -8.232 1.00 3.65 C

ATOM 535 HA THR A 65 15.464 -16.668 -8.154 1.00 3.65 H

ATOM 536 C THR A 65 16.055 -15.009 -9.363 1.00 3.37 C

ATOM 537 O THR A 65 16.971 -14.338 -9.837 1.00 4.35 O

ATOM 538 CB THR A 65 16.396 -15.152 -6.946 1.00 3.95 C

ATOM 539 HB THR A 65 15.529 -14.494 -6.898 1.00 3.95 H

ATOM 540 OG1 THR A 65 17.593 -14.366 -6.960 1.00 5.08 O

ATOM 541 HG1 THR A 65 18.325 -14.929 -6.697 1.00 5.08 H

ATOM 542 CG2 THR A 65 16.418 -16.025 -5.707 1.00 4.33 C

ATOM 543 1HG2 THR A 65 16.470 -15.386 -4.827 1.00 4.33 H

ATOM 544 2HG2 THR A 65 17.288 -16.681 -5.728 1.00 4.33 H

ATOM 545 3HG2 THR A 65 15.509 -16.623 -5.661 1.00 4.33 H

ATOM 546 N THR A 66 14.797 -14.906 -9.779 1.00 3.37 N

ATOM 547 H THR A 66 14.106 -15.572 -9.461 1.00 3.37 H

ATOM 548 CA THR A 66 14.363 -13.836 -10.675 1.00 3.75 C

ATOM 549 HA THR A 66 15.150 -13.084 -10.729 1.00 3.75 H

ATOM 550 C THR A 66 13.090 -13.185 -10.141 1.00 3.54 C

ATOM 551 O THR A 66 12.042 -13.828 -10.074 1.00 5.79 O

ATOM 552 CB THR A 66 14.095 -14.353 -12.102 1.00 5.11 C

ATOM 553 HB THR A 66 13.239 -15.028 -12.087 1.00 5.11 H

ATOM 554 OG1 THR A 66 15.247 -15.066 -12.568 1.00 5.90 O

ATOM 555 HG1 THR A 66 15.515 -15.684 -11.884 1.00 5.90 H

ATOM 556 CG2 THR A 66 13.811 -13.204 -13.062 1.00 6.45 C

ATOM 557 1HG2 THR A 66 14.639 -12.495 -13.044 1.00 6.45 H

ATOM 558 2HG2 THR A 66 12.892 -12.697 -12.769 1.00 6.45 H

ATOM 559 3HG2 THR A 66 13.696 -13.597 -14.072 1.00 6.45 H

ATOM 560 N ALA A 67 13.181 -11.920 -9.745 1.00 3.83 N

ATOM 561 H ALA A 67 14.069 -11.439 -9.799 1.00 3.83 H

ATOM 562 CA ALA A 67 12.035 -11.185 -9.217 1.00 4.10 C

ATOM 563 HA ALA A 67 11.208 -11.879 -9.065 1.00 4.10 H

ATOM 564 C ALA A 67 11.583 -10.099 -10.192 1.00 3.90 C

ATOM 565 O ALA A 67 12.406 -9.445 -10.833 1.00 4.80 O

ATOM 566 CB ALA A 67 12.391 -10.573 -7.869 1.00 5.06 C

ATOM 567 1HB ALA A 67 12.817 -11.338 -7.220 1.00 5.06 H

ATOM 568 2HB ALA A 67 11.490 -10.174 -7.403 1.00 5.06 H

ATOM 569 3HB ALA A 67 13.117 -9.772 -8.008 1.00 5.06 H

ATOM 570 N THR A 68 10.274 -9.912 -10.323 1.00 4.51 N

ATOM 571 H THR A 68 9.630 -10.459 -9.767 1.00 4.51 H

ATOM 572 CA THR A 68 9.716 -8.980 -11.300 1.00 5.31 C

ATOM 573 HA THR A 68 10.519 -8.363 -11.703 1.00 5.31 H

ATOM 574 C THR A 68 8.663 -8.067 -10.676 1.00 5.30 C

ATOM 575 O THR A 68 7.839 -8.513 -9.878 1.00 5.92 O

ATOM 576 CB THR A 68 9.074 -9.768 -12.462 1.00 5.98 C

ATOM 577 HB THR A 68 8.200 -10.302 -12.092 1.00 5.98 H

ATOM 578 OG1 THR A 68 10.020 -10.719 -12.966 1.00 6.43 O

ATOM 579 HG1 THR A 68 10.864 -10.277 -13.082 1.00 6.43 H

ATOM 580 CG2 THR A 68 8.654 -8.855 -13.608 1.00 7.59 C

ATOM 581 1HG2 THR A 68 8.323 -9.463 -14.449 1.00 7.59 H

ATOM 582 2HG2 THR A 68 9.498 -8.240 -13.919 1.00 7.59 H

ATOM 583 3HG2 THR A 68 7.833 -8.214 -13.287 1.00 7.59 H

ATOM 584 N GLY A 69 8.676 -6.789 -11.037 1.00 5.31 N

ATOM 585 H GLY A 69 9.391 -6.441 -11.661 1.00 5.31 H

ATOM 586 CA GLY A 69 7.687 -5.854 -10.529 1.00 6.16 C

ATOM 587 1HA GLY A 69 7.537 -5.054 -11.253 1.00 6.16 H

ATOM 588 2HA GLY A 69 6.741 -6.372 -10.376 1.00 6.16 H

ATOM 589 C GLY A 69 8.135 -5.242 -9.216 1.00 5.64 C

ATOM 590 O GLY A 69 9.024 -4.396 -9.209 1.00 6.88 O

ATOM 591 N THR A 70 7.536 -5.671 -8.111 1.00 6.21 N

ATOM 592 H THR A 70 6.789 -6.350 -8.175 1.00 6.21 H

ATOM 593 CA THR A 70 7.915 -5.226 -6.771 1.00 6.87 C

ATOM 594 HA THR A 70 8.909 -4.779 -6.801 1.00 6.87 H

ATOM 595 C THR A 70 7.942 -6.450 -5.856 1.00 6.33 C

ATOM 596 O THR A 70 7.711 -6.372 -4.650 1.00 6.73 O

ATOM 597 CB THR A 70 6.918 -4.173 -6.243 1.00 8.01 C

ATOM 598 HB THR A 70 6.002 -4.673 -5.931 1.00 8.01 H

ATOM 599 OG1 THR A 70 6.605 -3.256 -7.298 1.00 9.95 O

ATOM 600 HG1 THR A 70 6.423 -3.770 -8.098 1.00 9.95 H

ATOM 601 CG2 THR A 70 7.488 -3.374 -5.075 1.00 9.91 C

ATOM 602 1HG2 THR A 70 8.480 -3.001 -5.329 1.00 9.91 H

ATOM 603 2HG2 THR A 70 7.554 -4.005 -4.189 1.00 9.91 H

ATOM 604 3HG2 THR A 70 6.831 -2.530 -4.862 1.00 9.91 H

ATOM 605 N ALA A 71 8.192 -7.606 -6.461 1.00 5.67 N

ATOM 606 H ALA A 71 8.398 -7.609 -7.450 1.00 5.67 H

ATOM 607 CA ALA A 71 8.133 -8.893 -5.776 1.00 5.03 C

ATOM 608 HA ALA A 71 7.341 -8.857 -5.027 1.00 5.03 H

ATOM 609 C ALA A 71 9.435 -9.257 -5.067 1.00 4.55 C

ATOM 610 O ALA A 71 10.480 -8.654 -5.313 1.00 6.17 O

ATOM 611 CB ALA A 71 7.776 -9.968 -6.790 1.00 5.98 C

ATOM 612 1HB ALA A 71 6.861 -9.686 -7.312 1.00 5.98 H

ATOM 613 2HB ALA A 71 7.615 -10.915 -6.277 1.00 5.98 H

ATOM 614 3HB ALA A 71 8.587 -10.078 -7.510 1.00 5.98 H

ATOM 615 N MET A 72 9.365 -10.261 -4.200 1.00 5.02 N

ATOM 616 H MET A 72 8.476 -10.719 -4.047 1.00 5.02 H

ATOM 617 CA MET A 72 10.524 -10.768 -3.470 1.00 5.27 C

ATOM 618 HA MET A 72 11.408 -10.190 -3.739 1.00 5.27 H

ATOM 619 C MET A 72 10.773 -12.233 -3.821 1.00 4.56 C

ATOM 620 O MET A 72 9.861 -13.055 -3.746 1.00 5.78 O

ATOM 621 CB MET A 72 10.281 -10.640 -1.963 1.00 0.00 C

ATOM 622 1HB MET A 72 10.181 -9.583 -1.716 1.00 0.00 H

ATOM 623 2HB MET A 72 9.337 -11.132 -1.729 1.00 0.00 H

ATOM 624 CG MET A 72 11.364 -11.255 -1.085 1.00 0.00 C

ATOM 625 1HG MET A 72 11.034 -11.215 -0.046 1.00 0.00 H

ATOM 626 2HG MET A 72 11.485 -12.303 -1.358 1.00 0.00 H

ATOM 627 SD MET A 72 12.967 -10.436 -1.216 1.00 0.00 S

ATOM 628 CE MET A 72 12.766 -9.069 -0.066 1.00 0.00 C

ATOM 629 1HE MET A 72 12.506 -9.456 0.919 1.00 0.00 H

ATOM 630 2HE MET A 72 11.973 -8.410 -0.418 1.00 0.00 H

ATOM 631 3HE MET A 72 13.699 -8.510 0.000 1.00 0.00 H

ATOM 632 N ALA A 73 12.008 -12.561 -4.187 1.00 4.40 N

ATOM 633 H ALA A 73 12.719 -11.843 -4.250 1.00 4.40 H

ATOM 634 CA ALA A 73 12.394 -13.938 -4.481 1.00 4.40 C

ATOM 635 HA ALA A 73 11.635 -14.610 -4.079 1.00 4.40 H

ATOM 636 C ALA A 73 13.730 -14.273 -3.823 1.00 4.18 C

ATOM 637 O ALA A 73 14.719 -13.568 -4.028 1.00 4.82 O

ATOM 638 CB ALA A 73 12.483 -14.148 -5.988 1.00 5.16 C

ATOM 639 1HB ALA A 73 12.790 -15.173 -6.196 1.00 5.16 H

ATOM 640 2HB ALA A 73 13.218 -13.462 -6.410 1.00 5.16 H

ATOM 641 3HB ALA A 73 11.510 -13.962 -6.443 1.00 5.16 H

ATOM 642 N THR A 74 13.766 -15.336 -3.025 1.00 4.36 N

ATOM 643 H THR A 74 12.922 -15.868 -2.854 1.00 4.36 H

ATOM 644 CA THR A 74 14.994 -15.756 -2.353 1.00 4.45 C

ATOM 645 HA THR A 74 15.821 -15.162 -2.739 1.00 4.45 H

ATOM 646 C THR A 74 15.311 -17.230 -2.588 1.00 4.26 C

ATOM 647 O THR A 74 14.403 -18.057 -2.672 1.00 5.49 O

ATOM 648 CB THR A 74 14.922 -15.543 -0.827 1.00 5.48 C

ATOM 649 HB THR A 74 15.862 -15.865 -0.380 1.00 5.48 H

ATOM 650 OG1 THR A 74 13.852 -16.329 -0.287 1.00 7.20 O

ATOM 651 HG1 THR A 74 13.965 -17.235 -0.583 1.00 7.20 H

ATOM 652 CG2 THR A 74 14.684 -14.078 -0.477 1.00 5.54 C

ATOM 653 1HG2 THR A 74 13.681 -13.783 -0.784 1.00 5.54 H

ATOM 654 2HG2 THR A 74 15.418 -13.453 -0.985 1.00 5.54 H

ATOM 655 3HG2 THR A 74 14.782 -13.944 0.600 1.00 5.54 H

ATOM 656 N SER A 75 16.594 -17.568 -2.680 1.00 3.54 N

ATOM 657 H SER A 75 17.307 -16.851 -2.649 1.00 3.54 H

ATOM 658 CA SER A 75 17.019 -18.957 -2.826 1.00 3.40 C

ATOM 659 HA SER A 75 16.205 -19.607 -2.503 1.00 3.40 H

ATOM 660 C SER A 75 18.245 -19.267 -1.971 1.00 3.84 C

ATOM 661 O SER A 75 19.171 -18.460 -1.883 1.00 4.30 O

ATOM 662 CB SER A 75 17.342 -19.271 -4.288 1.00 4.27 C

ATOM 663 1HB SER A 75 18.123 -18.594 -4.636 1.00 4.27 H

ATOM 664 2HB SER A 75 17.704 -20.296 -4.362 1.00 4.27 H

ATOM 665 OG SER A 75 16.197 -19.126 -5.110 1.00 5.04 O

ATOM 666 HG SER A 75 16.459 -19.235 -6.028 1.00 5.04 H

ATOM 667 N ASN A 76 18.232 -20.435 -1.339 1.00 3.67 N

ATOM 668 H ASN A 76 17.420 -21.034 -1.419 1.00 3.67 H

ATOM 669 CA ASN A 76 19.332 -20.902 -0.501 1.00 3.84 C

ATOM 670 HA ASN A 76 20.243 -20.361 -0.756 1.00 3.84 H

ATOM 671 C ASN A 76 19.557 -22.395 -0.734 1.00 4.05 C

ATOM 672 O ASN A 76 18.631 -23.190 -0.576 1.00 5.18 O

ATOM 673 CB ASN A 76 18.987 -20.644 0.968 1.00 4.39 C

ATOM 674 1HB ASN A 76 18.678 -19.605 1.082 1.00 4.39 H

ATOM 675 2HB ASN A 76 18.159 -21.291 1.254 1.00 4.39 H

ATOM 676 CG ASN A 76 20.151 -20.908 1.898 1.00 4.83 C

ATOM 677 OD1 ASN A 76 21.245 -21.271 1.473 1.00 6.01 O

ATOM 678 ND2 ASN A 76 19.916 -20.736 3.189 1.00 6.14 N

ATOM 679 1HD2 ASN A 76 20.658 -20.901 3.851 1.00 6.14 H

ATOM 680 2HD2 ASN A 76 19.005 -20.433 3.501 1.00 6.14 H

ATOM 681 N ALA A 77 20.768 -22.785 -1.116 1.00 3.89 N

ATOM 682 H ALA A 77 21.513 -22.107 -1.200 1.00 3.89 H

ATOM 683 CA ALA A 77 21.066 -24.184 -1.414 1.00 3.82 C

ATOM 684 HA ALA A 77 20.263 -24.804 -1.016 1.00 3.82 H

ATOM 685 C ALA A 77 22.372 -24.635 -0.762 1.00 4.18 C

ATOM 686 O ALA A 77 23.383 -23.938 -0.835 1.00 4.88 O

ATOM 687 CB ALA A 77 21.123 -24.387 -2.921 1.00 4.50 C

ATOM 688 1HB ALA A 77 20.187 -24.057 -3.370 1.00 4.50 H

ATOM 689 2HB ALA A 77 21.270 -25.444 -3.138 1.00 4.50 H

ATOM 690 3HB ALA A 77 21.948 -23.810 -3.339 1.00 4.50 H

ATOM 691 N ILE A 78 22.349 -25.790 -0.106 1.00 4.79 N

ATOM 692 H ILE A 78 21.507 -26.349 -0.110 1.00 4.79 H

ATOM 693 CA ILE A 78 23.482 -26.264 0.686 1.00 5.53 C

ATOM 694 HA ILE A 78 24.325 -25.585 0.551 1.00 5.53 H

ATOM 695 C ILE A 78 23.907 -27.662 0.242 1.00 6.16 C

ATOM 696 O ILE A 78 23.085 -28.577 0.199 1.00 9.89 O

ATOM 697 CB ILE A 78 23.113 -26.319 2.189 1.00 6.75 C

ATOM 698 HB ILE A 78 22.307 -27.045 2.298 1.00 6.75 H

ATOM 699 CG1 ILE A 78 22.579 -24.976 2.707 1.00 8.73 C

ATOM 700 1HG1 ILE A 78 21.696 -24.704 2.129 1.00 8.73 H

ATOM 701 2HG1 ILE A 78 22.256 -25.121 3.738 1.00 8.73 H

ATOM 702 CG2 ILE A 78 24.291 -26.809 3.030 1.00 8.69 C

ATOM 703 1HG2 ILE A 78 24.510 -27.849 2.785 1.00 8.69 H

ATOM 704 2HG2 ILE A 78 24.042 -26.741 4.089 1.00 8.69 H

ATOM 705 3HG2 ILE A 78 25.174 -26.203 2.829 1.00 8.69 H

ATOM 706 CD1 ILE A 78 23.537 -23.796 2.687 1.00 10.77 C

ATOM 707 1HD1 ILE A 78 24.392 -24.003 3.330 1.00 10.77 H

ATOM 708 2HD1 ILE A 78 23.022 -22.911 3.059 1.00 10.77 H

ATOM 709 3HD1 ILE A 78 23.879 -23.610 1.669 1.00 10.77 H

ATOM 710 N GLY A 79 25.186 -27.829 -0.077 1.00 5.90 N

ATOM 711 H GLY A 79 25.817 -27.038 -0.054 1.00 5.90 H

ATOM 712 CA GLY A 79 25.722 -29.111 -0.503 1.00 5.96 C

ATOM 713 1HA GLY A 79 26.640 -29.321 0.046 1.00 5.96 H

ATOM 714 2HA GLY A 79 25.000 -29.900 -0.295 1.00 5.96 H

ATOM 715 C GLY A 79 26.036 -29.105 -1.986 1.00 5.73 C

ATOM 716 O GLY A 79 27.017 -28.506 -2.425 1.00 6.97 O

ATOM 717 N GLU A 80 25.179 -29.753 -2.765 1.00 5.47 N

ATOM 718 H GLU A 80 24.427 -30.288 -2.350 1.00 5.47 H

ATOM 719 CA GLU A 80 25.209 -29.688 -4.224 1.00 5.26 C

ATOM 720 HA GLU A 80 25.762 -28.803 -4.537 1.00 5.26 H

ATOM 721 C GLU A 80 23.764 -29.546 -4.702 1.00 4.99 C

ATOM 722 O GLU A 80 23.394 -29.944 -5.806 1.00 6.03 O

ATOM 723 CB GLU A 80 25.895 -30.922 -4.819 1.00 0.00 C

ATOM 724 1HB GLU A 80 25.916 -30.817 -5.904 1.00 0.00 H

ATOM 725 2HB GLU A 80 26.926 -30.942 -4.464 1.00 0.00 H

ATOM 726 CG GLU A 80 25.249 -32.256 -4.470 1.00 0.00 C

ATOM 727 1HG GLU A 80 25.196 -32.357 -3.386 1.00 0.00 H

ATOM 728 2HG GLU A 80 24.236 -32.280 -4.873 1.00 0.00 H

ATOM 729 CD GLU A 80 26.031 -33.428 -5.030 1.00 0.00 C

ATOM 730 OE1 GLU A 80 26.163 -33.534 -6.268 1.00 0.00 O

ATOM 731 OE2 GLU A 80 26.528 -34.253 -4.234 1.00 0.00 O

ATOM 732 N ALA A 81 22.945 -28.978 -3.823 1.00 4.84 N

ATOM 733 H ALA A 81 23.330 -28.652 -2.948 1.00 4.84 H

ATOM 734 CA ALA A 81 21.508 -28.842 -4.027 1.00 5.06 C

ATOM 735 HA ALA A 81 21.129 -29.761 -4.474 1.00 5.06 H

ATOM 736 C ALA A 81 21.164 -27.680 -4.957 1.00 4.86 C

ATOM 737 O ALA A 81 21.993 -26.805 -5.210 1.00 5.96 O

ATOM 738 CB ALA A 81 20.836 -28.649 -2.674 1.00 0.00 C

ATOM 739 1HB ALA A 81 21.157 -27.704 -2.235 1.00 0.00 H

ATOM 740 2HB ALA A 81 21.108 -29.468 -2.009 1.00 0.00 H

ATOM 741 3HB ALA A 81 19.755 -28.641 -2.803 1.00 0.00 H

ATOM 742 N THR A 82 19.934 -27.665 -5.458 1.00 4.82 N

ATOM 743 H THR A 82 19.289 -28.408 -5.221 1.00 4.82 H

ATOM 744 CA THR A 82 19.476 -26.612 -6.361 1.00 4.66 C

ATOM 745 HA THR A 82 20.269 -25.873 -6.467 1.00 4.66 H

ATOM 746 C THR A 82 18.228 -25.912 -5.830 1.00 4.06 C

ATOM 747 O THR A 82 17.218 -26.556 -5.553 1.00 5.28 O

ATOM 748 CB THR A 82 19.165 -27.188 -7.756 1.00 5.99 C

ATOM 749 HB THR A 82 18.320 -27.872 -7.683 1.00 5.99 H

ATOM 750 OG1 THR A 82 20.310 -27.905 -8.233 1.00 9.53 O

ATOM 751 HG1 THR A 82 20.583 -28.524 -7.552 1.00 9.53 H

ATOM 752 CG2 THR A 82 18.834 -26.085 -8.754 1.00 6.56 C

ATOM 753 1HG2 THR A 82 18.712 -26.520 -9.746 1.00 6.56 H

ATOM 754 2HG2 THR A 82 19.641 -25.353 -8.778 1.00 6.56 H

ATOM 755 3HG2 THR A 82 17.906 -25.595 -8.463 1.00 6.56 H

ATOM 756 N ALA A 83 18.283 -24.591 -5.695 1.00 3.77 N

ATOM 757 H ALA A 83 19.137 -24.099 -5.923 1.00 3.77 H

ATOM 758 CA ALA A 83 17.138 -23.810 -5.235 1.00 3.95 C

ATOM 759 HA ALA A 83 16.277 -24.468 -5.117 1.00 3.95 H

ATOM 760 C ALA A 83 16.785 -22.738 -6.263 1.00 4.03 C

ATOM 761 O ALA A 83 17.574 -21.825 -6.510 1.00 4.55 O

ATOM 762 CB ALA A 83 17.449 -23.173 -3.886 1.00 4.86 C

ATOM 763 1HB ALA A 83 18.337 -22.546 -3.971 1.00 4.86 H

ATOM 764 2HB ALA A 83 17.627 -23.953 -3.146 1.00 4.86 H

ATOM 765 3HB ALA A 83 16.605 -22.561 -3.569 1.00 4.86 H

ATOM 766 N THR A 84 15.609 -22.852 -6.872 1.00 4.06 N

ATOM 767 H THR A 84 14.987 -23.615 -6.639 1.00 4.06 H

ATOM 768 CA THR A 84 15.163 -21.892 -7.879 1.00 4.16 C

ATOM 769 HA THR A 84 15.930 -21.130 -8.008 1.00 4.16 H

ATOM 770 C THR A 84 13.877 -21.202 -7.437 1.00 3.71 C

ATOM 771 O THR A 84 12.965 -21.838 -6.908 1.00 5.04 O

ATOM 772 CB THR A 84 14.903 -22.558 -9.246 1.00 5.52 C

ATOM 773 HB THR A 84 14.531 -21.806 -9.942 1.00 5.52 H

ATOM 774 OG1 THR A 84 13.920 -23.590 -9.099 1.00 6.74 O

ATOM 775 HG1 THR A 84 14.222 -24.205 -8.426 1.00 6.74 H

ATOM 776 CG2 THR A 84 16.176 -23.171 -9.816 1.00 6.62 C

ATOM 777 1HG2 THR A 84 16.962 -22.417 -9.858 1.00 6.62 H

ATOM 778 2HG2 THR A 84 15.979 -23.542 -10.822 1.00 6.62 H

ATOM 779 3HG2 THR A 84 16.500 -24.000 -9.186 1.00 6.62 H

ATOM 780 N THR A 85 13.820 -19.889 -7.626 1.00 3.92 N

ATOM 781 H THR A 85 14.599 -19.403 -8.050 1.00 3.92 H

ATOM 782 CA THR A 85 12.693 -19.095 -7.146 1.00 3.64 C

ATOM 783 HA THR A 85 11.822 -19.740 -7.032 1.00 3.64 H

ATOM 784 C THR A 85 12.348 -17.942 -8.084 1.00 3.55 C

ATOM 785 O THR A 85 13.232 -17.195 -8.503 1.00 3.99 O

ATOM 786 CB THR A 85 13.042 -18.492 -5.771 1.00 4.71 C

ATOM 787 HB THR A 85 13.834 -17.756 -5.896 1.00 4.71 H

ATOM 788 OG1 THR A 85 13.496 -19.531 -4.897 1.00 5.62 O

ATOM 789 HG1 THR A 85 13.903 -19.122 -4.129 1.00 5.62 H

ATOM 790 CG2 THR A 85 11.838 -17.829 -5.132 1.00 4.99 C

ATOM 791 1HG2 THR A 85 11.059 -18.573 -4.966 1.00 4.99 H

ATOM 792 2HG2 THR A 85 11.460 -17.038 -5.779 1.00 4.99 H

ATOM 793 3HG2 THR A 85 12.136 -17.397 -4.178 1.00 4.99 H

ATOM 794 N THR A 86 11.068 -17.780 -8.404 1.00 3.62 N

ATOM 795 H THR A 86 10.380 -18.461 -8.108 1.00 3.62 H

ATOM 796 CA THR A 86 10.602 -16.618 -9.157 1.00 3.84 C

ATOM 797 HA THR A 86 11.399 -15.877 -9.191 1.00 3.84 H

ATOM 798 C THR A 86 9.389 -15.988 -8.479 1.00 3.78 C

ATOM 799 O THR A 86 8.639 -16.667 -7.780 1.00 5.73 O

ATOM 800 CB THR A 86 10.206 -16.978 -10.605 1.00 4.70 C

ATOM 801 HB THR A 86 9.858 -16.078 -11.112 1.00 4.70 H

ATOM 802 OG1 THR A 86 9.149 -17.946 -10.587 1.00 5.87 O

ATOM 803 HG1 THR A 86 8.985 -18.230 -11.489 1.00 5.87 H

ATOM 804 CG2 THR A 86 11.384 -17.556 -11.380 1.00 4.71 C

ATOM 805 1HG2 THR A 86 11.111 -17.658 -12.430 1.00 4.71 H

ATOM 806 2HG2 THR A 86 11.644 -18.537 -10.981 1.00 4.71 H

ATOM 807 3HG2 THR A 86 12.243 -16.891 -11.295 1.00 4.71 H

ATOM 808 N ALA A 87 9.190 -14.688 -8.668 1.00 3.83 N

ATOM 809 H ALA A 87 9.835 -14.150 -9.230 1.00 3.83 H

ATOM 810 CA ALA A 87 8.040 -13.998 -8.091 1.00 4.33 C

ATOM 811 HA ALA A 87 7.180 -14.667 -8.105 1.00 4.33 H

ATOM 812 C ALA A 87 7.709 -12.757 -8.915 1.00 4.58 C

ATOM 813 O ALA A 87 8.610 -12.117 -9.458 1.00 5.22 O

ATOM 814 CB ALA A 87 8.334 -13.608 -6.647 1.00 5.11 C

ATOM 815 1HB ALA A 87 7.436 -13.187 -6.195 1.00 5.11 H

ATOM 816 2HB ALA A 87 9.135 -12.870 -6.622 1.00 5.11 H

ATOM 817 3HB ALA A 87 8.638 -14.489 -6.083 1.00 5.11 H

ATOM 818 N THR A 88 6.429 -12.415 -9.019 1.00 4.38 N

ATOM 819 H THR A 88 5.715 -12.973 -8.569 1.00 4.38 H

ATOM 820 CA THR A 88 6.016 -11.230 -9.768 1.00 5.21 C

ATOM 821 HA THR A 88 6.849 -10.529 -9.752 1.00 5.21 H

ATOM 822 C THR A 88 4.821 -10.521 -9.137 1.00 5.34 C

ATOM 823 O THR A 88 4.001 -11.137 -8.456 1.00 7.10 O

ATOM 824 CB THR A 88 5.727 -11.562 -11.247 1.00 6.05 C

ATOM 825 HB THR A 88 6.555 -12.149 -11.645 1.00 6.05 H

ATOM 826 OG1 THR A 88 5.622 -10.344 -11.995 1.00 7.27 O

ATOM 827 HG1 THR A 88 5.434 -10.567 -12.910 1.00 7.27 H

ATOM 828 CG2 THR A 88 4.438 -12.353 -11.417 1.00 8.34 C

ATOM 829 1HG2 THR A 88 3.592 -11.758 -11.074 1.00 8.34 H

ATOM 830 2HG2 THR A 88 4.493 -13.277 -10.841 1.00 8.34 H

ATOM 831 3HG2 THR A 88 4.300 -12.598 -12.470 1.00 8.34 H

ATOM 832 N GLY A 89 4.740 -9.211 -9.350 1.00 5.76 N

ATOM 833 H GLY A 89 5.420 -8.759 -9.945 1.00 5.76 H

ATOM 834 CA GLY A 89 3.732 -8.391 -8.700 1.00 6.31 C

ATOM 835 1HA GLY A 89 3.457 -7.560 -9.348 1.00 6.31 H

ATOM 836 2HA GLY A 89 2.847 -8.994 -8.496 1.00 6.31 H

ATOM 837 C GLY A 89 4.278 -7.843 -7.396 1.00 7.98 C

ATOM 838 O GLY A 89 5.312 -7.178 -7.389 1.00 11.24 O

ATOM 839 N ARG A 93 3.606 -8.146 -6.291 1.00 9.76 N

ATOM 840 H ARG A 93 2.722 -8.635 -6.370 1.00 9.76 H

ATOM 841 CA ARG A 93 4.117 -7.901 -4.943 1.00 9.18 C

ATOM 842 HA ARG A 93 5.140 -7.527 -4.992 1.00 9.18 H

ATOM 843 C ARG A 93 4.135 -9.224 -4.183 1.00 8.26 C

ATOM 844 O ARG A 93 4.018 -9.267 -2.960 1.00 10.36 O

ATOM 845 CB ARG A 93 3.241 -6.878 -4.212 1.00 0.00 C

ATOM 846 1HB ARG A 93 2.202 -7.205 -4.259 1.00 0.00 H

ATOM 847 2HB ARG A 93 3.544 -6.839 -3.166 1.00 0.00 H

ATOM 848 CG ARG A 93 3.355 -5.475 -4.791 1.00 0.00 C

ATOM 849 1HG ARG A 93 4.380 -5.130 -4.662 1.00 0.00 H

ATOM 850 2HG ARG A 93 3.126 -5.505 -5.857 1.00 0.00 H

ATOM 851 CD ARG A 93 2.419 -4.487 -4.113 1.00 0.00 C

ATOM 852 1HD ARG A 93 1.386 -4.804 -4.259 1.00 0.00 H

ATOM 853 2HD ARG A 93 2.631 -4.476 -3.043 1.00 0.00 H

ATOM 854 NE ARG A 93 2.611 -3.139 -4.651 1.00 0.00 N

ATOM 855 HE ARG A 93 3.224 -2.526 -4.135 1.00 0.00 H

ATOM 856 CZ ARG A 93 2.058 -2.660 -5.758 1.00 0.00 C

ATOM 857 NH1 ARG A 93 2.344 -1.430 -6.130 1.00 0.00 N

ATOM 858 1HH1 ARG A 93 2.982 -0.883 -5.573 1.00 0.00 H

ATOM 859 2HH1 ARG A 93 1.931 -1.053 -6.969 1.00 0.00 H

ATOM 860 NH2 ARG A 93 1.241 -3.365 -6.515 1.00 0.00 N

ATOM 861 1HH2 ARG A 93 1.002 -4.306 -6.244 1.00 0.00 H

ATOM 862 2HH2 ARG A 93 0.859 -2.949 -7.350 1.00 0.00 H

ATOM 863 N ALA A 95 4.257 -10.305 -4.943 1.00 5.73 N

ATOM 864 H ALA A 95 4.333 -10.196 -5.944 1.00 5.73 H

ATOM 865 CA ALA A 95 4.273 -11.658 -4.399 1.00 6.16 C

ATOM 866 HA ALA A 95 3.485 -11.755 -3.652 1.00 6.16 H

ATOM 867 C ALA A 95 5.610 -11.959 -3.729 1.00 6.39 C

ATOM 868 O ALA A 95 6.608 -11.279 -3.972 1.00 9.87 O

ATOM 869 CB ALA A 95 4.007 -12.655 -5.518 1.00 7.46 C

ATOM 870 1HB ALA A 95 4.833 -12.638 -6.230 1.00 7.46 H

ATOM 871 2HB ALA A 95 3.081 -12.394 -6.030 1.00 7.46 H

ATOM 872 3HB ALA A 95 3.912 -13.654 -5.096 1.00 7.46 H

ATOM 873 N THR A 96 5.627 -12.989 -2.891 1.00 5.49 N

ATOM 874 H THR A 96 4.776 -13.510 -2.719 1.00 5.49 H

ATOM 875 CA THR A 96 6.846 -13.432 -2.221 1.00 5.70 C

ATOM 876 HA THR A 96 7.700 -12.886 -2.621 1.00 5.70 H

ATOM 877 C THR A 96 7.052 -14.922 -2.466 1.00 5.07 C

ATOM 878 O THR A 96 6.138 -15.722 -2.264 1.00 6.51 O

ATOM 879 CB THR A 96 6.764 -13.184 -0.702 1.00 8.31 C

ATOM 880 HB THR A 96 5.955 -13.782 -0.286 1.00 8.31 H

ATOM 881 OG1 THR A 96 6.490 -11.797 -0.466 1.00 10.93 O

ATOM 882 HG1 THR A 96 5.724 -11.550 -0.989 1.00 10.93 H

ATOM 883 CG2 THR A 96 8.069 -13.546 -0.001 1.00 7.83 C

ATOM 884 1HG2 THR A 96 8.905 -13.052 -0.496 1.00 7.83 H

ATOM 885 2HG2 THR A 96 8.215 -14.626 -0.032 1.00 7.83 H

ATOM 886 3HG2 THR A 96 8.023 -13.222 1.039 1.00 7.83 H

ATOM 887 N SER A 97 8.253 -15.297 -2.892 1.00 4.56 N

ATOM 888 H SER A 97 8.967 -14.605 -3.076 1.00 4.56 H

ATOM 889 CA SER A 97 8.587 -16.704 -3.089 1.00 4.26 C

ATOM 890 HA SER A 97 7.830 -17.307 -2.592 1.00 4.26 H

ATOM 891 C SER A 97 9.947 -17.043 -2.487 1.00 4.41 C

ATOM 892 O SER A 97 10.829 -16.186 -2.411 1.00 5.01 O

ATOM 893 CB SER A 97 8.584 -17.043 -4.580 1.00 0.00 C

ATOM 894 1HB SER A 97 9.348 -16.449 -5.080 1.00 0.00 H

ATOM 895 2HB SER A 97 8.812 -18.101 -4.710 1.00 0.00 H

ATOM 896 OG SER A 97 7.328 -16.763 -5.170 1.00 0.00 O

ATOM 897 HG SER A 97 7.426 -16.806 -6.124 1.00 0.00 H

ATOM 898 N SER A 98 10.123 -18.293 -2.070 1.00 4.24 N

ATOM 899 H SER A 98 9.363 -18.957 -2.129 1.00 4.24 H

ATOM 900 CA SER A 98 11.395 -18.745 -1.511 1.00 4.02 C

ATOM 901 HA SER A 98 12.196 -18.192 -1.999 1.00 4.02 H

ATOM 902 C SER A 98 11.653 -20.236 -1.718 1.00 3.64 C

ATOM 903 O SER A 98 10.721 -21.039 -1.747 1.00 4.07 O

ATOM 904 CB SER A 98 11.434 -18.433 -0.013 1.00 5.86 C

ATOM 905 1HB SER A 98 12.402 -18.725 0.393 1.00 5.86 H

ATOM 906 2HB SER A 98 11.297 -17.361 0.132 1.00 5.86 H

ATOM 907 OG SER A 98 10.406 -19.128 0.670 1.00 8.31 O

ATOM 908 HG SER A 98 9.562 -18.853 0.302 1.00 8.31 H

ATOM 909 N SER A 99 12.923 -20.606 -1.844 1.00 3.19 N

ATOM 910 H SER A 99 13.651 -19.905 -1.857 1.00 3.19 H

ATOM 911 CA SER A 99 13.318 -22.008 -1.955 1.00 3.31 C

ATOM 912 HA SER A 99 12.503 -22.635 -1.593 1.00 3.31 H

ATOM 913 C SER A 99 14.557 -22.278 -1.106 1.00 3.20 C

ATOM 914 O SER A 99 15.545 -21.550 -1.198 1.00 4.21 O

ATOM 915 CB SER A 99 13.607 -22.371 -3.413 1.00 0.00 C

ATOM 916 1HB SER A 99 14.389 -21.715 -3.796 1.00 0.00 H

ATOM 917 2HB SER A 99 13.954 -23.403 -3.464 1.00 0.00 H

ATOM 918 OG SER A 99 12.448 -22.231 -4.216 1.00 0.00 O

ATOM 919 HG SER A 99 12.723 -22.099 -5.127 1.00 0.00 H

ATOM 920 N THR A 100 14.499 -23.312 -0.273 1.00 3.77 N

ATOM 921 H THR A 100 13.656 -23.868 -0.222 1.00 3.77 H

ATOM 922 CA THR A 100 15.616 -23.678 0.596 1.00 3.70 C

ATOM 923 HA THR A 100 16.504 -23.130 0.282 1.00 3.70 H

ATOM 924 C THR A 100 15.893 -25.175 0.497 1.00 3.56 C

ATOM 925 O THR A 100 15.000 -25.987 0.738 1.00 4.14 O

ATOM 926 CB THR A 100 15.321 -23.326 2.068 1.00 4.76 C

ATOM 927 HB THR A 100 14.484 -23.929 2.422 1.00 4.76 H

ATOM 928 OG1 THR A 100 14.971 -21.939 2.158 1.00 5.45 O

ATOM 929 HG1 THR A 100 14.268 -21.767 1.532 1.00 5.45 H

ATOM 930 CG2 THR A 100 16.533 -23.579 2.956 1.00 5.59 C

ATOM 931 1HG2 THR A 100 17.397 -23.042 2.565 1.00 5.59 H

ATOM 932 2HG2 THR A 100 16.752 -24.646 2.987 1.00 5.59 H

ATOM 933 3HG2 THR A 100 16.320 -23.229 3.966 1.00 5.59 H

ATOM 934 N THR A 101 17.111 -25.551 0.121 1.00 4.00 N

ATOM 935 H THR A 101 17.827 -24.857 -0.048 1.00 4.00 H

ATOM 936 CA THR A 101 17.434 -26.956 -0.120 1.00 4.05 C

ATOM 937 HA THR A 101 16.630 -27.571 0.283 1.00 4.05 H

ATOM 938 C THR A 101 18.740 -27.404 0.530 1.00 4.53 C

ATOM 939 O THR A 101 19.635 -26.595 0.776 1.00 5.04 O

ATOM 940 CB THR A 101 17.547 -27.238 -1.632 1.00 4.38 C

ATOM 941 HB THR A 101 17.764 -28.296 -1.784 1.00 4.38 H

ATOM 942 OG1 THR A 101 18.605 -26.447 -2.186 1.00 4.76 O

ATOM 943 HG1 THR A 101 18.567 -26.519 -3.142 1.00 4.76 H

ATOM 944 CG2 THR A 101 16.257 -26.887 -2.357 1.00 4.70 C

ATOM 945 1HG2 THR A 101 16.263 -27.350 -3.342 1.00 4.70 H

ATOM 946 2HG2 THR A 101 16.177 -25.805 -2.467 1.00 4.70 H

ATOM 947 3HG2 THR A 101 15.402 -27.257 -1.792 1.00 4.70 H

ATOM 948 N HIS A 102 18.851 -28.702 0.797 1.00 4.17 N

ATOM 949 H HIS A 102 18.073 -29.320 0.603 1.00 4.17 H

ATOM 950 CA HIS A 102 20.060 -29.282 1.377 1.00 5.03 C

ATOM 951 HA HIS A 102 20.907 -28.641 1.138 1.00 5.03 H

ATOM 952 C HIS A 102 20.353 -30.672 0.821 1.00 4.60 C

ATOM 953 O HIS A 102 19.457 -31.511 0.728 1.00 4.61 O

ATOM 954 CB HIS A 102 19.918 -29.358 2.901 1.00 0.00 C

ATOM 955 1HB HIS A 102 19.748 -28.353 3.288 1.00 0.00 H

ATOM 956 2HB HIS A 102 19.047 -29.967 3.143 1.00 0.00 H

ATOM 957 CG HIS A 102 21.110 -29.949 3.586 1.00 0.00 C

ATOM 958 ND1 HIS A 102 21.293 -31.311 3.748 1.00 0.00 N

ATOM 959 HD1 HIS A 102 20.640 -32.024 3.456 1.00 0.00 H

ATOM 960 CD2 HIS A 102 22.204 -29.380 4.150 1.00 0.00 C

ATOM 961 HD2 HIS A 102 22.399 -28.319 4.218 1.00 0.00 H

ATOM 962 CE1 HIS A 102 22.447 -31.534 4.353 1.00 0.00 C

ATOM 963 HE1 HIS A 102 22.847 -32.507 4.605 1.00 0.00 H

ATOM 964 NE2 HIS A 102 23.023 -30.378 4.607 1.00 0.00 N

ATOM 965 HE2 HIS A 102 23.912 -30.248 5.069 1.00 0.00 H

ATOM 966 N GLY A 103 21.612 -30.912 0.467 1.00 4.87 N

ATOM 967 H GLY A 103 22.299 -30.174 0.543 1.00 4.87 H

ATOM 968 CA GLY A 103 22.041 -32.199 -0.051 1.00 5.13 C

ATOM 969 1HA GLY A 103 22.999 -32.468 0.394 1.00 5.13 H

ATOM 970 2HA GLY A 103 21.307 -32.963 0.208 1.00 5.13 H

ATOM 971 C GLY A 103 22.204 -32.157 -1.557 1.00 4.98 C

ATOM 972 O GLY A 103 22.995 -31.371 -2.074 1.00 6.31 O

ATOM 973 N ARG A 104 21.441 -32.992 -2.253 1.00 4.82 N

ATOM 974 H ARG A 104 20.833 -33.629 -1.752 1.00 4.82 H

ATOM 975 CA ARG A 104 21.380 -33.025 -3.714 1.00 5.36 C

ATOM 976 HA ARG A 104 22.072 -32.294 -4.136 1.00 5.36 H

ATOM 977 C ARG A 104 19.961 -32.678 -4.160 1.00 4.76 C

ATOM 978 O ARG A 104 19.593 -32.811 -5.325 1.00 5.73 O

ATOM 979 CB ARG A 104 21.770 -34.433 -4.176 1.00 0.00 C

ATOM 980 1HB ARG A 104 22.635 -34.751 -3.594 1.00 0.00 H

ATOM 981 2HB ARG A 104 20.944 -35.104 -3.944 1.00 0.00 H

ATOM 982 CG ARG A 104 22.111 -34.589 -5.653 1.00 0.00 C

ATOM 983 1HG ARG A 104 21.250 -34.305 -6.256 1.00 0.00 H

ATOM 984 2HG ARG A 104 22.951 -33.942 -5.905 1.00 0.00 H

ATOM 985 CD ARG A 104 22.476 -36.034 -5.967 1.00 0.00 C

ATOM 986 1HD ARG A 104 21.696 -36.687 -5.572 1.00 0.00 H

ATOM 987 2HD ARG A 104 22.518 -36.160 -7.050 1.00 0.00 H

ATOM 988 NE ARG A 104 23.768 -36.410 -5.393 1.00 0.00 N

ATOM 989 HE ARG A 104 24.383 -35.660 -5.114 1.00 0.00 H

ATOM 990 CZ ARG A 104 24.201 -37.653 -5.218 1.00 0.00 C

ATOM 991 NH1 ARG A 104 25.399 -37.842 -4.705 1.00 0.00 N

ATOM 992 1HH1 ARG A 104 25.965 -37.040 -4.472 1.00 0.00 H

ATOM 993 2HH1 ARG A 104 25.736 -38.780 -4.558 1.00 0.00 H

ATOM 994 NH2 ARG A 104 23.480 -38.712 -5.526 1.00 0.00 N

ATOM 995 1HH2 ARG A 104 22.549 -38.589 -5.894 1.00 0.00 H

ATOM 996 2HH2 ARG A 104 23.864 -39.633 -5.382 1.00 0.00 H

ATOM 997 N ALA A 105 19.163 -32.256 -3.185 1.00 5.73 N

ATOM 998 H ALA A 105 19.555 -32.149 -2.261 1.00 5.73 H

ATOM 999 CA ALA A 105 17.744 -31.961 -3.352 1.00 4.82 C

ATOM 1000 HA ALA A 105 17.254 -32.821 -3.810 1.00 4.82 H

ATOM 1001 C ALA A 105 17.493 -30.746 -4.241 1.00 4.82 C

ATOM 1002 O ALA A 105 18.398 -29.948 -4.491 1.00 4.82 O

ATOM 1003 CB ALA A 105 17.137 -31.735 -1.974 1.00 0.00 C

ATOM 1004 1HB ALA A 105 17.385 -32.571 -1.320 1.00 0.00 H

ATOM 1005 2HB ALA A 105 16.054 -31.663 -2.060 1.00 0.00 H

ATOM 1006 3HB ALA A 105 17.530 -30.812 -1.546 1.00 0.00 H

ATOM 1007 N THR A 20 16.259 -30.596 -4.709 1.00 8.01 N

ATOM 1008 H THR A 20 15.539 -31.265 -4.464 1.00 8.01 H

ATOM 1009 CA THR A 20 15.888 -29.463 -5.552 1.00 7.32 C

ATOM 1010 HA THR A 20 16.618 -28.675 -5.380 1.00 7.32 H

ATOM 1011 C THR A 20 14.514 -28.900 -5.197 1.00 6.71 C

ATOM 1012 O THR A 20 13.615 -29.635 -4.790 1.00 7.40 O

ATOM 1013 CB THR A 20 15.939 -29.829 -7.051 1.00 8.21 C

ATOM 1014 HB THR A 20 16.895 -30.308 -7.263 1.00 8.21 H

ATOM 1015 OG1 THR A 20 15.837 -28.635 -7.838 1.00 8.28 O

ATOM 1016 HG1 THR A 20 15.804 -28.886 -8.764 1.00 8.28 H

ATOM 1017 CG2 THR A 20 14.815 -30.776 -7.454 1.00 10.11 C

ATOM 1018 1HG2 THR A 20 14.961 -31.091 -8.487 1.00 10.11 H

ATOM 1019 2HG2 THR A 20 13.856 -30.265 -7.370 1.00 10.11 H

ATOM 1020 3HG2 THR A 20 14.818 -31.654 -6.809 1.00 10.11 H

ATOM 1021 N SER A 21 14.349 -27.588 -5.334 1.00 5.95 N

ATOM 1022 H SER A 21 15.129 -27.009 -5.611 1.00 5.95 H

ATOM 1023 CA SER A 21 13.055 -26.950 -5.111 1.00 6.66 C

ATOM 1024 HA SER A 21 12.279 -27.697 -5.271 1.00 6.66 H

ATOM 1025 C SER A 21 12.799 -25.790 -6.069 1.00 5.82 C

ATOM 1026 O SER A 21 13.712 -25.029 -6.393 1.00 7.46 O

ATOM 1027 CB SER A 21 12.940 -26.437 -3.674 1.00 0.00 C

ATOM 1028 1HB SER A 21 13.752 -25.737 -3.474 1.00 0.00 H

ATOM 1029 2HB SER A 21 11.989 -25.919 -3.553 1.00 0.00 H

ATOM 1030 OG SER A 21 13.001 -27.509 -2.749 1.00 0.00 O

ATOM 1031 HG SER A 21 13.730 -28.084 -2.993 1.00 0.00 H

ATOM 1032 N THR A 22 11.549 -25.666 -6.503 1.00 5.49 N

ATOM 1033 H THR A 22 10.856 -26.342 -6.211 1.00 5.49 H

ATOM 1034 CA THR A 22 11.125 -24.594 -7.401 1.00 6.16 C

ATOM 1035 HA THR A 22 11.921 -23.852 -7.466 1.00 6.16 H

ATOM 1036 C THR A 22 9.855 -23.921 -6.888 1.00 5.28 C

ATOM 1037 O THR A 22 8.783 -24.527 -6.890 1.00 8.78 O

ATOM 1038 CB THR A 22 10.843 -25.127 -8.821 1.00 0.00 C

ATOM 1039 HB THR A 22 9.974 -25.784 -8.792 1.00 0.00 H

ATOM 1040 OG1 THR A 22 11.975 -25.873 -9.284 1.00 0.00 O

ATOM 1041 HG1 THR A 22 11.790 -26.186 -10.172 1.00 0.00 H

ATOM 1042 CG2 THR A 22 10.575 -23.988 -9.798 1.00 0.00 C

ATOM 1043 1HG2 THR A 22 11.413 -23.291 -9.792 1.00 0.00 H

ATOM 1044 2HG2 THR A 22 9.664 -23.463 -9.511 1.00 0.00 H

ATOM 1045 3HG2 THR A 22 10.452 -24.395 -10.802 1.00 0.00 H

ATOM 1046 N ALA A 23 9.971 -22.677 -6.436 1.00 4.67 N

ATOM 1047 H ALA A 23 10.880 -22.235 -6.412 1.00 4.67 H

ATOM 1048 CA ALA A 23 8.824 -21.913 -5.953 1.00 4.90 C

ATOM 1049 HA ALA A 23 7.956 -22.569 -5.892 1.00 4.90 H

ATOM 1050 C ALA A 23 8.494 -20.756 -6.894 1.00 4.49 C

ATOM 1051 O ALA A 23 9.397 -20.101 -7.415 1.00 6.75 O

ATOM 1052 CB ALA A 23 9.112 -21.375 -4.558 1.00 5.85 C

ATOM 1053 1HB ALA A 23 9.980 -20.716 -4.591 1.00 5.85 H

ATOM 1054 2HB ALA A 23 9.312 -22.205 -3.881 1.00 5.85 H

ATOM 1055 3HB ALA A 23 8.248 -20.816 -4.198 1.00 5.85 H

ATOM 1056 N THR A 24 7.208 -20.490 -7.099 1.00 4.38 N

ATOM 1057 H THR A 24 6.493 -21.079 -6.693 1.00 4.38 H

ATOM 1058 CA THR A 24 6.782 -19.341 -7.894 1.00 4.55 C

ATOM 1059 HA THR A 24 7.543 -18.572 -7.783 1.00 4.55 H

ATOM 1060 C THR A 24 5.462 -18.753 -7.400 1.00 4.01 C

ATOM 1061 O THR A 24 4.576 -19.482 -6.953 1.00 4.76 O

ATOM 1062 CB THR A 24 6.684 -19.684 -9.396 1.00 0.00 C

ATOM 1063 HB THR A 24 7.603 -20.184 -9.703 1.00 0.00 H

ATOM 1064 OG1 THR A 24 6.541 -18.474 -10.151 1.00 0.00 O

ATOM 1065 HG1 THR A 24 7.399 -18.045 -10.195 1.00 0.00 H

ATOM 1066 CG2 THR A 24 5.503 -20.596 -9.706 1.00 0.00 C

ATOM 1067 1HG2 THR A 24 4.571 -20.063 -9.521 1.00 0.00 H

ATOM 1068 2HG2 THR A 24 5.547 -21.487 -9.080 1.00 0.00 H

ATOM 1069 3HG2 THR A 24 5.543 -20.893 -10.754 1.00 0.00 H

ATOM 1070 N ALA A 25 5.333 -17.432 -7.468 1.00 4.01 N

ATOM 1071 H ALA A 25 6.070 -16.871 -7.869 1.00 4.01 H

ATOM 1072 CA ALA A 25 4.135 -16.751 -6.985 1.00 4.72 C

ATOM 1073 HA ALA A 25 3.299 -17.445 -7.065 1.00 4.72 H

ATOM 1074 C ALA A 25 3.800 -15.515 -7.818 1.00 4.13 C

ATOM 1075 O ALA A 25 4.690 -14.795 -8.273 1.00 5.18 O

ATOM 1076 CB ALA A 25 4.299 -16.368 -5.520 1.00 5.59 C

ATOM 1077 1HB ALA A 25 3.366 -15.947 -5.147 1.00 5.59 H

ATOM 1078 2HB ALA A 25 5.094 -15.630 -5.421 1.00 5.59 H

ATOM 1079 3HB ALA A 25 4.551 -17.252 -4.935 1.00 5.59 H

ATOM 1080 N THR A 26 2.507 -15.291 -8.029 1.00 4.82 N

ATOM 1081 H THR A 26 1.826 -15.909 -7.606 1.00 4.82 H

ATOM 1082 CA THR A 26 2.015 -14.221 -8.893 1.00 5.37 C

ATOM 1083 HA THR A 26 2.842 -13.563 -9.158 1.00 5.37 H

ATOM 1084 C THR A 26 0.922 -13.393 -8.222 1.00 5.36 C

ATOM 1085 O THR A 26 -0.088 -13.940 -7.781 1.00 6.16 O

ATOM 1086 CB THR A 26 1.419 -14.816 -10.186 1.00 6.60 C

ATOM 1087 HB THR A 26 0.583 -15.464 -9.925 1.00 6.60 H

ATOM 1088 OG1 THR A 26 2.421 -15.592 -10.855 1.00 7.43 O

ATOM 1089 HG1 THR A 26 2.010 -16.048 -11.592 1.00 7.43 H

ATOM 1090 CG2 THR A 26 0.921 -13.736 -11.139 1.00 7.34 C

ATOM 1091 1HG2 THR A 26 0.644 -14.194 -12.089 1.00 7.34 H

ATOM 1092 2HG2 THR A 26 1.705 -13.000 -11.311 1.00 7.34 H

ATOM 1093 3HG2 THR A 26 0.047 -13.244 -10.713 1.00 7.34 H

ATOM 1094 N GLY A 27 1.113 -12.079 -8.172 1.00 5.68 N

ATOM 1095 H GLY A 27 1.977 -11.681 -8.514 1.00 5.68 H

ATOM 1096 CA GLY A 27 0.115 -11.181 -7.614 1.00 5.95 C

ATOM 1097 1HA GLY A 27 -0.066 -10.361 -8.309 1.00 5.95 H

ATOM 1098 2HA GLY A 27 -0.820 -11.720 -7.460 1.00 5.95 H

ATOM 1099 C GLY A 27 0.572 -10.603 -6.290 1.00 5.58 C

ATOM 1100 O GLY A 27 1.458 -9.752 -6.253 1.00 6.00 O

ATOM 1101 N GLU A 28 -0.013 -11.085 -5.201 1.00 6.18 N

ATOM 1102 H GLU A 28 -0.773 -11.748 -5.291 1.00 6.18 H

ATOM 1103 CA GLU A 28 0.419 -10.763 -3.843 1.00 6.09 C

ATOM 1104 HA GLU A 28 1.447 -10.400 -3.857 1.00 6.09 H

ATOM 1105 C GLU A 28 0.359 -12.079 -3.068 1.00 6.05 C

ATOM 1106 O GLU A 28 -0.075 -12.153 -1.920 1.00 7.67 O

ATOM 1107 CB GLU A 28 -0.489 -9.693 -3.233 1.00 0.00 C

ATOM 1108 1HB GLU A 28 -0.652 -8.914 -3.978 1.00 0.00 H

ATOM 1109 2HB GLU A 28 -1.452 -10.146 -3.000 1.00 0.00 H

ATOM 1110 CG GLU A 28 0.065 -9.033 -1.980 1.00 0.00 C

ATOM 1111 1HG GLU A 28 0.281 -9.796 -1.232 1.00 0.00 H

ATOM 1112 2HG GLU A 28 0.993 -8.520 -2.233 1.00 0.00 H

ATOM 1113 CD GLU A 28 -0.914 -8.034 -1.394 1.00 0.00 C

ATOM 1114 OE1 GLU A 28 -0.568 -6.836 -1.308 1.00 0.00 O

ATOM 1115 OE2 GLU A 28 -2.039 -8.438 -1.029 1.00 0.00 O

ATOM 1116 N ALA A 30 0.768 -13.137 -3.760 1.00 5.99 N

ATOM 1117 H ALA A 30 1.108 -13.000 -4.701 1.00 5.99 H

ATOM 1118 CA ALA A 30 0.698 -14.505 -3.259 1.00 5.30 C

ATOM 1119 HA ALA A 30 -0.110 -14.577 -2.531 1.00 5.30 H

ATOM 1120 C ALA A 30 1.996 -14.933 -2.577 1.00 5.13 C

ATOM 1121 O ALA A 30 3.025 -14.267 -2.697 1.00 6.92 O

ATOM 1122 CB ALA A 30 0.378 -15.433 -4.424 1.00 5.69 C

ATOM 1123 1HB ALA A 30 1.175 -15.381 -5.166 1.00 5.69 H

ATOM 1124 2HB ALA A 30 -0.562 -15.130 -4.884 1.00 5.69 H

ATOM 1125 3HB ALA A 30 0.284 -16.456 -4.063 1.00 5.69 H

ATOM 1126 N MET A 31 1.943 -16.057 -1.870 1.00 4.72 N

ATOM 1127 H MET A 31 1.073 -16.573 -1.828 1.00 4.72 H

ATOM 1128 CA MET A 31 3.094 -16.603 -1.157 1.00 5.29 C

ATOM 1129 HA MET A 31 3.980 -16.010 -1.382 1.00 5.29 H

ATOM 1130 C MET A 31 3.349 -18.047 -1.582 1.00 4.60 C

ATOM 1131 O MET A 31 2.456 -18.887 -1.486 1.00 4.98 O

ATOM 1132 CB MET A 31 2.824 -16.554 0.350 1.00 6.39 C

ATOM 1133 1HB MET A 31 2.698 -15.512 0.646 1.00 6.39 H

ATOM 1134 2HB MET A 31 1.887 -17.075 0.544 1.00 6.39 H

ATOM 1135 CG MET A 31 3.903 -17.188 1.218 1.00 8.61 C

ATOM 1136 1HG MET A 31 3.555 -17.202 2.251 1.00 8.61 H

ATOM 1137 2HG MET A 31 4.051 -18.219 0.898 1.00 8.61 H

ATOM 1138 SD MET A 31 5.491 -16.332 1.155 1.00 11.04 S

ATOM 1139 CE MET A 31 5.225 -14.975 2.303 1.00 8.94 C

ATOM 1140 1HE MET A 31 6.142 -14.395 2.397 1.00 8.94 H

ATOM 1141 2HE MET A 31 4.947 -15.373 3.279 1.00 8.94 H

ATOM 1142 3HE MET A 31 4.427 -14.332 1.931 1.00 8.94 H

ATOM 1143 N ALA A 32 4.561 -18.353 -2.031 1.00 3.90 N

ATOM 1144 H ALA A 32 5.270 -17.635 -2.106 1.00 3.90 H

ATOM 1145 CA ALA A 32 4.911 -19.720 -2.405 1.00 3.55 C

ATOM 1146 HA ALA A 32 4.177 -20.394 -1.968 1.00 3.55 H

ATOM 1147 C ALA A 32 6.288 -20.112 -1.878 1.00 3.90 C

ATOM 1148 O ALA A 32 7.224 -19.314 -1.910 1.00 5.01 O

ATOM 1149 CB ALA A 32 4.869 -19.888 -3.916 1.00 4.55 C

ATOM 1150 1HB ALA A 32 3.885 -19.604 -4.289 1.00 4.55 H

ATOM 1151 2HB ALA A 32 5.063 -20.929 -4.172 1.00 4.55 H

ATOM 1152 3HB ALA A 32 5.629 -19.256 -4.376 1.00 4.55 H

ATOM 1153 N SER A 33 6.430 -21.343 -1.399 1.00 4.02 N

ATOM 1154 H SER A 33 5.640 -21.973 -1.360 1.00 4.02 H

ATOM 1155 CA SER A 33 7.726 -21.808 -0.914 1.00 3.79 C

ATOM 1156 HA SER A 33 8.498 -21.311 -1.499 1.00 3.79 H

ATOM 1157 C SER A 33 7.931 -23.312 -1.065 1.00 4.33 C

ATOM 1158 O SER A 33 6.982 -24.095 -1.017 1.00 4.84 O

ATOM 1159 CB SER A 33 7.904 -21.407 0.552 1.00 4.55 C

ATOM 1160 1HB SER A 33 8.891 -21.716 0.896 1.00 4.55 H

ATOM 1161 2HB SER A 33 7.822 -20.323 0.637 1.00 4.55 H

ATOM 1162 OG SER A 33 6.908 -22.009 1.361 1.00 5.25 O

ATOM 1163 HG SER A 33 6.049 -21.769 1.005 1.00 5.25 H

ATOM 1164 N GLY A 34 9.187 -23.702 -1.252 1.00 4.96 N

ATOM 1165 H GLY A 34 9.925 -23.012 -1.309 1.00 4.96 H

ATOM 1166 CA GLY A 34 9.546 -25.105 -1.361 1.00 4.50 C

ATOM 1167 1HA GLY A 34 8.728 -25.713 -0.974 1.00 4.50 H

ATOM 1168 2HA GLY A 34 9.707 -25.360 -2.408 1.00 4.50 H

ATOM 1169 C GLY A 34 10.803 -25.427 -0.579 1.00 4.64 C

ATOM 1170 O GLY A 34 11.822 -24.749 -0.716 1.00 4.96 O

ATOM 1171 N THR A 35 10.726 -26.460 0.253 1.00 4.35 N

ATOM 1172 H THR A 35 9.856 -26.967 0.346 1.00 4.35 H

ATOM 1173 CA THR A 35 11.857 -26.896 1.068 1.00 4.02 C

ATOM 1174 HA THR A 35 12.747 -26.344 0.771 1.00 4.02 H

ATOM 1175 C THR A 35 12.094 -28.385 0.840 1.00 4.55 C

ATOM 1176 O THR A 35 11.157 -29.181 0.908 1.00 4.35 O

ATOM 1177 CB THR A 35 11.592 -26.644 2.566 1.00 5.12 C

ATOM 1178 HB THR A 35 10.755 -27.262 2.890 1.00 5.12 H

ATOM 1179 OG1 THR A 35 11.255 -25.265 2.762 1.00 5.51 O

ATOM 1180 HG1 THR A 35 11.027 -25.140 3.686 1.00 5.51 H

ATOM 1181 CG2 THR A 35 12.817 -26.964 3.415 1.00 5.97 C

ATOM 1182 1HG2 THR A 35 13.053 -28.025 3.338 1.00 5.97 H

ATOM 1183 2HG2 THR A 35 12.607 -26.720 4.457 1.00 5.97 H

ATOM 1184 3HG2 THR A 35 13.668 -26.375 3.072 1.00 5.97 H

ATOM 1185 N SER A 36 13.334 -28.766 0.551 1.00 4.63 N

ATOM 1186 H SER A 36 14.078 -28.084 0.512 1.00 4.63 H

ATOM 1187 CA SER A 36 13.658 -30.162 0.270 1.00 4.58 C

ATOM 1188 HA SER A 36 12.881 -30.790 0.705 1.00 4.58 H

ATOM 1189 C SER A 36 14.995 -30.584 0.873 1.00 5.05 C

ATOM 1190 O SER A 36 16.000 -29.883 0.748 1.00 6.22 O

ATOM 1191 CB SER A 36 13.670 -30.393 -1.242 1.00 5.69 C

ATOM 1192 1HB SER A 36 13.840 -31.451 -1.443 1.00 5.69 H

ATOM 1193 2HB SER A 36 12.705 -30.105 -1.658 1.00 5.69 H

ATOM 1194 OG SER A 36 14.690 -29.626 -1.856 1.00 7.12 O

ATOM 1195 HG SER A 36 15.527 -29.854 -1.444 1.00 7.12 H

ATOM 1196 N ASP A 37 14.992 -31.734 1.539 1.00 6.11 N

ATOM 1197 H ASP A 37 14.133 -32.262 1.615 1.00 6.11 H

ATOM 1198 CA ASP A 37 16.184 -32.275 2.187 1.00 7.09 C

ATOM 1199 HA ASP A 37 17.023 -31.602 2.007 1.00 7.09 H

ATOM 1200 C ASP A 37 16.550 -33.656 1.649 1.00 7.13 C

ATOM 1201 O ASP A 37 15.684 -34.507 1.441 1.00 6.93 O

ATOM 1202 CB ASP A 37 15.955 -32.388 3.696 1.00 0.00 C

ATOM 1203 1HB ASP A 37 15.043 -32.958 3.874 1.00 0.00 H

ATOM 1204 2HB ASP A 37 16.793 -32.926 4.140 1.00 0.00 H

ATOM 1205 CG ASP A 37 15.836 -31.037 4.373 1.00 0.00 C

ATOM 1206 OD1 ASP A 37 16.723 -30.183 4.163 1.00 0.00 O

ATOM 1207 OD2 ASP A 37 14.867 -30.833 5.135 1.00 0.00 O

ATOM 1208 N GLY A 38 17.843 -33.877 1.436 1.00 8.52 N

ATOM 1209 H GLY A 38 18.514 -33.137 1.587 1.00 8.52 H

ATOM 1210 CA GLY A 38 18.322 -35.150 0.927 1.00 9.47 C

ATOM 1211 1HA GLY A 38 19.265 -35.410 1.408 1.00 9.47 H

ATOM 1212 2HA GLY A 38 17.590 -35.927 1.144 1.00 9.47 H

ATOM 1213 C GLY A 38 18.538 -35.081 -0.572 1.00 8.75 C

ATOM 1214 O GLY A 38 19.449 -34.404 -1.043 1.00 11.96 O

ATOM 1215 N GLU A 39 17.688 -35.775 -1.319 1.00 9.12 N

ATOM 1216 H GLU A 39 16.972 -36.336 -0.874 1.00 9.12 H

ATOM 1217 CA GLU A 39 17.698 -35.768 -2.780 1.00 9.05 C

ATOM 1218 HA GLU A 39 18.381 -35.002 -3.146 1.00 9.05 H

ATOM 1219 C GLU A 39 16.283 -35.462 -3.272 1.00 6.82 C

ATOM 1220 O GLU A 39 15.914 -35.767 -4.405 1.00 8.59 O

ATOM 1221 CB GLU A 39 18.144 -37.133 -3.312 1.00 0.00 C

ATOM 1222 1HB GLU A 39 17.399 -37.874 -3.022 1.00 0.00 H

ATOM 1223 2HB GLU A 39 18.176 -37.086 -4.400 1.00 0.00 H

ATOM 1224 CG GLU A 39 19.500 -37.605 -2.805 1.00 0.00 C

ATOM 1225 1HG GLU A 39 20.240 -36.824 -2.978 1.00 0.00 H

ATOM 1226 2HG GLU A 39 19.436 -37.790 -1.732 1.00 0.00 H

ATOM 1227 CD GLU A 39 19.957 -38.876 -3.495 1.00 0.00 C

ATOM 1228 OE1 GLU A 39 20.214 -38.833 -4.718 1.00 0.00 O

ATOM 1229 OE2 GLU A 39 20.073 -39.920 -2.820 1.00 0.00 O

ATOM 1230 N ALA A 40 15.479 -34.893 -2.380 1.00 5.73 N

ATOM 1231 H ALA A 40 15.865 -34.619 -1.487 1.00 5.73 H

ATOM 1232 CA ALA A 40 14.043 -34.719 -2.577 1.00 6.38 C

ATOM 1233 HA ALA A 40 13.656 -35.614 -3.064 1.00 6.38 H

ATOM 1234 C ALA A 40 13.659 -33.521 -3.443 1.00 6.39 C

ATOM 1235 O ALA A 40 14.478 -32.642 -3.714 1.00 7.96 O

ATOM 1236 CB ALA A 40 13.383 -34.611 -1.210 1.00 0.00 C

ATOM 1237 1HB ALA A 40 13.635 -33.655 -0.752 1.00 0.00 H

ATOM 1238 2HB ALA A 40 13.732 -35.422 -0.571 1.00 0.00 H

ATOM 1239 3HB ALA A 40 12.303 -34.691 -1.322 1.00 0.00 H

ATOM 1240 N THR A 41 12.399 -33.486 -3.867 1.00 5.91 N

ATOM 1241 H THR A 41 11.763 -34.222 -3.590 1.00 5.91 H

ATOM 1242 CA THR A 41 11.879 -32.432 -4.736 1.00 6.38 C

ATOM 1243 HA THR A 41 12.660 -31.690 -4.899 1.00 6.38 H

ATOM 1244 C THR A 41 10.655 -31.737 -4.144 1.00 5.07 C

ATOM 1245 O THR A 41 9.682 -32.395 -3.777 1.00 6.00 O

ATOM 1246 CB THR A 41 11.476 -33.021 -6.103 1.00 9.04 C

ATOM 1247 HB THR A 41 10.663 -33.733 -5.959 1.00 9.04 H

ATOM 1248 OG1 THR A 41 12.599 -33.705 -6.671 1.00 12.54 O

ATOM 1249 HG1 THR A 41 12.962 -34.293 -6.003 1.00 12.54 H

ATOM 1250 CG2 THR A 41 11.020 -31.938 -7.074 1.00 11.48 C

ATOM 1251 1HG2 THR A 41 11.764 -31.143 -7.119 1.00 11.48 H

ATOM 1252 2HG2 THR A 41 10.067 -31.525 -6.744 1.00 11.48 H

ATOM 1253 3HG2 THR A 41 10.896 -32.372 -8.067 1.00 11.48 H

ATOM 1254 N SER A 42 10.681 -30.410 -4.072 1.00 4.36 N

ATOM 1255 H SER A 42 11.517 -29.910 -4.344 1.00 4.36 H

ATOM 1256 CA SER A 42 9.528 -29.634 -3.620 1.00 4.85 C

ATOM 1257 HA SER A 42 8.666 -30.296 -3.543 1.00 4.85 H

ATOM 1258 C SER A 42 9.181 -28.506 -4.589 1.00 4.00 C

ATOM 1259 O SER A 42 10.037 -27.691 -4.931 1.00 6.02 O

ATOM 1260 CB SER A 42 9.806 -29.034 -2.241 1.00 4.79 C

ATOM 1261 1HB SER A 42 10.701 -28.415 -2.292 1.00 4.79 H

ATOM 1262 2HB SER A 42 8.962 -28.413 -1.941 1.00 4.79 H

ATOM 1263 OG SER A 42 9.996 -30.054 -1.277 1.00 5.43 O

ATOM 1264 HG SER A 42 10.328 -29.654 -0.468 1.00 5.43 H

ATOM 1265 N THR A 43 7.929 -28.439 -5.028 1.00 4.22 N

ATOM 1266 H THR A 43 7.256 -29.141 -4.747 1.00 4.22 H

ATOM 1267 CA THR A 43 7.489 -27.384 -5.939 1.00 4.40 C

ATOM 1268 HA THR A 43 8.262 -26.617 -5.986 1.00 4.40 H

ATOM 1269 C THR A 43 6.196 -26.732 -5.460 1.00 4.25 C

ATOM 1270 O THR A 43 5.290 -27.419 -4.989 1.00 4.58 O

ATOM 1271 CB THR A 43 7.239 -27.915 -7.365 1.00 6.18 C

ATOM 1272 HB THR A 43 6.900 -27.093 -7.995 1.00 6.18 H

ATOM 1273 OG1 THR A 43 6.224 -28.925 -7.325 1.00 7.73 O

ATOM 1274 HG1 THR A 43 5.453 -28.561 -6.884 1.00 7.73 H

ATOM 1275 CG2 THR A 43 8.504 -28.513 -7.970 1.00 7.05 C

ATOM 1276 1HG2 THR A 43 8.328 -28.743 -9.020 1.00 7.05 H

ATOM 1277 2HG2 THR A 43 8.769 -29.429 -7.442 1.00 7.05 H

ATOM 1278 3HG2 THR A 43 9.324 -27.799 -7.891 1.00 7.05 H

ATOM 1279 N ALA A 44 6.100 -25.413 -5.588 1.00 4.22 N

ATOM 1280 H ALA A 44 6.849 -24.888 -6.020 1.00 4.22 H

ATOM 1281 CA ALA A 44 4.935 -24.676 -5.106 1.00 3.91 C

ATOM 1282 HA ALA A 44 4.083 -25.355 -5.079 1.00 3.91 H

ATOM 1283 C ALA A 44 4.570 -23.503 -6.012 1.00 4.09 C

ATOM 1284 O ALA A 44 5.437 -22.736 -6.430 1.00 5.06 O

ATOM 1285 CB ALA A 44 5.193 -24.173 -3.692 1.00 4.40 C

ATOM 1286 1HB ALA A 44 6.058 -23.510 -3.690 1.00 4.40 H

ATOM 1287 2HB ALA A 44 5.383 -25.020 -3.032 1.00 4.40 H

ATOM 1288 3HB ALA A 44 4.320 -23.627 -3.334 1.00 4.40 H

ATOM 1289 N THR A 45 3.282 -23.383 -6.318 1.00 3.81 N

ATOM 1290 H THR A 45 2.626 -24.075 -5.982 1.00 3.81 H

ATOM 1291 CA THR A 45 2.753 -22.299 -7.144 1.00 4.32 C

ATOM 1292 HA THR A 45 3.532 -21.556 -7.313 1.00 4.32 H

ATOM 1293 C THR A 45 1.564 -21.636 -6.456 1.00 3.59 C

ATOM 1294 O THR A 45 0.619 -22.320 -6.065 1.00 5.04 O

ATOM 1295 CB THR A 45 2.269 -22.837 -8.506 1.00 5.80 C

ATOM 1296 HB THR A 45 1.459 -23.548 -8.340 1.00 5.80 H

ATOM 1297 OG1 THR A 45 3.352 -23.511 -9.158 1.00 6.79 O

ATOM 1298 HG1 THR A 45 3.710 -24.162 -8.550 1.00 6.79 H

ATOM 1299 CG2 THR A 45 1.767 -21.718 -9.411 1.00 7.13 C

ATOM 1300 1HG2 THR A 45 1.586 -22.116 -10.410 1.00 7.13 H

ATOM 1301 2HG2 THR A 45 2.510 -20.924 -9.468 1.00 7.13 H

ATOM 1302 3HG2 THR A 45 0.835 -21.314 -9.014 1.00 7.13 H

ATOM 1303 N ALA A 46 1.591 -20.315 -6.315 1.00 3.81 N

ATOM 1304 H ALA A 46 2.391 -19.787 -6.635 1.00 3.81 H

ATOM 1305 CA ALA A 46 0.477 -19.585 -5.716 1.00 4.29 C

ATOM 1306 HA ALA A 46 -0.397 -20.235 -5.702 1.00 4.29 H

ATOM 1307 C ALA A 46 0.129 -18.341 -6.530 1.00 3.95 C

ATOM 1308 O ALA A 46 1.017 -17.634 -7.007 1.00 5.13 O

ATOM 1309 CB ALA A 46 0.812 -19.203 -4.283 1.00 4.76 C

ATOM 1310 1HB ALA A 46 -0.061 -18.745 -3.818 1.00 4.76 H

ATOM 1311 2HB ALA A 46 1.641 -18.495 -4.275 1.00 4.76 H

ATOM 1312 3HB ALA A 46 1.091 -20.093 -3.721 1.00 4.76 H

ATOM 1313 N THR A 47 -1.161 -18.078 -6.713 1.00 4.04 N

ATOM 1314 H THR A 47 -1.862 -18.688 -6.314 1.00 4.04 H

ATOM 1315 CA THR A 47 -1.607 -16.935 -7.508 1.00 4.82 C

ATOM 1316 HA THR A 47 -0.757 -16.275 -7.678 1.00 4.82 H

ATOM 1317 C THR A 47 -2.700 -16.133 -6.810 1.00 5.03 C

ATOM 1318 O THR A 47 -3.508 -16.682 -6.061 1.00 5.88 O

ATOM 1319 CB THR A 47 -2.168 -17.371 -8.877 1.00 5.41 C

ATOM 1320 HB THR A 47 -2.473 -16.485 -9.433 1.00 5.41 H

ATOM 1321 OG1 THR A 47 -3.308 -18.213 -8.673 1.00 6.69 O

ATOM 1322 HG1 THR A 47 -3.005 -19.037 -8.284 1.00 6.69 H

ATOM 1323 CG2 THR A 47 -1.134 -18.137 -9.695 1.00 6.48 C

ATOM 1324 1HG2 THR A 47 -1.519 -18.299 -10.701 1.00 6.48 H

ATOM 1325 2HG2 THR A 47 -0.934 -19.102 -9.228 1.00 6.48 H

ATOM 1326 3HG2 THR A 47 -0.209 -17.564 -9.752 1.00 6.48 H

ATOM 1327 N GLY A 48 -2.727 -14.830 -7.068 1.00 5.74 N

ATOM 1328 H GLY A 48 -2.032 -14.420 -7.678 1.00 5.74 H

ATOM 1329 CA GLY A 48 -3.690 -13.948 -6.433 1.00 6.29 C

ATOM 1330 1HA GLY A 48 -3.849 -13.065 -7.052 1.00 6.29 H

ATOM 1331 2HA GLY A 48 -4.638 -14.471 -6.305 1.00 6.29 H

ATOM 1332 C GLY A 48 -3.173 -13.511 -5.078 1.00 6.04 C

ATOM 1333 O GLY A 48 -2.237 -12.717 -4.996 1.00 7.17 O

ATOM 1334 N HIS A 49 -3.767 -14.048 -4.019 1.00 7.24 N

ATOM 1335 H HIS A 49 -4.549 -14.678 -4.150 1.00 7.24 H

ATOM 1336 CA HIS A 49 -3.331 -13.807 -2.646 1.00 7.44 C

ATOM 1337 HA HIS A 49 -2.390 -13.256 -2.640 1.00 7.44 H

ATOM 1338 C HIS A 49 -3.134 -15.149 -1.944 1.00 7.78 C

ATOM 1339 O HIS A 49 -3.080 -15.231 -0.718 1.00 9.08 O

ATOM 1340 CB HIS A 49 -4.397 -12.995 -1.908 1.00 0.00 C

ATOM 1341 1HB HIS A 49 -5.305 -13.595 -1.856 1.00 0.00 H

ATOM 1342 2HB HIS A 49 -4.053 -12.800 -0.892 1.00 0.00 H

ATOM 1343 CG HIS A 49 -4.725 -11.691 -2.566 1.00 0.00 C

ATOM 1344 ND1 HIS A 49 -4.004 -10.530 -2.394 1.00 0.00 N

ATOM 1345 HD1 HIS A 49 -3.193 -10.410 -1.805 1.00 0.00 H

ATOM 1346 CD2 HIS A 49 -5.719 -11.362 -3.430 1.00 0.00 C

ATOM 1347 HD2 HIS A 49 -6.483 -12.042 -3.776 1.00 0.00 H

ATOM 1348 CE1 HIS A 49 -4.563 -9.583 -3.160 1.00 0.00 C

ATOM 1349 HE1 HIS A 49 -4.203 -8.566 -3.230 1.00 0.00 H

ATOM 1350 NE2 HIS A 49 -5.610 -10.048 -3.814 1.00 0.00 N

ATOM 1351 N ALA A 51 -3.073 -16.210 -2.742 1.00 5.09 N

ATOM 1352 H ALA A 51 -3.089 -16.073 -3.742 1.00 5.09 H

ATOM 1353 CA ALA A 51 -3.033 -17.581 -2.246 1.00 4.61 C

ATOM 1354 HA ALA A 51 -3.801 -17.698 -1.480 1.00 4.61 H

ATOM 1355 C ALA A 51 -1.686 -17.943 -1.626 1.00 4.04 C

ATOM 1356 O ALA A 51 -0.684 -17.262 -1.842 1.00 5.46 O

ATOM 1357 CB ALA A 51 -3.352 -18.527 -3.393 1.00 5.38 C

ATOM 1358 1HB ALA A 51 -3.438 -19.543 -3.013 1.00 5.38 H

ATOM 1359 2HB ALA A 51 -2.557 -18.483 -4.137 1.00 5.38 H

ATOM 1360 3HB ALA A 51 -4.297 -18.237 -3.853 1.00 5.38 H

ATOM 1361 N THR A 52 -1.666 -19.031 -0.863 1.00 4.43 N

ATOM 1362 H THR A 52 -2.522 -19.550 -0.718 1.00 4.43 H

ATOM 1363 CA THR A 52 -0.445 -19.519 -0.226 1.00 4.42 C

ATOM 1364 HA THR A 52 0.394 -18.914 -0.564 1.00 4.42 H

ATOM 1365 C THR A 52 -0.179 -20.977 -0.591 1.00 4.05 C

ATOM 1366 O THR A 52 -1.057 -21.825 -0.446 1.00 4.74 O

ATOM 1367 CB THR A 52 -0.543 -19.393 1.308 1.00 6.38 C

ATOM 1368 HB THR A 52 -1.357 -20.022 1.668 1.00 6.38 H

ATOM 1369 OG1 THR A 52 -0.820 -18.029 1.649 1.00 7.81 O

ATOM 1370 HG1 THR A 52 -1.589 -17.744 1.152 1.00 7.81 H

ATOM 1371 CG2 THR A 52 0.753 -19.811 1.991 1.00 8.46 C

ATOM 1372 1HG2 THR A 52 1.592 -19.267 1.557 1.00 8.46 H

ATOM 1373 2HG2 THR A 52 0.909 -20.882 1.862 1.00 8.46 H

ATOM 1374 3HG2 THR A 52 0.689 -19.586 3.055 1.00 8.46 H

ATOM 1375 N ALA A 53 1.028 -21.286 -1.054 1.00 3.48 N

ATOM 1376 H ALA A 53 1.724 -20.562 -1.169 1.00 3.48 H

ATOM 1377 CA ALA A 53 1.394 -22.658 -1.395 1.00 3.86 C

ATOM 1378 HA ALA A 53 0.630 -23.329 -1.005 1.00 3.86 H

ATOM 1379 C ALA A 53 2.732 -23.046 -0.770 1.00 3.97 C

ATOM 1380 O ALA A 53 3.707 -22.302 -0.870 1.00 4.44 O

ATOM 1381 CB ALA A 53 1.444 -22.819 -2.909 1.00 4.72 C

ATOM 1382 1HB ALA A 53 1.677 -23.852 -3.161 1.00 4.72 H

ATOM 1383 2HB ALA A 53 2.214 -22.166 -3.322 1.00 4.72 H

ATOM 1384 3HB ALA A 53 0.477 -22.555 -3.336 1.00 4.72 H

ATOM 1385 N LYS A 54 2.785 -24.197 -0.105 1.00 3.86 N

ATOM 1386 H LYS A 54 1.956 -24.772 -0.034 1.00 3.86 H

ATOM 1387 CA LYS A 54 4.005 -24.647 0.564 1.00 3.61 C

ATOM 1388 HA LYS A 54 4.855 -24.119 0.132 1.00 3.61 H

ATOM 1389 C LYS A 54 4.256 -26.143 0.410 1.00 3.76 C

ATOM 1390 O LYS A 54 3.419 -26.959 0.791 1.00 4.35 O

ATOM 1391 CB LYS A 54 3.925 -24.303 2.053 1.00 0.00 C

ATOM 1392 1HB LYS A 54 3.701 -23.240 2.152 1.00 0.00 H

ATOM 1393 2HB LYS A 54 3.109 -24.870 2.501 1.00 0.00 H

ATOM 1394 CG LYS A 54 5.207 -24.605 2.817 1.00 0.00 C

ATOM 1395 1HG LYS A 54 5.431 -25.669 2.736 1.00 0.00 H

ATOM 1396 2HG LYS A 54 6.027 -24.039 2.375 1.00 0.00 H

ATOM 1397 CD LYS A 54 5.083 -24.236 4.286 1.00 0.00 C

ATOM 1398 1HD LYS A 54 4.809 -23.184 4.370 1.00 0.00 H

ATOM 1399 2HD LYS A 54 4.304 -24.845 4.744 1.00 0.00 H

ATOM 1400 CE LYS A 54 6.398 -24.471 5.010 1.00 0.00 C

ATOM 1401 1HE LYS A 54 6.687 -25.518 4.893 1.00 0.00 H

ATOM 1402 2HE LYS A 54 7.171 -23.848 4.552 1.00 0.00 H

ATOM 1403 NZ LYS A 54 6.232 -24.127 6.460 1.00 0.00 N

ATOM 1404 1HZ LYS A 54 7.096 -24.282 6.961 1.00 0.00 H

ATOM 1405 2HZ LYS A 54 5.509 -24.699 6.877 1.00 0.00 H

ATOM 1406 3HZ LYS A 54 5.973 -23.155 6.562 1.00 0.00 H

ATOM 1407 N SER A 55 5.419 -26.493 -0.129 1.00 3.50 N

ATOM 1408 H SER A 55 6.066 -25.778 -0.430 1.00 3.50 H

ATOM 1409 CA SER A 55 5.795 -27.891 -0.315 1.00 3.85 C

ATOM 1410 HA SER A 55 4.971 -28.525 0.013 1.00 3.85 H

ATOM 1411 C SER A 55 7.036 -28.266 0.489 1.00 3.48 C

ATOM 1412 O SER A 55 8.019 -27.526 0.516 1.00 5.07 O

ATOM 1413 CB SER A 55 6.042 -28.158 -1.801 1.00 4.32 C

ATOM 1414 1HB SER A 55 6.810 -27.476 -2.166 1.00 4.32 H

ATOM 1415 2HB SER A 55 6.383 -29.185 -1.933 1.00 4.32 H

ATOM 1416 OG SER A 55 4.850 -27.959 -2.538 1.00 5.06 O

ATOM 1417 HG SER A 55 5.075 -27.887 -3.470 1.00 5.06 H

ATOM 1418 N MET A 56 6.975 -29.419 1.147 1.00 3.82 N

ATOM 1419 H MET A 56 6.128 -29.971 1.101 1.00 3.82 H

ATOM 1420 CA MET A 56 8.070 -29.937 1.963 1.00 3.77 C

ATOM 1421 HA MET A 56 8.969 -29.345 1.794 1.00 3.77 H

ATOM 1422 C MET A 56 8.357 -31.390 1.594 1.00 3.75 C

ATOM 1423 O MET A 56 7.443 -32.213 1.561 1.00 4.60 O

ATOM 1424 CB MET A 56 7.684 -29.859 3.443 1.00 0.00 C

ATOM 1425 1HB MET A 56 7.542 -28.812 3.711 1.00 0.00 H

ATOM 1426 2HB MET A 56 6.734 -30.377 3.576 1.00 0.00 H

ATOM 1427 CG MET A 56 8.699 -30.479 4.392 1.00 0.00 C

ATOM 1428 1HG MET A 56 8.301 -30.452 5.407 1.00 0.00 H

ATOM 1429 2HG MET A 56 8.857 -31.520 4.111 1.00 0.00 H

ATOM 1430 SD MET A 56 10.282 -29.617 4.361 1.00 0.00 S

ATOM 1431 CE MET A 56 11.385 -30.846 5.063 1.00 0.00 C

ATOM 1432 1HE MET A 56 11.020 -31.147 6.046 1.00 0.00 H

ATOM 1433 2HE MET A 56 12.383 -30.420 5.162 1.00 0.00 H

ATOM 1434 3HE MET A 56 11.425 -31.716 4.407 1.00 0.00 H

ATOM 1435 N SER A 57 9.616 -31.720 1.329 1.00 3.70 N

ATOM 1436 H SER A 57 10.348 -31.023 1.384 1.00 3.70 H

ATOM 1437 CA SER A 57 9.983 -33.086 0.968 1.00 3.84 C

ATOM 1438 HA SER A 57 9.197 -33.749 1.324 1.00 3.84 H

ATOM 1439 C SER A 57 11.295 -33.538 1.604 1.00 4.67 C

ATOM 1440 O SER A 57 12.212 -32.742 1.807 1.00 4.48 O

ATOM 1441 CB SER A 57 10.056 -33.218 -0.555 1.00 4.96 C

ATOM 1442 1HB SER A 57 10.233 -34.261 -0.818 1.00 4.96 H

ATOM 1443 2HB SER A 57 9.108 -32.901 -0.990 1.00 4.96 H

ATOM 1444 OG SER A 57 11.099 -32.416 -1.079 1.00 5.59 O

ATOM 1445 HG SER A 57 10.770 -31.519 -1.178 1.00 5.59 H

ATOM 1446 N THR A 58 11.372 -34.819 1.949 1.00 4.69 N

ATOM 1447 H THR A 58 10.577 -35.425 1.799 1.00 4.69 H

ATOM 1448 CA THR A 58 12.560 -35.385 2.585 1.00 5.34 C

ATOM 1449 HA THR A 58 13.408 -34.724 2.406 1.00 5.34 H

ATOM 1450 C THR A 58 12.885 -36.770 2.032 1.00 5.71 C

ATOM 1451 O THR A 58 12.005 -37.626 1.948 1.00 5.86 O

ATOM 1452 CB THR A 58 12.348 -35.515 4.107 1.00 6.55 C

ATOM 1453 HB THR A 58 11.581 -36.265 4.300 1.00 6.55 H

ATOM 1454 OG1 THR A 58 11.910 -34.257 4.634 1.00 7.24 O

ATOM 1455 HG1 THR A 58 12.331 -33.557 4.130 1.00 7.24 H

ATOM 1456 CG2 THR A 58 13.632 -35.919 4.823 1.00 7.81 C

ATOM 1457 1HG2 THR A 58 14.434 -35.230 4.559 1.00 7.81 H

ATOM 1458 2HG2 THR A 58 13.912 -36.932 4.534 1.00 7.81 H

ATOM 1459 3HG2 THR A 58 13.470 -35.887 5.900 1.00 7.81 H

ATOM 1460 N GLY A 59 14.143 -36.995 1.668 1.00 6.42 N

ATOM 1461 H GLY A 59 14.825 -36.250 1.728 1.00 6.42 H

ATOM 1462 CA GLY A 59 14.570 -38.281 1.144 1.00 6.74 C

ATOM 1463 1HA GLY A 59 15.530 -38.553 1.583 1.00 6.74 H

ATOM 1464 2HA GLY A 59 13.837 -39.046 1.401 1.00 6.74 H

ATOM 1465 C GLY A 59 14.725 -38.226 -0.362 1.00 6.76 C

ATOM 1466 O GLY A 59 15.607 -37.533 -0.864 1.00 8.12 O

ATOM 1467 N ARG A 60 13.861 -38.932 -1.084 1.00 6.31 N

ATOM 1468 H ARG A 60 13.197 -39.535 -0.614 1.00 6.31 H

ATOM 1469 CA ARG A 60 13.757 -38.832 -2.540 1.00 7.22 C

ATOM 1470 HA ARG A 60 14.363 -38.001 -2.903 1.00 7.22 H

ATOM 1471 C ARG A 60 12.298 -38.559 -2.893 1.00 7.28 C

ATOM 1472 O ARG A 60 11.807 -38.928 -3.958 1.00 10.66 O

ATOM 1473 CB ARG A 60 14.227 -40.132 -3.199 1.00 8.68 C

ATOM 1474 1HB ARG A 60 13.659 -40.963 -2.780 1.00 8.68 H

ATOM 1475 2HB ARG A 60 14.020 -40.077 -4.268 1.00 8.68 H

ATOM 1476 CG ARG A 60 15.713 -40.400 -3.013 1.00 10.30 C

ATOM 1477 1HG ARG A 60 16.275 -39.628 -3.536 1.00 10.30 H

ATOM 1478 2HG ARG A 60 15.959 -40.358 -1.952 1.00 10.30 H

ATOM 1479 CD ARG A 60 16.128 -41.758 -3.556 1.00 12.50 C

ATOM 1480 1HD ARG A 60 15.570 -42.537 -3.036 1.00 12.50 H

ATOM 1481 2HD ARG A 60 15.899 -41.802 -4.622 1.00 12.50 H

ATOM 1482 NE ARG A 60 17.562 -41.962 -3.356 1.00 14.16 N

ATOM 1483 HE ARG A 60 18.082 -41.203 -2.941 1.00 14.16 H

ATOM 1484 CZ ARG A 60 18.238 -43.060 -3.672 1.00 16.01 C

ATOM 1485 NH1 ARG A 60 17.663 -44.118 -4.208 1.00 18.66 N

ATOM 1486 1HH1 ARG A 60 16.674 -44.101 -4.398 1.00 18.66 H

ATOM 1487 2HH1 ARG A 60 18.222 -44.930 -4.422 1.00 18.66 H

ATOM 1488 NH2 ARG A 60 19.533 -43.096 -3.442 1.00 14.46 N

ATOM 1489 1HH2 ARG A 60 19.983 -42.281 -3.052 1.00 14.46 H

ATOM 1490 2HH2 ARG A 60 20.058 -43.927 -3.666 1.00 14.46 H

ATOM 1491 N ALA A 61 11.609 -37.936 -1.944 1.00 6.27 N

ATOM 1492 H ALA A 61 12.089 -37.654 -1.103 1.00 6.27 H

ATOM 1493 CA ALA A 61 10.174 -37.693 -2.024 1.00 5.65 C

ATOM 1494 HA ALA A 61 9.689 -38.569 -2.455 1.00 5.65 H

ATOM 1495 C ALA A 61 9.853 -36.481 -2.893 1.00 5.32 C

ATOM 1496 O ALA A 61 10.717 -35.641 -3.142 1.00 6.32 O

ATOM 1497 CB ALA A 61 9.637 -37.486 -0.616 1.00 0.00 C

ATOM 1498 1HB ALA A 61 10.043 -36.565 -0.199 1.00 0.00 H

ATOM 1499 2HB ALA A 61 9.925 -38.327 0.013 1.00 0.00 H

ATOM 1500 3HB ALA A 61 8.550 -37.424 -0.648 1.00 0.00 H

ATOM 1501 N THR A 62 8.606 -36.384 -3.342 1.00 4.60 N

ATOM 1502 H THR A 62 7.938 -37.109 -3.116 1.00 4.60 H

ATOM 1503 CA THR A 62 8.157 -35.253 -4.150 1.00 4.61 C

ATOM 1504 HA THR A 62 8.960 -34.519 -4.203 1.00 4.61 H

ATOM 1505 C THR A 62 6.928 -34.591 -3.535 1.00 4.46 C

ATOM 1506 O THR A 62 5.924 -35.256 -3.281 1.00 4.81 O

ATOM 1507 CB THR A 62 7.818 -35.698 -5.587 1.00 6.27 C

ATOM 1508 HB THR A 62 6.974 -36.386 -5.560 1.00 6.27 H

ATOM 1509 OG1 THR A 62 8.952 -36.368 -6.150 1.00 8.08 O

ATOM 1510 HG1 THR A 62 9.229 -37.057 -5.540 1.00 8.08 H

ATOM 1511 CG2 THR A 62 7.468 -34.507 -6.472 1.00 7.68 C

ATOM 1512 1HG2 THR A 62 7.341 -34.847 -7.500 1.00 7.68 H

ATOM 1513 2HG2 THR A 62 8.269 -33.769 -6.433 1.00 7.68 H

ATOM 1514 3HG2 THR A 62 6.538 -34.053 -6.129 1.00 7.68 H

ATOM 1515 N ALA A 63 6.994 -33.284 -3.303 1.00 3.88 N

ATOM 1516 H ALA A 63 7.844 -32.778 -3.513 1.00 3.88 H

ATOM 1517 CA ALA A 63 5.858 -32.536 -2.772 1.00 3.86 C

ATOM 1518 HA ALA A 63 5.010 -33.213 -2.692 1.00 3.86 H

ATOM 1519 C ALA A 63 5.467 -31.390 -3.703 1.00 3.67 C

ATOM 1520 O ALA A 63 6.311 -30.585 -4.097 1.00 4.75 O

ATOM 1521 CB ALA A 63 6.173 -32.013 -1.378 1.00 4.25 C

ATOM 1522 1HB ALA A 63 6.355 -32.854 -0.708 1.00 4.25 H

ATOM 1523 2HB ALA A 63 5.328 -31.434 -1.007 1.00 4.25 H

ATOM 1524 3HB ALA A 63 7.060 -31.380 -1.414 1.00 4.25 H

ATOM 1525 N THR A 64 4.192 -31.333 -4.074 1.00 3.90 N

ATOM 1526 H THR A 64 3.538 -32.008 -3.699 1.00 3.90 H

ATOM 1527 CA THR A 64 3.697 -30.377 -5.063 1.00 4.12 C

ATOM 1528 HA THR A 64 4.478 -29.647 -5.273 1.00 4.12 H

ATOM 1529 C THR A 64 2.450 -29.642 -4.580 1.00 3.87 C

ATOM 1530 O THR A 64 1.485 -30.268 -4.146 1.00 4.48 O

ATOM 1531 CB THR A 64 3.349 -31.109 -6.376 1.00 5.25 C

ATOM 1532 HB THR A 64 2.526 -31.798 -6.192 1.00 5.25 H

ATOM 1533 OG1 THR A 64 4.488 -31.857 -6.818 1.00 6.91 O

ATOM 1534 HG1 THR A 64 4.246 -32.337 -7.613 1.00 6.91 H

ATOM 1535 CG2 THR A 64 2.948 -30.135 -7.478 1.00 6.15 C

ATOM 1536 1HG2 THR A 64 3.718 -29.372 -7.597 1.00 6.15 H

ATOM 1537 2HG2 THR A 64 2.001 -29.659 -7.223 1.00 6.15 H

ATOM 1538 3HG2 THR A 64 2.832 -30.679 -8.416 1.00 6.15 H

ATOM 1539 N THR A 65 2.448 -28.316 -4.658 1.00 3.77 N

ATOM 1540 H THR A 65 3.273 -27.827 -4.978 1.00 3.77 H

ATOM 1541 CA THR A 65 1.282 -27.522 -4.277 1.00 3.65 C

ATOM 1542 HA THR A 65 0.423 -28.179 -4.142 1.00 3.65 H

ATOM 1543 C THR A 65 0.922 -26.471 -5.323 1.00 3.37 C

ATOM 1544 O THR A 65 1.799 -25.825 -5.895 1.00 4.35 O

ATOM 1545 CB THR A 65 1.517 -26.753 -2.964 1.00 3.95 C

ATOM 1546 HB THR A 65 0.683 -26.073 -2.791 1.00 3.95 H

ATOM 1547 OG1 THR A 65 2.734 -26.003 -3.060 1.00 5.08 O

ATOM 1548 HG1 THR A 65 3.469 -26.614 -2.971 1.00 5.08 H

ATOM 1549 CG2 THR A 65 1.641 -27.692 -1.781 1.00 4.33 C

ATOM 1550 1HG2 THR A 65 1.700 -27.103 -0.867 1.00 4.33 H

ATOM 1551 2HG2 THR A 65 2.539 -28.299 -1.880 1.00 4.33 H

ATOM 1552 3HG2 THR A 65 0.769 -28.340 -1.729 1.00 4.33 H

ATOM 1553 N THR A 66 -0.374 -26.290 -5.553 1.00 3.37 N

ATOM 1554 H THR A 66 -1.049 -26.917 -5.137 1.00 3.37 H

ATOM 1555 CA THR A 66 -0.878 -25.196 -6.379 1.00 3.75 C

ATOM 1556 HA THR A 66 -0.082 -24.474 -6.555 1.00 3.75 H

ATOM 1557 C THR A 66 -2.027 -24.518 -5.639 1.00 3.54 C

ATOM 1558 O THR A 66 -2.917 -25.203 -5.143 1.00 5.79 O

ATOM 1559 CB THR A 66 -1.385 -25.715 -7.741 1.00 5.11 C

ATOM 1560 HB THR A 66 -2.232 -26.381 -7.579 1.00 5.11 H

ATOM 1561 OG1 THR A 66 -0.334 -26.445 -8.385 1.00 5.90 O

ATOM 1562 HG1 THR A 66 0.002 -27.099 -7.767 1.00 5.90 H

ATOM 1563 CG2 THR A 66 -1.811 -24.571 -8.652 1.00 6.45 C

ATOM 1564 1HG2 THR A 66 -2.687 -24.073 -8.235 1.00 6.45 H

ATOM 1565 2HG2 THR A 66 -2.061 -24.967 -9.636 1.00 6.45 H

ATOM 1566 3HG2 THR A 66 -0.996 -23.853 -8.750 1.00 6.45 H

ATOM 1567 N ALA A 67 -2.032 -23.194 -5.537 1.00 3.83 N

ATOM 1568 H ALA A 67 -1.277 -22.646 -5.927 1.00 3.83 H

ATOM 1569 CA ALA A 67 -3.131 -22.498 -4.873 1.00 4.10 C

ATOM 1570 HA ALA A 67 -3.989 -23.167 -4.855 1.00 4.10 H

ATOM 1571 C ALA A 67 -3.543 -21.231 -5.618 1.00 3.90 C

ATOM 1572 O ALA A 67 -2.695 -20.509 -6.142 1.00 4.80 O

ATOM 1573 CB ALA A 67 -2.756 -22.173 -3.434 1.00 5.06 C

ATOM 1574 1HB ALA A 67 -2.438 -23.081 -2.924 1.00 5.06 H

ATOM 1575 2HB ALA A 67 -3.623 -21.761 -2.918 1.00 5.06 H

ATOM 1576 3HB ALA A 67 -1.944 -21.447 -3.420 1.00 5.06 H

ATOM 1577 N THR A 68 -4.843 -20.961 -5.678 1.00 4.51 N

ATOM 1578 H THR A 68 -5.508 -21.599 -5.262 1.00 4.51 H

ATOM 1579 CA THR A 68 -5.357 -19.774 -6.358 1.00 5.31 C

ATOM 1580 HA THR A 68 -4.530 -19.090 -6.549 1.00 5.31 H

ATOM 1581 C THR A 68 -6.388 -19.052 -5.493 1.00 5.30 C

ATOM 1582 O THR A 68 -6.971 -19.645 -4.586 1.00 5.92 O

ATOM 1583 CB THR A 68 -6.019 -20.132 -7.706 1.00 5.98 C

ATOM 1584 HB THR A 68 -6.334 -19.217 -8.208 1.00 5.98 H

ATOM 1585 OG1 THR A 68 -7.164 -20.956 -7.466 1.00 6.43 O

ATOM 1586 HG1 THR A 68 -7.794 -20.460 -6.937 1.00 6.43 H

ATOM 1587 CG2 THR A 68 -5.092 -20.920 -8.623 1.00 7.59 C

ATOM 1588 1HG2 THR A 68 -4.133 -20.411 -8.715 1.00 7.59 H

ATOM 1589 2HG2 THR A 68 -5.549 -21.006 -9.609 1.00 7.59 H

ATOM 1590 3HG2 THR A 68 -4.933 -21.919 -8.217 1.00 7.59 H

ATOM 1591 N GLY A 69 -6.624 -17.771 -5.760 1.00 5.31 N

ATOM 1592 H GLY A 69 -6.128 -17.302 -6.503 1.00 5.31 H

ATOM 1593 CA GLY A 69 -7.559 -17.009 -4.950 1.00 6.16 C

ATOM 1594 1HA GLY A 69 -7.917 -16.142 -5.505 1.00 6.16 H

ATOM 1595 2HA GLY A 69 -8.408 -17.645 -4.699 1.00 6.16 H

ATOM 1596 C GLY A 69 -6.906 -16.537 -3.665 1.00 5.64 C

ATOM 1597 O GLY A 69 -5.851 -15.908 -3.696 1.00 6.88 O

ATOM 1598 N THR A 70 -7.523 -16.861 -2.534 1.00 6.21 N

ATOM 1599 H THR A 70 -8.405 -17.355 -2.578 1.00 6.21 H

ATOM 1600 CA THR A 70 -6.969 -16.606 -1.205 1.00 6.87 C

ATOM 1601 HA THR A 70 -6.006 -16.102 -1.288 1.00 6.87 H

ATOM 1602 C THR A 70 -6.762 -17.941 -0.489 1.00 6.33 C

ATOM 1603 O THR A 70 -6.575 -18.000 0.725 1.00 6.73 O

ATOM 1604 CB THR A 70 -7.919 -15.742 -0.348 1.00 8.01 C

ATOM 1605 HB THR A 70 -7.509 -15.639 0.657 1.00 8.01 H

ATOM 1606 OG1 THR A 70 -9.202 -16.375 -0.275 1.00 9.95 O

ATOM 1607 HG1 THR A 70 -9.508 -16.539 -1.169 1.00 9.95 H

ATOM 1608 CG2 THR A 70 -8.127 -14.356 -0.943 1.00 9.91 C

ATOM 1609 1HG2 THR A 70 -8.497 -14.438 -1.965 1.00 9.91 H

ATOM 1610 2HG2 THR A 70 -7.185 -13.809 -0.936 1.00 9.91 H

ATOM 1611 3HG2 THR A 70 -8.855 -13.813 -0.341 1.00 9.91 H

ATOM 1612 N ALA A 71 -6.821 -19.019 -1.263 1.00 5.67 N

ATOM 1613 H ALA A 71 -6.943 -18.898 -2.258 1.00 5.67 H

ATOM 1614 CA ALA A 71 -6.737 -20.385 -0.758 1.00 5.03 C

ATOM 1615 HA ALA A 71 -7.370 -20.476 0.125 1.00 5.03 H

ATOM 1616 C ALA A 71 -5.318 -20.793 -0.368 1.00 4.55 C

ATOM 1617 O ALA A 71 -4.348 -20.140 -0.754 1.00 6.17 O

ATOM 1618 CB ALA A 71 -7.271 -21.314 -1.833 1.00 5.98 C

ATOM 1619 1HB ALA A 71 -8.237 -20.947 -2.179 1.00 5.98 H

ATOM 1620 2HB ALA A 71 -7.400 -22.317 -1.431 1.00 5.98 H

ATOM 1621 3HB ALA A 71 -6.572 -21.347 -2.668 1.00 5.98 H

ATOM 1622 N MET A 72 -5.189 -21.879 0.388 1.00 5.02 N

ATOM 1623 H MET A 72 -6.016 -22.397 0.656 1.00 5.02 H

ATOM 1624 CA MET A 72 -3.887 -22.373 0.825 1.00 5.27 C

ATOM 1625 HA MET A 72 -3.123 -21.806 0.297 1.00 5.27 H

ATOM 1626 C MET A 72 -3.673 -23.851 0.506 1.00 4.56 C

ATOM 1627 O MET A 72 -4.518 -24.688 0.813 1.00 5.78 O

ATOM 1628 CB MET A 72 -3.702 -22.129 2.325 1.00 0.00 C

ATOM 1629 1HB MET A 72 -3.824 -21.062 2.513 1.00 0.00 H

ATOM 1630 2HB MET A 72 -4.476 -22.667 2.873 1.00 0.00 H

ATOM 1631 CG MET A 72 -2.338 -22.561 2.848 1.00 0.00 C

ATOM 1632 1HG MET A 72 -2.278 -23.650 2.836 1.00 0.00 H

ATOM 1633 2HG MET A 72 -1.569 -22.169 2.183 1.00 0.00 H

ATOM 1634 SD MET A 72 -1.998 -21.963 4.516 1.00 0.00 S

ATOM 1635 CE MET A 72 -3.040 -23.004 5.540 1.00 0.00 C

ATOM 1636 1HE MET A 72 -2.784 -24.050 5.375 1.00 0.00 H

ATOM 1637 2HE MET A 72 -4.086 -22.838 5.285 1.00 0.00 H

ATOM 1638 3HE MET A 72 -2.881 -22.754 6.589 1.00 0.00 H

ATOM 1639 N ALA A 73 -2.537 -24.173 -0.101 1.00 4.40 N

ATOM 1640 H ALA A 73 -1.874 -23.444 -0.332 1.00 4.40 H

ATOM 1641 CA ALA A 73 -2.187 -25.548 -0.446 1.00 4.40 C

ATOM 1642 HA ALA A 73 -2.986 -26.212 -0.121 1.00 4.40 H

ATOM 1643 C ALA A 73 -0.891 -25.961 0.249 1.00 4.18 C

ATOM 1644 O ALA A 73 0.125 -25.280 0.112 1.00 4.82 O

ATOM 1645 CB ALA A 73 -2.033 -25.674 -1.955 1.00 5.16 C

ATOM 1646 1HB ALA A 73 -1.347 -24.909 -2.318 1.00 5.16 H

ATOM 1647 2HB ALA A 73 -3.004 -25.548 -2.434 1.00 5.16 H

ATOM 1648 3HB ALA A 73 -1.630 -26.654 -2.207 1.00 5.16 H

ATOM 1649 N THR A 74 -0.915 -27.057 1.001 1.00 4.36 N

ATOM 1650 H THR A 74 -1.779 -27.571 1.112 1.00 4.36 H

ATOM 1651 CA THR A 74 0.273 -27.535 1.707 1.00 4.45 C

ATOM 1652 HA THR A 74 1.138 -26.969 1.364 1.00 4.45 H

ATOM 1653 C THR A 74 0.533 -29.020 1.467 1.00 4.26 C

ATOM 1654 O THR A 74 -0.356 -29.840 1.687 1.00 5.49 O

ATOM 1655 CB THR A 74 0.130 -27.317 3.227 1.00 5.48 C

ATOM 1656 HB THR A 74 -0.656 -27.966 3.612 1.00 5.48 H

ATOM 1657 OG1 THR A 74 -0.228 -25.952 3.478 1.00 7.20 O

ATOM 1658 HG1 THR A 74 -0.314 -25.830 4.426 1.00 7.20 H

ATOM 1659 CG2 THR A 74 1.433 -27.615 3.959 1.00 5.54 C

ATOM 1660 1HG2 THR A 74 2.242 -27.025 3.529 1.00 5.54 H

ATOM 1661 2HG2 THR A 74 1.670 -28.675 3.873 1.00 5.54 H

ATOM 1662 3HG2 THR A 74 1.322 -27.359 5.013 1.00 5.54 H

ATOM 1663 N SER A 75 1.737 -29.382 1.036 1.00 3.54 N

ATOM 1664 H SER A 75 2.451 -28.682 0.883 1.00 3.54 H

ATOM 1665 CA SER A 75 2.077 -30.783 0.798 1.00 3.40 C

ATOM 1666 HA SER A 75 1.277 -31.395 1.208 1.00 3.40 H

ATOM 1667 C SER A 75 3.381 -31.191 1.479 1.00 3.84 C

ATOM 1668 O SER A 75 4.373 -30.465 1.423 1.00 4.30 O

ATOM 1669 CB SER A 75 2.181 -31.078 -0.700 1.00 4.27 C

ATOM 1670 1HB SER A 75 2.929 -30.422 -1.144 1.00 4.27 H

ATOM 1671 2HB SER A 75 2.495 -32.113 -0.837 1.00 4.27 H

ATOM 1672 OG SER A 75 0.937 -30.884 -1.350 1.00 5.04 O

ATOM 1673 HG SER A 75 1.098 -30.768 -2.290 1.00 5.04 H

ATOM 1674 N ASN A 76 3.371 -32.361 2.109 1.00 3.67 N

ATOM 1675 H ASN A 76 2.519 -32.906 2.120 1.00 3.67 H

ATOM 1676 CA ASN A 76 4.532 -32.897 2.812 1.00 3.84 C

ATOM 1677 HA ASN A 76 5.412 -32.303 2.566 1.00 3.84 H

ATOM 1678 C ASN A 76 4.771 -34.345 2.391 1.00 4.05 C

ATOM 1679 O ASN A 76 3.879 -35.179 2.530 1.00 5.18 O

ATOM 1680 CB ASN A 76 4.292 -32.848 4.325 1.00 4.39 C

ATOM 1681 1HB ASN A 76 3.292 -33.223 4.539 1.00 4.39 H

ATOM 1682 2HB ASN A 76 5.020 -33.495 4.813 1.00 4.39 H

ATOM 1683 CG ASN A 76 4.421 -31.453 4.900 1.00 4.83 C

ATOM 1684 OD1 ASN A 76 3.777 -30.506 4.458 1.00 6.01 O

ATOM 1685 ND2 ASN A 76 5.261 -31.322 5.914 1.00 6.14 N

ATOM 1686 1HD2 ASN A 76 5.382 -30.413 6.337 1.00 6.14 H

ATOM 1687 2HD2 ASN A 76 5.763 -32.124 6.265 1.00 6.14 H

ATOM 1688 N ALA A 77 5.956 -34.662 1.881 1.00 3.89 N

ATOM 1689 H ALA A 77 6.666 -33.950 1.771 1.00 3.89 H

ATOM 1690 CA ALA A 77 6.262 -36.021 1.440 1.00 3.82 C

ATOM 1691 HA ALA A 77 5.472 -36.688 1.786 1.00 3.82 H

ATOM 1692 C ALA A 77 7.588 -36.517 2.013 1.00 4.18 C

ATOM 1693 O ALA A 77 8.579 -35.788 2.009 1.00 4.88 O

ATOM 1694 CB ALA A 77 6.289 -36.069 -0.080 1.00 4.50 C

ATOM 1695 1HB ALA A 77 5.376 -35.628 -0.478 1.00 4.50 H

ATOM 1696 2HB ALA A 77 6.356 -37.106 -0.407 1.00 4.50 H

ATOM 1697 3HB ALA A 77 7.149 -35.511 -0.451 1.00 4.50 H

ATOM 1698 N ILE A 78 7.616 -37.740 2.533 1.00 4.79 N

ATOM 1699 H ILE A 78 6.775 -38.301 2.526 1.00 4.79 H

ATOM 1700 CA ILE A 78 8.807 -38.284 3.183 1.00 5.53 C

ATOM 1701 HA ILE A 78 9.656 -37.635 2.970 1.00 5.53 H

ATOM 1702 C ILE A 78 9.134 -39.685 2.668 1.00 6.16 C

ATOM 1703 O ILE A 78 8.275 -40.564 2.658 1.00 9.89 O

ATOM 1704 CB ILE A 78 8.613 -38.349 4.717 1.00 6.75 C

ATOM 1705 HB ILE A 78 7.772 -39.008 4.926 1.00 6.75 H

ATOM 1706 CG1 ILE A 78 8.308 -36.965 5.304 1.00 8.73 C

ATOM 1707 1HG1 ILE A 78 8.887 -36.837 6.218 1.00 8.73 H

ATOM 1708 2HG1 ILE A 78 8.630 -36.198 4.600 1.00 8.73 H

ATOM 1709 CG2 ILE A 78 9.848 -38.926 5.406 1.00 8.69 C

ATOM 1710 1HG2 ILE A 78 9.695 -38.936 6.485 1.00 8.69 H

ATOM 1711 2HG2 ILE A 78 10.722 -38.320 5.171 1.00 8.69 H

ATOM 1712 3HG2 ILE A 78 10.016 -39.949 5.069 1.00 8.69 H

ATOM 1713 CD1 ILE A 78 6.846 -36.731 5.643 1.00 10.77 C

ATOM 1714 1HD1 ILE A 78 6.494 -37.517 6.312 1.00 10.77 H

ATOM 1715 2HD1 ILE A 78 6.250 -36.732 4.731 1.00 10.77 H

ATOM 1716 3HD1 ILE A 78 6.740 -35.765 6.136 1.00 10.77 H

ATOM 1717 N GLY A 79 10.376 -39.908 2.249 1.00 5.90 N

ATOM 1718 H GLY A 79 11.055 -39.158 2.257 1.00 5.90 H

ATOM 1719 CA GLY A 79 10.789 -41.204 1.737 1.00 5.96 C

ATOM 1720 1HA GLY A 79 11.766 -41.465 2.143 1.00 5.96 H

ATOM 1721 2HA GLY A 79 10.070 -41.966 2.037 1.00 5.96 H

ATOM 1722 C GLY A 79 10.883 -41.177 0.225 1.00 5.73 C

ATOM 1723 O GLY A 79 11.615 -40.365 -0.335 1.00 6.97 O

ATOM 1724 N GLU A 80 10.129 -42.042 -0.442 1.00 5.47 N

ATOM 1725 H GLU A 80 9.603 -42.745 0.064 1.00 5.47 H

ATOM 1726 CA GLU A 80 9.965 -42.003 -1.894 1.00 5.26 C

ATOM 1727 HA GLU A 80 10.652 -41.281 -2.336 1.00 5.26 H

ATOM 1728 C GLU A 80 8.534 -41.572 -2.207 1.00 4.99 C

ATOM 1729 O GLU A 80 8.037 -41.729 -3.322 1.00 6.03 O

ATOM 1730 CB GLU A 80 10.236 -43.385 -2.497 1.00 0.00 C

ATOM 1731 1HB GLU A 80 9.634 -44.123 -1.968 1.00 0.00 H

ATOM 1732 2HB GLU A 80 9.927 -43.376 -3.542 1.00 0.00 H

ATOM 1733 CG GLU A 80 11.697 -43.809 -2.442 1.00 0.00 C

ATOM 1734 1HG GLU A 80 11.837 -44.676 -3.088 1.00 0.00 H

ATOM 1735 2HG GLU A 80 12.313 -42.994 -2.822 1.00 0.00 H

ATOM 1736 CD GLU A 80 12.152 -44.161 -1.038 1.00 0.00 C

ATOM 1737 OE1 GLU A 80 13.095 -43.509 -0.540 1.00 0.00 O

ATOM 1738 OE2 GLU A 80 11.585 -45.092 -0.428 1.00 0.00 O

ATOM 1739 N ALA A 81 7.860 -41.049 -1.189 1.00 4.84 N

ATOM 1740 H ALA A 81 8.331 -40.920 -0.305 1.00 4.84 H

ATOM 1741 CA ALA A 81 6.448 -40.698 -1.282 1.00 5.06 C

ATOM 1742 HA ALA A 81 5.929 -41.525 -1.762 1.00 5.06 H

ATOM 1743 C ALA A 81 6.183 -39.449 -2.118 1.00 4.86 C

ATOM 1744 O ALA A 81 7.061 -38.605 -2.298 1.00 5.96 O

ATOM 1745 CB ALA A 81 5.883 -40.534 0.118 1.00 0.00 C

ATOM 1746 1HB ALA A 81 6.225 -39.592 0.547 1.00 0.00 H

ATOM 1747 2HB ALA A 81 6.211 -41.362 0.747 1.00 0.00 H

ATOM 1748 3HB ALA A 81 4.796 -40.542 0.061 1.00 0.00 H

ATOM 1749 N THR A 82 4.959 -39.329 -2.622 1.00 4.82 N

ATOM 1750 H THR A 82 4.285 -40.066 -2.477 1.00 4.82 H

ATOM 1751 CA THR A 82 4.544 -38.153 -3.383 1.00 4.66 C

ATOM 1752 HA THR A 82 5.358 -37.430 -3.387 1.00 4.66 H

ATOM 1753 C THR A 82 3.313 -37.513 -2.749 1.00 4.06 C

ATOM 1754 O THR A 82 2.297 -38.180 -2.563 1.00 5.28 O

ATOM 1755 CB THR A 82 4.205 -38.513 -4.844 1.00 5.99 C

ATOM 1756 HB THR A 82 3.339 -39.174 -4.860 1.00 5.99 H

ATOM 1757 OG1 THR A 82 5.322 -39.187 -5.435 1.00 9.53 O

ATOM 1758 HG1 THR A 82 5.610 -39.880 -4.836 1.00 9.53 H

ATOM 1759 CG2 THR A 82 3.890 -37.270 -5.667 1.00 6.56 C

ATOM 1760 1HG2 THR A 82 3.774 -37.550 -6.714 1.00 6.56 H

ATOM 1761 2HG2 THR A 82 4.701 -36.548 -5.575 1.00 6.56 H

ATOM 1762 3HG2 THR A 82 2.962 -36.819 -5.314 1.00 6.56 H

ATOM 1763 N ALA A 83 3.398 -36.226 -2.431 1.00 3.77 N

ATOM 1764 H ALA A 83 4.259 -35.724 -2.604 1.00 3.77 H

ATOM 1765 CA ALA A 83 2.275 -35.481 -1.870 1.00 3.95 C

ATOM 1766 HA ALA A 83 1.400 -36.128 -1.828 1.00 3.95 H

ATOM 1767 C ALA A 83 1.965 -34.292 -2.775 1.00 4.03 C

ATOM 1768 O ALA A 83 2.834 -33.466 -3.041 1.00 4.55 O

ATOM 1769 CB ALA A 83 2.596 -35.007 -0.460 1.00 4.86 C

ATOM 1770 1HB ALA A 83 2.819 -35.865 0.173 1.00 4.86 H

ATOM 1771 2HB ALA A 83 1.739 -34.472 -0.053 1.00 4.86 H

ATOM 1772 3HB ALA A 83 3.458 -34.339 -0.484 1.00 4.86 H

ATOM 1773 N THR A 84 0.738 -34.204 -3.275 1.00 4.06 N

ATOM 1774 H THR A 84 0.036 -34.877 -2.998 1.00 4.06 H

ATOM 1775 CA THR A 84 0.359 -33.149 -4.213 1.00 4.16 C

ATOM 1776 HA THR A 84 1.167 -32.424 -4.292 1.00 4.16 H

ATOM 1777 C THR A 84 -0.875 -32.469 -3.627 1.00 3.71 C

ATOM 1778 O THR A 84 -1.572 -33.106 -2.848 1.00 5.04 O

ATOM 1779 CB THR A 84 0.111 -33.760 -5.609 1.00 5.52 C

ATOM 1780 HB THR A 84 -0.683 -34.502 -5.543 1.00 5.52 H

ATOM 1781 OG1 THR A 84 1.309 -34.416 -6.044 1.00 6.74 O

ATOM 1782 HG1 THR A 84 1.602 -35.003 -5.341 1.00 6.74 H

ATOM 1783 CG2 THR A 84 -0.279 -32.717 -6.648 1.00 6.62 C

ATOM 1784 1HG2 THR A 84 0.382 -31.854 -6.579 1.00 6.62 H

ATOM 1785 2HG2 THR A 84 -1.308 -32.399 -6.478 1.00 6.62 H

ATOM 1786 3HG2 THR A 84 -0.202 -33.153 -7.644 1.00 6.62 H

ATOM 1787 N THR A 85 -1.190 -31.210 -3.907 1.00 3.92 N

ATOM 1788 H THR A 85 -0.528 -30.599 -4.366 1.00 3.92 H

ATOM 1789 CA THR A 85 -2.503 -30.685 -3.533 1.00 3.64 C

ATOM 1790 HA THR A 85 -3.238 -31.418 -3.859 1.00 3.64 H

ATOM 1791 C THR A 85 -2.794 -29.373 -4.258 1.00 3.55 C

ATOM 1792 O THR A 85 -1.867 -28.633 -4.587 1.00 3.99 O

ATOM 1793 CB THR A 85 -2.660 -30.526 -1.998 1.00 4.71 C

ATOM 1794 HB THR A 85 -2.239 -31.398 -1.499 1.00 4.71 H

ATOM 1795 OG1 THR A 85 -4.044 -30.431 -1.642 1.00 5.62 O

ATOM 1796 HG1 THR A 85 -4.116 -30.498 -0.676 1.00 5.62 H

ATOM 1797 CG2 THR A 85 -1.966 -29.290 -1.472 1.00 4.99 C

ATOM 1798 1HG2 THR A 85 -1.745 -29.422 -0.414 1.00 4.99 H

ATOM 1799 2HG2 THR A 85 -2.625 -28.431 -1.598 1.00 4.99 H

ATOM 1800 3HG2 THR A 85 -1.040 -29.129 -2.021 1.00 4.99 H

ATOM 1801 N THR A 86 -4.062 -29.090 -4.537 1.00 3.62 N

ATOM 1802 H THR A 86 -4.788 -29.741 -4.263 1.00 3.62 H

ATOM 1803 CA THR A 86 -4.453 -27.870 -5.241 1.00 3.84 C

ATOM 1804 HA THR A 86 -3.579 -27.229 -5.314 1.00 3.84 H

ATOM 1805 C THR A 86 -5.559 -27.110 -4.511 1.00 3.78 C

ATOM 1806 O THR A 86 -6.655 -27.627 -4.331 1.00 5.73 O

ATOM 1807 CB THR A 86 -4.922 -28.182 -6.678 1.00 4.70 C

ATOM 1808 HB THR A 86 -5.808 -28.812 -6.641 1.00 4.70 H

ATOM 1809 OG1 THR A 86 -3.886 -28.891 -7.370 1.00 5.87 O

ATOM 1810 HG1 THR A 86 -3.672 -29.681 -6.862 1.00 5.87 H

ATOM 1811 CG2 THR A 86 -5.251 -26.911 -7.453 1.00 4.71 C

ATOM 1812 1HG2 THR A 86 -6.148 -26.450 -7.038 1.00 4.71 H

ATOM 1813 2HG2 THR A 86 -5.431 -27.162 -8.499 1.00 4.71 H

ATOM 1814 3HG2 THR A 86 -4.421 -26.209 -7.388 1.00 4.71 H

ATOM 1815 N ALA A 87 -5.323 -25.885 -4.060 1.00 3.83 N

ATOM 1816 H ALA A 87 -4.420 -25.452 -4.206 1.00 3.83 H

ATOM 1817 CA ALA A 87 -6.366 -25.137 -3.363 1.00 4.33 C

ATOM 1818 HA ALA A 87 -7.195 -25.803 -3.127 1.00 4.33 H

ATOM 1819 C ALA A 87 -6.898 -24.004 -4.239 1.00 4.58 C

ATOM 1820 O ALA A 87 -6.157 -23.435 -5.038 1.00 5.22 O

ATOM 1821 CB ALA A 87 -5.815 -24.594 -2.054 1.00 5.11 C

ATOM 1822 1HB ALA A 87 -6.641 -24.282 -1.416 1.00 5.11 H

ATOM 1823 2HB ALA A 87 -5.160 -23.746 -2.252 1.00 5.11 H

ATOM 1824 3HB ALA A 87 -5.253 -25.376 -1.545 1.00 5.11 H

ATOM 1825 N THR A 88 -8.176 -23.665 -4.116 1.00 4.38 N

ATOM 1826 H THR A 88 -8.807 -24.238 -3.570 1.00 4.38 H

ATOM 1827 CA THR A 88 -8.718 -22.467 -4.754 1.00 5.21 C

ATOM 1828 HA THR A 88 -7.938 -21.708 -4.719 1.00 5.21 H

ATOM 1829 C THR A 88 -9.901 -21.893 -3.979 1.00 5.34 C

ATOM 1830 O THR A 88 -10.476 -22.547 -3.110 1.00 7.10 O

ATOM 1831 CB THR A 88 -9.044 -22.670 -6.252 1.00 6.05 C

ATOM 1832 HB THR A 88 -8.150 -23.033 -6.760 1.00 6.05 H

ATOM 1833 OG1 THR A 88 -9.450 -21.421 -6.825 1.00 7.27 O

ATOM 1834 HG1 THR A 88 -9.555 -21.546 -7.768 1.00 7.27 H

ATOM 1835 CG2 THR A 88 -10.169 -23.637 -6.524 1.00 8.34 C

ATOM 1836 1HG2 THR A 88 -11.111 -23.187 -6.214 1.00 8.34 H

ATOM 1837 2HG2 THR A 88 -9.989 -24.568 -5.989 1.00 8.34 H

ATOM 1838 3HG2 THR A 88 -10.212 -23.846 -7.593 1.00 8.34 H

ATOM 1839 N GLY A 89 -10.222 -20.637 -4.273 1.00 5.76 N

ATOM 1840 H GLY A 89 -9.751 -20.172 -5.036 1.00 5.76 H

ATOM 1841 CA GLY A 89 -11.195 -19.884 -3.501 1.00 6.31 C

ATOM 1842 1HA GLY A 89 -11.632 -19.108 -4.128 1.00 6.31 H

ATOM 1843 2HA GLY A 89 -11.988 -20.555 -3.178 1.00 6.31 H

ATOM 1844 C GLY A 89 -10.563 -19.226 -2.290 1.00 7.98 C

ATOM 1845 O GLY A 89 -9.755 -18.315 -2.457 1.00 11.24 O

ATOM 1846 N ARG A 93 -10.902 -19.673 -1.085 1.00 9.76 N

ATOM 1847 H ARG A 93 -11.589 -20.416 -1.020 1.00 9.76 H

ATOM 1848 CA ARG A 93 -10.311 -19.166 0.154 1.00 9.18 C

ATOM 1849 HA ARG A 93 -9.374 -18.666 -0.090 1.00 9.18 H

ATOM 1850 C ARG A 93 -9.973 -20.270 1.147 1.00 8.26 C

ATOM 1851 O ARG A 93 -9.727 -20.023 2.327 1.00 10.36 O

ATOM 1852 CB ARG A 93 -11.233 -18.144 0.828 1.00 0.00 C

ATOM 1853 1HB ARG A 93 -10.661 -17.646 1.611 1.00 0.00 H

ATOM 1854 2HB ARG A 93 -11.513 -17.393 0.089 1.00 0.00 H

ATOM 1855 CG ARG A 93 -12.503 -18.698 1.459 1.00 0.00 C

ATOM 1856 1HG ARG A 93 -13.102 -19.179 0.687 1.00 0.00 H

ATOM 1857 2HG ARG A 93 -12.244 -19.429 2.224 1.00 0.00 H

ATOM 1858 CD ARG A 93 -13.308 -17.572 2.097 1.00 0.00 C

ATOM 1859 1HD ARG A 93 -12.670 -17.021 2.788 1.00 0.00 H

ATOM 1860 2HD ARG A 93 -13.629 -16.888 1.311 1.00 0.00 H

ATOM 1861 NE ARG A 93 -14.493 -18.063 2.802 1.00 0.00 N

ATOM 1862 HE ARG A 93 -15.313 -18.240 2.240 1.00 0.00 H

ATOM 1863 CZ ARG A 93 -14.591 -18.307 4.102 1.00 0.00 C

ATOM 1864 NH1 ARG A 93 -15.723 -18.775 4.583 1.00 0.00 N

ATOM 1865 1HH1 ARG A 93 -16.477 -19.006 3.953 1.00 0.00 H

ATOM 1866 2HH1 ARG A 93 -15.827 -18.917 5.576 1.00 0.00 H

ATOM 1867 NH2 ARG A 93 -13.597 -18.101 4.943 1.00 0.00 N

ATOM 1868 1HH2 ARG A 93 -12.714 -17.765 4.593 1.00 0.00 H

ATOM 1869 2HH2 ARG A 93 -13.734 -18.291 5.923 1.00 0.00 H

ATOM 1870 N ALA A 95 -10.006 -21.503 0.663 1.00 5.73 N

ATOM 1871 H ALA A 95 -10.173 -21.659 -0.320 1.00 5.73 H

ATOM 1872 CA ALA A 95 -9.890 -22.660 1.539 1.00 6.16 C

ATOM 1873 HA ALA A 95 -10.154 -22.359 2.553 1.00 6.16 H

ATOM 1874 C ALA A 95 -8.505 -23.292 1.576 1.00 6.39 C

ATOM 1875 O ALA A 95 -7.633 -22.974 0.772 1.00 9.87 O

ATOM 1876 CB ALA A 95 -10.894 -23.677 1.086 1.00 7.46 C

ATOM 1877 1HB ALA A 95 -11.010 -24.437 1.859 1.00 7.46 H

ATOM 1878 2HB ALA A 95 -10.545 -24.134 0.160 1.00 7.46 H

ATOM 1879 3HB ALA A 95 -11.851 -23.188 0.909 1.00 7.46 H

ATOM 1880 N THR A 96 -8.312 -24.208 2.516 1.00 5.49 N

ATOM 1881 H THR A 96 -9.077 -24.471 3.124 1.00 5.49 H

ATOM 1882 CA THR A 96 -7.041 -24.894 2.705 1.00 5.70 C

ATOM 1883 HA THR A 96 -6.269 -24.376 2.140 1.00 5.70 H

ATOM 1884 C THR A 96 -7.158 -26.327 2.205 1.00 5.07 C

ATOM 1885 O THR A 96 -8.000 -27.096 2.672 1.00 6.51 O

ATOM 1886 CB THR A 96 -6.634 -24.930 4.191 1.00 8.31 C

ATOM 1887 HB THR A 96 -7.363 -25.515 4.750 1.00 8.31 H

ATOM 1888 OG1 THR A 96 -6.619 -23.591 4.701 1.00 10.93 O

ATOM 1889 HG1 THR A 96 -7.489 -23.208 4.562 1.00 10.93 H

ATOM 1890 CG2 THR A 96 -5.257 -25.553 4.376 1.00 7.83 C

ATOM 1891 1HG2 THR A 96 -4.960 -25.466 5.421 1.00 7.83 H

ATOM 1892 2HG2 THR A 96 -4.530 -25.037 3.749 1.00 7.83 H

ATOM 1893 3HG2 THR A 96 -5.290 -26.607 4.101 1.00 7.83 H

ATOM 1894 N SER A 97 -6.290 -26.682 1.265 1.00 4.56 N

ATOM 1895 H SER A 97 -5.662 -25.993 0.871 1.00 4.56 H

ATOM 1896 CA SER A 97 -6.139 -28.072 0.858 1.00 4.26 C

ATOM 1897 HA SER A 97 -6.903 -28.637 1.382 1.00 4.26 H

ATOM 1898 C SER A 97 -4.762 -28.568 1.285 1.00 4.41 C

ATOM 1899 O SER A 97 -3.797 -27.806 1.261 1.00 5.01 O

ATOM 1900 CB SER A 97 -6.346 -28.242 -0.647 1.00 0.00 C

ATOM 1901 1HB SER A 97 -5.602 -27.656 -1.186 1.00 0.00 H

ATOM 1902 2HB SER A 97 -6.237 -29.294 -0.912 1.00 0.00 H

ATOM 1903 OG SER A 97 -7.644 -27.801 -1.003 1.00 0.00 O

ATOM 1904 HG SER A 97 -7.830 -26.988 -0.524 1.00 0.00 H

ATOM 1905 N SER A 98 -4.650 -29.826 1.697 1.00 4.24 N

ATOM 1906 H SER A 98 -5.458 -30.434 1.713 1.00 4.24 H

ATOM 1907 CA SER A 98 -3.359 -30.340 2.144 1.00 4.02 C

ATOM 1908 HA SER A 98 -2.591 -29.771 1.622 1.00 4.02 H

ATOM 1909 C SER A 98 -3.118 -31.810 1.821 1.00 3.64 C

ATOM 1910 O SER A 98 -4.044 -32.566 1.529 1.00 4.07 O

ATOM 1911 CB SER A 98 -3.172 -30.095 3.643 1.00 5.86 C

ATOM 1912 1HB SER A 98 -2.137 -30.308 3.911 1.00 5.86 H

ATOM 1913 2HB SER A 98 -3.383 -29.049 3.863 1.00 5.86 H

ATOM 1914 OG SER A 98 -4.024 -30.917 4.421 1.00 8.31 O

ATOM 1915 HG SER A 98 -3.863 -30.731 5.349 1.00 8.31 H

ATOM 1916 N SER A 99 -1.851 -32.205 1.858 1.00 3.19 N

ATOM 1917 H SER A 99 -1.120 -31.536 2.061 1.00 3.19 H

ATOM 1918 CA SER A 99 -1.466 -33.584 1.584 1.00 3.31 C

ATOM 1919 HA SER A 99 -2.278 -34.239 1.891 1.00 3.31 H

ATOM 1920 C SER A 99 -0.185 -33.995 2.303 1.00 3.20 C

ATOM 1921 O SER A 99 0.834 -33.324 2.174 1.00 4.21 O

ATOM 1922 CB SER A 99 -1.260 -33.751 0.082 1.00 0.00 C

ATOM 1923 1HB SER A 99 -2.192 -33.527 -0.435 1.00 0.00 H

ATOM 1924 2HB SER A 99 -0.482 -33.066 -0.258 1.00 0.00 H

ATOM 1925 OG SER A 99 -0.867 -35.081 -0.193 1.00 0.00 O

ATOM 1926 HG SER A 99 -1.459 -35.683 0.269 1.00 0.00 H

ATOM 1927 N THR A 100 -0.214 -35.084 3.063 1.00 3.77 N

ATOM 1928 H THR A 100 -1.079 -35.598 3.172 1.00 3.77 H

ATOM 1929 CA THR A 100 0.976 -35.552 3.771 1.00 3.70 C

ATOM 1930 HA THR A 100 1.835 -34.987 3.414 1.00 3.70 H

ATOM 1931 C THR A 100 1.253 -37.035 3.550 1.00 3.56 C

ATOM 1932 O THR A 100 0.437 -37.884 3.894 1.00 4.14 O

ATOM 1933 CB THR A 100 0.846 -35.303 5.285 1.00 4.76 C

ATOM 1934 HB THR A 100 -0.009 -35.858 5.667 1.00 4.76 H

ATOM 1935 OG1 THR A 100 0.625 -33.906 5.514 1.00 5.45 O

ATOM 1936 HG1 THR A 100 -0.085 -33.617 4.937 1.00 5.45 H

ATOM 1937 CG2 THR A 100 2.097 -35.740 6.036 1.00 5.59 C

ATOM 1938 1HG2 THR A 100 2.186 -36.826 6.002 1.00 5.59 H

ATOM 1939 2HG2 THR A 100 2.024 -35.422 7.076 1.00 5.59 H

ATOM 1940 3HG2 THR A 100 2.980 -35.290 5.582 1.00 5.59 H

ATOM 1941 N THR A 101 2.402 -37.387 2.988 1.00 4.00 N

ATOM 1942 H THR A 101 3.077 -36.683 2.716 1.00 4.00 H

ATOM 1943 CA THR A 101 2.709 -38.794 2.745 1.00 4.05 C

ATOM 1944 HA THR A 101 1.937 -39.420 3.191 1.00 4.05 H

ATOM 1945 C THR A 101 4.059 -39.236 3.275 1.00 4.53 C

ATOM 1946 O THR A 101 4.989 -38.439 3.384 1.00 5.04 O

ATOM 1947 CB THR A 101 2.809 -39.115 1.252 1.00 4.38 C

ATOM 1948 HB THR A 101 3.038 -40.171 1.114 1.00 4.38 H

ATOM 1949 OG1 THR A 101 3.815 -38.304 0.633 1.00 4.76 O

ATOM 1950 HG1 THR A 101 3.592 -38.202 -0.297 1.00 4.76 H

ATOM 1951 CG2 THR A 101 1.520 -38.799 0.574 1.00 4.70 C

ATOM 1952 1HG2 THR A 101 1.671 -38.967 -0.488 1.00 4.70 H

ATOM 1953 2HG2 THR A 101 1.264 -37.752 0.729 1.00 4.70 H

ATOM 1954 3HG2 THR A 101 0.734 -39.450 0.956 1.00 4.70 H

ATOM 1955 N HIS A 102 4.172 -40.530 3.549 1.00 4.17 N

ATOM 1956 H HIS A 102 3.363 -41.139 3.525 1.00 4.17 H

ATOM 1957 CA HIS A 102 5.471 -41.099 3.874 1.00 5.03 C

ATOM 1958 HA HIS A 102 6.195 -40.536 3.289 1.00 5.03 H

ATOM 1959 C HIS A 102 5.615 -42.541 3.401 1.00 4.60 C

ATOM 1960 O HIS A 102 4.622 -43.230 3.187 1.00 4.61 O

ATOM 1961 CB HIS A 102 5.822 -40.861 5.348 1.00 0.00 C

ATOM 1962 1HB HIS A 102 6.782 -41.327 5.571 1.00 0.00 H

ATOM 1963 2HB HIS A 102 5.923 -39.786 5.491 1.00 0.00 H

ATOM 1964 CG HIS A 102 4.804 -41.325 6.344 1.00 0.00 C

ATOM 1965 ND1 HIS A 102 3.581 -40.705 6.534 1.00 0.00 N

ATOM 1966 HD1 HIS A 102 3.185 -40.002 5.926 1.00 0.00 H

ATOM 1967 CD2 HIS A 102 4.882 -42.247 7.335 1.00 0.00 C

ATOM 1968 HD2 HIS A 102 5.699 -42.936 7.493 1.00 0.00 H

ATOM 1969 CE1 HIS A 102 3.002 -41.185 7.622 1.00 0.00 C

ATOM 1970 HE1 HIS A 102 2.040 -40.877 8.009 1.00 0.00 H

ATOM 1971 NE2 HIS A 102 3.773 -42.123 8.131 1.00 0.00 N

ATOM 1972 HE2 HIS A 102 3.575 -42.667 8.959 1.00 0.00 H

ATOM 1973 N GLY A 103 6.862 -42.944 3.178 1.00 4.87 N

ATOM 1974 H GLY A 103 7.622 -42.301 3.352 1.00 4.87 H

ATOM 1975 CA GLY A 103 7.192 -44.242 2.614 1.00 5.13 C

ATOM 1976 1HA GLY A 103 8.086 -44.624 3.107 1.00 5.13 H

ATOM 1977 2HA GLY A 103 6.379 -44.941 2.804 1.00 5.13 H

ATOM 1978 C GLY A 103 7.466 -44.194 1.122 1.00 4.98 C

ATOM 1979 O GLY A 103 8.605 -43.975 0.713 1.00 6.31 O

ATOM 1980 N ARG A 104 6.427 -44.391 0.316 1.00 4.82 N

ATOM 1981 H ARG A 104 5.526 -44.582 0.742 1.00 4.82 H

ATOM 1982 CA ARG A 104 6.478 -44.367 -1.145 1.00 5.36 C

ATOM 1983 HA ARG A 104 7.209 -43.627 -1.470 1.00 5.36 H

ATOM 1984 C ARG A 104 5.107 -43.934 -1.660 1.00 4.76 C

ATOM 1985 O ARG A 104 5.044 -43.371 -2.772 1.00 5.73 O

ATOM 1986 OXT ARG A 104 4.105 -44.144 -0.946 1.00 5.73 O

ATOM 1987 CB ARG A 104 6.905 -45.737 -1.689 1.00 0.00 C

ATOM 1988 1HB ARG A 104 7.919 -45.654 -2.079 1.00 0.00 H

ATOM 1989 2HB ARG A 104 6.937 -46.432 -0.850 1.00 0.00 H

ATOM 1990 CG ARG A 104 6.025 -46.364 -2.766 1.00 0.00 C

ATOM 1991 1HG ARG A 104 6.164 -47.443 -2.719 1.00 0.00 H

ATOM 1992 2HG ARG A 104 4.984 -46.145 -2.535 1.00 0.00 H

ATOM 1993 CD ARG A 104 6.285 -45.940 -4.204 1.00 0.00 C

ATOM 1994 1HD ARG A 104 6.225 -44.855 -4.287 1.00 0.00 H

ATOM 1995 2HD ARG A 104 7.285 -46.261 -4.495 1.00 0.00 H

ATOM 1996 NE ARG A 104 5.298 -46.567 -5.084 1.00 0.00 N

ATOM 1997 HE ARG A 104 5.547 -47.455 -5.494 1.00 0.00 H

ATOM 1998 CZ ARG A 104 4.100 -46.073 -5.374 1.00 0.00 C

ATOM 1999 NH1 ARG A 104 3.294 -46.765 -6.150 1.00 0.00 N

ATOM 2000 1HH1 ARG A 104 3.598 -47.659 -6.503 1.00 0.00 H

ATOM 2001 2HH1 ARG A 104 2.382 -46.397 -6.373 1.00 0.00 H

ATOM 2002 NH2 ARG A 104 3.670 -44.916 -4.916 1.00 0.00 N

ATOM 2003 1HH2 ARG A 104 4.273 -44.355 -4.333 1.00 0.00 H

ATOM 2004 2HH2 ARG A 104 2.738 -44.609 -5.147 1.00 0.00 H

TER 2005 ARG A 104

END

REMARK RiAFP-m9 model coordinates

SEQRES 1 A 159 ALA SER ARG ALA GLU ALA ARG GLY GLU ALA MET ALA GLU

SEQRES 2 A 159 GLY HIS SER ARG GLY CYS ALA THR SER HIS ALA ASN ALA

SEQRES 3 A 159 THR GLY HIS ALA ASP ALA ARG SER MET SER GLU GLY ASN

SEQRES 4 A 159 ALA GLU ALA TYR THR GLU ALA LYS GLY THR ALA MET ALA

SEQRES 5 A 159 THR SER GLU ALA SER GLY GLU ALA ARG ALA GLN THR ASN

SEQRES 6 A 159 ALA ASP GLY ARG ALA HIS SER SER SER ARG THR HIS GLY

SEQRES 7 A 159 ARG ALA ASP SER THR ALA SER ALA LYS GLY GLU ALA MET

SEQRES 8 A 159 ALA GLU GLY THR SER ASP GLY ASP ALA LYS SER TYR ALA

SEQRES 9 A 159 SER ALA ASP GLY ASN ALA CYS ALA LYS SER MET SER THR

SEQRES 10 A 159 GLY HIS ALA ASP ALA THR THR ASN ALA HIS GLY THR ALA

SEQRES 11 A 159 MET ALA ASP SER ASN ALA ILE GLY GLU ALA ARG ALA GLU

SEQRES 12 A 159 THR ARG ALA GLU GLY ARG ALA GLU SER SER SER ASP THR

SEQRES 13 A 159 ASP GLY CYS

ATOM 1 N ALA A 1 30.812 -18.601 -10.254 1.00 8.01 N

ATOM 2 1H ALA A 1 31.750 -18.947 -10.407 1.00 8.01 H

ATOM 3 2H ALA A 1 30.159 -19.302 -10.563 1.00 8.01 H

ATOM 4 3H ALA A 1 30.685 -18.424 -9.266 1.00 8.01 H

ATOM 5 CA ALA A 1 30.619 -17.349 -11.043 1.00 7.32 C

ATOM 6 HA ALA A 1 31.251 -16.566 -10.616 1.00 7.32 H

ATOM 7 C ALA A 1 29.175 -16.860 -10.956 1.00 6.71 C

ATOM 8 O ALA A 1 28.236 -17.639 -11.084 1.00 7.40 O

ATOM 9 CB ALA A 1 31.018 -17.552 -12.513 1.00 0.00 C

ATOM 10 1HB ALA A 1 32.044 -17.920 -12.549 1.00 0.00 H

ATOM 11 2HB ALA A 1 30.950 -16.616 -13.068 1.00 0.00 H

ATOM 12 3HB ALA A 1 30.365 -18.278 -12.998 1.00 0.00 H

ATOM 13 N SER A 2 28.987 -15.570 -10.720 1.00 5.95 N

ATOM 14 H SER A 2 29.761 -14.936 -10.606 1.00 5.95 H

ATOM 15 CA SER A 2 27.644 -15.011 -10.592 1.00 6.66 C

ATOM 16 HA SER A 2 26.959 -15.666 -11.121 1.00 6.66 H

ATOM 17 C SER A 2 27.471 -13.602 -11.156 1.00 5.82 C

ATOM 18 O SER A 2 28.443 -12.892 -11.439 1.00 7.46 O

ATOM 19 CB SER A 2 27.263 -15.011 -9.108 1.00 0.00 C

ATOM 20 1HB SER A 2 26.223 -14.682 -8.986 1.00 0.00 H

ATOM 21 2HB SER A 2 27.350 -16.035 -8.724 1.00 0.00 H

ATOM 22 OG SER A 2 28.106 -14.163 -8.343 1.00 0.00 O

ATOM 23 HG SER A 2 27.570 -13.663 -7.725 1.00 0.00 H

ATOM 24 N ARG A 3 26.210 -13.201 -11.323 1.00 5.49 N

ATOM 25 H ARG A 3 25.442 -13.814 -11.085 1.00 5.49 H

ATOM 26 CA ARG A 3 25.878 -11.883 -11.854 1.00 6.16 C

ATOM 27 HA ARG A 3 26.685 -11.179 -11.622 1.00 6.16 H

ATOM 28 C ARG A 3 24.590 -11.326 -11.243 1.00 5.28 C

ATOM 29 O ARG A 3 23.621 -12.071 -11.087 1.00 8.78 O

ATOM 30 CB ARG A 3 25.725 -11.957 -13.375 1.00 0.00 C

ATOM 31 1HB ARG A 3 26.613 -12.427 -13.775 1.00 0.00 H

ATOM 32 2HB ARG A 3 24.859 -12.569 -13.586 1.00 0.00 H

ATOM 33 CG ARG A 3 25.537 -10.603 -14.064 1.00 0.00 C

ATOM 34 1HG ARG A 3 24.681 -10.089 -13.631 1.00 0.00 H

ATOM 35 2HG ARG A 3 26.408 -9.966 -13.916 1.00 0.00 H

ATOM 36 CD ARG A 3 25.282 -10.758 -15.556 1.00 0.00 C

ATOM 37 1HD ARG A 3 24.431 -11.429 -15.682 1.00 0.00 H

ATOM 38 2HD ARG A 3 25.025 -9.783 -15.975 1.00 0.00 H

ATOM 39 NE ARG A 3 26.442 -11.301 -16.267 1.00 0.00 N

ATOM 40 HE ARG A 3 26.497 -12.306 -16.340 1.00 0.00 H

ATOM 41 CZ ARG A 3 27.452 -10.600 -16.771 1.00 0.00 C

ATOM 42 NH1 ARG A 3 28.415 -11.239 -17.406 1.00 0.00 N

ATOM 43 1HH1 ARG A 3 28.394 -12.254 -17.427 1.00 0.00 H

ATOM 44 2HH1 ARG A 3 29.151 -10.708 -17.853 1.00 0.00 H

ATOM 45 NH2 ARG A 3 27.511 -9.283 -16.704 1.00 0.00 N

ATOM 46 1HH2 ARG A 3 26.706 -8.776 -16.353 1.00 0.00 H

ATOM 47 2HH2 ARG A 3 28.306 -8.802 -17.074 1.00 0.00 H

ATOM 48 N ALA A 4 24.582 -10.038 -10.929 1.00 4.67 N

ATOM 49 H ALA A 4 25.417 -9.475 -11.018 1.00 4.67 H

ATOM 50 CA ALA A 4 23.399 -9.339 -10.430 1.00 4.90 C

ATOM 51 HA ALA A 4 22.563 -10.023 -10.319 1.00 4.90 H

ATOM 52 C ALA A 4 23.025 -8.272 -11.455 1.00 4.49 C

ATOM 53 O ALA A 4 23.905 -7.592 -11.986 1.00 6.75 O

ATOM 54 CB ALA A 4 23.694 -8.710 -9.081 1.00 5.85 C

ATOM 55 1HB ALA A 4 23.924 -9.483 -8.345 1.00 5.85 H

ATOM 56 2HB ALA A 4 22.805 -8.165 -8.755 1.00 5.85 H

ATOM 57 3HB ALA A 4 24.541 -8.026 -9.170 1.00 5.85 H

ATOM 58 N GLU A 5 21.732 -8.156 -11.744 1.00 4.38 N

ATOM 59 H GLU A 5 21.044 -8.779 -11.338 1.00 4.38 H

ATOM 60 CA GLU A 5 21.245 -7.138 -12.675 1.00 4.55 C

ATOM 61 HA GLU A 5 22.028 -6.391 -12.812 1.00 4.55 H

ATOM 62 C GLU A 5 20.009 -6.452 -12.079 1.00 4.01 C

ATOM 63 O GLU A 5 19.027 -7.114 -11.735 1.00 4.76 O

ATOM 64 CB GLU A 5 20.912 -7.791 -14.025 1.00 0.00 C

ATOM 65 1HB GLU A 5 21.766 -8.385 -14.332 1.00 0.00 H

ATOM 66 2HB GLU A 5 20.046 -8.436 -13.923 1.00 0.00 H

ATOM 67 CG GLU A 5 20.597 -6.800 -15.130 1.00 0.00 C

ATOM 68 1HG GLU A 5 19.761 -6.164 -14.845 1.00 0.00 H

ATOM 69 2HG GLU A 5 21.487 -6.194 -15.275 1.00 0.00 H

ATOM 70 CD GLU A 5 20.269 -7.492 -16.441 1.00 0.00 C

ATOM 71 OE1 GLU A 5 21.135 -8.239 -16.944 1.00 0.00 O

ATOM 72 OE2 GLU A 5 19.139 -7.317 -16.967 1.00 0.00 O

ATOM 73 N ALA A 6 20.044 -5.125 -11.972 1.00 4.01 N

ATOM 74 H ALA A 6 20.882 -4.618 -12.228 1.00 4.01 H

ATOM 75 CA ALA A 6 18.895 -4.378 -11.468 1.00 4.72 C

ATOM 76 HA ALA A 6 17.982 -4.942 -11.669 1.00 4.72 H

ATOM 77 C ALA A 6 18.770 -3.025 -12.166 1.00 4.13 C

ATOM 78 O ALA A 6 19.754 -2.479 -12.659 1.00 5.18 O

ATOM 79 CB ALA A 6 19.000 -4.186 -9.966 1.00 5.59 C

ATOM 80 1HB ALA A 6 19.050 -5.156 -9.474 1.00 5.59 H

ATOM 81 2HB ALA A 6 18.122 -3.647 -9.616 1.00 5.59 H

ATOM 82 3HB ALA A 6 19.914 -3.652 -9.718 1.00 5.59 H

ATOM 83 N ARG A 7 17.548 -2.501 -12.205 1.00 4.82 N

ATOM 84 H ARG A 7 16.792 -3.004 -11.752 1.00 4.82 H

ATOM 85 CA ARG A 7 17.266 -1.214 -12.822 1.00 5.37 C

ATOM 86 HA ARG A 7 18.129 -0.570 -12.672 1.00 5.37 H

ATOM 87 C ARG A 7 16.059 -0.543 -12.182 1.00 5.36 C

ATOM 88 O ARG A 7 15.108 -1.207 -11.784 1.00 6.16 O

ATOM 89 CB ARG A 7 17.030 -1.381 -14.330 1.00 0.00 C

ATOM 90 1HB ARG A 7 16.921 -0.392 -14.782 1.00 0.00 H

ATOM 91 2HB ARG A 7 17.922 -1.854 -14.747 1.00 0.00 H

ATOM 92 CG ARG A 7 15.823 -2.264 -14.683 1.00 0.00 C

ATOM 93 1HG ARG A 7 15.959 -3.231 -14.228 1.00 0.00 H

ATOM 94 2HG ARG A 7 14.908 -1.827 -14.315 1.00 0.00 H

ATOM 95 CD ARG A 7 15.697 -2.478 -16.185 1.00 0.00 C

ATOM 96 1HD ARG A 7 16.613 -2.968 -16.527 1.00 0.00 H

ATOM 97 2HD ARG A 7 14.847 -3.134 -16.395 1.00 0.00 H

ATOM 98 NE ARG A 7 15.524 -1.224 -16.917 1.00 0.00 N

ATOM 99 HE ARG A 7 16.358 -0.785 -17.266 1.00 0.00 H

ATOM 100 CZ ARG A 7 14.367 -0.598 -17.126 1.00 0.00 C

ATOM 101 NH1 ARG A 7 14.344 0.537 -17.800 1.00 0.00 N

ATOM 102 1HH1 ARG A 7 15.188 0.899 -18.208 1.00 0.00 H

ATOM 103 2HH1 ARG A 7 13.465 1.015 -17.914 1.00 0.00 H

ATOM 104 NH2 ARG A 7 13.230 -1.081 -16.681 1.00 0.00 N

ATOM 105 1HH2 ARG A 7 13.207 -1.976 -16.229 1.00 0.00 H

ATOM 106 2HH2 ARG A 7 12.378 -0.548 -16.829 1.00 0.00 H

ATOM 107 N GLY A 8 16.126 0.779 -12.085 1.00 5.68 N

ATOM 108 H GLY A 8 16.902 1.290 -12.478 1.00 5.68 H

ATOM 109 CA GLY A 8 15.142 1.542 -11.329 1.00 5.95 C

ATOM 110 1HA GLY A 8 15.042 2.541 -11.765 1.00 5.95 H

ATOM 111 2HA GLY A 8 14.188 1.006 -11.331 1.00 5.95 H

ATOM 112 C GLY A 8 15.582 1.705 -9.885 1.00 5.58 C

ATOM 113 O GLY A 8 16.726 2.086 -9.636 1.00 6.00 O

ATOM 114 N GLU A 9 14.681 1.407 -8.961 1.00 6.18 N

ATOM 115 H GLU A 9 13.754 1.099 -9.212 1.00 6.18 H

ATOM 116 CA GLU A 9 15.009 1.397 -7.542 1.00 6.09 C

ATOM 117 HA GLU A 9 15.929 1.967 -7.372 1.00 6.09 H

ATOM 118 C GLU A 9 15.213 -0.041 -7.063 1.00 6.05 C

ATOM 119 O GLU A 9 15.461 -0.309 -5.885 1.00 7.67 O

ATOM 120 CB GLU A 9 13.876 2.027 -6.735 1.00 0.00 C

ATOM 121 1HB GLU A 9 12.953 1.476 -6.940 1.00 0.00 H

ATOM 122 2HB GLU A 9 14.150 1.934 -5.679 1.00 0.00 H

ATOM 123 CG GLU A 9 13.615 3.490 -7.037 1.00 0.00 C

ATOM 124 1HG GLU A 9 13.049 3.908 -6.215 1.00 0.00 H

ATOM 125 2HG GLU A 9 14.560 4.018 -7.078 1.00 0.00 H

ATOM 126 CD GLU A 9 12.869 3.688 -8.346 1.00 0.00 C

ATOM 127 OE1 GLU A 9 11.745 3.165 -8.493 1.00 0.00 O

ATOM 128 OE2 GLU A 9 13.417 4.368 -9.242 1.00 0.00 O

ATOM 129 N ALA A 10 15.089 -0.953 -8.018 1.00 5.99 N

ATOM 130 H ALA A 10 14.901 -0.642 -8.963 1.00 5.99 H

ATOM 131 CA ALA A 10 15.239 -2.381 -7.776 1.00 5.30 C

ATOM 132 HA ALA A 10 14.621 -2.667 -6.921 1.00 5.30 H

ATOM 133 C ALA A 10 16.689 -2.735 -7.436 1.00 5.13 C

ATOM 134 O ALA A 10 17.625 -2.080 -7.883 1.00 6.92 O

ATOM 135 CB ALA A 10 14.787 -3.154 -9.010 1.00 5.69 C

ATOM 136 1HB ALA A 10 13.771 -2.859 -9.267 1.00 5.69 H

ATOM 137 2HB ALA A 10 14.813 -4.223 -8.809 1.00 5.69 H

ATOM 138 3HB ALA A 10 15.458 -2.938 -9.840 1.00 5.69 H

ATOM 139 N MET A 11 16.851 -3.778 -6.622 1.00 4.72 N

ATOM 140 H MET A 11 16.046 -4.315 -6.329 1.00 4.72 H

ATOM 141 CA MET A 11 18.164 -4.203 -6.166 1.00 5.29 C

ATOM 142 HA MET A 11 18.933 -3.691 -6.742 1.00 5.29 H

ATOM 143 C MET A 11 18.327 -5.705 -6.369 1.00 4.60 C

ATOM 144 O MET A 11 17.389 -6.471 -6.154 1.00 4.98 O

ATOM 145 CB MET A 11 18.316 -3.910 -4.670 1.00 6.39 C

ATOM 146 1HB MET A 11 17.502 -4.382 -4.110 1.00 6.39 H

ATOM 147 2HB MET A 11 19.262 -4.346 -4.331 1.00 6.39 H

ATOM 148 CG MET A 11 18.341 -2.440 -4.319 1.00 8.61 C

ATOM 149 1HG MET A 11 17.462 -1.944 -4.728 1.00 8.61 H

ATOM 150 2HG MET A 11 18.315 -2.336 -3.236 1.00 8.61 H

ATOM 151 SD MET A 11 19.812 -1.595 -4.916 1.00 11.04 S

ATOM 152 CE MET A 11 19.118 -0.018 -5.450 1.00 8.94 C

ATOM 153 1HE MET A 11 19.917 0.559 -5.917 1.00 8.94 H

ATOM 154 2HE MET A 11 18.747 0.519 -4.575 1.00 8.94 H

ATOM 155 3HE MET A 11 18.311 -0.212 -6.158 1.00 8.94 H

ATOM 156 N ALA A 12 19.522 -6.135 -6.756 1.00 3.90 N

ATOM 157 H ALA A 12 20.292 -5.490 -6.873 1.00 3.90 H

ATOM 158 CA ALA A 12 19.766 -7.555 -6.990 1.00 3.55 C

ATOM 159 HA ALA A 12 18.984 -8.127 -6.487 1.00 3.55 H

ATOM 160 C ALA A 12 21.110 -7.967 -6.415 1.00 3.90 C

ATOM 161 O ALA A 12 22.032 -7.159 -6.329 1.00 5.01 O

ATOM 162 CB ALA A 12 19.729 -7.852 -8.491 1.00 4.55 C

ATOM 163 1HB ALA A 12 18.739 -7.583 -8.874 1.00 4.55 H

ATOM 164 2HB ALA A 12 19.916 -8.916 -8.671 1.00 4.55 H

ATOM 165 3HB ALA A 12 20.504 -7.265 -8.998 1.00 4.55 H

ATOM 166 N GLU A 13 21.233 -9.228 -6.033 1.00 4.02 N

ATOM 167 H GLU A 13 20.450 -9.864 -6.082 1.00 4.02 H

ATOM 168 CA GLU A 13 22.506 -9.758 -5.572 1.00 3.79 C

ATOM 169 HA GLU A 13 23.297 -9.186 -6.070 1.00 3.79 H

ATOM 170 C GLU A 13 22.699 -11.224 -5.935 1.00 4.33 C

ATOM 171 O GLU A 13 21.749 -12.014 -5.940 1.00 4.84 O

ATOM 172 CB GLU A 13 22.674 -9.583 -4.063 1.00 0.00 C

ATOM 173 1HB GLU A 13 23.710 -9.818 -3.823 1.00 0.00 H

ATOM 174 2HB GLU A 13 22.506 -8.534 -3.815 1.00 0.00 H

ATOM 175 CG GLU A 13 21.815 -10.482 -3.194 1.00 0.00 C

ATOM 176 1HG GLU A 13 20.766 -10.303 -3.412 1.00 0.00 H

ATOM 177 2HG GLU A 13 22.029 -11.530 -3.391 1.00 0.00 H

ATOM 178 CD GLU A 13 22.050 -10.224 -1.732 1.00 0.00 C

ATOM 179 OE1 GLU A 13 23.172 -10.433 -1.221 1.00 0.00 O

ATOM 180 OE2 GLU A 13 21.089 -9.782 -1.071 1.00 0.00 O

ATOM 181 N GLY A 14 23.938 -11.607 -6.230 1.00 4.96 N

ATOM 182 H GLY A 14 24.710 -10.958 -6.209 1.00 4.96 H

ATOM 183 CA GLY A 14 24.244 -12.969 -6.630 1.00 4.50 C

ATOM 184 1HA GLY A 14 23.524 -13.633 -6.145 1.00 4.50 H

ATOM 185 2HA GLY A 14 24.116 -13.085 -7.713 1.00 4.50 H

ATOM 186 C GLY A 14 25.645 -13.390 -6.249 1.00 4.64 C

ATOM 187 O GLY A 14 26.620 -12.725 -6.595 1.00 4.96 O

ATOM 188 N HIS A 15 25.748 -14.521 -5.562 1.00 4.35 N

ATOM 189 H HIS A 15 24.900 -15.013 -5.317 1.00 4.35 H

ATOM 190 CA HIS A 15 27.019 -15.097 -5.145 1.00 4.02 C

ATOM 191 HA HIS A 15 27.823 -14.651 -5.735 1.00 4.02 H

ATOM 192 C HIS A 15 26.993 -16.601 -5.391 1.00 4.55 C

ATOM 193 O HIS A 15 26.096 -17.304 -4.927 1.00 4.35 O

ATOM 194 CB HIS A 15 27.275 -14.830 -3.661 1.00 0.00 C

ATOM 195 1HB HIS A 15 27.525 -13.777 -3.534 1.00 0.00 H

ATOM 196 2HB HIS A 15 26.367 -15.026 -3.093 1.00 0.00 H

ATOM 197 CG HIS A 15 28.358 -15.690 -3.074 1.00 0.00 C

ATOM 198 ND1 HIS A 15 28.148 -16.956 -2.576 1.00 0.00 N

ATOM 199 HD1 HIS A 15 27.271 -17.447 -2.532 1.00 0.00 H

ATOM 200 CD2 HIS A 15 29.683 -15.457 -2.884 1.00 0.00 C

ATOM 201 HD2 HIS A 15 30.203 -14.543 -3.181 1.00 0.00 H

ATOM 202 CE1 HIS A 15 29.319 -17.409 -2.101 1.00 0.00 C

ATOM 203 HE1 HIS A 15 29.458 -18.376 -1.643 1.00 0.00 H

ATOM 204 NE2 HIS A 15 30.283 -16.511 -2.241 1.00 0.00 N

ATOM 205 N SER A 16 27.984 -17.096 -6.125 1.00 4.63 N

ATOM 206 H SER A 16 28.612 -16.480 -6.615 1.00 4.63 H

ATOM 207 CA SER A 16 28.212 -18.523 -6.269 1.00 4.58 C

ATOM 208 HA SER A 16 28.011 -18.988 -5.305 1.00 4.58 H

ATOM 209 C SER A 16 29.670 -18.773 -6.608 1.00 5.05 C

ATOM 210 O SER A 16 30.268 -18.076 -7.429 1.00 6.22 O

ATOM 211 CB SER A 16 27.296 -19.093 -7.346 1.00 5.69 C

ATOM 212 1HB SER A 16 27.476 -20.160 -7.416 1.00 5.69 H

ATOM 213 2HB SER A 16 26.270 -18.904 -7.052 1.00 5.69 H

ATOM 214 OG SER A 16 27.541 -18.470 -8.594 1.00 7.12 O

ATOM 215 HG SER A 16 28.332 -18.858 -8.972 1.00 7.12 H

ATOM 216 N ARG A 17 30.248 -19.783 -5.967 1.00 6.11 N

ATOM 217 H ARG A 17 29.724 -20.309 -5.278 1.00 6.11 H

ATOM 218 CA ARG A 17 31.641 -20.173 -6.184 1.00 7.09 C

ATOM 219 HA ARG A 17 31.876 -20.177 -7.256 1.00 7.09 H

ATOM 220 C ARG A 17 31.836 -21.592 -5.640 1.00 7.13 C

ATOM 221 O ARG A 17 31.053 -22.001 -4.786 1.00 6.93 O

ATOM 222 CB ARG A 17 32.561 -19.180 -5.451 1.00 0.00 C

ATOM 223 1HB ARG A 17 33.594 -19.439 -5.641 1.00 0.00 H

ATOM 224 2HB ARG A 17 32.406 -18.197 -5.876 1.00 0.00 H

ATOM 225 CG ARG A 17 32.360 -19.090 -3.947 1.00 0.00 C

ATOM 226 1HG ARG A 17 31.311 -18.878 -3.745 1.00 0.00 H

ATOM 227 2HG ARG A 17 32.598 -20.053 -3.495 1.00 0.00 H

ATOM 228 CD ARG A 17 33.195 -17.977 -3.311 1.00 0.00 C

ATOM 229 1HD ARG A 17 32.971 -17.025 -3.785 1.00 0.00 H

ATOM 230 2HD ARG A 17 32.911 -17.891 -2.265 1.00 0.00 H

ATOM 231 NE ARG A 17 34.629 -18.235 -3.426 1.00 0.00 N

ATOM 232 HE ARG A 17 35.125 -17.727 -4.145 1.00 0.00 H

ATOM 233 CZ ARG A 17 35.349 -19.017 -2.622 1.00 0.00 C

ATOM 234 NH1 ARG A 17 36.645 -19.127 -2.846 1.00 0.00 N

ATOM 235 1HH1 ARG A 17 37.072 -18.626 -3.613 1.00 0.00 H

ATOM 236 2HH1 ARG A 17 37.196 -19.751 -2.287 1.00 0.00 H

ATOM 237 NH2 ARG A 17 34.820 -19.690 -1.626 1.00 0.00 N

ATOM 238 1HH2 ARG A 17 33.832 -19.609 -1.443 1.00 0.00 H

ATOM 239 2HH2 ARG A 17 35.401 -20.318 -1.093 1.00 0.00 H

ATOM 240 N GLY A 18 32.838 -22.359 -6.062 1.00 8.52 N

ATOM 241 H GLY A 18 33.025 -23.219 -5.569 1.00 8.52 H

ATOM 242 CA GLY A 18 33.670 -22.045 -7.219 1.00 9.47 C

ATOM 243 1HA GLY A 18 33.978 -21.000 -7.177 1.00 9.47 H

ATOM 244 2HA GLY A 18 34.558 -22.678 -7.222 1.00 9.47 H

ATOM 245 C GLY A 18 32.937 -22.296 -8.519 1.00 8.75 C

ATOM 246 O GLY A 18 32.732 -21.351 -9.280 1.00 11.96 O

ATOM 247 N CYS A 19 32.541 -23.541 -8.757 1.00 9.12 N

ATOM 248 H CYS A 19 32.742 -24.274 -8.091 1.00 9.12 H

ATOM 249 CA CYS A 19 31.822 -23.930 -9.970 1.00 9.05 C

ATOM 250 HA CYS A 19 32.264 -23.415 -10.835 1.00 9.05 H

ATOM 251 C CYS A 19 30.343 -23.570 -9.906 1.00 6.82 C

ATOM 252 O CYS A 19 29.657 -23.538 -10.922 1.00 8.59 O

ATOM 253 CB CYS A 19 31.942 -25.439 -10.177 1.00 0.00 C

ATOM 254 1HB CYS A 19 31.356 -25.931 -9.400 1.00 0.00 H

ATOM 255 2HB CYS A 19 31.536 -25.688 -11.158 1.00 0.00 H

ATOM 256 SG CYS A 19 33.633 -26.082 -10.085 1.00 0.00 S

ATOM 257 HG CYS A 19 33.317 -27.382 -10.115 1.00 0.00 H

ATOM 258 N ALA A 20 29.835 -23.347 -8.703 1.00 5.73 N

ATOM 259 H ALA A 20 30.440 -23.399 -7.894 1.00 5.73 H

ATOM 260 CA ALA A 20 28.432 -22.985 -8.534 1.00 6.38 C

ATOM 261 HA ALA A 20 27.799 -23.804 -8.883 1.00 6.38 H

ATOM 262 C ALA A 20 28.109 -21.734 -9.354 1.00 6.39 C

ATOM 263 O ALA A 20 28.938 -20.841 -9.479 1.00 7.96 O

ATOM 264 CB ALA A 20 28.162 -22.740 -7.046 1.00 0.00 C

ATOM 265 1HB ALA A 20 28.321 -23.674 -6.514 1.00 0.00 H

ATOM 266 2HB ALA A 20 27.123 -22.445 -6.927 1.00 0.00 H

ATOM 267 3HB ALA A 20 28.811 -21.956 -6.662 1.00 0.00 H

ATOM 268 N THR A 21 26.915 -21.698 -9.930 1.00 5.91 N

ATOM 269 H THR A 21 26.265 -22.454 -9.755 1.00 5.91 H

ATOM 270 CA THR A 21 26.494 -20.626 -10.828 1.00 6.38 C

ATOM 271 HA THR A 21 27.267 -19.865 -10.861 1.00 6.38 H

ATOM 272 C THR A 21 25.186 -20.013 -10.337 1.00 5.07 C

ATOM 273 O THR A 21 24.234 -20.742 -10.054 1.00 6.00 O

ATOM 274 CB THR A 21 26.266 -21.169 -12.256 1.00 9.04 C

ATOM 275 HB THR A 21 25.446 -21.883 -12.222 1.00 9.04 H

ATOM 276 OG1 THR A 21 27.449 -21.842 -12.692 1.00 12.54 O

ATOM 277 HG1 THR A 21 27.707 -22.431 -11.977 1.00 12.54 H

ATOM 278 CG2 THR A 21 25.938 -20.059 -13.256 1.00 11.48 C

ATOM 279 1HG2 THR A 21 25.669 -20.514 -14.208 1.00 11.48 H

ATOM 280 2HG2 THR A 21 26.823 -19.442 -13.404 1.00 11.48 H

ATOM 281 3HG2 THR A 21 25.111 -19.466 -12.872 1.00 11.48 H

ATOM 282 N SER A 22 25.121 -18.688 -10.225 1.00 4.36 N

ATOM 283 H SER A 22 25.929 -18.130 -10.462 1.00 4.36 H

ATOM 284 CA SER A 22 23.903 -18.008 -9.780 1.00 4.85 C

ATOM 285 HA SER A 22 23.040 -18.643 -9.993 1.00 4.85 H

ATOM 286 C SER A 22 23.697 -16.668 -10.475 1.00 4.00 C

ATOM 287 O SER A 22 24.649 -16.079 -10.986 1.00 6.02 O

ATOM 288 CB SER A 22 23.958 -17.770 -8.267 1.00 4.79 C

ATOM 289 1HB SER A 22 23.014 -17.329 -7.936 1.00 4.79 H

ATOM 290 2HB SER A 22 24.123 -18.725 -7.762 1.00 4.79 H

ATOM 291 OG SER A 22 25.017 -16.889 -7.916 1.00 5.43 O

ATOM 292 HG SER A 22 25.857 -17.340 -8.041 1.00 5.43 H

ATOM 293 N HIS A 23 22.459 -16.179 -10.470 1.00 4.22 N

ATOM 294 H HIS A 23 21.696 -16.726 -10.098 1.00 4.22 H

ATOM 295 CA HIS A 23 22.165 -14.824 -10.934 1.00 4.40 C

ATOM 296 HA HIS A 23 22.951 -14.168 -10.568 1.00 4.40 H

ATOM 297 C HIS A 23 20.845 -14.316 -10.370 1.00 4.25 C

ATOM 298 O HIS A 23 19.993 -15.089 -9.948 1.00 4.58 O

ATOM 299 CB HIS A 23 22.174 -14.716 -12.468 1.00 0.00 C

ATOM 300 1HB HIS A 23 22.142 -13.652 -12.730 1.00 0.00 H

ATOM 301 2HB HIS A 23 23.103 -15.143 -12.861 1.00 0.00 H

ATOM 302 CG HIS A 23 21.032 -15.408 -13.149 1.00 0.00 C

ATOM 303 ND1 HIS A 23 21.173 -16.429 -14.067 1.00 0.00 N

ATOM 304 HD1 HIS A 23 22.041 -16.865 -14.333 1.00 0.00 H

ATOM 305 CD2 HIS A 23 19.695 -15.201 -13.077 1.00 0.00 C

ATOM 306 HD2 HIS A 23 19.214 -14.468 -12.447 1.00 0.00 H

ATOM 307 CE1 HIS A 23 19.956 -16.751 -14.514 1.00 0.00 C

ATOM 308 HE1 HIS A 23 19.756 -17.494 -15.286 1.00 0.00 H

ATOM 309 NE2 HIS A 23 19.018 -16.053 -13.905 1.00 0.00 N

ATOM 310 N ALA A 24 20.705 -12.993 -10.394 1.00 4.22 N

ATOM 311 H ALA A 24 21.433 -12.412 -10.785 1.00 4.22 H

ATOM 312 CA ALA A 24 19.516 -12.330 -9.876 1.00 3.91 C

ATOM 313 HA ALA A 24 18.679 -13.019 -9.904 1.00 3.91 H

ATOM 314 C ALA A 24 19.137 -11.121 -10.723 1.00 4.09 C

ATOM 315 O ALA A 24 19.993 -10.297 -11.047 1.00 5.06 O

ATOM 316 CB ALA A 24 19.742 -11.896 -8.440 1.00 4.40 C

ATOM 317 1HB ALA A 24 19.777 -12.787 -7.806 1.00 4.40 H

ATOM 318 2HB ALA A 24 18.922 -11.263 -8.093 1.00 4.40 H

ATOM 319 3HB ALA A 24 20.687 -11.349 -8.365 1.00 4.40 H

ATOM 320 N ASN A 25 17.866 -11.030 -11.086 1.00 3.81 N

ATOM 321 H ASN A 25 17.210 -11.736 -10.785 1.00 3.81 H

ATOM 322 CA ASN A 25 17.382 -9.961 -11.956 1.00 4.32 C

ATOM 323 HA ASN A 25 18.196 -9.252 -12.101 1.00 4.32 H

ATOM 324 C ASN A 25 16.205 -9.230 -11.319 1.00 3.59 C

ATOM 325 O ASN A 25 15.149 -9.834 -11.129 1.00 5.04 O

ATOM 326 CB ASN A 25 16.980 -10.573 -13.297 1.00 0.00 C

ATOM 327 1HB ASN A 25 16.142 -11.252 -13.151 1.00 0.00 H

ATOM 328 2HB ASN A 25 16.667 -9.776 -13.968 1.00 0.00 H

ATOM 329 CG ASN A 25 18.110 -11.350 -13.941 1.00 0.00 C

ATOM 330 OD1 ASN A 25 19.209 -10.845 -14.139 1.00 0.00 O

ATOM 331 ND2 ASN A 25 17.840 -12.597 -14.287 1.00 0.00 N

ATOM 332 1HD2 ASN A 25 18.524 -13.127 -14.791 1.00 0.00 H

ATOM 333 2HD2 ASN A 25 16.963 -13.027 -14.025 1.00 0.00 H

ATOM 334 N ALA A 26 16.340 -7.954 -10.989 1.00 3.81 N

ATOM 335 H ALA A 26 17.214 -7.481 -11.161 1.00 3.81 H

ATOM 336 CA ALA A 26 15.280 -7.199 -10.321 1.00 4.29 C

ATOM 337 HA ALA A 26 14.404 -7.848 -10.217 1.00 4.29 H

ATOM 338 C ALA A 26 14.907 -5.982 -11.155 1.00 3.95 C

ATOM 339 O ALA A 26 15.775 -5.254 -11.638 1.00 5.13 O

ATOM 340 CB ALA A 26 15.729 -6.772 -8.935 1.00 4.76 C

ATOM 341 1HB ALA A 26 16.096 -7.642 -8.386 1.00 4.76 H

ATOM 342 2HB ALA A 26 14.866 -6.363 -8.403 1.00 4.76 H

ATOM 343 3HB ALA A 26 16.522 -6.025 -9.027 1.00 4.76 H

ATOM 344 N THR A 27 13.612 -5.734 -11.296 1.00 4.04 N

ATOM 345 H THR A 27 12.926 -6.385 -10.936 1.00 4.04 H

ATOM 346 CA THR A 27 13.155 -4.558 -12.039 1.00 4.82 C

ATOM 347 HA THR A 27 14.020 -3.915 -12.231 1.00 4.82 H

ATOM 348 C THR A 27 12.135 -3.737 -11.258 1.00 5.03 C

ATOM 349 O THR A 27 11.321 -4.282 -10.512 1.00 5.88 O

ATOM 350 CB THR A 27 12.472 -4.895 -13.381 1.00 5.41 C

ATOM 351 HB THR A 27 12.172 -3.965 -13.857 1.00 5.41 H

ATOM 352 OG1 THR A 27 11.318 -5.706 -13.172 1.00 6.69 O

ATOM 353 HG1 THR A 27 11.596 -6.505 -12.736 1.00 6.69 H

ATOM 354 CG2 THR A 27 13.413 -5.628 -14.307 1.00 6.48 C

ATOM 355 1HG2 THR A 27 12.927 -5.865 -15.255 1.00 6.48 H

ATOM 356 2HG2 THR A 27 13.734 -6.559 -13.837 1.00 6.48 H

ATOM 357 3HG2 THR A 27 14.286 -5.011 -14.518 1.00 6.48 H

ATOM 358 N GLY A 28 12.202 -2.429 -11.462 1.00 5.74 N

ATOM 359 H GLY A 28 12.946 -2.038 -12.025 1.00 5.74 H

ATOM 360 CA GLY A 28 11.256 -1.494 -10.855 1.00 6.29 C

ATOM 361 1HA GLY A 28 11.155 -0.615 -11.479 1.00 6.29 H

ATOM 362 2HA GLY A 28 10.284 -1.968 -10.799 1.00 6.29 H

ATOM 363 C GLY A 28 11.677 -1.024 -9.476 1.00 6.04 C

ATOM 364 O GLY A 28 12.371 -0.020 -9.358 1.00 7.17 O

ATOM 365 N HIS A 29 11.231 -1.740 -8.453 1.00 7.24 N

ATOM 366 H HIS A 29 10.625 -2.534 -8.607 1.00 7.24 H

ATOM 367 CA HIS A 29 11.610 -1.526 -7.055 1.00 7.44 C

ATOM 368 HA HIS A 29 12.554 -0.980 -7.034 1.00 7.44 H

ATOM 369 C HIS A 29 11.795 -2.881 -6.350 1.00 7.78 C

ATOM 370 O HIS A 29 11.850 -2.989 -5.121 1.00 9.08 O

ATOM 371 CB HIS A 29 10.496 -0.698 -6.397 1.00 0.00 C

ATOM 372 1HB HIS A 29 10.081 -0.027 -7.148 1.00 0.00 H

ATOM 373 2HB HIS A 29 9.715 -1.360 -6.029 1.00 0.00 H

ATOM 374 CG HIS A 29 10.962 0.140 -5.245 1.00 0.00 C

ATOM 375 ND1 HIS A 29 11.594 -0.370 -4.129 1.00 0.00 N

ATOM 376 HD1 HIS A 29 11.874 -1.326 -3.985 1.00 0.00 H

ATOM 377 CD2 HIS A 29 10.842 1.466 -5.017 1.00 0.00 C

ATOM 378 HD2 HIS A 29 10.391 2.181 -5.709 1.00 0.00 H

ATOM 379 CE1 HIS A 29 11.813 0.650 -3.285 1.00 0.00 C

ATOM 380 HE1 HIS A 29 12.311 0.557 -2.322 1.00 0.00 H

ATOM 381 NE2 HIS A 29 11.345 1.783 -3.769 1.00 0.00 N

ATOM 382 N ALA A 30 11.876 -3.919 -7.161 1.00 5.09 N

ATOM 383 H ALA A 30 11.890 -3.757 -8.154 1.00 5.09 H

ATOM 384 CA ALA A 30 11.832 -5.307 -6.694 1.00 4.61 C

ATOM 385 HA ALA A 30 11.120 -5.427 -5.873 1.00 4.61 H

ATOM 386 C ALA A 30 13.195 -5.733 -6.171 1.00 4.04 C

ATOM 387 O ALA A 30 14.221 -5.111 -6.465 1.00 5.46 O

ATOM 388 CB ALA A 30 11.420 -6.216 -7.843 1.00 5.38 C

ATOM 389 1HB ALA A 30 10.487 -5.871 -8.274 1.00 5.38 H

ATOM 390 2HB ALA A 30 11.261 -7.226 -7.482 1.00 5.38 H

ATOM 391 3HB ALA A 30 12.184 -6.225 -8.614 1.00 5.38 H

ATOM 392 N ASP A 31 13.213 -6.812 -5.402 1.00 4.43 N

ATOM 393 H ASP A 31 12.332 -7.253 -5.155 1.00 4.43 H

ATOM 394 CA ASP A 31 14.450 -7.395 -4.889 1.00 4.42 C

ATOM 395 HA ASP A 31 15.296 -6.817 -5.261 1.00 4.42 H

ATOM 396 C ASP A 31 14.612 -8.842 -5.349 1.00 4.05 C

ATOM 397 O ASP A 31 13.724 -9.661 -5.136 1.00 4.74 O

ATOM 398 CB ASP A 31 14.454 -7.361 -3.358 1.00 0.00 C

ATOM 399 1HB ASP A 31 13.565 -7.853 -2.987 1.00 0.00 H

ATOM 400 2HB ASP A 31 15.303 -7.915 -2.979 1.00 0.00 H

ATOM 401 CG ASP A 31 14.545 -5.950 -2.809 1.00 0.00 C

ATOM 402 OD1 ASP A 31 15.358 -5.138 -3.306 1.00 0.00 O

ATOM 403 OD2 ASP A 31 13.822 -5.638 -1.837 1.00 0.00 O

ATOM 404 N ALA A 32 15.749 -9.179 -5.950 1.00 3.48 N

ATOM 405 H ALA A 32 16.454 -8.471 -6.105 1.00 3.48 H

ATOM 406 CA ALA A 32 16.067 -10.531 -6.377 1.00 3.86 C

ATOM 407 HA ALA A 32 15.289 -11.172 -5.994 1.00 3.86 H

ATOM 408 C ALA A 32 17.416 -10.916 -5.780 1.00 3.97 C

ATOM 409 O ALA A 32 18.398 -10.195 -5.947 1.00 4.44 O

ATOM 410 CB ALA A 32 16.082 -10.599 -7.903 1.00 4.72 C

ATOM 411 1HB ALA A 32 15.128 -10.242 -8.296 1.00 4.72 H

ATOM 412 2HB ALA A 32 16.246 -11.632 -8.208 1.00 4.72 H

ATOM 413 3HB ALA A 32 16.876 -9.950 -8.275 1.00 4.72 H

ATOM 414 N ARG A 33 17.432 -12.021 -5.031 1.00 3.86 N

ATOM 415 H ARG A 33 16.571 -12.549 -4.938 1.00 3.86 H

ATOM 416 CA ARG A 33 18.596 -12.414 -4.245 1.00 3.61 C

ATOM 417 HA ARG A 33 19.453 -11.829 -4.588 1.00 3.61 H

ATOM 418 C ARG A 33 18.962 -13.889 -4.393 1.00 3.76 C

ATOM 419 O ARG A 33 18.222 -14.758 -3.937 1.00 4.35 O

ATOM 420 CB ARG A 33 18.357 -12.064 -2.774 1.00 0.00 C

ATOM 421 1HB ARG A 33 17.631 -12.758 -2.357 1.00 0.00 H

ATOM 422 2HB ARG A 33 19.287 -12.215 -2.230 1.00 0.00 H

ATOM 423 CG ARG A 33 17.869 -10.641 -2.518 1.00 0.00 C

ATOM 424 1HG ARG A 33 18.595 -9.931 -2.906 1.00 0.00 H

ATOM 425 2HG ARG A 33 16.906 -10.515 -3.006 1.00 0.00 H

ATOM 426 CD ARG A 33 17.672 -10.396 -1.042 1.00 0.00 C

ATOM 427 1HD ARG A 33 17.023 -11.179 -0.641 1.00 0.00 H

ATOM 428 2HD ARG A 33 18.651 -10.440 -0.557 1.00 0.00 H

ATOM 429 NE ARG A 33 17.032 -9.114 -0.760 1.00 0.00 N

ATOM 430 HE ARG A 33 16.038 -9.126 -0.573 1.00 0.00 H

ATOM 431 CZ ARG A 33 17.640 -7.932 -0.771 1.00 0.00 C

ATOM 432 NH1 ARG A 33 16.920 -6.862 -0.505 1.00 0.00 N

ATOM 433 1HH1 ARG A 33 15.927 -6.979 -0.365 1.00 0.00 H

ATOM 434 2HH1 ARG A 33 17.364 -5.966 -0.439 1.00 0.00 H

ATOM 435 NH2 ARG A 33 18.943 -7.795 -0.967 1.00 0.00 N

ATOM 436 1HH2 ARG A 33 19.519 -8.613 -1.051 1.00 0.00 H

ATOM 437 2HH2 ARG A 33 19.357 -6.886 -0.950 1.00 0.00 H

ATOM 438 N SER A 34 20.101 -14.179 -5.002 1.00 3.50 N

ATOM 439 H SER A 34 20.693 -13.431 -5.335 1.00 3.50 H

ATOM 440 CA SER A 34 20.523 -15.554 -5.245 1.00 3.85 C

ATOM 441 HA SER A 34 19.774 -16.220 -4.842 1.00 3.85 H

ATOM 442 C SER A 34 21.849 -15.879 -4.575 1.00 3.48 C

ATOM 443 O SER A 34 22.757 -15.045 -4.529 1.00 5.07 O

ATOM 444 CB SER A 34 20.691 -15.790 -6.746 1.00 4.32 C

ATOM 445 1HB SER A 34 21.432 -15.106 -7.159 1.00 4.32 H

ATOM 446 2HB SER A 34 21.035 -16.809 -6.915 1.00 4.32 H

ATOM 447 OG SER A 34 19.475 -15.584 -7.436 1.00 5.06 O

ATOM 448 HG SER A 34 19.657 -15.446 -8.361 1.00 5.06 H

ATOM 449 N MET A 35 21.962 -17.097 -4.073 1.00 3.82 N

ATOM 450 H MET A 35 21.162 -17.713 -4.037 1.00 3.82 H

ATOM 451 CA MET A 35 23.202 -17.572 -3.468 1.00 3.77 C

ATOM 452 HA MET A 35 24.019 -17.084 -3.991 1.00 3.77 H

ATOM 453 C MET A 35 23.391 -19.089 -3.599 1.00 3.75 C

ATOM 454 O MET A 35 22.439 -19.852 -3.415 1.00 4.60 O

ATOM 455 CB MET A 35 23.266 -17.160 -1.995 1.00 0.00 C

ATOM 456 1HB MET A 35 24.202 -17.544 -1.555 1.00 0.00 H

ATOM 457 2HB MET A 35 23.317 -16.057 -1.960 1.00 0.00 H

ATOM 458 CG MET A 35 22.095 -17.635 -1.142 1.00 0.00 C

ATOM 459 1HG MET A 35 21.172 -17.241 -1.558 1.00 0.00 H

ATOM 460 2HG MET A 35 22.073 -18.721 -1.153 1.00 0.00 H

ATOM 461 SD MET A 35 22.187 -17.073 0.543 1.00 0.00 S

ATOM 462 CE MET A 35 23.525 -18.087 1.188 1.00 0.00 C

ATOM 463 1HE MET A 35 23.576 -17.991 2.271 1.00 0.00 H

ATOM 464 2HE MET A 35 24.459 -17.734 0.753 1.00 0.00 H

ATOM 465 3HE MET A 35 23.398 -19.137 0.929 1.00 0.00 H

ATOM 466 N SER A 36 24.602 -19.514 -3.930 1.00 3.70 N

ATOM 467 H SER A 36 25.331 -18.856 -4.162 1.00 3.70 H

ATOM 468 CA SER A 36 24.893 -20.948 -3.991 1.00 3.84 C

ATOM 469 HA SER A 36 24.324 -21.436 -3.204 1.00 3.84 H

ATOM 470 C SER A 36 26.365 -21.267 -3.741 1.00 4.67 C

ATOM 471 O SER A 36 27.205 -20.370 -3.737 1.00 4.48 O

ATOM 472 CB SER A 36 24.420 -21.508 -5.334 1.00 4.96 C

ATOM 473 1HB SER A 36 24.571 -22.595 -5.346 1.00 4.96 H

ATOM 474 2HB SER A 36 23.343 -21.302 -5.423 1.00 4.96 H

ATOM 475 OG SER A 36 25.112 -20.942 -6.431 1.00 5.59 O

ATOM 476 HG SER A 36 25.006 -21.539 -7.187 1.00 5.59 H

ATOM 477 N GLU A 37 26.689 -22.526 -3.492 1.00 4.69 N

ATOM 478 H GLU A 37 25.959 -23.227 -3.463 1.00 4.69 H

ATOM 479 CA GLU A 37 28.075 -22.950 -3.316 1.00 5.34 C

ATOM 480 HA GLU A 37 28.700 -22.297 -3.930 1.00 5.34 H

ATOM 481 C GLU A 37 28.347 -24.378 -3.775 1.00 5.71 C

ATOM 482 O GLU A 37 27.449 -25.221 -3.837 1.00 5.86 O

ATOM 483 CB GLU A 37 28.464 -22.747 -1.852 1.00 0.00 C

ATOM 484 1HB GLU A 37 29.542 -22.833 -1.766 1.00 0.00 H

ATOM 485 2HB GLU A 37 28.182 -21.741 -1.561 1.00 0.00 H

ATOM 486 CG GLU A 37 27.819 -23.729 -0.886 1.00 0.00 C

ATOM 487 1HG GLU A 37 26.727 -23.693 -0.997 1.00 0.00 H

ATOM 488 2HG GLU A 37 28.153 -24.745 -1.142 1.00 0.00 H

ATOM 489 CD GLU A 37 28.152 -23.429 0.568 1.00 0.00 C

ATOM 490 OE1 GLU A 37 28.878 -22.445 0.837 1.00 0.00 O

ATOM 491 OE2 GLU A 37 27.683 -24.194 1.440 1.00 0.00 O

ATOM 492 N GLY A 38 29.613 -24.657 -4.096 1.00 6.42 N

ATOM 493 H GLY A 38 30.331 -23.949 -4.005 1.00 6.42 H

ATOM 494 CA GLY A 38 30.022 -25.968 -4.573 1.00 6.74 C

ATOM 495 1HA GLY A 38 31.079 -26.110 -4.297 1.00 6.74 H

ATOM 496 2HA GLY A 38 29.412 -26.747 -4.090 1.00 6.74 H

ATOM 497 C GLY A 38 29.848 -26.050 -6.078 1.00 6.76 C

ATOM 498 O GLY A 38 30.582 -25.406 -6.828 1.00 8.12 O

ATOM 499 N ASN A 39 28.838 -26.813 -6.489 1.00 6.31 N

ATOM 500 H ASN A 39 28.344 -27.377 -5.811 1.00 6.31 H

ATOM 501 CA ASN A 39 28.389 -26.887 -7.870 1.00 7.22 C

ATOM 502 HA ASN A 39 28.986 -26.237 -8.511 1.00 7.22 H

ATOM 503 C ASN A 39 26.928 -26.457 -8.021 1.00 7.28 C

ATOM 504 O ASN A 39 26.341 -26.514 -9.102 1.00 10.66 O

ATOM 505 CB ASN A 39 28.549 -28.331 -8.356 1.00 0.00 C

ATOM 506 1HB ASN A 39 27.936 -28.965 -7.716 1.00 0.00 H

ATOM 507 2HB ASN A 39 28.194 -28.430 -9.383 1.00 0.00 H

ATOM 508 CG ASN A 39 29.990 -28.809 -8.311 1.00 0.00 C

ATOM 509 OD1 ASN A 39 30.884 -28.245 -8.956 1.00 0.00 O

ATOM 510 ND2 ASN A 39 30.227 -29.877 -7.579 1.00 0.00 N

ATOM 511 1HD2 ASN A 39 31.176 -30.183 -7.471 1.00 0.00 H

ATOM 512 2HD2 ASN A 39 29.460 -30.380 -7.181 1.00 0.00 H

ATOM 513 N ALA A 40 26.315 -26.041 -6.919 1.00 6.27 N

ATOM 514 H ALA A 40 26.845 -25.949 -6.065 1.00 6.27 H

ATOM 515 CA ALA A 40 24.883 -25.732 -6.852 1.00 5.65 C

ATOM 516 HA ALA A 40 24.290 -26.565 -7.230 1.00 5.65 H

ATOM 517 C ALA A 40 24.495 -24.504 -7.687 1.00 5.32 C

ATOM 518 O ALA A 40 25.343 -23.690 -8.059 1.00 6.32 O

ATOM 519 CB ALA A 40 24.511 -25.536 -5.401 1.00 0.00 C

ATOM 520 1HB ALA A 40 24.760 -26.425 -4.809 1.00 0.00 H

ATOM 521 2HB ALA A 40 23.430 -25.376 -5.335 1.00 0.00 H

ATOM 522 3HB ALA A 40 25.045 -24.677 -4.980 1.00 0.00 H

ATOM 523 N GLU A 41 23.198 -24.377 -7.943 1.00 4.60 N

ATOM 524 H GLU A 41 22.549 -25.065 -7.585 1.00 4.60 H

ATOM 525 CA GLU A 41 22.683 -23.270 -8.734 1.00 4.61 C

ATOM 526 HA GLU A 41 23.488 -22.555 -8.888 1.00 4.61 H

ATOM 527 C GLU A 41 21.520 -22.547 -8.063 1.00 4.46 C

ATOM 528 O GLU A 41 20.631 -23.166 -7.487 1.00 4.81 O

ATOM 529 CB GLU A 41 22.194 -23.768 -10.102 1.00 0.00 C

ATOM 530 1HB GLU A 41 21.444 -24.538 -9.954 1.00 0.00 H

ATOM 531 2HB GLU A 41 21.754 -22.918 -10.616 1.00 0.00 H

ATOM 532 CG GLU A 41 23.257 -24.381 -10.994 1.00 0.00 C

ATOM 533 1HG GLU A 41 24.014 -23.627 -11.178 1.00 0.00 H

ATOM 534 2HG GLU A 41 23.719 -25.212 -10.471 1.00 0.00 H

ATOM 535 CD GLU A 41 22.693 -24.866 -12.319 1.00 0.00 C

ATOM 536 OE1 GLU A 41 21.890 -25.817 -12.315 1.00 0.00 O

ATOM 537 OE2 GLU A 41 23.019 -24.290 -13.373 1.00 0.00 O

ATOM 538 N ALA A 42 21.516 -21.217 -8.141 1.00 3.88 N

ATOM 539 H ALA A 42 22.243 -20.725 -8.642 1.00 3.88 H

ATOM 540 CA ALA A 42 20.458 -20.401 -7.543 1.00 3.86 C

ATOM 541 HA ALA A 42 19.568 -21.019 -7.394 1.00 3.86 H

ATOM 542 C ALA A 42 20.094 -19.226 -8.446 1.00 3.67 C

ATOM 543 O ALA A 42 20.948 -18.371 -8.705 1.00 4.75 O

ATOM 544 CB ALA A 42 20.939 -19.901 -6.195 1.00 4.25 C

ATOM 545 1HB ALA A 42 21.020 -20.729 -5.484 1.00 4.25 H

ATOM 546 2HB ALA A 42 20.208 -19.189 -5.801 1.00 4.25 H

ATOM 547 3HB ALA A 42 21.907 -19.399 -6.276 1.00 4.25 H

ATOM 548 N TYR A 43 18.847 -19.152 -8.901 1.00 3.90 N

ATOM 549 H TYR A 43 18.179 -19.890 -8.702 1.00 3.90 H

ATOM 550 CA TYR A 43 18.434 -18.107 -9.841 1.00 4.12 C

ATOM 551 HA TYR A 43 19.247 -17.379 -9.896 1.00 4.12 H

ATOM 552 C TYR A 43 17.169 -17.376 -9.398 1.00 3.87 C

ATOM 553 O TYR A 43 16.231 -18.001 -8.904 1.00 4.48 O

ATOM 554 CB TYR A 43 18.239 -18.688 -11.242 1.00 0.00 C

ATOM 555 1HB TYR A 43 17.363 -19.327 -11.221 1.00 0.00 H

ATOM 556 2HB TYR A 43 18.050 -17.870 -11.927 1.00 0.00 H

ATOM 557 CG TYR A 43 19.426 -19.480 -11.762 1.00 0.00 C

ATOM 558 CD1 TYR A 43 19.302 -20.844 -12.049 1.00 0.00 C

ATOM 559 HD1 TYR A 43 18.345 -21.336 -11.911 1.00 0.00 H

ATOM 560 CD2 TYR A 43 20.663 -18.872 -11.963 1.00 0.00 C

ATOM 561 HD2 TYR A 43 20.784 -17.836 -11.678 1.00 0.00 H

ATOM 562 CE1 TYR A 43 20.384 -21.561 -12.567 1.00 0.00 C

ATOM 563 HE1 TYR A 43 20.252 -22.617 -12.809 1.00 0.00 H

ATOM 564 CE2 TYR A 43 21.748 -19.595 -12.448 1.00 0.00 C

ATOM 565 HE2 TYR A 43 22.720 -19.119 -12.558 1.00 0.00 H

ATOM 566 CZ TYR A 43 21.602 -20.938 -12.767 1.00 0.00 C

ATOM 567 OH TYR A 43 22.666 -21.628 -13.291 1.00 0.00 O

ATOM 568 HH TYR A 43 22.457 -22.550 -13.485 1.00 0.00 H

ATOM 569 N THR A 44 17.142 -16.060 -9.549 1.00 3.77 N

ATOM 570 H THR A 44 17.940 -15.567 -9.925 1.00 3.77 H

ATOM 571 CA THR A 44 15.965 -15.270 -9.172 1.00 3.65 C

ATOM 572 HA THR A 44 15.120 -15.941 -9.066 1.00 3.65 H

ATOM 573 C THR A 44 15.605 -14.207 -10.207 1.00 3.37 C

ATOM 574 O THR A 44 16.467 -13.582 -10.821 1.00 4.35 O

ATOM 575 CB THR A 44 16.176 -14.550 -7.835 1.00 3.95 C

ATOM 576 HB THR A 44 15.300 -13.929 -7.625 1.00 3.95 H

ATOM 577 OG1 THR A 44 17.334 -13.719 -7.916 1.00 5.08 O

ATOM 578 HG1 THR A 44 17.649 -13.536 -7.029 1.00 5.08 H

ATOM 579 CG2 THR A 44 16.333 -15.548 -6.688 1.00 4.33 C

ATOM 580 1HG2 THR A 44 16.401 -14.997 -5.748 1.00 4.33 H

ATOM 581 2HG2 THR A 44 17.242 -16.143 -6.809 1.00 4.33 H

ATOM 582 3HG2 THR A 44 15.466 -16.211 -6.648 1.00 4.33 H

ATOM 583 N GLU A 45 14.303 -13.984 -10.364 1.00 3.37 N

ATOM 584 H GLU A 45 13.626 -14.611 -9.954 1.00 3.37 H

ATOM 585 CA GLU A 45 13.796 -12.802 -11.066 1.00 3.75 C

ATOM 586 HA GLU A 45 14.610 -12.076 -11.188 1.00 3.75 H

ATOM 587 C GLU A 45 12.647 -12.170 -10.272 1.00 3.54 C

ATOM 588 O GLU A 45 11.671 -12.846 -9.964 1.00 5.79 O

ATOM 589 CB GLU A 45 13.281 -13.187 -12.459 1.00 0.00 C

ATOM 590 1HB GLU A 45 12.496 -13.919 -12.315 1.00 0.00 H

ATOM 591 2HB GLU A 45 12.880 -12.294 -12.932 1.00 0.00 H

ATOM 592 CG GLU A 45 14.294 -13.799 -13.399 1.00 0.00 C

ATOM 593 1HG GLU A 45 15.117 -13.090 -13.503 1.00 0.00 H

ATOM 594 2HG GLU A 45 14.667 -14.725 -12.953 1.00 0.00 H

ATOM 595 CD GLU A 45 13.695 -14.095 -14.760 1.00 0.00 C

ATOM 596 OE1 GLU A 45 12.674 -14.822 -14.857 1.00 0.00 O

ATOM 597 OE2 GLU A 45 14.226 -13.544 -15.749 1.00 0.00 O

ATOM 598 N ALA A 46 12.737 -10.876 -9.991 1.00 3.83 N

ATOM 599 H ALA A 46 13.557 -10.355 -10.282 1.00 3.83 H

ATOM 600 CA ALA A 46 11.668 -10.168 -9.278 1.00 4.10 C

ATOM 601 HA ALA A 46 10.802 -10.829 -9.196 1.00 4.10 H

ATOM 602 C ALA A 46 11.240 -8.931 -10.062 1.00 3.90 C

ATOM 603 O ALA A 46 12.069 -8.240 -10.660 1.00 4.80 O

ATOM 604 CB ALA A 46 12.111 -9.794 -7.876 1.00 5.06 C

ATOM 605 1HB ALA A 46 12.507 -10.674 -7.368 1.00 5.06 H

ATOM 606 2HB ALA A 46 11.254 -9.432 -7.305 1.00 5.06 H

ATOM 607 3HB ALA A 46 12.876 -9.017 -7.924 1.00 5.06 H

ATOM 608 N LYS A 47 9.935 -8.680 -10.085 1.00 4.51 N

ATOM 609 H LYS A 47 9.308 -9.288 -9.569 1.00 4.51 H

ATOM 610 CA LYS A 47 9.363 -7.598 -10.883 1.00 5.31 C

ATOM 611 HA LYS A 47 10.154 -6.905 -11.179 1.00 5.31 H

ATOM 612 C LYS A 47 8.293 -6.834 -10.113 1.00 5.30 C

ATOM 613 O LYS A 47 7.491 -7.390 -9.376 1.00 5.92 O

ATOM 614 CB LYS A 47 8.717 -8.161 -12.158 1.00 0.00 C

ATOM 615 1HB LYS A 47 7.933 -8.852 -11.833 1.00 0.00 H

ATOM 616 2HB LYS A 47 8.267 -7.341 -12.725 1.00 0.00 H

ATOM 617 CG LYS A 47 9.698 -8.905 -13.055 1.00 0.00 C

ATOM 618 1HG LYS A 47 10.524 -8.225 -13.291 1.00 0.00 H

ATOM 619 2HG LYS A 47 10.112 -9.750 -12.492 1.00 0.00 H

ATOM 620 CD LYS A 47 9.075 -9.391 -14.353 1.00 0.00 C

ATOM 621 1HD LYS A 47 8.222 -10.033 -14.132 1.00 0.00 H

ATOM 622 2HD LYS A 47 8.731 -8.540 -14.942 1.00 0.00 H

ATOM 623 CE LYS A 47 10.083 -10.196 -15.164 1.00 0.00 C

ATOM 624 1HE LYS A 47 10.922 -9.548 -15.423 1.00 0.00 H

ATOM 625 2HE LYS A 47 10.448 -11.044 -14.582 1.00 0.00 H

ATOM 626 NZ LYS A 47 9.429 -10.701 -16.424 1.00 0.00 N

ATOM 627 1HZ LYS A 47 10.080 -11.242 -16.987 1.00 0.00 H

ATOM 628 2HZ LYS A 47 9.106 -9.926 -16.979 1.00 0.00 H

ATOM 629 3HZ LYS A 47 8.636 -11.305 -16.219 1.00 0.00 H

ATOM 630 N GLY A 48 8.299 -5.520 -10.298 1.00 5.31 N

ATOM 631 H GLY A 48 8.972 -5.102 -10.928 1.00 5.31 H

ATOM 632 CA GLY A 48 7.368 -4.669 -9.574 1.00 6.16 C

ATOM 633 1HA GLY A 48 7.088 -3.821 -10.199 1.00 6.16 H

ATOM 634 2HA GLY A 48 6.465 -5.234 -9.337 1.00 6.16 H

ATOM 635 C GLY A 48 8.006 -4.203 -8.282 1.00 5.64 C

ATOM 636 O GLY A 48 9.058 -3.576 -8.312 1.00 6.88 O

ATOM 637 N THR A 49 7.396 -4.529 -7.145 1.00 6.21 N

ATOM 638 H THR A 49 6.516 -5.029 -7.189 1.00 6.21 H

ATOM 639 CA THR A 49 8.021 -4.349 -5.834 1.00 6.87 C

ATOM 640 HA THR A 49 9.066 -4.055 -5.972 1.00 6.87 H

ATOM 641 C THR A 49 7.934 -5.707 -5.139 1.00 6.33 C

ATOM 642 O THR A 49 7.368 -5.846 -4.056 1.00 6.73 O

ATOM 643 CB THR A 49 7.318 -3.268 -4.975 1.00 8.01 C

ATOM 644 HB THR A 49 6.345 -3.628 -4.658 1.00 8.01 H

ATOM 645 OG1 THR A 49 7.155 -2.089 -5.767 1.00 9.95 O

ATOM 646 HG1 THR A 49 7.074 -2.348 -6.697 1.00 9.95 H

ATOM 647 CG2 THR A 49 8.165 -2.879 -3.768 1.00 9.91 C

ATOM 648 1HG2 THR A 49 7.667 -2.090 -3.204 1.00 9.91 H

ATOM 649 2HG2 THR A 49 9.151 -2.531 -4.081 1.00 9.91 H

ATOM 650 3HG2 THR A 49 8.304 -3.754 -3.132 1.00 9.91 H

ATOM 651 N ALA A 50 8.459 -6.732 -5.798 1.00 5.67 N

ATOM 652 H ALA A 50 8.923 -6.566 -6.680 1.00 5.67 H

ATOM 653 CA ALA A 50 8.345 -8.116 -5.360 1.00 5.03 C

ATOM 654 HA ALA A 50 7.553 -8.171 -4.619 1.00 5.03 H

ATOM 655 C ALA A 50 9.661 -8.600 -4.744 1.00 4.55 C

ATOM 656 O ALA A 50 10.695 -7.937 -4.830 1.00 6.17 O

ATOM 657 CB ALA A 50 7.958 -8.974 -6.554 1.00 5.98 C

ATOM 658 1HB ALA A 50 7.052 -8.579 -7.018 1.00 5.98 H

ATOM 659 2HB ALA A 50 7.783 -9.996 -6.221 1.00 5.98 H

ATOM 660 3HB ALA A 50 8.776 -8.971 -7.278 1.00 5.98 H

ATOM 661 N MET A 51 9.616 -9.794 -4.157 1.00 5.02 N

ATOM 662 H MET A 51 8.744 -10.300 -4.150 1.00 5.02 H

ATOM 663 CA MET A 51 10.784 -10.400 -3.528 1.00 5.27 C

ATOM 664 HA MET A 51 11.672 -9.835 -3.815 1.00 5.27 H

ATOM 665 C MET A 51 10.944 -11.841 -4.014 1.00 4.56 C

ATOM 666 O MET A 51 10.032 -12.655 -3.871 1.00 5.78 O

ATOM 667 CB MET A 51 10.613 -10.409 -2.008 1.00 0.00 C

ATOM 668 1HB MET A 51 10.606 -9.388 -1.637 1.00 0.00 H

ATOM 669 2HB MET A 51 9.662 -10.883 -1.782 1.00 0.00 H

ATOM 670 CG MET A 51 11.699 -11.161 -1.245 1.00 0.00 C

ATOM 671 1HG MET A 51 11.425 -11.199 -0.202 1.00 0.00 H

ATOM 672 2HG MET A 51 11.749 -12.169 -1.632 1.00 0.00 H

ATOM 673 SD MET A 51 13.334 -10.404 -1.376 1.00 0.00 S

ATOM 674 CE MET A 51 13.243 -9.142 -0.106 1.00 0.00 C

ATOM 675 1HE MET A 51 14.162 -8.558 -0.083 1.00 0.00 H

ATOM 676 2HE MET A 51 13.118 -9.599 0.876 1.00 0.00 H

ATOM 677 3HE MET A 51 12.435 -8.443 -0.328 1.00 0.00 H

ATOM 678 N ALA A 52 12.115 -12.172 -4.546 1.00 4.40 N

ATOM 679 H ALA A 52 12.832 -11.462 -4.638 1.00 4.40 H

ATOM 680 CA ALA A 52 12.413 -13.540 -4.976 1.00 4.40 C

ATOM 681 HA ALA A 52 11.677 -14.220 -4.548 1.00 4.40 H

ATOM 682 C ALA A 52 13.799 -13.959 -4.490 1.00 4.18 C

ATOM 683 O ALA A 52 14.804 -13.300 -4.763 1.00 4.82 O

ATOM 684 CB ALA A 52 12.354 -13.656 -6.494 1.00 5.16 C

ATOM 685 1HB ALA A 52 11.351 -13.410 -6.853 1.00 5.16 H

ATOM 686 2HB ALA A 52 12.619 -14.668 -6.814 1.00 5.16 H

ATOM 687 3HB ALA A 52 13.049 -12.931 -6.925 1.00 5.16 H

ATOM 688 N THR A 53 13.856 -15.064 -3.758 1.00 4.36 N

ATOM 689 H THR A 53 13.014 -15.601 -3.586 1.00 4.36 H

ATOM 690 CA THR A 53 15.108 -15.553 -3.184 1.00 4.45 C

ATOM 691 HA THR A 53 15.931 -14.958 -3.595 1.00 4.45 H

ATOM 692 C THR A 53 15.360 -17.012 -3.565 1.00 4.26 C

ATOM 693 O THR A 53 14.424 -17.797 -3.722 1.00 5.49 O

ATOM 694 CB THR A 53 15.115 -15.441 -1.642 1.00 5.48 C

ATOM 695 HB THR A 53 16.069 -15.797 -1.262 1.00 5.48 H

ATOM 696 OG1 THR A 53 14.047 -16.229 -1.098 1.00 7.20 O

ATOM 697 HG1 THR A 53 13.222 -15.963 -1.499 1.00 7.20 H

ATOM 698 CG2 THR A 53 14.937 -13.998 -1.181 1.00 5.54 C

ATOM 699 1HG2 THR A 53 15.114 -13.915 -0.100 1.00 5.54 H

ATOM 700 2HG2 THR A 53 13.924 -13.630 -1.393 1.00 5.54 H

ATOM 701 3HG2 THR A 53 15.668 -13.355 -1.690 1.00 5.54 H

ATOM 702 N SER A 54 16.641 -17.369 -3.708 1.00 3.54 N

ATOM 703 H SER A 54 17.372 -16.671 -3.645 1.00 3.54 H

ATOM 704 CA SER A 54 17.038 -18.745 -4.006 1.00 3.40 C

ATOM 705 HA SER A 54 16.254 -19.433 -3.671 1.00 3.40 H

ATOM 706 C SER A 54 18.333 -19.087 -3.281 1.00 3.84 C

ATOM 707 O SER A 54 19.243 -18.271 -3.170 1.00 4.30 O

ATOM 708 CB SER A 54 17.228 -18.928 -5.513 1.00 4.27 C

ATOM 709 1HB SER A 54 18.016 -18.239 -5.829 1.00 4.27 H

ATOM 710 2HB SER A 54 17.537 -19.957 -5.726 1.00 4.27 H

ATOM 711 OG SER A 54 16.036 -18.675 -6.235 1.00 5.04 O

ATOM 712 HG SER A 54 16.254 -18.556 -7.160 1.00 5.04 H

ATOM 713 N GLU A 55 18.428 -20.324 -2.802 1.00 3.67 N

ATOM 714 H GLU A 55 17.655 -20.965 -2.932 1.00 3.67 H

ATOM 715 CA GLU A 55 19.590 -20.820 -2.068 1.00 3.84 C

ATOM 716 HA GLU A 55 20.472 -20.273 -2.403 1.00 3.84 H

ATOM 717 C GLU A 55 19.763 -22.301 -2.389 1.00 4.05 C

ATOM 718 O GLU A 55 18.798 -23.065 -2.333 1.00 5.18 O

ATOM 719 CB GLU A 55 19.374 -20.607 -0.564 1.00 0.00 C

ATOM 720 1HB GLU A 55 19.200 -19.545 -0.392 1.00 0.00 H

ATOM 721 2HB GLU A 55 18.460 -21.120 -0.264 1.00 0.00 H

ATOM 722 CG GLU A 55 20.504 -21.116 0.329 1.00 0.00 C

ATOM 723 1HG GLU A 55 20.634 -22.192 0.200 1.00 0.00 H

ATOM 724 2HG GLU A 55 21.440 -20.620 0.064 1.00 0.00 H

ATOM 725 CD GLU A 55 20.181 -20.833 1.786 1.00 0.00 C

ATOM 726 OE1 GLU A 55 19.061 -21.150 2.250 1.00 0.00 O

ATOM 727 OE2 GLU A 55 21.089 -20.304 2.477 1.00 0.00 O

ATOM 728 N ALA A 56 20.982 -22.718 -2.711 1.00 3.89 N

ATOM 729 H ALA A 56 21.744 -22.059 -2.800 1.00 3.89 H

ATOM 730 CA ALA A 56 21.267 -24.126 -2.975 1.00 3.82 C

ATOM 731 HA ALA A 56 20.607 -24.726 -2.347 1.00 3.82 H

ATOM 732 C ALA A 56 22.720 -24.394 -2.614 1.00 4.18 C

ATOM 733 O ALA A 56 23.598 -23.563 -2.777 1.00 4.88 O

ATOM 734 CB ALA A 56 21.025 -24.457 -4.436 1.00 4.50 C

ATOM 735 1HB ALA A 56 19.988 -24.239 -4.685 1.00 4.50 H

ATOM 736 2HB ALA A 56 21.245 -25.502 -4.638 1.00 4.50 H

ATOM 737 3HB ALA A 56 21.661 -23.837 -5.067 1.00 4.50 H

ATOM 738 N SER A 57 23.004 -25.581 -2.069 1.00 4.79 N

ATOM 739 H SER A 57 22.251 -26.246 -1.950 1.00 4.79 H

ATOM 740 CA SER A 57 24.358 -25.954 -1.690 1.00 5.53 C

ATOM 741 HA SER A 57 25.061 -25.294 -2.202 1.00 5.53 H

ATOM 742 C SER A 57 24.669 -27.388 -2.109 1.00 6.16 C

ATOM 743 O SER A 57 23.781 -28.238 -2.141 1.00 9.89 O

ATOM 744 CB SER A 57 24.550 -25.800 -0.176 1.00 0.00 C

ATOM 745 1HB SER A 57 23.905 -26.514 0.321 1.00 0.00 H

ATOM 746 2HB SER A 57 25.581 -26.023 0.069 1.00 0.00 H

ATOM 747 OG SER A 57 24.229 -24.485 0.246 1.00 0.00 O

ATOM 748 HG SER A 57 23.479 -24.194 -0.278 1.00 0.00 H

ATOM 749 N GLY A 58 25.943 -27.645 -2.397 1.00 5.90 N

ATOM 750 H GLY A 58 26.641 -26.919 -2.307 1.00 5.90 H

ATOM 751 CA GLY A 58 26.350 -28.947 -2.899 1.00 5.96 C

ATOM 752 1HA GLY A 58 27.350 -29.165 -2.553 1.00 5.96 H

ATOM 753 2HA GLY A 58 25.663 -29.703 -2.545 1.00 5.96 H

ATOM 754 C GLY A 58 26.327 -28.968 -4.416 1.00 5.73 C

ATOM 755 O GLY A 58 27.220 -28.415 -5.047 1.00 6.97 O

ATOM 756 N GLU A 59 25.305 -29.594 -4.976 1.00 5.47 N

ATOM 757 H GLU A 59 24.616 -30.054 -4.397 1.00 5.47 H

ATOM 758 CA GLU A 59 25.049 -29.615 -6.418 1.00 5.26 C

ATOM 759 HA GLU A 59 25.485 -28.735 -6.882 1.00 5.26 H

ATOM 760 C GLU A 59 23.533 -29.557 -6.624 1.00 4.99 C

ATOM 761 O GLU A 59 22.970 -30.139 -7.542 1.00 6.03 O

ATOM 762 CB GLU A 59 25.700 -30.861 -7.042 1.00 0.00 C

ATOM 763 1HB GLU A 59 25.419 -30.865 -8.095 1.00 0.00 H

ATOM 764 2HB GLU A 59 26.780 -30.812 -6.901 1.00 0.00 H

ATOM 765 CG GLU A 59 25.211 -32.180 -6.453 1.00 0.00 C

ATOM 766 1HG GLU A 59 25.343 -32.191 -5.367 1.00 0.00 H

ATOM 767 2HG GLU A 59 24.142 -32.269 -6.656 1.00 0.00 H

ATOM 768 CD GLU A 59 25.899 -33.372 -7.073 1.00 0.00 C

ATOM 769 OE1 GLU A 59 26.659 -33.200 -8.050 1.00 0.00 O

ATOM 770 OE2 GLU A 59 25.695 -34.492 -6.566 1.00 0.00 O

ATOM 771 N ALA A 60 22.881 -28.826 -5.736 1.00 4.84 N

ATOM 772 H ALA A 60 23.400 -28.390 -4.988 1.00 4.84 H

ATOM 773 CA ALA A 60 21.431 -28.653 -5.771 1.00 5.06 C

ATOM 774 HA ALA A 60 20.952 -29.541 -6.205 1.00 5.06 H

ATOM 775 C ALA A 60 21.032 -27.454 -6.633 1.00 4.86 C

ATOM 776 O ALA A 60 21.878 -26.636 -7.020 1.00 5.96 O

ATOM 777 CB ALA A 60 20.925 -28.494 -4.341 1.00 0.00 C

ATOM 778 1HB ALA A 60 21.168 -29.354 -3.720 1.00 0.00 H

ATOM 779 2HB ALA A 60 19.843 -28.403 -4.376 1.00 0.00 H

ATOM 780 3HB ALA A 60 21.325 -27.588 -3.894 1.00 0.00 H

ATOM 781 N ARG A 61 19.743 -27.333 -6.934 1.00 4.82 N

ATOM 782 H ARG A 61 19.064 -27.983 -6.555 1.00 4.82 H

ATOM 783 CA ARG A 61 19.250 -26.223 -7.735 1.00 4.66 C

ATOM 784 HA ARG A 61 20.046 -25.486 -7.856 1.00 4.66 H

ATOM 785 C ARG A 61 18.064 -25.557 -7.037 1.00 4.06 C

ATOM 786 O ARG A 61 17.169 -26.222 -6.507 1.00 5.28 O

ATOM 787 CB ARG A 61 18.797 -26.698 -9.118 1.00 0.00 C

ATOM 788 1HB ARG A 61 17.963 -27.375 -8.994 1.00 0.00 H

ATOM 789 2HB ARG A 61 18.401 -25.842 -9.647 1.00 0.00 H

ATOM 790 CG ARG A 61 19.848 -27.353 -9.985 1.00 0.00 C

ATOM 791 1HG ARG A 61 20.720 -26.693 -10.050 1.00 0.00 H

ATOM 792 2HG ARG A 61 20.155 -28.277 -9.482 1.00 0.00 H

ATOM 793 CD ARG A 61 19.344 -27.686 -11.382 1.00 0.00 C

ATOM 794 1HD ARG A 61 19.017 -26.760 -11.859 1.00 0.00 H

ATOM 795 2HD ARG A 61 20.166 -28.092 -11.975 1.00 0.00 H

ATOM 796 NE ARG A 61 18.216 -28.625 -11.343 1.00 0.00 N

ATOM 797 HE ARG A 61 17.293 -28.234 -11.444 1.00 0.00 H

ATOM 798 CZ ARG A 61 18.301 -29.935 -11.177 1.00 0.00 C

ATOM 799 NH1 ARG A 61 17.200 -30.655 -11.153 1.00 0.00 N

ATOM 800 1HH1 ARG A 61 16.315 -30.194 -11.289 1.00 0.00 H

ATOM 801 2HH1 ARG A 61 17.246 -31.641 -10.951 1.00 0.00 H

ATOM 802 NH2 ARG A 61 19.457 -30.537 -10.995 1.00 0.00 N

ATOM 803 1HH2 ARG A 61 20.310 -29.986 -11.019 1.00 0.00 H

ATOM 804 2HH2 ARG A 61 19.472 -31.516 -10.819 1.00 0.00 H

ATOM 805 N ALA A 62 18.053 -24.229 -7.052 1.00 3.77 N

ATOM 806 H ALA A 62 18.848 -23.711 -7.403 1.00 3.77 H

ATOM 807 CA ALA A 62 16.883 -23.484 -6.599 1.00 3.95 C

ATOM 808 HA ALA A 62 16.020 -24.145 -6.591 1.00 3.95 H

ATOM 809 C ALA A 62 16.587 -22.331 -7.554 1.00 4.03 C

ATOM 810 O ALA A 62 17.479 -21.575 -7.926 1.00 4.55 O

ATOM 811 CB ALA A 62 17.101 -22.956 -5.193 1.00 4.86 C

ATOM 812 1HB ALA A 62 17.213 -23.785 -4.500 1.00 4.86 H

ATOM 813 2HB ALA A 62 16.261 -22.340 -4.888 1.00 4.86 H

ATOM 814 3HB ALA A 62 18.005 -22.357 -5.159 1.00 4.86 H

ATOM 815 N GLN A 63 15.326 -22.175 -7.952 1.00 4.06 N

ATOM 816 H GLN A 63 14.596 -22.801 -7.638 1.00 4.06 H

ATOM 817 CA GLN A 63 14.967 -21.070 -8.841 1.00 4.16 C

ATOM 818 HA GLN A 63 15.681 -20.266 -8.636 1.00 4.16 H

ATOM 819 C GLN A 63 13.587 -20.493 -8.556 1.00 3.71 C

ATOM 820 O GLN A 63 12.647 -21.220 -8.246 1.00 5.04 O

ATOM 821 CB GLN A 63 15.059 -21.447 -10.322 1.00 0.00 C

ATOM 822 1HB GLN A 63 14.650 -20.636 -10.922 1.00 0.00 H

ATOM 823 2HB GLN A 63 16.111 -21.545 -10.582 1.00 0.00 H

ATOM 824 CG GLN A 63 14.347 -22.748 -10.708 1.00 0.00 C

ATOM 825 1HG GLN A 63 13.384 -22.849 -10.199 1.00 0.00 H

ATOM 826 2HG GLN A 63 14.134 -22.714 -11.782 1.00 0.00 H

ATOM 827 CD GLN A 63 15.183 -23.984 -10.419 1.00 0.00 C

ATOM 828 OE1 GLN A 63 16.343 -24.073 -10.815 1.00 0.00 O

ATOM 829 NE2 GLN A 63 14.582 -24.955 -9.739 1.00 0.00 N

ATOM 830 1HE2 GLN A 63 15.085 -25.816 -9.566 1.00 0.00 H

ATOM 831 2HE2 GLN A 63 13.618 -24.842 -9.445 1.00 0.00 H

ATOM 832 N THR A 64 13.487 -19.176 -8.623 1.00 3.92 N

ATOM 833 H THR A 64 14.246 -18.636 -9.012 1.00 3.92 H

ATOM 834 CA THR A 64 12.390 -18.454 -7.985 1.00 3.64 C

ATOM 835 HA THR A 64 11.510 -19.089 -7.913 1.00 3.64 H

ATOM 836 C THR A 64 12.031 -17.199 -8.769 1.00 3.55 C

ATOM 837 O THR A 64 12.924 -16.520 -9.272 1.00 3.99 O

ATOM 838 CB THR A 64 12.819 -18.073 -6.546 1.00 4.71 C

ATOM 839 HB THR A 64 13.648 -17.364 -6.603 1.00 4.71 H

ATOM 840 OG1 THR A 64 13.259 -19.248 -5.863 1.00 5.62 O

ATOM 841 HG1 THR A 64 13.874 -18.978 -5.170 1.00 5.62 H

ATOM 842 CG2 THR A 64 11.680 -17.460 -5.743 1.00 4.99 C

ATOM 843 1HG2 THR A 64 12.015 -17.265 -4.727 1.00 4.99 H

ATOM 844 2HG2 THR A 64 10.840 -18.151 -5.731 1.00 4.99 H

ATOM 845 3HG2 THR A 64 11.403 -16.514 -6.204 1.00 4.99 H

ATOM 846 N ASN A 65 10.749 -16.884 -8.878 1.00 3.62 N

ATOM 847 H ASN A 65 10.035 -17.532 -8.579 1.00 3.62 H

ATOM 848 CA ASN A 65 10.336 -15.570 -9.365 1.00 3.84 C

ATOM 849 HA ASN A 65 11.173 -14.891 -9.219 1.00 3.84 H

ATOM 850 C ASN A 65 9.149 -15.004 -8.589 1.00 3.78 C

ATOM 851 O ASN A 65 8.360 -15.747 -8.001 1.00 5.73 O

ATOM 852 CB ASN A 65 10.022 -15.639 -10.866 1.00 0.00 C

ATOM 853 1HB ASN A 65 10.031 -14.625 -11.254 1.00 0.00 H

ATOM 854 2HB ASN A 65 10.816 -16.167 -11.386 1.00 0.00 H

ATOM 855 CG ASN A 65 8.681 -16.286 -11.174 1.00 0.00 C

ATOM 856 OD1 ASN A 65 8.539 -17.509 -11.142 1.00 0.00 O

ATOM 857 ND2 ASN A 65 7.700 -15.470 -11.496 1.00 0.00 N

ATOM 858 1HD2 ASN A 65 6.773 -15.857 -11.652 1.00 0.00 H

ATOM 859 2HD2 ASN A 65 7.872 -14.492 -11.583 1.00 0.00 H

ATOM 860 N ALA A 66 9.026 -13.679 -8.601 1.00 3.83 N

ATOM 861 H ALA A 66 9.713 -13.107 -9.071 1.00 3.83 H

ATOM 862 CA ALA A 66 7.896 -13.012 -7.965 1.00 4.33 C

ATOM 863 HA ALA A 66 7.036 -13.679 -7.991 1.00 4.33 H

ATOM 864 C ALA A 66 7.526 -11.736 -8.714 1.00 4.58 C

ATOM 865 O ALA A 66 8.393 -11.050 -9.256 1.00 5.22 O

ATOM 866 CB ALA A 66 8.202 -12.682 -6.517 1.00 5.11 C

ATOM 867 1HB ALA A 66 8.486 -13.600 -5.994 1.00 5.11 H

ATOM 868 2HB ALA A 66 7.338 -12.244 -6.010 1.00 5.11 H

ATOM 869 3HB ALA A 66 9.028 -11.969 -6.457 1.00 5.11 H

ATOM 870 N ASP A 67 6.235 -11.435 -8.765 1.00 4.38 N

ATOM 871 H ASP A 67 5.572 -12.003 -8.257 1.00 4.38 H

ATOM 872 CA ASP A 67 5.718 -10.298 -9.519 1.00 5.21 C

ATOM 873 HA ASP A 67 6.528 -9.585 -9.683 1.00 5.21 H

ATOM 874 C ASP A 67 4.607 -9.534 -8.814 1.00 5.34 C

ATOM 875 O ASP A 67 3.593 -10.103 -8.406 1.00 7.10 O

ATOM 876 CB ASP A 67 5.203 -10.745 -10.891 1.00 0.00 C

ATOM 877 1HB ASP A 67 5.975 -11.318 -11.410 1.00 0.00 H

ATOM 878 2HB ASP A 67 4.328 -11.378 -10.726 1.00 0.00 H

ATOM 879 CG ASP A 67 4.817 -9.565 -11.787 1.00 0.00 C

ATOM 880 OD1 ASP A 67 5.422 -8.478 -11.659 1.00 0.00 O

ATOM 881 OD2 ASP A 67 3.920 -9.777 -12.629 1.00 0.00 O

ATOM 882 N GLY A 68 4.789 -8.217 -8.698 1.00 5.76 N

ATOM 883 H GLY A 68 5.634 -7.781 -9.045 1.00 5.76 H

ATOM 884 CA GLY A 68 3.779 -7.378 -8.060 1.00 6.31 C

ATOM 885 1HA GLY A 68 3.553 -6.518 -8.689 1.00 6.31 H

ATOM 886 2HA GLY A 68 2.865 -7.955 -7.923 1.00 6.31 H

ATOM 887 C GLY A 68 4.234 -6.848 -6.720 1.00 7.98 C

ATOM 888 O GLY A 68 5.094 -5.967 -6.646 1.00 11.24 O

ATOM 889 N ARG A 69 3.628 -7.383 -5.661 1.00 9.76 N

ATOM 890 H ARG A 69 2.760 -7.885 -5.800 1.00 9.76 H

ATOM 891 CA ARG A 69 4.190 -7.335 -4.317 1.00 9.18 C

ATOM 892 HA ARG A 69 5.169 -6.848 -4.328 1.00 9.18 H

ATOM 893 C ARG A 69 4.367 -8.749 -3.785 1.00 8.26 C

ATOM 894 O ARG A 69 4.451 -8.935 -2.568 1.00 10.36 O

ATOM 895 CB ARG A 69 3.245 -6.589 -3.369 1.00 0.00 C

ATOM 896 1HB ARG A 69 2.296 -7.134 -3.300 1.00 0.00 H

ATOM 897 2HB ARG A 69 3.707 -6.577 -2.375 1.00 0.00 H

ATOM 898 CG ARG A 69 2.956 -5.141 -3.760 1.00 0.00 C

ATOM 899 1HG ARG A 69 3.904 -4.614 -3.776 1.00 0.00 H

ATOM 900 2HG ARG A 69 2.490 -5.088 -4.740 1.00 0.00 H

ATOM 901 CD ARG A 69 2.068 -4.471 -2.722 1.00 0.00 C

ATOM 902 1HD ARG A 69 1.153 -5.042 -2.571 1.00 0.00 H

ATOM 903 2HD ARG A 69 2.605 -4.451 -1.774 1.00 0.00 H

ATOM 904 NE ARG A 69 1.740 -3.093 -3.091 1.00 0.00 N

ATOM 905 HE ARG A 69 2.371 -2.379 -2.759 1.00 0.00 H

ATOM 906 CZ ARG A 69 0.710 -2.694 -3.830 1.00 0.00 C

ATOM 907 NH1 ARG A 69 0.586 -1.423 -4.162 1.00 0.00 N

ATOM 908 1HH1 ARG A 69 1.290 -0.768 -3.871 1.00 0.00 H

ATOM 909 2HH1 ARG A 69 -0.231 -1.120 -4.656 1.00 0.00 H

ATOM 910 NH2 ARG A 69 -0.217 -3.536 -4.237 1.00 0.00 N

ATOM 911 1HH2 ARG A 69 -0.136 -4.517 -4.007 1.00 0.00 H

ATOM 912 2HH2 ARG A 69 -0.962 -3.201 -4.811 1.00 0.00 H

ATOM 913 N ALA A 70 4.342 -9.721 -4.691 1.00 5.73 N

ATOM 914 H ALA A 70 4.287 -9.473 -5.669 1.00 5.73 H

ATOM 915 CA ALA A 70 4.384 -11.128 -4.298 1.00 6.16 C

ATOM 916 HA ALA A 70 3.640 -11.320 -3.519 1.00 6.16 H

ATOM 917 C ALA A 70 5.749 -11.491 -3.723 1.00 6.39 C

ATOM 918 O ALA A 70 6.743 -10.821 -4.006 1.00 9.87 O

ATOM 919 CB ALA A 70 4.059 -11.990 -5.505 1.00 7.46 C

ATOM 920 1HB ALA A 70 3.002 -11.881 -5.742 1.00 7.46 H

ATOM 921 2HB ALA A 70 4.270 -13.036 -5.296 1.00 7.46 H

ATOM 922 3HB ALA A 70 4.660 -11.680 -6.358 1.00 7.46 H

ATOM 923 N HIS A 71 5.773 -12.531 -2.898 1.00 5.49 N

ATOM 924 H HIS A 71 4.918 -13.033 -2.695 1.00 5.49 H

ATOM 925 CA HIS A 71 7.012 -13.033 -2.292 1.00 5.70 C

ATOM 926 HA HIS A 71 7.874 -12.490 -2.694 1.00 5.70 H

ATOM 927 C HIS A 71 7.202 -14.514 -2.620 1.00 5.07 C

ATOM 928 O HIS A 71 6.245 -15.288 -2.579 1.00 6.51 O

ATOM 929 CB HIS A 71 6.960 -12.857 -0.769 1.00 0.00 C

ATOM 930 1HB HIS A 71 6.003 -13.235 -0.411 1.00 0.00 H

ATOM 931 2HB HIS A 71 7.760 -13.454 -0.331 1.00 0.00 H

ATOM 932 CG HIS A 71 7.127 -11.433 -0.325 1.00 0.00 C

ATOM 933 ND1 HIS A 71 6.241 -10.409 -0.595 1.00 0.00 N

ATOM 934 HD1 HIS A 71 5.376 -10.487 -1.108 1.00 0.00 H

ATOM 935 CD2 HIS A 71 8.103 -10.849 0.426 1.00 0.00 C

ATOM 936 HD2 HIS A 71 8.938 -11.379 0.853 1.00 0.00 H

ATOM 937 CE1 HIS A 71 6.727 -9.290 -0.038 1.00 0.00 C

ATOM 938 HE1 HIS A 71 6.241 -8.325 -0.085 1.00 0.00 H

ATOM 939 NE2 HIS A 71 7.864 -9.516 0.588 1.00 0.00 N

ATOM 940 N SER A 72 8.428 -14.895 -2.952 1.00 4.56 N

ATOM 941 H SER A 72 9.177 -14.219 -3.018 1.00 4.56 H

ATOM 942 CA SER A 72 8.725 -16.282 -3.292 1.00 4.26 C

ATOM 943 HA SER A 72 7.972 -16.925 -2.838 1.00 4.26 H

ATOM 944 C SER A 72 10.113 -16.690 -2.800 1.00 4.41 C

ATOM 945 O SER A 72 11.013 -15.854 -2.716 1.00 5.01 O

ATOM 946 CB SER A 72 8.634 -16.447 -4.811 1.00 0.00 C

ATOM 947 1HB SER A 72 9.431 -15.850 -5.248 1.00 0.00 H

ATOM 948 2HB SER A 72 8.768 -17.491 -5.089 1.00 0.00 H

ATOM 949 OG SER A 72 7.374 -16.010 -5.293 1.00 0.00 O

ATOM 950 HG SER A 72 7.433 -15.918 -6.249 1.00 0.00 H

ATOM 951 N SER A 73 10.291 -17.972 -2.491 1.00 4.24 N

ATOM 952 H SER A 73 9.501 -18.602 -2.521 1.00 4.24 H

ATOM 953 CA SER A 73 11.611 -18.457 -2.090 1.00 4.02 C

ATOM 954 HA SER A 73 12.359 -17.904 -2.656 1.00 4.02 H

ATOM 955 C SER A 73 11.803 -19.942 -2.396 1.00 3.64 C

ATOM 956 O SER A 73 10.855 -20.724 -2.385 1.00 4.07 O

ATOM 957 CB SER A 73 11.827 -18.195 -0.594 1.00 5.86 C

ATOM 958 1HB SER A 73 12.829 -18.496 -0.298 1.00 5.86 H

ATOM 959 2HB SER A 73 11.693 -17.134 -0.391 1.00 5.86 H

ATOM 960 OG SER A 73 10.902 -18.913 0.199 1.00 8.31 O

ATOM 961 HG SER A 73 10.006 -18.664 -0.062 1.00 8.31 H

ATOM 962 N SER A 74 13.043 -20.314 -2.697 1.00 3.19 N

ATOM 963 H SER A 74 13.755 -19.598 -2.777 1.00 3.19 H

ATOM 964 CA SER A 74 13.409 -21.716 -2.889 1.00 3.31 C

ATOM 965 HA SER A 74 12.618 -22.323 -2.432 1.00 3.31 H

ATOM 966 C SER A 74 14.721 -22.028 -2.182 1.00 3.20 C

ATOM 967 O SER A 74 15.652 -21.238 -2.252 1.00 4.21 O

ATOM 968 CB SER A 74 13.523 -22.044 -4.378 1.00 0.00 C

ATOM 969 1HB SER A 74 14.320 -21.447 -4.820 1.00 0.00 H

ATOM 970 2HB SER A 74 13.768 -23.099 -4.499 1.00 0.00 H

ATOM 971 OG SER A 74 12.301 -21.770 -5.033 1.00 0.00 O

ATOM 972 HG SER A 74 12.179 -20.823 -5.056 1.00 0.00 H

ATOM 973 N ARG A 75 14.775 -23.150 -1.461 1.00 3.77 N

ATOM 974 H ARG A 75 13.974 -23.772 -1.449 1.00 3.77 H

ATOM 975 CA ARG A 75 15.978 -23.530 -0.719 1.00 3.70 C

ATOM 976 HA ARG A 75 16.843 -23.007 -1.122 1.00 3.70 H

ATOM 977 C ARG A 75 16.255 -25.025 -0.818 1.00 3.56 C

ATOM 978 O ARG A 75 15.373 -25.838 -0.580 1.00 4.14 O

ATOM 979 CB ARG A 75 15.812 -23.148 0.757 1.00 0.00 C

ATOM 980 1HB ARG A 75 14.910 -23.631 1.128 1.00 0.00 H

ATOM 981 2HB ARG A 75 16.644 -23.563 1.324 1.00 0.00 H

ATOM 982 CG ARG A 75 15.711 -21.659 1.043 1.00 0.00 C

ATOM 983 1HG ARG A 75 16.649 -21.188 0.767 1.00 0.00 H

ATOM 984 2HG ARG A 75 14.908 -21.232 0.449 1.00 0.00 H

ATOM 985 CD ARG A 75 15.417 -21.376 2.511 1.00 0.00 C

ATOM 986 1HD ARG A 75 16.186 -21.866 3.105 1.00 0.00 H

ATOM 987 2HD ARG A 75 15.487 -20.307 2.694 1.00 0.00 H

ATOM 988 NE ARG A 75 14.097 -21.854 2.910 1.00 0.00 N

ATOM 989 HE ARG A 75 14.042 -22.763 3.347 1.00 0.00 H

ATOM 990 CZ ARG A 75 12.958 -21.197 2.708 1.00 0.00 C

ATOM 991 NH1 ARG A 75 11.830 -21.764 3.080 1.00 0.00 N

ATOM 992 1HH1 ARG A 75 11.871 -22.682 3.479 1.00 0.00 H

ATOM 993 2HH1 ARG A 75 10.950 -21.265 2.958 1.00 0.00 H

ATOM 994 NH2 ARG A 75 12.906 -19.997 2.166 1.00 0.00 N

ATOM 995 1HH2 ARG A 75 13.760 -19.555 1.868 1.00 0.00 H

ATOM 996 2HH2 ARG A 75 12.014 -19.547 1.990 1.00 0.00 H

ATOM 997 N THR A 76 17.488 -25.376 -1.189 1.00 4.00 N

ATOM 998 H THR A 76 18.189 -24.665 -1.357 1.00 4.00 H

ATOM 999 CA THR A 76 17.857 -26.776 -1.372 1.00 4.05 C

ATOM 1000 HA THR A 76 17.129 -27.400 -0.851 1.00 4.05 H

ATOM 1001 C THR A 76 19.253 -27.132 -0.869 1.00 4.53 C

ATOM 1002 O THR A 76 20.071 -26.244 -0.654 1.00 5.04 O

ATOM 1003 CB THR A 76 17.811 -27.136 -2.867 1.00 4.38 C

ATOM 1004 HB THR A 76 18.139 -28.168 -2.983 1.00 4.38 H

ATOM 1005 OG1 THR A 76 18.695 -26.284 -3.596 1.00 4.76 O

ATOM 1006 HG1 THR A 76 18.499 -26.382 -4.528 1.00 4.76 H

ATOM 1007 CG2 THR A 76 16.395 -26.986 -3.410 1.00 4.70 C

ATOM 1008 1HG2 THR A 76 16.343 -27.451 -4.397 1.00 4.70 H

ATOM 1009 2HG2 THR A 76 16.132 -25.928 -3.500 1.00 4.70 H

ATOM 1010 3HG2 THR A 76 15.672 -27.480 -2.754 1.00 4.70 H

ATOM 1011 N HIS A 77 19.515 -28.426 -0.678 1.00 4.17 N

ATOM 1012 H HIS A 77 18.782 -29.111 -0.801 1.00 4.17 H

ATOM 1013 CA HIS A 77 20.829 -28.955 -0.322 1.00 5.03 C

ATOM 1014 HA HIS A 77 21.598 -28.363 -0.825 1.00 5.03 H

ATOM 1015 C HIS A 77 20.979 -30.418 -0.752 1.00 4.60 C

ATOM 1016 O HIS A 77 20.074 -31.226 -0.558 1.00 4.61 O

ATOM 1017 CB HIS A 77 21.028 -28.813 1.193 1.00 0.00 C

ATOM 1018 1HB HIS A 77 20.917 -27.762 1.459 1.00 0.00 H

ATOM 1019 2HB HIS A 77 20.252 -29.378 1.706 1.00 0.00 H

ATOM 1020 CG HIS A 77 22.346 -29.329 1.684 1.00 0.00 C

ATOM 1021 ND1 HIS A 77 22.585 -30.640 2.020 1.00 0.00 N

ATOM 1022 HD1 HIS A 77 21.921 -31.400 1.985 1.00 0.00 H

ATOM 1023 CD2 HIS A 77 23.530 -28.695 1.894 1.00 0.00 C

ATOM 1024 HD2 HIS A 77 23.715 -27.661 1.682 1.00 0.00 H

ATOM 1025 CE1 HIS A 77 23.859 -30.736 2.440 1.00 0.00 C

ATOM 1026 HE1 HIS A 77 24.316 -31.643 2.801 1.00 0.00 H

ATOM 1027 NE2 HIS A 77 24.481 -29.571 2.385 1.00 0.00 N

ATOM 1028 N GLY A 78 22.132 -30.758 -1.316 1.00 4.87 N

ATOM 1029 H GLY A 78 22.861 -30.073 -1.448 1.00 4.87 H

ATOM 1030 CA GLY A 78 22.341 -32.090 -1.859 1.00 5.13 C

ATOM 1031 1HA GLY A 78 23.329 -32.471 -1.571 1.00 5.13 H

ATOM 1032 2HA GLY A 78 21.558 -32.776 -1.512 1.00 5.13 H

ATOM 1033 C GLY A 78 22.331 -31.986 -3.371 1.00 4.98 C

ATOM 1034 O GLY A 78 23.118 -31.229 -3.937 1.00 6.31 O

ATOM 1035 N ARG A 79 21.408 -32.708 -4.011 1.00 4.82 N

ATOM 1036 H ARG A 79 20.848 -33.373 -3.493 1.00 4.82 H

ATOM 1037 CA ARG A 79 21.124 -32.574 -5.437 1.00 5.36 C

ATOM 1038 HA ARG A 79 21.669 -31.708 -5.829 1.00 5.36 H

ATOM 1039 C ARG A 79 19.627 -32.330 -5.600 1.00 4.76 C

ATOM 1040 O ARG A 79 19.004 -32.699 -6.587 1.00 5.73 O

ATOM 1041 CB ARG A 79 21.596 -33.835 -6.176 1.00 0.00 C

ATOM 1042 1HB ARG A 79 22.575 -34.108 -5.790 1.00 0.00 H

ATOM 1043 2HB ARG A 79 20.907 -34.653 -5.987 1.00 0.00 H

ATOM 1044 CG ARG A 79 21.720 -33.654 -7.677 1.00 0.00 C

ATOM 1045 1HG ARG A 79 20.794 -33.245 -8.072 1.00 0.00 H

ATOM 1046 2HG ARG A 79 22.504 -32.928 -7.876 1.00 0.00 H

ATOM 1047 CD ARG A 79 22.013 -34.980 -8.389 1.00 0.00 C

ATOM 1048 1HD ARG A 79 21.234 -35.696 -8.123 1.00 0.00 H

ATOM 1049 2HD ARG A 79 21.988 -34.797 -9.463 1.00 0.00 H

ATOM 1050 NE ARG A 79 23.341 -35.528 -8.057 1.00 0.00 N

ATOM 1051 HE ARG A 79 23.897 -35.007 -7.395 1.00 0.00 H

ATOM 1052 CZ ARG A 79 23.843 -36.667 -8.523 1.00 0.00 C

ATOM 1053 NH1 ARG A 79 25.024 -37.050 -8.106 1.00 0.00 N

ATOM 1054 1HH1 ARG A 79 25.542 -36.446 -7.487 1.00 0.00 H

ATOM 1055 2HH1 ARG A 79 25.400 -37.915 -8.422 1.00 0.00 H

ATOM 1056 NH2 ARG A 79 23.178 -37.425 -9.373 1.00 0.00 N

ATOM 1057 1HH2 ARG A 79 22.265 -37.123 -9.704 1.00 0.00 H

ATOM 1058 2HH2 ARG A 79 23.590 -38.288 -9.699 1.00 0.00 H

ATOM 1059 N ALA A 80 19.060 -31.721 -4.574 1.00 5.73 N

ATOM 1060 H ALA A 80 19.626 -31.505 -3.768 1.00 5.73 H

ATOM 1061 CA ALA A 80 17.634 -31.427 -4.490 1.00 4.82 C

ATOM 1062 HA ALA A 80 17.027 -32.306 -4.723 1.00 4.82 H

ATOM 1063 C ALA A 80 17.263 -30.328 -5.474 1.00 4.82 C

ATOM 1064 O ALA A 80 18.121 -29.606 -5.976 1.00 4.82 O

ATOM 1065 CB ALA A 80 17.310 -31.000 -3.049 1.00 0.00 C

ATOM 1066 1HB ALA A 80 17.367 -31.858 -2.373 1.00 0.00 H

ATOM 1067 2HB ALA A 80 16.299 -30.592 -3.027 1.00 0.00 H

ATOM 1068 3HB ALA A 80 18.016 -30.239 -2.711 1.00 0.00 H

ATOM 1069 N ASP A 81 15.971 -30.191 -5.783 1.00 8.01 N

ATOM 1070 H ASP A 81 15.276 -30.768 -5.329 1.00 8.01 H

ATOM 1071 CA ASP A 81 15.504 -29.205 -6.747 1.00 7.32 C

ATOM 1072 HA ASP A 81 16.249 -28.416 -6.830 1.00 7.32 H

ATOM 1073 C ASP A 81 14.169 -28.573 -6.353 1.00 6.71 C

ATOM 1074 O ASP A 81 13.148 -29.255 -6.252 1.00 7.40 O

ATOM 1075 CB ASP A 81 15.355 -29.862 -8.126 1.00 0.00 C

ATOM 1076 1HB ASP A 81 16.256 -30.435 -8.348 1.00 0.00 H

ATOM 1077 2HB ASP A 81 14.518 -30.558 -8.076 1.00 0.00 H

ATOM 1078 CG ASP A 81 15.090 -28.840 -9.218 1.00 0.00 C

ATOM 1079 OD1 ASP A 81 15.381 -27.633 -9.015 1.00 0.00 O

ATOM 1080 OD2 ASP A 81 14.599 -29.254 -10.286 1.00 0.00 O

ATOM 1081 N SER A 82 14.184 -27.265 -6.116 1.00 5.95 N

ATOM 1082 H SER A 82 15.047 -26.746 -6.231 1.00 5.95 H

ATOM 1083 CA SER A 82 12.971 -26.551 -5.727 1.00 6.66 C

ATOM 1084 HA SER A 82 12.108 -27.209 -5.854 1.00 6.66 H

ATOM 1085 C SER A 82 12.706 -25.295 -6.545 1.00 5.82 C

ATOM 1086 O SER A 82 13.626 -24.569 -6.908 1.00 7.46 O

ATOM 1087 CB SER A 82 13.043 -26.165 -4.251 1.00 0.00 C

ATOM 1088 1HB SER A 82 13.924 -25.530 -4.099 1.00 0.00 H

ATOM 1089 2HB SER A 82 12.134 -25.612 -3.977 1.00 0.00 H

ATOM 1090 OG SER A 82 13.119 -27.317 -3.432 1.00 0.00 O

ATOM 1091 HG SER A 82 13.850 -27.848 -3.760 1.00 0.00 H

ATOM 1092 N THR A 83 11.433 -25.060 -6.837 1.00 5.49 N

ATOM 1093 H THR A 83 10.734 -25.695 -6.475 1.00 5.49 H

ATOM 1094 CA THR A 83 11.002 -23.936 -7.662 1.00 6.16 C

ATOM 1095 HA THR A 83 11.824 -23.223 -7.738 1.00 6.16 H

ATOM 1096 C THR A 83 9.793 -23.253 -7.043 1.00 5.28 C

ATOM 1097 O THR A 83 8.831 -23.925 -6.669 1.00 8.78 O

ATOM 1098 CB THR A 83 10.613 -24.399 -9.078 1.00 0.00 C

ATOM 1099 HB THR A 83 9.753 -25.068 -9.007 1.00 0.00 H

ATOM 1100 OG1 THR A 83 11.708 -25.114 -9.661 1.00 0.00 O

ATOM 1101 HG1 THR A 83 11.366 -25.650 -10.387 1.00 0.00 H

ATOM 1102 CG2 THR A 83 10.285 -23.249 -10.037 1.00 0.00 C

ATOM 1103 1HG2 THR A 83 10.055 -23.671 -11.017 1.00 0.00 H

ATOM 1104 2HG2 THR A 83 11.132 -22.561 -10.103 1.00 0.00 H

ATOM 1105 3HG2 THR A 83 9.390 -22.735 -9.679 1.00 0.00 H

ATOM 1106 N ALA A 84 9.825 -21.930 -6.963 1.00 4.67 N

ATOM 1107 H ALA A 84 10.652 -21.411 -7.234 1.00 4.67 H

ATOM 1108 CA ALA A 84 8.654 -21.202 -6.466 1.00 4.90 C

ATOM 1109 HA ALA A 84 7.782 -21.868 -6.489 1.00 4.90 H

ATOM 1110 C ALA A 84 8.320 -19.982 -7.321 1.00 4.49 C

ATOM 1111 O ALA A 84 9.199 -19.319 -7.858 1.00 6.75 O

ATOM 1112 CB ALA A 84 8.894 -20.766 -5.032 1.00 5.85 C

ATOM 1113 1HB ALA A 84 9.116 -21.654 -4.440 1.00 5.85 H

ATOM 1114 2HB ALA A 84 8.001 -20.275 -4.643 1.00 5.85 H

ATOM 1115 3HB ALA A 84 9.751 -20.092 -5.000 1.00 5.85 H

ATOM 1116 N SER A 85 7.033 -19.687 -7.445 1.00 4.38 N

ATOM 1117 H SER A 85 6.360 -20.237 -6.927 1.00 4.38 H

ATOM 1118 CA SER A 85 6.548 -18.595 -8.278 1.00 4.55 C

ATOM 1119 HA SER A 85 7.316 -17.818 -8.348 1.00 4.55 H

ATOM 1120 C SER A 85 5.271 -17.999 -7.691 1.00 4.01 C

ATOM 1121 O SER A 85 4.317 -18.718 -7.376 1.00 4.76 O

ATOM 1122 CB SER A 85 6.265 -19.132 -9.680 1.00 0.00 C

ATOM 1123 1HB SER A 85 7.142 -19.660 -10.043 1.00 0.00 H

ATOM 1124 2HB SER A 85 5.432 -19.825 -9.635 1.00 0.00 H

ATOM 1125 OG SER A 85 5.948 -18.092 -10.587 1.00 0.00 O

ATOM 1126 HG SER A 85 6.784 -17.740 -10.892 1.00 0.00 H

ATOM 1127 N ALA A 86 5.244 -16.678 -7.554 1.00 4.01 N

ATOM 1128 H ALA A 86 6.013 -16.107 -7.878 1.00 4.01 H

ATOM 1129 CA ALA A 86 4.104 -16.003 -6.931 1.00 4.72 C

ATOM 1130 HA ALA A 86 3.229 -16.643 -7.022 1.00 4.72 H

ATOM 1131 C ALA A 86 3.803 -14.696 -7.656 1.00 4.13 C

ATOM 1132 O ALA A 86 4.705 -14.000 -8.111 1.00 5.18 O

ATOM 1133 CB ALA A 86 4.346 -15.773 -5.443 1.00 5.59 C

ATOM 1134 1HB ALA A 86 4.584 -16.725 -4.968 1.00 5.59 H

ATOM 1135 2HB ALA A 86 3.458 -15.356 -4.966 1.00 5.59 H

ATOM 1136 3HB ALA A 86 5.179 -15.078 -5.320 1.00 5.59 H

ATOM 1137 N LYS A 87 2.519 -14.395 -7.812 1.00 4.82 N

ATOM 1138 H LYS A 87 1.817 -15.004 -7.409 1.00 4.82 H

ATOM 1139 CA LYS A 87 2.040 -13.222 -8.545 1.00 5.37 C

ATOM 1140 HA LYS A 87 2.878 -12.539 -8.668 1.00 5.37 H

ATOM 1141 C LYS A 87 0.939 -12.470 -7.800 1.00 5.36 C

ATOM 1142 O LYS A 87 0.070 -13.062 -7.162 1.00 6.16 O

ATOM 1143 CB LYS A 87 1.524 -13.652 -9.924 1.00 0.00 C

ATOM 1144 1HB LYS A 87 0.672 -14.330 -9.795 1.00 0.00 H

ATOM 1145 2HB LYS A 87 1.212 -12.744 -10.452 1.00 0.00 H

ATOM 1146 CG LYS A 87 2.560 -14.364 -10.769 1.00 0.00 C

ATOM 1147 1HG LYS A 87 3.424 -13.720 -10.903 1.00 0.00 H

ATOM 1148 2HG LYS A 87 2.861 -15.283 -10.273 1.00 0.00 H

ATOM 1149 CD LYS A 87 2.021 -14.752 -12.144 1.00 0.00 C

ATOM 1150 1HD LYS A 87 1.099 -15.316 -12.026 1.00 0.00 H

ATOM 1151 2HD LYS A 87 1.842 -13.854 -12.729 1.00 0.00 H

ATOM 1152 CE LYS A 87 3.053 -15.616 -12.869 1.00 0.00 C

ATOM 1153 1HE LYS A 87 3.961 -15.018 -13.009 1.00 0.00 H

ATOM 1154 2HE LYS A 87 3.241 -16.508 -12.257 1.00 0.00 H

ATOM 1155 NZ LYS A 87 2.515 -16.054 -14.200 1.00 0.00 N

ATOM 1156 1HZ LYS A 87 3.224 -16.559 -14.705 1.00 0.00 H

ATOM 1157 2HZ LYS A 87 2.227 -15.255 -14.750 1.00 0.00 H

ATOM 1158 3HZ LYS A 87 1.736 -16.667 -14.076 1.00 0.00 H

ATOM 1159 N GLY A 88 0.979 -11.141 -7.898 1.00 5.68 N

ATOM 1160 H GLY A 88 1.723 -10.679 -8.407 1.00 5.68 H

ATOM 1161 CA GLY A 88 0.013 -10.320 -7.182 1.00 5.95 C

ATOM 1162 1HA GLY A 88 -0.242 -9.434 -7.759 1.00 5.95 H

ATOM 1163 2HA GLY A 88 -0.892 -10.886 -6.974 1.00 5.95 H

ATOM 1164 C GLY A 88 0.618 -9.862 -5.863 1.00 5.58 C

ATOM 1165 O GLY A 88 1.448 -8.956 -5.834 1.00 6.00 O

ATOM 1166 N GLU A 89 0.215 -10.526 -4.782 1.00 6.18 N

ATOM 1167 H GLU A 89 -0.502 -11.234 -4.872 1.00 6.18 H

ATOM 1168 CA GLU A 89 0.783 -10.334 -3.454 1.00 6.09 C

ATOM 1169 HA GLU A 89 1.818 -10.007 -3.544 1.00 6.09 H

ATOM 1170 C GLU A 89 0.747 -11.705 -2.778 1.00 6.05 C

ATOM 1171 O GLU A 89 0.534 -11.846 -1.575 1.00 7.67 O

ATOM 1172 CB GLU A 89 0.002 -9.262 -2.695 1.00 0.00 C

ATOM 1173 1HB GLU A 89 -0.090 -8.403 -3.362 1.00 0.00 H

ATOM 1174 2HB GLU A 89 -0.993 -9.638 -2.462 1.00 0.00 H

ATOM 1175 CG GLU A 89 0.661 -8.770 -1.416 1.00 0.00 C

ATOM 1176 1HG GLU A 89 0.728 -9.580 -0.686 1.00 0.00 H

ATOM 1177 2HG GLU A 89 1.659 -8.406 -1.669 1.00 0.00 H

ATOM 1178 CD GLU A 89 -0.144 -7.647 -0.770 1.00 0.00 C

ATOM 1179 OE1 GLU A 89 -0.236 -6.574 -1.401 1.00 0.00 O

ATOM 1180 OE2 GLU A 89 -0.653 -7.839 0.353 1.00 0.00 O

ATOM 1181 N ALA A 90 0.937 -12.737 -3.590 1.00 5.99 N

ATOM 1182 H ALA A 90 1.109 -12.562 -4.572 1.00 5.99 H

ATOM 1183 CA ALA A 90 0.881 -14.126 -3.137 1.00 5.30 C

ATOM 1184 HA ALA A 90 0.120 -14.229 -2.367 1.00 5.30 H

ATOM 1185 C ALA A 90 2.216 -14.577 -2.541 1.00 5.13 C

ATOM 1186 O ALA A 90 3.237 -13.916 -2.731 1.00 6.92 O

ATOM 1187 CB ALA A 90 0.497 -15.029 -4.318 1.00 5.69 C

ATOM 1188 1HB ALA A 90 -0.503 -14.756 -4.639 1.00 5.69 H

ATOM 1189 2HB ALA A 90 0.517 -16.059 -3.978 1.00 5.69 H

ATOM 1190 3HB ALA A 90 1.191 -14.896 -5.141 1.00 5.69 H

ATOM 1191 N MET A 91 2.197 -15.704 -1.848 1.00 4.72 N

ATOM 1192 H MET A 91 1.320 -16.203 -1.754 1.00 4.72 H

ATOM 1193 CA MET A 91 3.371 -16.298 -1.233 1.00 5.29 C

ATOM 1194 HA MET A 91 4.249 -15.721 -1.538 1.00 5.29 H

ATOM 1195 C MET A 91 3.529 -17.732 -1.736 1.00 4.60 C

ATOM 1196 O MET A 91 2.607 -18.536 -1.643 1.00 4.98 O

ATOM 1197 CB MET A 91 3.258 -16.287 0.293 1.00 6.39 C

ATOM 1198 1HB MET A 91 3.176 -15.258 0.638 1.00 6.39 H

ATOM 1199 2HB MET A 91 2.344 -16.807 0.576 1.00 6.39 H

ATOM 1200 CG MET A 91 4.432 -16.978 1.001 1.00 8.61 C

ATOM 1201 1HG MET A 91 4.159 -17.118 2.041 1.00 8.61 H

ATOM 1202 2HG MET A 91 4.596 -17.964 0.581 1.00 8.61 H

ATOM 1203 SD MET A 91 5.973 -16.036 0.856 1.00 11.04 S

ATOM 1204 CE MET A 91 5.719 -14.704 2.047 1.00 8.94 C

ATOM 1205 1HE MET A 91 6.572 -14.022 1.995 1.00 8.94 H

ATOM 1206 2HE MET A 91 5.663 -15.124 3.054 1.00 8.94 H

ATOM 1207 3HE MET A 91 4.778 -14.190 1.837 1.00 8.94 H

ATOM 1208 N ALA A 92 4.698 -18.062 -2.270 1.00 3.90 N

ATOM 1209 H ALA A 92 5.432 -17.382 -2.388 1.00 3.90 H

ATOM 1210 CA ALA A 92 4.968 -19.441 -2.675 1.00 3.55 C

ATOM 1211 HA ALA A 92 4.295 -20.095 -2.127 1.00 3.55 H

ATOM 1212 C ALA A 92 6.398 -19.834 -2.312 1.00 3.90 C

ATOM 1213 O ALA A 92 7.334 -19.052 -2.507 1.00 5.01 O

ATOM 1214 CB ALA A 92 4.706 -19.647 -4.165 1.00 4.55 C

ATOM 1215 1HB ALA A 92 3.693 -19.354 -4.430 1.00 4.55 H

ATOM 1216 2HB ALA A 92 4.842 -20.702 -4.395 1.00 4.55 H

ATOM 1217 3HB ALA A 92 5.426 -19.082 -4.754 1.00 4.55 H

ATOM 1218 N GLU A 93 6.614 -21.018 -1.763 1.00 4.02 N

ATOM 1219 H GLU A 93 5.840 -21.632 -1.545 1.00 4.02 H

ATOM 1220 CA GLU A 93 7.963 -21.472 -1.431 1.00 3.79 C

ATOM 1221 HA GLU A 93 8.644 -21.056 -2.170 1.00 3.79 H

ATOM 1222 C GLU A 93 8.133 -22.986 -1.530 1.00 4.33 C

ATOM 1223 O GLU A 93 7.168 -23.739 -1.351 1.00 4.84 O

ATOM 1224 CB GLU A 93 8.384 -20.949 -0.062 1.00 0.00 C

ATOM 1225 1HB GLU A 93 9.451 -21.106 0.044 1.00 0.00 H

ATOM 1226 2HB GLU A 93 8.188 -19.883 -0.034 1.00 0.00 H

ATOM 1227 CG GLU A 93 7.695 -21.612 1.128 1.00 0.00 C

ATOM 1228 1HG GLU A 93 6.613 -21.501 1.053 1.00 0.00 H

ATOM 1229 2HG GLU A 93 7.942 -22.674 1.117 1.00 0.00 H

ATOM 1230 CD GLU A 93 8.121 -21.020 2.460 1.00 0.00 C

ATOM 1231 OE1 GLU A 93 9.147 -20.312 2.506 1.00 0.00 O

ATOM 1232 OE2 GLU A 93 7.423 -21.238 3.470 1.00 0.00 O

ATOM 1233 N GLY A 94 9.357 -23.407 -1.827 1.00 4.96 N

ATOM 1234 H GLY A 94 10.112 -22.740 -1.905 1.00 4.96 H

ATOM 1235 CA GLY A 94 9.688 -24.814 -1.920 1.00 4.50 C

ATOM 1236 1HA GLY A 94 8.925 -25.417 -1.431 1.00 4.50 H

ATOM 1237 2HA GLY A 94 9.718 -25.066 -2.978 1.00 4.50 H

ATOM 1238 C GLY A 94 11.028 -25.109 -1.285 1.00 4.64 C

ATOM 1239 O GLY A 94 12.034 -24.480 -1.614 1.00 4.96 O

ATOM 1240 N THR A 95 11.055 -26.055 -0.358 1.00 4.35 N

ATOM 1241 H THR A 95 10.221 -26.566 -0.104 1.00 4.35 H

ATOM 1242 CA THR A 95 12.284 -26.423 0.326 1.00 4.02 C

ATOM 1243 HA THR A 95 13.130 -25.960 -0.184 1.00 4.02 H

ATOM 1244 C THR A 95 12.465 -27.929 0.264 1.00 4.55 C

ATOM 1245 O THR A 95 11.546 -28.680 0.622 1.00 4.35 O

ATOM 1246 CB THR A 95 12.271 -25.957 1.787 1.00 5.12 C

ATOM 1247 HB THR A 95 11.502 -26.478 2.344 1.00 5.12 H

ATOM 1248 OG1 THR A 95 12.012 -24.546 1.828 1.00 5.51 O

ATOM 1249 HG1 THR A 95 11.276 -24.360 1.251 1.00 5.51 H

ATOM 1250 CG2 THR A 95 13.618 -26.230 2.489 1.00 5.97 C

ATOM 1251 1HG2 THR A 95 13.709 -25.538 3.327 1.00 5.97 H

ATOM 1252 2HG2 THR A 95 14.453 -26.090 1.798 1.00 5.97 H

ATOM 1253 3HG2 THR A 95 13.597 -27.254 2.865 1.00 5.97 H

ATOM 1254 N SER A 96 13.644 -28.361 -0.172 1.00 4.63 N

ATOM 1255 H SER A 96 14.354 -27.714 -0.487 1.00 4.63 H

ATOM 1256 CA SER A 96 13.934 -29.790 -0.233 1.00 4.58 C

ATOM 1257 HA SER A 96 13.353 -30.255 0.569 1.00 4.58 H

ATOM 1258 C SER A 96 15.395 -30.153 0.007 1.00 5.05 C

ATOM 1259 O SER A 96 16.318 -29.384 -0.275 1.00 6.22 O

ATOM 1260 CB SER A 96 13.492 -30.382 -1.573 1.00 5.69 C

ATOM 1261 1HB SER A 96 13.664 -31.462 -1.570 1.00 5.69 H

ATOM 1262 2HB SER A 96 12.426 -30.201 -1.725 1.00 5.69 H

ATOM 1263 OG SER A 96 14.212 -29.802 -2.649 1.00 7.12 O

ATOM 1264 HG SER A 96 14.300 -30.469 -3.342 1.00 7.12 H

ATOM 1265 N ASP A 97 15.605 -31.333 0.582 1.00 6.11 N

ATOM 1266 H ASP A 97 14.822 -31.938 0.791 1.00 6.11 H

ATOM 1267 CA ASP A 97 16.942 -31.794 0.947 1.00 7.09 C

ATOM 1268 HA ASP A 97 17.664 -31.162 0.426 1.00 7.09 H

ATOM 1269 C ASP A 97 17.154 -33.238 0.499 1.00 7.13 C

ATOM 1270 O ASP A 97 16.255 -34.078 0.617 1.00 6.93 O

ATOM 1271 CB ASP A 97 17.105 -31.676 2.465 1.00 0.00 C

ATOM 1272 1HB ASP A 97 16.319 -32.228 2.981 1.00 0.00 H

ATOM 1273 2HB ASP A 97 18.054 -32.119 2.764 1.00 0.00 H

ATOM 1274 CG ASP A 97 17.088 -30.238 2.929 1.00 0.00 C

ATOM 1275 OD1 ASP A 97 17.821 -29.424 2.349 1.00 0.00 O

ATOM 1276 OD2 ASP A 97 16.364 -29.904 3.898 1.00 0.00 O

ATOM 1277 N GLY A 98 18.347 -33.499 -0.029 1.00 8.52 N

ATOM 1278 H GLY A 98 19.056 -32.783 -0.085 1.00 8.52 H

ATOM 1279 CA GLY A 98 18.682 -34.820 -0.546 1.00 9.47 C

ATOM 1280 1HA GLY A 98 19.659 -35.123 -0.205 1.00 9.47 H

ATOM 1281 2HA GLY A 98 17.953 -35.539 -0.197 1.00 9.47 H

ATOM 1282 C GLY A 98 18.684 -34.846 -2.060 1.00 8.75 C

ATOM 1283 O GLY A 98 19.414 -34.098 -2.712 1.00 11.96 O

ATOM 1284 N ASP A 99 17.823 -35.686 -2.631 1.00 9.12 N

ATOM 1285 H ASP A 99 17.282 -36.297 -2.031 1.00 9.12 H

ATOM 1286 CA ASP A 99 17.491 -35.668 -4.054 1.00 9.05 C

ATOM 1287 HA ASP A 99 18.153 -34.974 -4.576 1.00 9.05 H

ATOM 1288 C ASP A 99 16.047 -35.198 -4.212 1.00 6.82 C

ATOM 1289 O ASP A 99 15.484 -35.192 -5.308 1.00 8.59 O

ATOM 1290 CB ASP A 99 17.602 -37.067 -4.657 1.00 0.00 C

ATOM 1291 1HB ASP A 99 16.999 -37.753 -4.065 1.00 0.00 H

ATOM 1292 2HB ASP A 99 17.230 -37.054 -5.678 1.00 0.00 H

ATOM 1293 CG ASP A 99 19.037 -37.550 -4.705 1.00 0.00 C

ATOM 1294 OD1 ASP A 99 19.923 -36.791 -5.155 1.00 0.00 O

ATOM 1295 OD2 ASP A 99 19.293 -38.717 -4.319 1.00 0.00 O

ATOM 1296 N ALA A 100 15.439 -34.832 -3.100 1.00 5.73 N

ATOM 1297 H ALA A 100 15.981 -34.823 -2.248 1.00 5.73 H

ATOM 1298 CA ALA A 100 14.019 -34.523 -3.014 1.00 6.38 C

ATOM 1299 HA ALA A 100 13.484 -35.353 -3.481 1.00 6.38 H

ATOM 1300 C ALA A 100 13.709 -33.229 -3.743 1.00 6.39 C

ATOM 1301 O ALA A 100 14.587 -32.399 -3.965 1.00 7.96 O

ATOM 1302 CB ALA A 100 13.633 -34.413 -1.545 1.00 0.00 C

ATOM 1303 1HB ALA A 100 13.772 -35.371 -1.054 1.00 0.00 H

ATOM 1304 2HB ALA A 100 12.589 -34.122 -1.487 1.00 0.00 H

ATOM 1305 3HB ALA A 100 14.244 -33.657 -1.058 1.00 0.00 H

ATOM 1306 N LYS A 101 12.442 -33.071 -4.118 1.00 5.91 N

ATOM 1307 H LYS A 101 11.776 -33.792 -3.888 1.00 5.91 H

ATOM 1308 CA LYS A 101 11.992 -31.905 -4.869 1.00 6.38 C

ATOM 1309 HA LYS A 101 12.763 -31.142 -4.831 1.00 6.38 H

ATOM 1310 C LYS A 101 10.737 -31.288 -4.262 1.00 5.07 C

ATOM 1311 O LYS A 101 9.854 -32.009 -3.814 1.00 6.00 O

ATOM 1312 CB LYS A 101 11.790 -32.285 -6.336 1.00 0.00 C

ATOM 1313 1HB LYS A 101 10.948 -32.974 -6.409 1.00 0.00 H

ATOM 1314 2HB LYS A 101 11.573 -31.373 -6.897 1.00 0.00 H

ATOM 1315 CG LYS A 101 13.015 -32.931 -6.945 1.00 0.00 C

ATOM 1316 1HG LYS A 101 13.902 -32.341 -6.738 1.00 0.00 H

ATOM 1317 2HG LYS A 101 13.140 -33.906 -6.485 1.00 0.00 H

ATOM 1318 CD LYS A 101 12.897 -33.098 -8.456 1.00 0.00 C

ATOM 1319 1HD LYS A 101 11.997 -33.671 -8.670 1.00 0.00 H

ATOM 1320 2HD LYS A 101 12.812 -32.127 -8.939 1.00 0.00 H

ATOM 1321 CE LYS A 101 14.107 -33.817 -9.017 1.00 0.00 C

ATOM 1322 1HE LYS A 101 15.006 -33.254 -8.721 1.00 0.00 H

ATOM 1323 2HE LYS A 101 14.182 -34.822 -8.572 1.00 0.00 H

ATOM 1324 NZ LYS A 101 13.990 -33.939 -10.510 1.00 0.00 N

ATOM 1325 1HZ LYS A 101 14.880 -34.207 -10.892 1.00 0.00 H

ATOM 1326 2HZ LYS A 101 13.706 -33.056 -10.923 1.00 0.00 H

ATOM 1327 3HZ LYS A 101 13.282 -34.619 -10.753 1.00 0.00 H

ATOM 1328 N SER A 102 10.671 -29.963 -4.227 1.00 4.36 N

ATOM 1329 H SER A 102 11.411 -29.418 -4.645 1.00 4.36 H

ATOM 1330 CA SER A 102 9.528 -29.257 -3.655 1.00 4.85 C

ATOM 1331 HA SER A 102 8.649 -29.900 -3.646 1.00 4.85 H

ATOM 1332 C SER A 102 9.225 -27.990 -4.450 1.00 4.00 C

ATOM 1333 O SER A 102 10.026 -27.055 -4.490 1.00 6.02 O

ATOM 1334 CB SER A 102 9.862 -28.831 -2.220 1.00 4.79 C

ATOM 1335 1HB SER A 102 10.757 -28.211 -2.256 1.00 4.79 H

ATOM 1336 2HB SER A 102 9.040 -28.269 -1.782 1.00 4.79 H

ATOM 1337 OG SER A 102 10.086 -29.952 -1.376 1.00 5.43 O

ATOM 1338 HG SER A 102 10.552 -29.650 -0.595 1.00 5.43 H

ATOM 1339 N TYR A 103 8.068 -27.953 -5.100 1.00 4.22 N

ATOM 1340 H TYR A 103 7.409 -28.717 -5.033 1.00 4.22 H

ATOM 1341 CA TYR A 103 7.736 -26.807 -5.938 1.00 4.40 C

ATOM 1342 HA TYR A 103 8.369 -25.977 -5.629 1.00 4.40 H

ATOM 1343 C TYR A 103 6.296 -26.349 -5.734 1.00 4.25 C

ATOM 1344 O TYR A 103 5.383 -27.140 -5.508 1.00 4.58 O

ATOM 1345 CB TYR A 103 8.038 -27.099 -7.414 1.00 0.00 C

ATOM 1346 1HB TYR A 103 7.649 -26.274 -8.006 1.00 0.00 H

ATOM 1347 2HB TYR A 103 9.116 -27.128 -7.548 1.00 0.00 H

ATOM 1348 CG TYR A 103 7.466 -28.406 -7.926 1.00 0.00 C

ATOM 1349 CD1 TYR A 103 6.218 -28.443 -8.550 1.00 0.00 C

ATOM 1350 HD1 TYR A 103 5.616 -27.548 -8.629 1.00 0.00 H

ATOM 1351 CD2 TYR A 103 8.184 -29.603 -7.812 1.00 0.00 C

ATOM 1352 HD2 TYR A 103 9.125 -29.621 -7.294 1.00 0.00 H

ATOM 1353 CE1 TYR A 103 5.723 -29.630 -9.099 1.00 0.00 C

ATOM 1354 HE1 TYR A 103 4.779 -29.647 -9.612 1.00 0.00 H

ATOM 1355 CE2 TYR A 103 7.679 -30.791 -8.313 1.00 0.00 C

ATOM 1356 HE2 TYR A 103 8.229 -31.715 -8.208 1.00 0.00 H

ATOM 1357 CZ TYR A 103 6.471 -30.791 -8.977 1.00 0.00 C

ATOM 1358 OH TYR A 103 5.984 -31.952 -9.529 1.00 0.00 O

ATOM 1359 HH TYR A 103 5.162 -31.787 -10.007 1.00 0.00 H

ATOM 1360 N ALA A 104 6.124 -25.033 -5.764 1.00 4.22 N

ATOM 1361 H ALA A 104 6.894 -24.433 -6.031 1.00 4.22 H

ATOM 1362 CA ALA A 104 4.873 -24.389 -5.384 1.00 3.91 C

ATOM 1363 HA ALA A 104 4.044 -25.079 -5.506 1.00 3.91 H

ATOM 1364 C ALA A 104 4.610 -23.157 -6.243 1.00 4.09 C

ATOM 1365 O ALA A 104 5.541 -22.432 -6.610 1.00 5.06 O

ATOM 1366 CB ALA A 104 4.962 -23.981 -3.920 1.00 4.40 C

ATOM 1367 1HB ALA A 104 5.138 -24.856 -3.293 1.00 4.40 H

ATOM 1368 2HB ALA A 104 4.039 -23.491 -3.617 1.00 4.40 H

ATOM 1369 3HB ALA A 104 5.787 -23.284 -3.777 1.00 4.40 H

ATOM 1370 N SER A 105 3.345 -22.901 -6.565 1.00 3.81 N

ATOM 1371 H SER A 105 2.609 -23.520 -6.245 1.00 3.81 H

ATOM 1372 CA SER A 105 2.985 -21.695 -7.309 1.00 4.32 C

ATOM 1373 HA SER A 105 3.783 -20.966 -7.218 1.00 4.32 H

ATOM 1374 C SER A 105 1.700 -21.084 -6.748 1.00 3.59 C

ATOM 1375 O SER A 105 0.830 -21.824 -6.285 1.00 5.04 O

ATOM 1376 CB SER A 105 2.814 -22.036 -8.786 1.00 0.00 C

ATOM 1377 1HB SER A 105 1.974 -22.713 -8.893 1.00 0.00 H

ATOM 1378 2HB SER A 105 2.623 -21.128 -9.350 1.00 0.00 H

ATOM 1379 OG SER A 105 3.987 -22.648 -9.280 1.00 0.00 O

ATOM 1380 HG SER A 105 4.586 -22.750 -8.542 1.00 0.00 H

ATOM 1381 N ALA A 106 1.581 -19.766 -6.771 1.00 3.81 N

ATOM 1382 H ALA A 106 2.309 -19.199 -7.183 1.00 3.81 H

ATOM 1383 CA ALA A 106 0.412 -19.090 -6.216 1.00 4.29 C

ATOM 1384 HA ALA A 106 -0.462 -19.716 -6.395 1.00 4.29 H

ATOM 1385 C ALA A 106 0.155 -17.745 -6.882 1.00 3.95 C

ATOM 1386 O ALA A 106 1.060 -17.127 -7.440 1.00 5.13 O

ATOM 1387 CB ALA A 106 0.586 -18.903 -4.708 1.00 4.76 C

ATOM 1388 1HB ALA A 106 0.739 -19.884 -4.263 1.00 4.76 H

ATOM 1389 2HB ALA A 106 -0.290 -18.439 -4.260 1.00 4.76 H

ATOM 1390 3HB ALA A 106 1.461 -18.284 -4.521 1.00 4.76 H

ATOM 1391 N ASP A 107 -1.095 -17.304 -6.846 1.00 4.04 N

ATOM 1392 H ASP A 107 -1.819 -17.846 -6.392 1.00 4.04 H

ATOM 1393 CA ASP A 107 -1.465 -15.986 -7.366 1.00 4.82 C

ATOM 1394 HA ASP A 107 -0.627 -15.300 -7.256 1.00 4.82 H

ATOM 1395 C ASP A 107 -2.654 -15.390 -6.609 1.00 5.03 C

ATOM 1396 O ASP A 107 -3.475 -16.125 -6.064 1.00 5.88 O

ATOM 1397 CB ASP A 107 -1.791 -16.077 -8.859 1.00 0.00 C

ATOM 1398 1HB ASP A 107 -1.894 -15.080 -9.270 1.00 0.00 H

ATOM 1399 2HB ASP A 107 -0.963 -16.569 -9.359 1.00 0.00 H

ATOM 1400 CG ASP A 107 -3.085 -16.845 -9.125 1.00 0.00 C

ATOM 1401 OD1 ASP A 107 -3.024 -18.032 -9.534 1.00 0.00 O

ATOM 1402 OD2 ASP A 107 -4.174 -16.268 -8.960 1.00 0.00 O

ATOM 1403 N GLY A 108 -2.713 -14.065 -6.551 1.00 5.74 N

ATOM 1404 H GLY A 108 -2.026 -13.502 -7.028 1.00 5.74 H

ATOM 1405 CA GLY A 108 -3.708 -13.374 -5.756 1.00 6.29 C

ATOM 1406 1HA GLY A 108 -4.100 -12.488 -6.273 1.00 6.29 H

ATOM 1407 2HA GLY A 108 -4.531 -14.061 -5.521 1.00 6.29 H

ATOM 1408 C GLY A 108 -3.052 -12.954 -4.455 1.00 6.04 C

ATOM 1409 O GLY A 108 -2.039 -12.257 -4.442 1.00 7.17 O

ATOM 1410 N ASN A 109 -3.616 -13.398 -3.336 1.00 7.24 N

ATOM 1411 H ASN A 109 -4.476 -13.927 -3.378 1.00 7.24 H

ATOM 1412 CA ASN A 109 -2.987 -13.260 -2.026 1.00 7.44 C

ATOM 1413 HA ASN A 109 -2.050 -12.698 -2.112 1.00 7.44 H

ATOM 1414 C ASN A 109 -2.702 -14.648 -1.452 1.00 7.78 C

ATOM 1415 O ASN A 109 -2.340 -14.796 -0.285 1.00 9.08 O

ATOM 1416 CB ASN A 109 -3.935 -12.506 -1.090 1.00 0.00 C

ATOM 1417 1HB ASN A 109 -4.856 -13.078 -0.986 1.00 0.00 H

ATOM 1418 2HB ASN A 109 -3.466 -12.406 -0.108 1.00 0.00 H

ATOM 1419 CG ASN A 109 -4.274 -11.120 -1.621 1.00 0.00 C

ATOM 1420 OD1 ASN A 109 -3.397 -10.297 -1.867 1.00 0.00 O

ATOM 1421 ND2 ASN A 109 -5.558 -10.864 -1.812 1.00 0.00 N

ATOM 1422 1HD2 ASN A 109 -5.818 -9.954 -2.158 1.00 0.00 H

ATOM 1423 2HD2 ASN A 109 -6.244 -11.582 -1.619 1.00 0.00 H

ATOM 1424 N ALA A 110 -2.915 -15.675 -2.265 1.00 5.09 N

ATOM 1425 H ALA A 110 -3.138 -15.495 -3.233 1.00 5.09 H

ATOM 1426 CA ALA A 110 -2.838 -17.071 -1.845 1.00 4.61 C

ATOM 1427 HA ALA A 110 -3.543 -17.238 -1.035 1.00 4.61 H

ATOM 1428 C ALA A 110 -1.453 -17.447 -1.349 1.00 4.04 C

ATOM 1429 O ALA A 110 -0.443 -16.855 -1.727 1.00 5.46 O

ATOM 1430 CB ALA A 110 -3.177 -17.973 -3.010 1.00 5.38 C

ATOM 1431 1HB ALA A 110 -4.221 -17.813 -3.288 1.00 5.38 H

ATOM 1432 2HB ALA A 110 -3.041 -19.027 -2.759 1.00 5.38 H

ATOM 1433 3HB ALA A 110 -2.556 -17.742 -3.882 1.00 5.38 H

ATOM 1434 N CYS A 111 -1.397 -18.476 -0.512 1.00 4.43 N

ATOM 1435 H CYS A 111 -2.246 -18.944 -0.223 1.00 4.43 H

ATOM 1436 CA CYS A 111 -0.134 -18.932 0.051 1.00 4.42 C

ATOM 1437 HA CYS A 111 0.689 -18.400 -0.430 1.00 4.42 H

ATOM 1438 C CYS A 111 0.083 -20.424 -0.113 1.00 4.05 C

ATOM 1439 O CYS A 111 -0.812 -21.203 0.220 1.00 4.74 O

ATOM 1440 CB CYS A 111 -0.130 -18.611 1.558 1.00 0.00 C

ATOM 1441 1HB CYS A 111 -0.994 -19.108 2.003 1.00 0.00 H

ATOM 1442 2HB CYS A 111 0.785 -18.996 2.012 1.00 0.00 H

ATOM 1443 SG CYS A 111 -0.245 -16.848 1.964 1.00 0.00 S

ATOM 1444 HG CYS A 111 -1.065 -16.491 0.989 1.00 0.00 H

ATOM 1445 N ALA A 112 1.247 -20.848 -0.607 1.00 3.48 N

ATOM 1446 H ALA A 112 1.971 -20.190 -0.852 1.00 3.48 H

ATOM 1447 CA ALA A 112 1.542 -22.257 -0.827 1.00 3.86 C

ATOM 1448 HA ALA A 112 0.824 -22.828 -0.244 1.00 3.86 H

ATOM 1449 C ALA A 112 2.952 -22.610 -0.364 1.00 3.97 C

ATOM 1450 O ALA A 112 3.880 -21.807 -0.496 1.00 4.44 O

ATOM 1451 CB ALA A 112 1.365 -22.592 -2.306 1.00 4.72 C

ATOM 1452 1HB ALA A 112 0.412 -22.198 -2.648 1.00 4.72 H

ATOM 1453 2HB ALA A 112 1.419 -23.666 -2.461 1.00 4.72 H

ATOM 1454 3HB ALA A 112 2.157 -22.122 -2.881 1.00 4.72 H

ATOM 1455 N LYS A 113 3.099 -23.802 0.213 1.00 3.86 N

ATOM 1456 H LYS A 113 2.293 -24.406 0.323 1.00 3.86 H

ATOM 1457 CA LYS A 113 4.383 -24.276 0.707 1.00 3.61 C

ATOM 1458 HA LYS A 113 5.166 -23.731 0.183 1.00 3.61 H

ATOM 1459 C LYS A 113 4.634 -25.770 0.466 1.00 3.76 C

ATOM 1460 O LYS A 113 3.910 -26.628 0.987 1.00 4.35 O

ATOM 1461 CB LYS A 113 4.497 -23.971 2.200 1.00 0.00 C

ATOM 1462 1HB LYS A 113 4.400 -22.898 2.364 1.00 0.00 H

ATOM 1463 2HB LYS A 113 3.677 -24.461 2.724 1.00 0.00 H

ATOM 1464 CG LYS A 113 5.809 -24.461 2.803 1.00 0.00 C

ATOM 1465 1HG LYS A 113 5.884 -25.540 2.638 1.00 0.00 H

ATOM 1466 2HG LYS A 113 6.658 -23.998 2.292 1.00 0.00 H

ATOM 1467 CD LYS A 113 5.894 -24.195 4.295 1.00 0.00 C

ATOM 1468 1HD LYS A 113 5.718 -23.137 4.473 1.00 0.00 H

ATOM 1469 2HD LYS A 113 5.136 -24.788 4.803 1.00 0.00 H

ATOM 1470 CE LYS A 113 7.262 -24.584 4.846 1.00 0.00 C

ATOM 1471 1HE LYS A 113 7.481 -25.605 4.523 1.00 0.00 H

ATOM 1472 2HE LYS A 113 8.018 -23.928 4.408 1.00 0.00 H

ATOM 1473 NZ LYS A 113 7.284 -24.486 6.339 1.00 0.00 N

ATOM 1474 1HZ LYS A 113 8.206 -24.729 6.685 1.00 0.00 H

ATOM 1475 2HZ LYS A 113 6.615 -25.125 6.733 1.00 0.00 H

ATOM 1476 3HZ LYS A 113 7.062 -23.544 6.633 1.00 0.00 H

ATOM 1477 N SER A 114 5.670 -26.084 -0.298 1.00 3.50 N

ATOM 1478 H SER A 114 6.246 -25.359 -0.705 1.00 3.50 H

ATOM 1479 CA SER A 114 6.058 -27.473 -0.560 1.00 3.85 C

ATOM 1480 HA SER A 114 5.245 -28.132 -0.247 1.00 3.85 H

ATOM 1481 C SER A 114 7.312 -27.836 0.224 1.00 3.48 C

ATOM 1482 O SER A 114 8.304 -27.113 0.188 1.00 5.07 O

ATOM 1483 CB SER A 114 6.320 -27.672 -2.055 1.00 4.32 C

ATOM 1484 1HB SER A 114 7.130 -27.016 -2.398 1.00 4.32 H

ATOM 1485 2HB SER A 114 6.609 -28.718 -2.211 1.00 4.32 H

ATOM 1486 OG SER A 114 5.165 -27.362 -2.812 1.00 5.06 O

ATOM 1487 HG SER A 114 5.371 -27.419 -3.751 1.00 5.06 H

ATOM 1488 N MET A 115 7.278 -28.976 0.915 1.00 3.82 N

ATOM 1489 H MET A 115 6.451 -29.553 0.901 1.00 3.82 H

ATOM 1490 CA MET A 115 8.418 -29.454 1.696 1.00 3.77 C

ATOM 1491 HA MET A 115 9.320 -28.917 1.431 1.00 3.77 H

ATOM 1492 C MET A 115 8.652 -30.934 1.416 1.00 3.75 C

ATOM 1493 O MET A 115 7.734 -31.747 1.465 1.00 4.60 O

ATOM 1494 CB MET A 115 8.133 -29.242 3.181 1.00 0.00 C

ATOM 1495 1HB MET A 115 7.923 -28.190 3.373 1.00 0.00 H

ATOM 1496 2HB MET A 115 7.241 -29.818 3.424 1.00 0.00 H

ATOM 1497 CG MET A 115 9.264 -29.678 4.104 1.00 0.00 C

ATOM 1498 1HG MET A 115 8.924 -29.605 5.141 1.00 0.00 H

ATOM 1499 2HG MET A 115 9.537 -30.707 3.854 1.00 0.00 H

ATOM 1500 SD MET A 115 10.745 -28.650 3.922 1.00 0.00 S

ATOM 1501 CE MET A 115 12.038 -29.896 3.801 1.00 0.00 C

ATOM 1502 1HE MET A 115 12.995 -29.394 3.667 1.00 0.00 H

ATOM 1503 2HE MET A 115 11.851 -30.548 2.948 1.00 0.00 H

ATOM 1504 3HE MET A 115 12.047 -30.495 4.711 1.00 0.00 H

ATOM 1505 N SER A 116 9.881 -31.342 1.117 1.00 3.70 N

ATOM 1506 H SER A 116 10.646 -30.684 1.102 1.00 3.70 H

ATOM 1507 CA SER A 116 10.199 -32.745 0.872 1.00 3.84 C

ATOM 1508 HA SER A 116 9.505 -33.356 1.443 1.00 3.84 H

ATOM 1509 C SER A 116 11.618 -33.110 1.304 1.00 4.67 C

ATOM 1510 O SER A 116 12.539 -32.292 1.226 1.00 4.48 O

ATOM 1511 CB SER A 116 10.021 -33.074 -0.606 1.00 4.96 C

ATOM 1512 1HB SER A 116 10.131 -34.156 -0.753 1.00 4.96 H

ATOM 1513 2HB SER A 116 9.015 -32.770 -0.929 1.00 4.96 H

ATOM 1514 OG SER A 116 10.973 -32.397 -1.405 1.00 5.59 O

ATOM 1515 HG SER A 116 10.614 -32.288 -2.292 1.00 5.59 H

ATOM 1516 N THR A 117 11.802 -34.338 1.788 1.00 4.69 N

ATOM 1517 H THR A 117 11.023 -34.984 1.840 1.00 4.69 H

ATOM 1518 CA THR A 117 13.096 -34.765 2.315 1.00 5.34 C

ATOM 1519 HA THR A 117 13.874 -34.080 1.971 1.00 5.34 H

ATOM 1520 C THR A 117 13.468 -36.167 1.854 1.00 5.71 C

ATOM 1521 O THR A 117 12.676 -37.102 1.980 1.00 5.86 O

ATOM 1522 CB THR A 117 13.084 -34.756 3.858 1.00 6.55 C

ATOM 1523 HB THR A 117 12.355 -35.483 4.230 1.00 6.55 H

ATOM 1524 OG1 THR A 117 12.665 -33.464 4.307 1.00 7.24 O

ATOM 1525 HG1 THR A 117 11.799 -33.315 3.912 1.00 7.24 H

ATOM 1526 CG2 THR A 117 14.452 -35.055 4.452 1.00 7.81 C

ATOM 1527 1HG2 THR A 117 14.421 -34.953 5.540 1.00 7.81 H

ATOM 1528 2HG2 THR A 117 15.193 -34.355 4.058 1.00 7.81 H

ATOM 1529 3HG2 THR A 117 14.776 -36.072 4.217 1.00 7.81 H

ATOM 1530 N GLY A 118 14.687 -36.313 1.343 1.00 6.42 N

ATOM 1531 H GLY A 118 15.277 -35.502 1.216 1.00 6.42 H

ATOM 1532 CA GLY A 118 15.203 -37.605 0.936 1.00 6.74 C

ATOM 1533 1HA GLY A 118 16.236 -37.716 1.298 1.00 6.74 H

ATOM 1534 2HA GLY A 118 14.589 -38.410 1.366 1.00 6.74 H

ATOM 1535 C GLY A 118 15.221 -37.765 -0.574 1.00 6.76 C

ATOM 1536 O GLY A 118 16.119 -37.265 -1.247 1.00 8.12 O

ATOM 1537 N HIS A 119 14.197 -38.425 -1.110 1.00 6.31 N

ATOM 1538 H HIS A 119 13.522 -38.877 -0.507 1.00 6.31 H

ATOM 1539 CA HIS A 119 14.000 -38.570 -2.554 1.00 7.22 C

ATOM 1540 HA HIS A 119 14.673 -37.898 -3.079 1.00 7.22 H

ATOM 1541 C HIS A 119 12.554 -38.183 -2.882 1.00 7.28 C

ATOM 1542 O HIS A 119 12.044 -38.428 -3.966 1.00 10.66 O

ATOM 1543 CB HIS A 119 14.270 -40.011 -2.985 1.00 0.00 C

ATOM 1544 1HB HIS A 119 13.461 -40.633 -2.600 1.00 0.00 H

ATOM 1545 2HB HIS A 119 14.242 -40.073 -4.074 1.00 0.00 H

ATOM 1546 CG HIS A 119 15.584 -40.546 -2.495 1.00 0.00 C

ATOM 1547 ND1 HIS A 119 16.816 -40.184 -3.009 1.00 0.00 N

ATOM 1548 HD1 HIS A 119 16.982 -39.522 -3.758 1.00 0.00 H

ATOM 1549 CD2 HIS A 119 15.874 -41.425 -1.495 1.00 0.00 C

ATOM 1550 HD2 HIS A 119 15.133 -41.898 -0.853 1.00 0.00 H

ATOM 1551 CE1 HIS A 119 17.760 -40.834 -2.308 1.00 0.00 C

ATOM 1552 HE1 HIS A 119 18.819 -40.708 -2.446 1.00 0.00 H

ATOM 1553 NE2 HIS A 119 17.227 -41.626 -1.392 1.00 0.00 N

ATOM 1554 N ALA A 120 11.887 -37.617 -1.877 1.00 6.27 N

ATOM 1555 H ALA A 120 12.405 -37.408 -1.034 1.00 6.27 H

ATOM 1556 CA ALA A 120 10.452 -37.353 -1.867 1.00 5.65 C

ATOM 1557 HA ALA A 120 9.951 -38.230 -2.276 1.00 5.65 H

ATOM 1558 C ALA A 120 10.072 -36.153 -2.732 1.00 5.32 C

ATOM 1559 O ALA A 120 10.877 -35.273 -3.006 1.00 6.32 O

ATOM 1560 CB ALA A 120 9.962 -37.140 -0.433 1.00 0.00 C

ATOM 1561 1HB ALA A 120 10.192 -38.034 0.155 1.00 0.00 H

ATOM 1562 2HB ALA A 120 8.881 -36.970 -0.424 1.00 0.00 H

ATOM 1563 3HB ALA A 120 10.478 -36.276 0.003 1.00 0.00 H

ATOM 1564 N ASP A 121 8.812 -36.122 -3.154 1.00 4.60 N

ATOM 1565 H ASP A 121 8.177 -36.860 -2.872 1.00 4.60 H

ATOM 1566 CA ASP A 121 8.292 -35.032 -3.969 1.00 4.61 C

ATOM 1567 HA ASP A 121 9.078 -34.287 -4.091 1.00 4.61 H

ATOM 1568 C ASP A 121 7.105 -34.343 -3.304 1.00 4.46 C

ATOM 1569 O ASP A 121 6.257 -34.982 -2.681 1.00 4.81 O

ATOM 1570 CB ASP A 121 7.885 -35.556 -5.345 1.00 0.00 C

ATOM 1571 1HB ASP A 121 7.152 -36.346 -5.209 1.00 0.00 H

ATOM 1572 2HB ASP A 121 7.426 -34.756 -5.918 1.00 0.00 H

ATOM 1573 CG ASP A 121 9.070 -36.094 -6.130 1.00 0.00 C

ATOM 1574 OD1 ASP A 121 10.076 -35.361 -6.292 1.00 0.00 O

ATOM 1575 OD2 ASP A 121 9.011 -37.250 -6.633 1.00 0.00 O

ATOM 1576 N ALA A 122 7.038 -33.024 -3.454 1.00 3.88 N

ATOM 1577 H ALA A 122 7.767 -32.529 -3.948 1.00 3.88 H

ATOM 1578 CA ALA A 122 5.945 -32.230 -2.911 1.00 3.86 C

ATOM 1579 HA ALA A 122 5.097 -32.900 -2.755 1.00 3.86 H

ATOM 1580 C ALA A 122 5.493 -31.127 -3.867 1.00 3.67 C

ATOM 1581 O ALA A 122 6.344 -30.410 -4.406 1.00 4.75 O

ATOM 1582 CB ALA A 122 6.335 -31.643 -1.566 1.00 4.25 C

ATOM 1583 1HB ALA A 122 6.600 -32.440 -0.878 1.00 4.25 H

ATOM 1584 2HB ALA A 122 5.495 -31.088 -1.161 1.00 4.25 H

ATOM 1585 3HB ALA A 122 7.168 -30.958 -1.678 1.00 4.25 H

ATOM 1586 N THR A 123 4.194 -31.005 -4.098 1.00 3.90 N

ATOM 1587 H THR A 123 3.536 -31.637 -3.669 1.00 3.90 H

ATOM 1588 CA THR A 123 3.665 -30.021 -5.038 1.00 4.12 C

ATOM 1589 HA THR A 123 4.432 -29.277 -5.292 1.00 4.12 H

ATOM 1590 C THR A 123 2.473 -29.295 -4.416 1.00 3.87 C

ATOM 1591 O THR A 123 1.526 -29.951 -3.985 1.00 4.48 O

ATOM 1592 CB THR A 123 3.198 -30.709 -6.330 1.00 5.25 C

ATOM 1593 HB THR A 123 2.357 -31.364 -6.097 1.00 5.25 H

ATOM 1594 OG1 THR A 123 4.258 -31.498 -6.864 1.00 6.91 O

ATOM 1595 HG1 THR A 123 4.741 -31.877 -6.122 1.00 6.91 H

ATOM 1596 CG2 THR A 123 2.721 -29.689 -7.364 1.00 6.15 C

ATOM 1597 1HG2 THR A 123 2.539 -30.204 -8.313 1.00 6.15 H

ATOM 1598 2HG2 THR A 123 3.479 -28.914 -7.512 1.00 6.15 H

ATOM 1599 3HG2 THR A 123 1.793 -29.213 -7.033 1.00 6.15 H

ATOM 1600 N THR A 124 2.507 -27.968 -4.403 1.00 3.77 N

ATOM 1601 H THR A 124 3.348 -27.492 -4.702 1.00 3.77 H

ATOM 1602 CA THR A 124 1.377 -27.163 -3.942 1.00 3.65 C

ATOM 1603 HA THR A 124 0.512 -27.804 -3.782 1.00 3.65 H

ATOM 1604 C THR A 124 0.978 -26.108 -4.965 1.00 3.37 C

ATOM 1605 O THR A 124 1.812 -25.589 -5.700 1.00 4.35 O

ATOM 1606 CB THR A 124 1.662 -26.475 -2.595 1.00 3.95 C

ATOM 1607 HB THR A 124 0.845 -25.799 -2.329 1.00 3.95 H

ATOM 1608 OG1 THR A 124 2.840 -25.659 -2.681 1.00 5.08 O

ATOM 1609 HG1 THR A 124 3.595 -26.227 -2.897 1.00 5.08 H

ATOM 1610 CG2 THR A 124 1.879 -27.489 -1.501 1.00 4.33 C

ATOM 1611 1HG2 THR A 124 1.856 -26.999 -0.526 1.00 4.33 H

ATOM 1612 2HG2 THR A 124 2.842 -27.996 -1.596 1.00 4.33 H

ATOM 1613 3HG2 THR A 124 1.108 -28.262 -1.522 1.00 4.33 H

ATOM 1614 N ASN A 125 -0.323 -25.807 -4.997 1.00 3.37 N

ATOM 1615 H ASN A 125 -0.962 -26.328 -4.412 1.00 3.37 H

ATOM 1616 CA ASN A 125 -0.834 -24.700 -5.798 1.00 3.75 C

ATOM 1617 HA ASN A 125 -0.072 -23.926 -5.886 1.00 3.75 H

ATOM 1618 C ASN A 125 -2.080 -24.114 -5.146 1.00 3.54 C

ATOM 1619 O ASN A 125 -3.010 -24.845 -4.801 1.00 5.79 O

ATOM 1620 CB ASN A 125 -1.189 -25.205 -7.200 1.00 0.00 C

ATOM 1621 1HB ASN A 125 -0.328 -25.720 -7.612 1.00 0.00 H

ATOM 1622 2HB ASN A 125 -2.016 -25.902 -7.118 1.00 0.00 H

ATOM 1623 CG ASN A 125 -1.600 -24.078 -8.129 1.00 0.00 C

ATOM 1624 OD1 ASN A 125 -2.721 -23.583 -8.072 1.00 0.00 O

ATOM 1625 ND2 ASN A 125 -0.683 -23.678 -9.008 1.00 0.00 N

ATOM 1626 1HD2 ASN A 125 -0.875 -22.898 -9.622 1.00 0.00 H

ATOM 1627 2HD2 ASN A 125 0.194 -24.161 -9.091 1.00 0.00 H

ATOM 1628 N ALA A 126 -2.083 -22.790 -4.973 1.00 3.83 N

ATOM 1629 H ALA A 126 -1.288 -22.232 -5.263 1.00 3.83 H

ATOM 1630 CA ALA A 126 -3.209 -22.112 -4.363 1.00 4.10 C

ATOM 1631 HA ALA A 126 -4.119 -22.691 -4.528 1.00 4.10 H

ATOM 1632 C ALA A 126 -3.403 -20.740 -5.002 1.00 3.90 C

ATOM 1633 O ALA A 126 -2.422 -20.046 -5.269 1.00 4.80 O

ATOM 1634 CB ALA A 126 -2.992 -21.975 -2.865 1.00 5.06 C

ATOM 1635 1HB ALA A 126 -3.074 -22.942 -2.376 1.00 5.06 H

ATOM 1636 2HB ALA A 126 -3.757 -21.318 -2.460 1.00 5.06 H

ATOM 1637 3HB ALA A 126 -2.021 -21.541 -2.644 1.00 5.06 H

ATOM 1638 N HIS A 127 -4.656 -20.366 -5.270 1.00 4.51 N

ATOM 1639 H HIS A 127 -5.423 -20.966 -5.009 1.00 4.51 H

ATOM 1640 CA HIS A 127 -4.941 -19.100 -5.945 1.00 5.31 C

ATOM 1641 HA HIS A 127 -4.127 -18.409 -5.739 1.00 5.31 H

ATOM 1642 C HIS A 127 -6.227 -18.443 -5.443 1.00 5.30 C

ATOM 1643 O HIS A 127 -7.175 -19.123 -5.033 1.00 5.92 O

ATOM 1644 CB HIS A 127 -4.969 -19.322 -7.463 1.00 0.00 C

ATOM 1645 1HB HIS A 127 -5.288 -18.410 -7.963 1.00 0.00 H

ATOM 1646 2HB HIS A 127 -3.957 -19.569 -7.774 1.00 0.00 H

ATOM 1647 CG HIS A 127 -5.868 -20.429 -7.911 1.00 0.00 C

ATOM 1648 ND1 HIS A 127 -5.507 -21.752 -7.964 1.00 0.00 N

ATOM 1649 HD1 HIS A 127 -4.597 -22.126 -7.730 1.00 0.00 H

ATOM 1650 CD2 HIS A 127 -7.159 -20.396 -8.337 1.00 0.00 C

ATOM 1651 HD2 HIS A 127 -7.767 -19.495 -8.406 1.00 0.00 H

ATOM 1652 CE1 HIS A 127 -6.557 -22.448 -8.400 1.00 0.00 C

ATOM 1653 HE1 HIS A 127 -6.572 -23.523 -8.541 1.00 0.00 H

ATOM 1654 NE2 HIS A 127 -7.585 -21.663 -8.664 1.00 0.00 N

ATOM 1655 N GLY A 128 -6.227 -17.122 -5.452 1.00 5.31 N

ATOM 1656 H GLY A 128 -5.443 -16.632 -5.861 1.00 5.31 H

ATOM 1657 CA GLY A 128 -7.270 -16.362 -4.803 1.00 6.16 C

ATOM 1658 1HA GLY A 128 -7.546 -15.521 -5.424 1.00 6.16 H

ATOM 1659 2HA GLY A 128 -8.128 -16.998 -4.645 1.00 6.16 H

ATOM 1660 C GLY A 128 -6.754 -15.871 -3.458 1.00 5.64 C

ATOM 1661 O GLY A 128 -5.858 -15.035 -3.398 1.00 6.88 O

ATOM 1662 N THR A 129 -7.347 -16.400 -2.399 1.00 6.21 N

ATOM 1663 H THR A 129 -8.167 -16.985 -2.511 1.00 6.21 H

ATOM 1664 CA THR A 129 -6.814 -16.254 -1.044 1.00 6.87 C

ATOM 1665 HA THR A 129 -5.835 -15.777 -1.101 1.00 6.87 H

ATOM 1666 C THR A 129 -6.618 -17.612 -0.375 1.00 6.33 C

ATOM 1667 O THR A 129 -6.423 -17.700 0.839 1.00 6.73 O

ATOM 1668 CB THR A 129 -7.727 -15.384 -0.155 1.00 8.01 C

ATOM 1669 HB THR A 129 -7.322 -15.324 0.861 1.00 8.01 H

ATOM 1670 OG1 THR A 129 -9.036 -15.956 -0.101 1.00 9.95 O

ATOM 1671 HG1 THR A 129 -9.542 -15.500 0.571 1.00 9.95 H

ATOM 1672 CG2 THR A 129 -7.858 -13.959 -0.717 1.00 9.91 C

ATOM 1673 1HG2 THR A 129 -8.331 -13.343 0.044 1.00 9.91 H

ATOM 1674 2HG2 THR A 129 -8.479 -13.959 -1.612 1.00 9.91 H

ATOM 1675 3HG2 THR A 129 -6.875 -13.549 -0.940 1.00 9.91 H

ATOM 1676 N ALA A 130 -6.651 -18.659 -1.195 1.00 5.67 N

ATOM 1677 H ALA A 130 -6.765 -18.496 -2.185 1.00 5.67 H

ATOM 1678 CA ALA A 130 -6.569 -20.051 -0.753 1.00 5.03 C

ATOM 1679 HA ALA A 130 -7.278 -20.221 0.067 1.00 5.03 H

ATOM 1680 C ALA A 130 -5.169 -20.405 -0.255 1.00 4.55 C

ATOM 1681 O ALA A 130 -4.201 -19.686 -0.505 1.00 6.17 O

ATOM 1682 CB ALA A 130 -6.972 -20.955 -1.916 1.00 5.98 C

ATOM 1683 1HB ALA A 130 -7.977 -20.684 -2.238 1.00 5.98 H

ATOM 1684 2HB ALA A 130 -6.954 -21.994 -1.592 1.00 5.98 H

ATOM 1685 3HB ALA A 130 -6.256 -20.820 -2.727 1.00 5.98 H

ATOM 1686 N MET A 131 -5.063 -21.537 0.447 1.00 5.02 N

ATOM 1687 H MET A 131 -5.885 -22.111 0.590 1.00 5.02 H

ATOM 1688 CA MET A 131 -3.796 -22.018 0.979 1.00 5.27 C

ATOM 1689 HA MET A 131 -3.008 -21.407 0.552 1.00 5.27 H

ATOM 1690 C MET A 131 -3.600 -23.495 0.627 1.00 4.56 C

ATOM 1691 O MET A 131 -4.538 -24.290 0.715 1.00 5.78 O

ATOM 1692 CB MET A 131 -3.768 -21.844 2.511 1.00 0.00 C

ATOM 1693 1HB MET A 131 -4.081 -20.825 2.744 1.00 0.00 H

ATOM 1694 2HB MET A 131 -4.491 -22.531 2.952 1.00 0.00 H

ATOM 1695 CG MET A 131 -2.401 -22.091 3.154 1.00 0.00 C

ATOM 1696 1HG MET A 131 -2.143 -23.135 2.960 1.00 0.00 H

ATOM 1697 2HG MET A 131 -1.672 -21.421 2.693 1.00 0.00 H

ATOM 1698 SD MET A 131 -2.422 -21.807 4.949 1.00 0.00 S

ATOM 1699 CE MET A 131 -3.127 -23.333 5.577 1.00 0.00 C

ATOM 1700 1HE MET A 131 -3.311 -23.210 6.650 1.00 0.00 H

ATOM 1701 2HE MET A 131 -2.490 -24.209 5.408 1.00 0.00 H

ATOM 1702 3HE MET A 131 -4.095 -23.472 5.085 1.00 0.00 H

ATOM 1703 N ALA A 132 -2.378 -23.855 0.250 1.00 4.40 N

ATOM 1704 H ALA A 132 -1.654 -23.160 0.152 1.00 4.40 H

ATOM 1705 CA ALA A 132 -2.039 -25.247 -0.043 1.00 4.40 C

ATOM 1706 HA ALA A 132 -2.786 -25.911 0.396 1.00 4.40 H

ATOM 1707 C ALA A 132 -0.671 -25.636 0.529 1.00 4.18 C

ATOM 1708 O ALA A 132 0.333 -24.985 0.243 1.00 4.82 O

ATOM 1709 CB ALA A 132 -2.054 -25.443 -1.552 1.00 5.16 C

ATOM 1710 1HB ALA A 132 -3.081 -25.466 -1.924 1.00 5.16 H

ATOM 1711 2HB ALA A 132 -1.571 -26.394 -1.789 1.00 5.16 H

ATOM 1712 3HB ALA A 132 -1.513 -24.628 -2.040 1.00 5.16 H

ATOM 1713 N ASP A 133 -0.626 -26.681 1.353 1.00 4.36 N

ATOM 1714 H ASP A 133 -1.467 -27.214 1.544 1.00 4.36 H

ATOM 1715 CA ASP A 133 0.619 -27.087 2.014 1.00 4.45 C

ATOM 1716 HA ASP A 133 1.468 -26.574 1.557 1.00 4.45 H

ATOM 1717 C ASP A 133 0.837 -28.591 1.882 1.00 4.26 C

ATOM 1718 O ASP A 133 -0.106 -29.373 1.988 1.00 5.49 O

ATOM 1719 CB ASP A 133 0.560 -26.674 3.482 1.00 0.00 C

ATOM 1720 1HB ASP A 133 -0.303 -27.150 3.964 1.00 0.00 H

ATOM 1721 2HB ASP A 133 1.477 -27.010 3.980 1.00 0.00 H

ATOM 1722 CG ASP A 133 0.450 -25.174 3.660 1.00 0.00 C

ATOM 1723 OD1 ASP A 133 1.136 -24.434 2.936 1.00 0.00 O

ATOM 1724 OD2 ASP A 133 -0.333 -24.707 4.516 1.00 0.00 O

ATOM 1725 N SER A 134 2.082 -29.007 1.626 1.00 3.54 N

ATOM 1726 H SER A 134 2.833 -28.332 1.525 1.00 3.54 H

ATOM 1727 CA SER A 134 2.402 -30.423 1.456 1.00 3.40 C

ATOM 1728 HA SER A 134 1.656 -30.989 2.006 1.00 3.40 H

ATOM 1729 C SER A 134 3.765 -30.817 2.011 1.00 3.84 C

ATOM 1730 O SER A 134 4.741 -30.073 1.922 1.00 4.30 O

ATOM 1731 CB SER A 134 2.284 -30.822 -0.012 1.00 4.27 C

ATOM 1732 1HB SER A 134 2.928 -30.196 -0.628 1.00 4.27 H

ATOM 1733 2HB SER A 134 2.623 -31.852 -0.116 1.00 4.27 H

ATOM 1734 OG SER A 134 0.960 -30.698 -0.469 1.00 5.04 O

ATOM 1735 HG SER A 134 0.513 -30.068 0.108 1.00 5.04 H

ATOM 1736 N ASN A 135 3.805 -32.007 2.600 1.00 3.67 N

ATOM 1737 H ASN A 135 2.972 -32.578 2.647 1.00 3.67 H

ATOM 1738 CA ASN A 135 5.010 -32.546 3.220 1.00 3.84 C

ATOM 1739 HA ASN A 135 5.875 -32.001 2.839 1.00 3.84 H

ATOM 1740 C ASN A 135 5.213 -34.013 2.876 1.00 4.05 C

ATOM 1741 O ASN A 135 4.349 -34.851 3.113 1.00 5.18 O

ATOM 1742 CB ASN A 135 4.940 -32.381 4.739 1.00 4.39 C

ATOM 1743 1HB ASN A 135 4.654 -31.360 4.973 1.00 4.39 H

ATOM 1744 2HB ASN A 135 4.178 -33.038 5.150 1.00 4.39 H

ATOM 1745 CG ASN A 135 6.261 -32.676 5.399 1.00 4.83 C

ATOM 1746 OD1 ASN A 135 7.301 -32.175 4.983 1.00 6.01 O

ATOM 1747 ND2 ASN A 135 6.229 -33.494 6.437 1.00 6.14 N

ATOM 1748 1HD2 ASN A 135 7.101 -33.734 6.895 1.00 6.14 H

ATOM 1749 2HD2 ASN A 135 5.340 -33.838 6.768 1.00 6.14 H

ATOM 1750 N ALA A 136 6.380 -34.346 2.319 1.00 3.89 N

ATOM 1751 H ALA A 136 7.059 -33.629 2.103 1.00 3.89 H

ATOM 1752 CA ALA A 136 6.683 -35.724 1.933 1.00 3.82 C

ATOM 1753 HA ALA A 136 5.951 -36.398 2.379 1.00 3.82 H

ATOM 1754 C ALA A 136 8.070 -36.131 2.417 1.00 4.18 C

ATOM 1755 O ALA A 136 9.051 -35.417 2.235 1.00 4.88 O

ATOM 1756 CB ALA A 136 6.591 -35.848 0.429 1.00 4.50 C

ATOM 1757 1HB ALA A 136 5.657 -35.394 0.092 1.00 4.50 H

ATOM 1758 2HB ALA A 136 6.607 -36.898 0.133 1.00 4.50 H

ATOM 1759 3HB ALA A 136 7.424 -35.317 -0.036 1.00 4.50 H

ATOM 1760 N ILE A 137 8.151 -37.283 3.075 1.00 4.79 N

ATOM 1761 H ILE A 137 7.331 -37.863 3.184 1.00 4.79 H

ATOM 1762 CA ILE A 137 9.406 -37.736 3.675 1.00 5.53 C

ATOM 1763 HA ILE A 137 10.193 -37.054 3.380 1.00 5.53 H

ATOM 1764 C ILE A 137 9.792 -39.134 3.195 1.00 6.16 C

ATOM 1765 O ILE A 137 9.002 -40.065 3.347 1.00 9.89 O

ATOM 1766 CB ILE A 137 9.352 -37.780 5.207 1.00 6.75 C

ATOM 1767 HB ILE A 137 8.587 -38.491 5.504 1.00 6.75 H

ATOM 1768 CG1 ILE A 137 9.017 -36.378 5.753 1.00 8.73 C

ATOM 1769 1HG1 ILE A 137 9.854 -35.697 5.577 1.00 8.73 H

ATOM 1770 2HG1 ILE A 137 8.131 -36.016 5.228 1.00 8.73 H

ATOM 1771 CG2 ILE A 137 10.697 -38.269 5.760 1.00 8.69 C

ATOM 1772 1HG2 ILE A 137 10.728 -38.166 6.840 1.00 8.69 H

ATOM 1773 2HG2 ILE A 137 11.539 -37.736 5.326 1.00 8.69 H

ATOM 1774 3HG2 ILE A 137 10.801 -39.325 5.529 1.00 8.69 H

ATOM 1775 CD1 ILE A 137 8.651 -36.342 7.236 1.00 10.77 C

ATOM 1776 1HD1 ILE A 137 8.241 -35.355 7.473 1.00 10.77 H

ATOM 1777 2HD1 ILE A 137 9.544 -36.506 7.847 1.00 10.77 H

ATOM 1778 3HD1 ILE A 137 7.916 -37.128 7.442 1.00 10.77 H

ATOM 1779 N GLY A 138 10.995 -39.287 2.649 1.00 5.90 N

ATOM 1780 H GLY A 138 11.620 -38.493 2.563 1.00 5.90 H

ATOM 1781 CA GLY A 138 11.464 -40.574 2.164 1.00 5.96 C

ATOM 1782 1HA GLY A 138 12.480 -40.762 2.503 1.00 5.96 H

ATOM 1783 2HA GLY A 138 10.819 -41.362 2.546 1.00 5.96 H

ATOM 1784 C GLY A 138 11.471 -40.631 0.651 1.00 5.73 C

ATOM 1785 O GLY A 138 12.168 -39.861 0.003 1.00 6.97 O

ATOM 1786 N GLU A 139 10.673 -41.543 0.089 1.00 5.47 N

ATOM 1787 H GLU A 139 10.129 -42.176 0.666 1.00 5.47 H

ATOM 1788 CA GLU A 139 10.411 -41.582 -1.341 1.00 5.26 C

ATOM 1789 HA GLU A 139 11.024 -40.823 -1.822 1.00 5.26 H

ATOM 1790 C GLU A 139 8.943 -41.224 -1.562 1.00 4.99 C

ATOM 1791 O GLU A 139 8.332 -41.563 -2.576 1.00 6.03 O

ATOM 1792 CB GLU A 139 10.721 -42.979 -1.906 1.00 0.00 C

ATOM 1793 1HB GLU A 139 10.132 -43.736 -1.381 1.00 0.00 H

ATOM 1794 2HB GLU A 139 10.447 -42.980 -2.964 1.00 0.00 H

ATOM 1795 CG GLU A 139 12.185 -43.389 -1.798 1.00 0.00 C

ATOM 1796 1HG GLU A 139 12.364 -44.216 -2.478 1.00 0.00 H

ATOM 1797 2HG GLU A 139 12.803 -42.552 -2.108 1.00 0.00 H

ATOM 1798 CD GLU A 139 12.594 -43.823 -0.398 1.00 0.00 C

ATOM 1799 OE1 GLU A 139 13.529 -43.236 0.186 1.00 0.00 O

ATOM 1800 OE2 GLU A 139 11.998 -44.770 0.145 1.00 0.00 O

ATOM 1801 N ALA A 140 8.394 -40.536 -0.567 1.00 4.84 N

ATOM 1802 H ALA A 140 8.972 -40.259 0.210 1.00 4.84 H

ATOM 1803 CA ALA A 140 6.979 -40.195 -0.504 1.00 5.06 C

ATOM 1804 HA ALA A 140 6.368 -41.068 -0.736 1.00 5.06 H

ATOM 1805 C ALA A 140 6.591 -39.105 -1.500 1.00 4.86 C

ATOM 1806 O ALA A 140 7.418 -38.339 -2.006 1.00 5.96 O

ATOM 1807 CB ALA A 140 6.639 -39.752 0.921 1.00 0.00 C

ATOM 1808 1HB ALA A 140 7.004 -40.491 1.637 1.00 0.00 H

ATOM 1809 2HB ALA A 140 5.558 -39.670 1.023 1.00 0.00 H

ATOM 1810 3HB ALA A 140 7.111 -38.793 1.132 1.00 0.00 H

ATOM 1811 N ARG A 141 5.297 -39.036 -1.771 1.00 4.82 N

ATOM 1812 H ARG A 141 4.655 -39.663 -1.306 1.00 4.82 H

ATOM 1813 CA ARG A 141 4.739 -38.049 -2.691 1.00 4.66 C

ATOM 1814 HA ARG A 141 5.470 -37.255 -2.867 1.00 4.66 H

ATOM 1815 C ARG A 141 3.473 -37.425 -2.118 1.00 4.06 C

ATOM 1816 O ARG A 141 2.500 -38.119 -1.839 1.00 5.28 O

ATOM 1817 CB ARG A 141 4.387 -38.673 -4.038 1.00 0.00 C

ATOM 1818 1HB ARG A 141 3.654 -39.463 -3.866 1.00 0.00 H

ATOM 1819 2HB ARG A 141 3.921 -37.909 -4.664 1.00 0.00 H

ATOM 1820 CG ARG A 141 5.581 -39.250 -4.784 1.00 0.00 C

ATOM 1821 1HG ARG A 141 6.256 -38.445 -5.068 1.00 0.00 H

ATOM 1822 2HG ARG A 141 6.126 -39.924 -4.125 1.00 0.00 H

ATOM 1823 CD ARG A 141 5.136 -40.018 -6.025 1.00 0.00 C

ATOM 1824 1HD ARG A 141 4.491 -39.373 -6.608 1.00 0.00 H

ATOM 1825 2HD ARG A 141 6.006 -40.241 -6.629 1.00 0.00 H

ATOM 1826 NE ARG A 141 4.427 -41.250 -5.668 1.00 0.00 N

ATOM 1827 HE ARG A 141 3.417 -41.222 -5.698 1.00 0.00 H

ATOM 1828 CZ ARG A 141 4.987 -42.392 -5.282 1.00 0.00 C

ATOM 1829 NH1 ARG A 141 4.211 -43.406 -4.960 1.00 0.00 N

ATOM 1830 1HH1 ARG A 141 3.217 -43.332 -5.087 1.00 0.00 H

ATOM 1831 2HH1 ARG A 141 4.628 -44.240 -4.558 1.00 0.00 H

ATOM 1832 NH2 ARG A 141 6.293 -42.582 -5.272 1.00 0.00 N

ATOM 1833 1HH2 ARG A 141 6.897 -41.835 -5.544 1.00 0.00 H

ATOM 1834 2HH2 ARG A 141 6.655 -43.489 -5.019 1.00 0.00 H

ATOM 1835 N ALA A 142 3.478 -36.110 -1.938 1.00 3.77 N

ATOM 1836 H ALA A 142 4.278 -35.561 -2.204 1.00 3.77 H

ATOM 1837 CA ALA A 142 2.335 -35.403 -1.371 1.00 3.95 C

ATOM 1838 HA ALA A 142 1.483 -36.079 -1.353 1.00 3.95 H

ATOM 1839 C ALA A 142 2.007 -34.190 -2.222 1.00 4.03 C

ATOM 1840 O ALA A 142 2.869 -33.343 -2.438 1.00 4.55 O

ATOM 1841 CB ALA A 142 2.666 -34.942 0.038 1.00 4.86 C

ATOM 1842 1HB ALA A 142 2.915 -35.797 0.658 1.00 4.86 H

ATOM 1843 2HB ALA A 142 1.833 -34.380 0.450 1.00 4.86 H

ATOM 1844 3HB ALA A 142 3.539 -34.298 0.004 1.00 4.86 H

ATOM 1845 N GLU A 143 0.773 -34.116 -2.720 1.00 4.06 N

ATOM 1846 H GLU A 143 0.083 -34.819 -2.496 1.00 4.06 H

ATOM 1847 CA GLU A 143 0.386 -33.020 -3.598 1.00 4.16 C

ATOM 1848 HA GLU A 143 1.126 -32.227 -3.489 1.00 4.16 H

ATOM 1849 C GLU A 143 -0.994 -32.465 -3.265 1.00 3.71 C

ATOM 1850 O GLU A 143 -1.938 -33.222 -3.077 1.00 5.04 O

ATOM 1851 CB GLU A 143 0.353 -33.510 -5.050 1.00 0.00 C

ATOM 1852 1HB GLU A 143 -0.411 -34.290 -5.122 1.00 0.00 H

ATOM 1853 2HB GLU A 143 0.063 -32.662 -5.679 1.00 0.00 H

ATOM 1854 CG GLU A 143 1.654 -34.103 -5.583 1.00 0.00 C

ATOM 1855 1HG GLU A 143 2.475 -33.426 -5.365 1.00 0.00 H

ATOM 1856 2HG GLU A 143 1.846 -35.055 -5.095 1.00 0.00 H

ATOM 1857 CD GLU A 143 1.586 -34.305 -7.088 1.00 0.00 C

ATOM 1858 OE1 GLU A 143 1.346 -33.316 -7.816 1.00 0.00 O

ATOM 1859 OE2 GLU A 143 1.778 -35.442 -7.554 1.00 0.00 O

ATOM 1860 N THR A 144 -1.127 -31.142 -3.190 1.00 3.92 N

ATOM 1861 H THR A 144 -0.329 -30.531 -3.318 1.00 3.92 H

ATOM 1862 CA THR A 144 -2.437 -30.566 -2.912 1.00 3.64 C

ATOM 1863 HA THR A 144 -3.207 -31.276 -3.247 1.00 3.64 H

ATOM 1864 C THR A 144 -2.707 -29.246 -3.628 1.00 3.55 C

ATOM 1865 O THR A 144 -1.797 -28.488 -3.968 1.00 3.99 O

ATOM 1866 CB THR A 144 -2.625 -30.388 -1.383 1.00 4.71 C

ATOM 1867 HB THR A 144 -2.255 -31.266 -0.851 1.00 4.71 H

ATOM 1868 OG1 THR A 144 -4.021 -30.276 -1.093 1.00 5.62 O

ATOM 1869 HG1 THR A 144 -4.480 -31.044 -1.446 1.00 5.62 H

ATOM 1870 CG2 THR A 144 -1.924 -29.132 -0.861 1.00 4.99 C

ATOM 1871 1HG2 THR A 144 -1.883 -29.148 0.223 1.00 4.99 H

ATOM 1872 2HG2 THR A 144 -2.501 -28.272 -1.180 1.00 4.99 H

ATOM 1873 3HG2 THR A 144 -0.921 -29.070 -1.269 1.00 4.99 H

ATOM 1874 N ARG A 145 -3.989 -29.003 -3.913 1.00 3.62 N

ATOM 1875 H ARG A 145 -4.685 -29.689 -3.636 1.00 3.62 H

ATOM 1876 CA ARG A 145 -4.435 -27.820 -4.660 1.00 3.84 C

ATOM 1877 HA ARG A 145 -3.609 -27.112 -4.718 1.00 3.84 H

ATOM 1878 C ARG A 145 -5.617 -27.132 -3.978 1.00 3.78 C

ATOM 1879 O ARG A 145 -6.476 -27.823 -3.437 1.00 5.73 O

ATOM 1880 CB ARG A 145 -4.844 -28.246 -6.075 1.00 0.00 C

ATOM 1881 1HB ARG A 145 -5.619 -29.001 -5.979 1.00 0.00 H

ATOM 1882 2HB ARG A 145 -5.276 -27.388 -6.581 1.00 0.00 H

ATOM 1883 CG ARG A 145 -3.701 -28.763 -6.942 1.00 0.00 C

ATOM 1884 1HG ARG A 145 -2.903 -28.021 -6.947 1.00 0.00 H

ATOM 1885 2HG ARG A 145 -3.296 -29.679 -6.512 1.00 0.00 H

ATOM 1886 CD ARG A 145 -4.181 -29.037 -8.355 1.00 0.00 C

ATOM 1887 1HD ARG A 145 -4.680 -28.151 -8.732 1.00 0.00 H

ATOM 1888 2HD ARG A 145 -3.330 -29.220 -9.001 1.00 0.00 H

ATOM 1889 NE ARG A 145 -5.135 -30.151 -8.448 1.00 0.00 N

ATOM 1890 HE ARG A 145 -6.112 -29.921 -8.362 1.00 0.00 H

ATOM 1891 CZ ARG A 145 -4.845 -31.435 -8.622 1.00 0.00 C

ATOM 1892 NH1 ARG A 145 -5.801 -32.332 -8.656 1.00 0.00 N

ATOM 1893 1HH1 ARG A 145 -6.758 -32.029 -8.531 1.00 0.00 H

ATOM 1894 2HH1 ARG A 145 -5.565 -33.298 -8.791 1.00 0.00 H

ATOM 1895 NH2 ARG A 145 -3.603 -31.848 -8.812 1.00 0.00 N

ATOM 1896 1HH2 ARG A 145 -2.840 -31.209 -8.729 1.00 0.00 H

ATOM 1897 2HH2 ARG A 145 -3.446 -32.793 -9.137 1.00 0.00 H

ATOM 1898 N ALA A 146 -5.667 -25.809 -4.012 1.00 3.83 N

ATOM 1899 H ALA A 146 -4.932 -25.297 -4.476 1.00 3.83 H

ATOM 1900 CA ALA A 146 -6.748 -25.057 -3.372 1.00 4.33 C

ATOM 1901 HA ALA A 146 -7.610 -25.719 -3.258 1.00 4.33 H

ATOM 1902 C ALA A 146 -7.185 -23.856 -4.218 1.00 4.58 C

ATOM 1903 O ALA A 146 -6.372 -23.263 -4.935 1.00 5.22 O

ATOM 1904 CB ALA A 146 -6.303 -24.607 -1.995 1.00 5.11 C

ATOM 1905 1HB ALA A 146 -5.906 -25.449 -1.423 1.00 5.11 H

ATOM 1906 2HB ALA A 146 -7.131 -24.164 -1.436 1.00 5.11 H

ATOM 1907 3HB ALA A 146 -5.515 -23.856 -2.084 1.00 5.11 H

ATOM 1908 N GLU A 147 -8.462 -23.505 -4.153 1.00 4.38 N

ATOM 1909 H GLU A 147 -9.082 -23.987 -3.512 1.00 4.38 H

ATOM 1910 CA GLU A 147 -9.053 -22.443 -4.967 1.00 5.21 C

ATOM 1911 HA GLU A 147 -8.275 -21.784 -5.344 1.00 5.21 H

ATOM 1912 C GLU A 147 -10.050 -21.604 -4.166 1.00 5.34 C

ATOM 1913 O GLU A 147 -10.821 -22.136 -3.365 1.00 7.10 O

ATOM 1914 CB GLU A 147 -9.758 -23.094 -6.162 1.00 0.00 C

ATOM 1915 1HB GLU A 147 -9.025 -23.644 -6.756 1.00 0.00 H

ATOM 1916 2HB GLU A 147 -10.472 -23.814 -5.763 1.00 0.00 H

ATOM 1917 CG GLU A 147 -10.524 -22.164 -7.092 1.00 0.00 C

ATOM 1918 1HG GLU A 147 -11.300 -21.650 -6.521 1.00 0.00 H

ATOM 1919 2HG GLU A 147 -9.850 -21.395 -7.471 1.00 0.00 H

ATOM 1920 CD GLU A 147 -11.150 -22.920 -8.253 1.00 0.00 C

ATOM 1921 OE1 GLU A 147 -10.945 -24.147 -8.356 1.00 0.00 O

ATOM 1922 OE2 GLU A 147 -11.906 -22.293 -9.025 1.00 0.00 O

ATOM 1923 N GLY A 148 -10.048 -20.291 -4.380 1.00 5.76 N

ATOM 1924 H GLY A 148 -9.390 -19.888 -5.033 1.00 5.76 H

ATOM 1925 CA GLY A 148 -10.976 -19.416 -3.673 1.00 6.31 C

ATOM 1926 1HA GLY A 148 -11.189 -18.544 -4.290 1.00 6.31 H

ATOM 1927 2HA GLY A 148 -11.908 -19.950 -3.494 1.00 6.31 H

ATOM 1928 C GLY A 148 -10.399 -18.942 -2.353 1.00 7.98 C

ATOM 1929 O GLY A 148 -9.406 -18.216 -2.355 1.00 11.24 O

ATOM 1930 N ARG A 149 -11.012 -19.359 -1.250 1.00 9.76 N

ATOM 1931 H ARG A 149 -11.870 -19.892 -1.337 1.00 9.76 H

ATOM 1932 CA ARG A 149 -10.464 -19.152 0.081 1.00 9.18 C

ATOM 1933 HA ARG A 149 -9.448 -18.768 -0.026 1.00 9.18 H

ATOM 1934 C ARG A 149 -10.384 -20.495 0.800 1.00 8.26 C

ATOM 1935 O ARG A 149 -10.421 -20.567 2.030 1.00 10.36 O

ATOM 1936 CB ARG A 149 -11.269 -18.139 0.899 1.00 0.00 C

ATOM 1937 1HB ARG A 149 -10.706 -17.928 1.817 1.00 0.00 H

ATOM 1938 2HB ARG A 149 -11.337 -17.189 0.357 1.00 0.00 H

ATOM 1939 CG ARG A 149 -12.672 -18.568 1.299 1.00 0.00 C

ATOM 1940 1HG ARG A 149 -13.308 -18.589 0.412 1.00 0.00 H

ATOM 1941 2HG ARG A 149 -12.678 -19.574 1.724 1.00 0.00 H

ATOM 1942 CD ARG A 149 -13.249 -17.586 2.301 1.00 0.00 C

ATOM 1943 1HD ARG A 149 -12.632 -17.552 3.204 1.00 0.00 H

ATOM 1944 2HD ARG A 149 -13.253 -16.582 1.863 1.00 0.00 H

ATOM 1945 NE ARG A 149 -14.616 -17.940 2.663 1.00 0.00 N

ATOM 1946 HE ARG A 149 -15.346 -17.428 2.199 1.00 0.00 H

ATOM 1947 CZ ARG A 149 -14.986 -18.865 3.539 1.00 0.00 C

ATOM 1948 NH1 ARG A 149 -16.272 -19.010 3.802 1.00 0.00 N

ATOM 1949 1HH1 ARG A 149 -16.958 -18.414 3.355 1.00 0.00 H

ATOM 1950 2HH1 ARG A 149 -16.578 -19.759 4.423 1.00 0.00 H

ATOM 1951 NH2 ARG A 149 -14.120 -19.646 4.154 1.00 0.00 N

ATOM 1952 1HH2 ARG A 149 -13.136 -19.529 3.978 1.00 0.00 H

ATOM 1953 2HH2 ARG A 149 -14.475 -20.368 4.759 1.00 0.00 H

ATOM 1954 N ALA A 150 -10.314 -21.567 0.005 1.00 5.73 N

ATOM 1955 H ALA A 150 -10.324 -21.432 -0.997 1.00 5.73 H

ATOM 1956 CA ALA A 150 -10.272 -22.932 0.518 1.00 6.16 C

ATOM 1957 HA ALA A 150 -11.007 -23.060 1.312 1.00 6.16 H

ATOM 1958 C ALA A 150 -8.884 -23.230 1.078 1.00 6.39 C

ATOM 1959 O ALA A 150 -7.915 -22.560 0.747 1.00 9.87 O

ATOM 1960 CB ALA A 150 -10.586 -23.926 -0.606 1.00 7.46 C

ATOM 1961 1HB ALA A 150 -11.610 -23.741 -0.940 1.00 7.46 H

ATOM 1962 2HB ALA A 150 -10.498 -24.959 -0.261 1.00 7.46 H

ATOM 1963 3HB ALA A 150 -9.887 -23.751 -1.427 1.00 7.46 H

ATOM 1964 N GLU A 151 -8.751 -24.278 1.884 1.00 5.49 N

ATOM 1965 H GLU A 151 -9.568 -24.808 2.154 1.00 5.49 H

ATOM 1966 CA GLU A 151 -7.463 -24.706 2.416 1.00 5.70 C

ATOM 1967 HA GLU A 151 -6.653 -24.198 1.892 1.00 5.70 H

ATOM 1968 C GLU A 151 -7.346 -26.213 2.206 1.00 5.07 C

ATOM 1969 O GLU A 151 -8.264 -26.966 2.520 1.00 6.51 O

ATOM 1970 CB GLU A 151 -7.355 -24.393 3.910 1.00 0.00 C

ATOM 1971 1HB GLU A 151 -8.152 -24.927 4.413 1.00 0.00 H

ATOM 1972 2HB GLU A 151 -6.401 -24.775 4.254 1.00 0.00 H

ATOM 1973 CG GLU A 151 -7.446 -22.905 4.271 1.00 0.00 C

ATOM 1974 1HG GLU A 151 -6.643 -22.338 3.803 1.00 0.00 H

ATOM 1975 2HG GLU A 151 -8.399 -22.506 3.924 1.00 0.00 H

ATOM 1976 CD GLU A 151 -7.355 -22.700 5.766 1.00 0.00 C

ATOM 1977 OE1 GLU A 151 -6.465 -23.264 6.427 1.00 0.00 O

ATOM 1978 OE2 GLU A 151 -8.214 -21.951 6.290 1.00 0.00 O

ATOM 1979 N SER A 152 -6.214 -26.642 1.664 1.00 4.56 N

ATOM 1980 H SER A 152 -5.474 -25.985 1.455 1.00 4.56 H

ATOM 1981 CA SER A 152 -5.960 -28.049 1.365 1.00 4.26 C

ATOM 1982 HA SER A 152 -6.695 -28.655 1.890 1.00 4.26 H

ATOM 1983 C SER A 152 -4.570 -28.466 1.846 1.00 4.41 C

ATOM 1984 O SER A 152 -3.655 -27.640 1.905 1.00 5.01 O

ATOM 1985 CB SER A 152 -6.085 -28.315 -0.139 1.00 0.00 C

ATOM 1986 1HB SER A 152 -5.275 -27.829 -0.667 1.00 0.00 H

ATOM 1987 2HB SER A 152 -6.023 -29.379 -0.320 1.00 0.00 H

ATOM 1988 OG SER A 152 -7.315 -27.805 -0.636 1.00 0.00 O

ATOM 1989 HG SER A 152 -7.498 -26.961 -0.219 1.00 0.00 H

ATOM 1990 N SER A 153 -4.397 -29.731 2.197 1.00 4.24 N

ATOM 1991 H SER A 153 -5.172 -30.375 2.149 1.00 4.24 H

ATOM 1992 CA SER A 153 -3.123 -30.222 2.721 1.00 4.02 C

ATOM 1993 HA SER A 153 -2.319 -29.625 2.294 1.00 4.02 H

ATOM 1994 C SER A 153 -2.859 -31.676 2.366 1.00 3.64 C

ATOM 1995 O SER A 153 -3.785 -32.470 2.229 1.00 4.07 O

ATOM 1996 CB SER A 153 -3.062 -30.057 4.241 1.00 5.86 C

ATOM 1997 1HB SER A 153 -2.088 -30.383 4.599 1.00 5.86 H

ATOM 1998 2HB SER A 153 -3.207 -29.010 4.505 1.00 5.86 H

ATOM 1999 OG SER A 153 -4.069 -30.846 4.861 1.00 8.31 O

ATOM 2000 HG SER A 153 -4.919 -30.551 4.524 1.00 8.31 H

ATOM 2001 N SER A 154 -1.587 -32.054 2.232 1.00 3.19 N

ATOM 2002 H SER A 154 -0.849 -31.368 2.303 1.00 3.19 H

ATOM 2003 CA SER A 154 -1.184 -33.429 1.976 1.00 3.31 C

ATOM 2004 HA SER A 154 -1.941 -34.098 2.360 1.00 3.31 H

ATOM 2005 C SER A 154 0.123 -33.772 2.691 1.00 3.20 C

ATOM 2006 O SER A 154 1.095 -33.024 2.633 1.00 4.21 O

ATOM 2007 CB SER A 154 -1.069 -33.679 0.475 1.00 0.00 C

ATOM 2008 1HB SER A 154 -2.018 -33.456 -0.009 1.00 0.00 H

ATOM 2009 2HB SER A 154 -0.314 -33.009 0.072 1.00 0.00 H

ATOM 2010 OG SER A 154 -0.713 -35.025 0.236 1.00 0.00 O

ATOM 2011 HG SER A 154 -1.421 -35.603 0.531 1.00 0.00 H

ATOM 2012 N ASP A 155 0.147 -34.890 3.416 1.00 3.77 N

ATOM 2013 H ASP A 155 -0.648 -35.515 3.408 1.00 3.77 H

ATOM 2014 CA ASP A 155 1.272 -35.234 4.273 1.00 3.70 C

ATOM 2015 HA ASP A 155 2.163 -34.697 3.948 1.00 3.70 H

ATOM 2016 C ASP A 155 1.524 -36.738 4.171 1.00 3.56 C

ATOM 2017 O ASP A 155 0.655 -37.554 4.463 1.00 4.14 O

ATOM 2018 CB ASP A 155 0.957 -34.845 5.719 1.00 0.00 C

ATOM 2019 1HB ASP A 155 0.034 -35.347 6.032 1.00 0.00 H

ATOM 2020 2HB ASP A 155 1.761 -35.226 6.361 1.00 0.00 H

ATOM 2021 CG ASP A 155 0.775 -33.358 5.897 1.00 0.00 C

ATOM 2022 OD1 ASP A 155 1.711 -32.608 5.534 1.00 0.00 O

ATOM 2023 OD2 ASP A 155 -0.277 -32.915 6.409 1.00 0.00 O

ATOM 2024 N THR A 156 2.733 -37.094 3.726 1.00 4.00 N

ATOM 2025 H THR A 156 3.404 -36.367 3.515 1.00 4.00 H

ATOM 2026 CA THR A 156 3.089 -38.488 3.483 1.00 4.05 C

ATOM 2027 HA THR A 156 2.388 -39.140 4.009 1.00 4.05 H

ATOM 2028 C THR A 156 4.498 -38.844 3.946 1.00 4.53 C

ATOM 2029 O THR A 156 5.382 -37.986 3.989 1.00 5.04 O

ATOM 2030 CB THR A 156 3.023 -38.839 1.980 1.00 4.38 C

ATOM 2031 HB THR A 156 3.302 -39.877 1.830 1.00 4.38 H

ATOM 2032 OG1 THR A 156 3.927 -37.975 1.277 1.00 4.76 O

ATOM 2033 HG1 THR A 156 3.688 -37.052 1.413 1.00 4.76 H

ATOM 2034 CG2 THR A 156 1.617 -38.647 1.449 1.00 4.70 C

ATOM 2035 1HG2 THR A 156 1.560 -38.983 0.417 1.00 4.70 H

ATOM 2036 2HG2 THR A 156 1.370 -37.588 1.507 1.00 4.70 H

ATOM 2037 3HG2 THR A 156 0.911 -39.224 2.045 1.00 4.70 H

ATOM 2038 N ASP A 157 4.716 -40.111 4.289 1.00 4.17 N

ATOM 2039 H ASP A 157 3.956 -40.771 4.355 1.00 4.17 H

ATOM 2040 CA ASP A 157 6.068 -40.613 4.517 1.00 5.03 C

ATOM 2041 HA ASP A 157 6.803 -40.062 3.934 1.00 5.03 H

ATOM 2042 C ASP A 157 6.111 -42.061 4.039 1.00 4.60 C

ATOM 2043 O ASP A 157 5.085 -42.738 4.022 1.00 4.61 O

ATOM 2044 CB ASP A 157 6.456 -40.520 5.999 1.00 0.00 C

ATOM 2045 1HB ASP A 157 7.494 -40.813 6.113 1.00 0.00 H

ATOM 2046 2HB ASP A 157 6.356 -39.496 6.339 1.00 0.00 H

ATOM 2047 CG ASP A 157 5.605 -41.398 6.889 1.00 0.00 C

ATOM 2048 OD1 ASP A 157 4.485 -40.982 7.272 1.00 0.00 O

ATOM 2049 OD2 ASP A 157 6.063 -42.503 7.235 1.00 0.00 O

ATOM 2050 N GLY A 158 7.285 -42.508 3.605 1.00 4.87 N

ATOM 2051 H GLY A 158 8.097 -41.909 3.665 1.00 4.87 H

ATOM 2052 CA GLY A 158 7.423 -43.798 2.967 1.00 5.13 C

ATOM 2053 1HA GLY A 158 8.298 -44.329 3.354 1.00 5.13 H

ATOM 2054 2HA GLY A 158 6.540 -44.416 3.152 1.00 5.13 H

ATOM 2055 C GLY A 158 7.596 -43.657 1.463 1.00 4.98 C

ATOM 2056 O GLY A 158 8.646 -43.230 0.983 1.00 6.31 O

ATOM 2057 N CYS A 159 6.535 -43.974 0.713 1.00 4.82 N

ATOM 2058 H CYS A 159 5.697 -44.315 1.170 1.00 4.82 H

ATOM 2059 CA CYS A 159 6.538 -43.837 -0.746 1.00 5.36 C

ATOM 2060 HA CYS A 159 7.135 -42.959 -1.019 1.00 5.36 H

ATOM 2061 C CYS A 159 5.143 -43.613 -1.319 1.00 4.76 C

ATOM 2062 O CYS A 159 4.906 -42.546 -1.927 1.00 5.73 O

ATOM 2063 OXT CYS A 159 4.300 -44.509 -1.155 1.00 5.73 O

ATOM 2064 CB CYS A 159 7.183 -45.074 -1.367 1.00 0.00 C

ATOM 2065 1HB CYS A 159 8.105 -45.308 -0.838 1.00 0.00 H

ATOM 2066 2HB CYS A 159 6.527 -45.937 -1.271 1.00 0.00 H

ATOM 2067 SG CYS A 159 7.549 -44.819 -3.110 1.00 0.00 S

ATOM 2068 HG CYS A 159 8.048 -43.587 -3.004 1.00 0.00 H

TER 2069 CYS A 159

END
